# Orbital eccentricity and internal feedbacks drove the Triassic megamonsoon variability

**DOI:** 10.1038/s41598-025-09295-2

**Published:** 2025-07-07

**Authors:** Runjian Chu, Huaichun Wu, Jian Zhang, Qiang Fang, Christian Zeeden, Peng Chen, Rukai Zhu, Jingwei Cui, Shihong Zhang, Tianshui Yang, Chengshan Wang

**Affiliations:** 1 State Key Laboratory of Geomicrobiology and Environmental Changes, China University of Geosciences, Beijing, 100083 China; 2 Frontiers Science Center for Deep-Time Digital Earth, China University of Geosciences (Beijing), Beijing, 100083 China; 3 Key Laboratory of Polar Geology and Marine Mineral Resources, School of Ocean Science, China University of Geosciences (Beijing), Beijing, 100083 China; 4 State Key Laboratory of Tibetan Plateau Earth System, Environment and Resources (TPESER), Chinese Academy of Sciences, Institute of Tibetan Plateau Research, Beijing, 100101 China; 5 LIAG-Leibniz Institute for Applied Geophysics, Stilleweg 2, 30655 Hannover, Germany; 6 Research Institute of Petroleum Exploration & Development, PetroChina, Beijing, 100083 China

**Keywords:** Climate sciences, Environmental sciences, Hydrology

## Abstract

The evolution of the Triassic megamonsoon was closely linked to Earth’s orbital variations. Despite recognizing secular orbital cycles as a fundamental pacemaker of the megamonsoon, the driving mechanisms remain unclear. Here, we use data-model synthesis to study orbital-scale megamonsoon variability during the Middle Triassic (~ 246–239 Ma). By integrating high-resolution reconstructions of hydrologic fluctuations, obtained from lithological and magnetic susceptibility data series in the lacustrine sediments of the Ordos Basin (Northeast Tethys), with the climate simulations, we identify monsoon cycles in the ~ 20, 100, and 405 kyr Milankovitch bands. Comparisons with other records further reveal an additional eccentricity-related ~ 3.3 Myr orbital cycle in monsoon variabilities, temperature oscillations, carbon cycles, and sea-level changes. Earth system models show the effects of orbital configurations and atmospheric CO₂ concentrations on megamonsoon dynamics, implying threshold responses to solar radiation and the impacts of temperature and sea-level fluctuations on long-term megamonsoon variability. These findings improve our understanding of the interplay between astronomical forcing and feedbacks in shaping orbital-scale monsoon dynamics.

## Introduction

Monsoon, as one of the most important large-scale climate systems, exert a profound impact on the livelihoods of billions of people today through influencing the hydrologic cycle, making their evolution and driving mechanisms central topics of study within the geoscience community^1^. The Triassic period is a particularly captivating geological interval of a greenhouse world for examining monsoon systems. Over tectonic timescales, the convergence of continents resulted in the formation of the supercontinent Pangea, which reached its peak during the Triassic and caused a megamonsoon system^2–6^. This megamonsoon system offers a deep-time case study that allows us to understand the characteristics and evolutionary processes of modern regional monsoon systems exhibiting analogous broad monsoonal circulations associated with extensive landmasses like Eurasia and Africa^1^. On orbital timescales, climate variations of today’s Earth are primarily dictated by high-latitude ice sheets and low-latitude monsoons (Milankovitch-Kutzbach hypothesis)^1,7–11^. In contrast, the reduced prevalence of glaciers during the Triassic, a period characterized by the highest atmospheric CO₂ concentrations of the Phanerozoic^12^, decoupled orbital-scale megamonsoon variability from glacial cycles^9,10^, thus offering a unique window to study monsoon behavior under high CO₂ levels. Therefore, understanding the orbital-scale megamonsoon variabilities is crucial for comprehending monsoon systems from a geological perspective, providing insights into Earth’s climate dynamics throughout its past and potentially into its future.

**Fig. 1:**
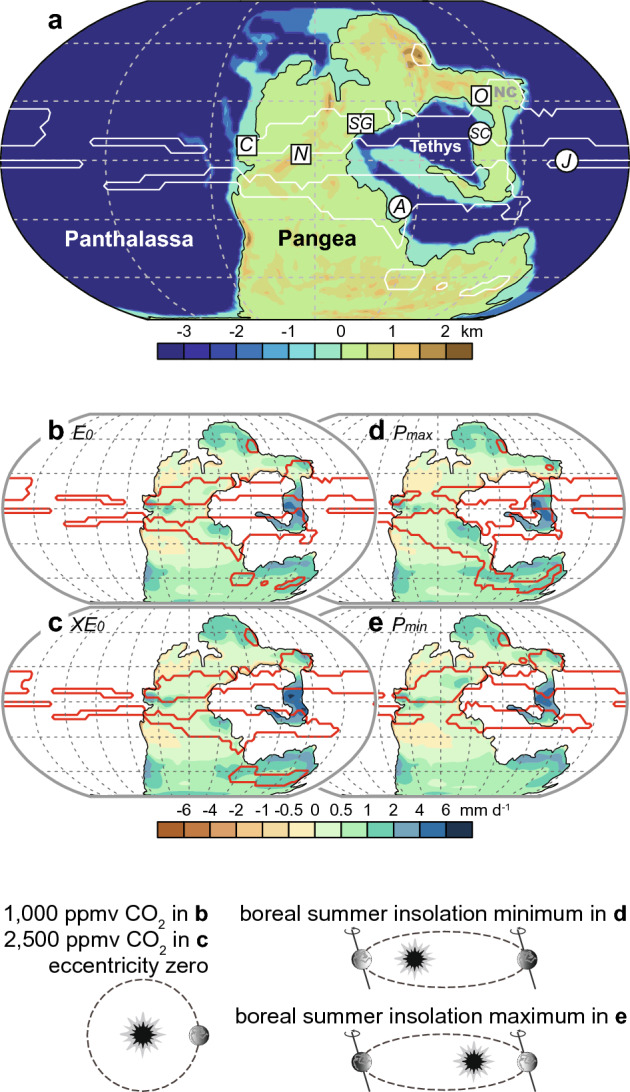
Simulated global monsoon regions and geological records with well-preserved orbital-scale megamonsoonal dynamics. (**a**) Paleogeography of the ~ 250 Ma Triassic world^42^ and simulated monsoon regions (outlined in white) in the simulations of *E*_*0*_ in panel (**b**) illustrating the location of the Ordos Basin and other sites mentioned in the text. White squares denote terrestrial settings, and white circles denote marine settings. Names: C = Colorado Plateau and Castile Evaporite Basin; N = Newark Basin; S = St. Audrie’s Bay; G = Germanic Basin; O = Ordos Basin; SC = South China; J = BSi data from the deep-sea bedded chert sequence at Inuyama, Japan; A = Arabian Platform. (**b**–**e**) Maps of global monsoon regions (outlined in red) and annual mean net precipitation (shaded; units: mm d^−1^) for the simulation experiments *E*_*0*_ (**b**), *XE*_*0*_ (**c**),* P*_*max*_ (**d**), and *P*_*min*_ (**e**).

Triassic megamonsoon variability has been extensively documented in sedimentary successions across Pangea and the superocean Panthalassa^13–26^ (Fig. 1a). Prominent examples include the ~ 40 Myr record of lake level fluctuations in the Newark Basin (North America)^13–16^ and the ~ 70 Myr record of biogenic silica (BSi) burial fluxes in pelagic deep-sea bedded chert sequences in Japan^17–21^. These long, high-resolution records not only capture conventional orbital cycles ranging from 10^4^ to 10^5^ years, with pronounced precession-related signals, but also exhibit a series of secular frequencies of the orbits—known as the orbital grand cycles—such as the ~ 1.75 Myr and ~ 3.3–3.6 Myr cycles^15,20,27,28^. These distinctive expressions of orbital grand cycles during the Triassic, contrasting with the present ~ 2.4 Myr eccentricity cycle and/or ∼1.2 Myr obliquity cycle, have attracted considerable attention concerning their astronomical origins and implications for pre-Cenozoic solar system evolution^15,20,27–29^. However, despite the recognition of orbital grand cycles as a fundamental pacemaker of monsoon hydrologic changes during the Triassic, the mechanisms underlying these hydrologic dynamics remain a critical knowledge gap in megamonsoon research. Existing theories, such as the Kutzbach^30^ orbital monsoon hypothesis, which attributes monsoon variations to summer insolation changes driven primarily by precession cycles, cannot fully explain the monsoon dynamics when coupled with the secular frequencies of orbits. Given that the contribution of orbital grand cycles to insolation change is considerably smaller compared to that of precession, attributing the megamonsoon variability solely to orbital forcing seems challenging^31,32^.

The unique setting of the Tethys region, a vast warm oceanic gulf that penetrated the low latitudes of eastern Pangea, makes it an ideal location to study megamonsoon dynamics (Fig. 1a). Similar to today’s Indo-Pacific Warm Pool, the Tethys Ocean acted as a ‘heat and moisture engine’ that drove the megamonsoon system^2,4,33–36^. However, monsoon variability in the Tethys region is less understood compared to coeval Pangea and Panthalassa (Fig. 1a), largely due to the rarity of long, well-dated proxy records. China, located at the northeastern edge of the largest seasonal swing of the Intertropical Convergence Zone within the Tethys region during the Triassic, exhibited a distinctive sedimentary arrangement with land in the north and ocean in the south, offering a unique opportunity to unravel monsoon variability in this region (Fig. 1 and Supplementary Fig. S1). Marine records from South China provided valuable insights into orbital-scale variabilities and ecological implications in the Tethys region^37–39^, yet they offer limited insight into continental responses. Terrestrial records, which respond exclusively to regional atmospheric circulation, are often better suited to address fundamental questions regarding the monsoon dynamics. Thus, precisely dated paleoclimate records from continental locations in North China would provide key information on the processes and mechanisms of Tethys regional monsoon.

The Ordos Basin, situated on the southwestern margin of the North China Craton (the largest craton in the northeast Pangaea) during the Triassic^40^ (Fig. 1a), contains fluvial-lacustrine-deltaic sequences, i.e., the Yanchang Formation, which hold significant potential for preserving a record of monsoon hydrology in the Tethys region^26^. The depositional environment of the Yanchang Formation was primarily driven by the hydrologic budget within the Ordos Basin, as geochemical evidence shows that the lake system remained freshwater^41^, precluding marine incursions. Here we present a continuous hydrologic record for the Middle Triassic by analyzing lithological variations and paleoenvironmental proxy series from the Yanchang Formation to improve our understanding of long-term, orbital-scale climate variability and dynamics in the Tethys region during the Middle Triassic. We bring these records into a global perspective through correlation in order to investigate the potential orbital grand cycles in the hydroclimate changes. We also performed simulations with an Earth System model under Triassic boundary conditions to evaluate the megamonsoon dynamics in response to orbital and CO₂ forcings, with a focus on the Ordos Basin and Tethys region.

## Results

Samples of the Yanchang Formation sediments were obtained from the Yaoye-1 drill core (35°13’N, 109°01’E) located in the Weibei Uplift of the southern Ordos Basin (Supplementary Fig. S1). Zircon grains from a bentonite layer at a depth of 243.36 m in the Yaoye-1 core were dated using high-precision U–Pb chemical abrasion-isotope dilution-thermal ionization mass spectrometry (CA-ID-TIMS), yielding a weighted mean age of 241.36 ± 0.12 Ma (2σ level)^43^. The stratigraphic interval between 150 and 405 m was selected due to its relatively stable internal structure during deposition without distinct sedimentary discontinuity. Two primary depositional environments are evident: lacustrine deposits characterized by grey-black mudstone/shale-dominant layers with horizontal lamination (333.2–300 m and 241.3–200 m), and deltaic environments primarily composed of sandstones (Supplementary Fig. S1). Notably, the upper lacustrine sediments, referred to as the Zhangjiatan Shale, comprise organic-rich black shales that represent promising hydrocarbon source rocks and were deposited in a deep lacustrine environment. Our previous study has visually identified ~ 100 kyr eccentricity and ~ 20 kyr precession cycles within these shales using multi-proxy data series^26^ (Supplementary Fig. S2). In this study, we integrated high-resolution lithological (Supplementary Table S1), magnetic susceptibility (MS) (Supplementary Table S2), and Fe/Ba (Supplementary Table S3) datasets with paleoclimate simulations to reconstruct the monsoon hydrologic evolution during the Middle Triassic.

### Lithology and proxy fluctuations indicate orbital hydrologic variability

**Fig. 2:**
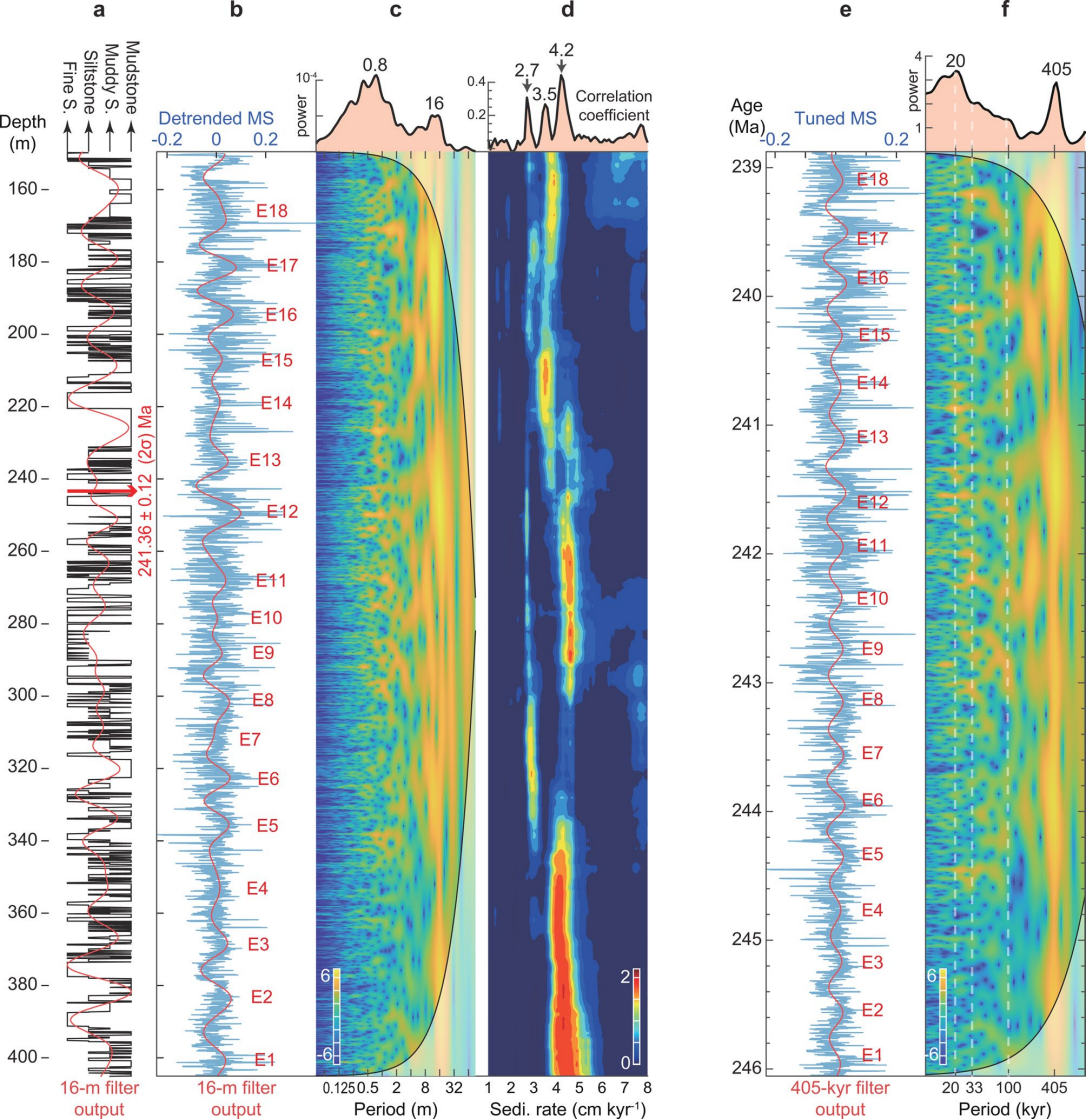
Cyclostratigraphic analysis at Yaoye-1 (150–405 m). (**a**) Stratigraphic column of the Yaoye-1 core illustrating the lithological variations and 16 m filtered cycles (passbands: 0.0625 ± 0.03 cycles m^−1^). The U–Pb age of 241.36 ± 0.12 Ma is reported by Cui, et al.^43^. (**b**) Magnetic susceptibility (MS) series after removing a 40 m ‘loess’ trend showing 16 m filtered cycles (passbands: 0.0625 ± 0.03 cycles m^−1^). (**c**) Wavelet power spectral analysis of the MS series. (**d**) COCO and eCOCO sedimentation rate map with null hypothesis (*H*_0_) of the MS series at Yaoye-1. (**e**) 405 kyr tuned MS series showing 405 kyr filtered cycles (passbands: 0.00247 ± 0.0005 cycles kyr^−1^). (**f**) Wavelet power spectral analysis of the tuned MS series. Note that both the 405 and 20 kyr cyclical signals exceed the 5% false discovery rate (FDR) confidence level (CL) (5% FDR CL = 99.99804% Chi-squared CL; Supplementary Fig. S3).

Cyclicity is visually evident in the lithological variations (or lithological index related to water depth) in the Yanchang Formation at Yaoye-1, as alternations of mudstone and sandstone beds; this quasi-cyclical pattern is captured by the lithological index and MS datasets (Fig. 2 and Supplementary Fig. S3). The evolution of MS data in the studied succession exhibits a strong correlation with both redox environments and lithological variations (Fig. 2a,b and Supplementary Fig. S3). MS data exhibit lower average values during the anoxic interval (~ 243–208 m in depth), which is distinguished by elevated gamma-ray (GR) values and uranium (U) concentrations^44^, compared to other intervals (Fig. 2a,b and Supplementary Fig. S1). This is attributed to the transition from preservation to dissolution of detrital magnetic minerals in anoxic environments^44^. Despite the influence of anoxic conditions, oscillations in MS through the succession reflect bed-scale alternations between mudstone, muddy siltstone, siltstone, and fine sandstone (Fig. 2a,b), which can be interpreted as a proxy for the degree of hydroclimate on the land surface during deposition. Mudstones and organic-rich black shales represent deeper water depth environments, while lithological combinations primarily consisting of siltstones and fine sandstones suggest a shallower water depth environment. Fluctuations in lithological and MS series can thus reflect climatic variability in precipitation and evaporation.

Visible 4 and 0.8 m-thick cycles of the deltaic and lacustrine sequences at Yaoye-1, between 197.85 and 258.4 m, were interpreted based on frequency ratios of statistically significant spectral peaks as sedimentological expressions of the ~ 100 kyr eccentricity and ~ 20 kyr precession cycles, respectively^26,44^ (Supplementary Fig. S2). Thus the ~ 16 m cycle detected spectrally in the MS and lithological series (Fig. 2c and Supplementary Fig. S3) from the whole 150–405 m interval was interpreted as sedimentological expressions of the 405 kyr eccentricity cycle. Statistical methods further support this interpretation, showing an optimal sedimentation rate of 4.2 cm kyr^-1^ by correlation coefficient (COCO) analysis for the MS series (Fig. 2d). Moreover, TimeOpt analysis suggests a discrepancy between the optimal sedimentation rates determined by ${r}_{spectral}^{2}$ and ${r}_{envelope}^{2}$, with estimates of 4.2 and 5.7 cm kyr^−1^, respectively (Supplementary Fig. S4). Based on the estimate of 4.2 cm kyr^−1^, the prominent wavelengths in the spectral analysis of the untuned MS data can be interpreted as Milankovitch imprints: a 405 kyr long-eccentricity cycle (16 m), a ~ 100 kyr short-eccentricity cycle (5.7–7.3 m), a ~ 33 kyr obliquity cycle (1.3–1.6 m), and a ~ 20 kyr precession cycle (0.72–0.9 m) (Supplementary Fig. S3). The eCOCO reveals a relatively uniform sedimentation rate at this site, with relatively higher values observed within the deltaic intervals compared to those in the lacustrine intervals (Fig. 2d).

### Establishing and testing a radioisotopically anchored astrochronology

The orbital cycles identified in the MS series enable the construction of an astronomical timescale. The ~ 16 m cycles were filtered and adjusted to align with the 405 kyr eccentricity cycles (Fig. 2e and Supplementary Fig. S3). Given the relatively stable frequency of the 405 kyr eccentricity cycle over geological time, which is minimally influenced by the chaotic behavior of the solar system^31,45^, this approach represents a general strategy for pre-Cenozoic astrochronologic construction^46^. The floating timescale was further anchored by the U–Pb age of 241.36 ± 0.12 Ma at the depth of 243.36 m^43^. Consequently, a total of eighteen 405 kyr cycles were identified, spanning from ~ 246 to 239 Ma (Fig. 2e). Wavelet power spectra of the tuned MS series also show the presence of 405 kyr long eccentricity, ~ 100 kyr short eccentricity, ~ 33 kyr obliquity, and ~ 20 kyr precession cycles across the studied interval, with strong signals at 405 kyr eccentricity and 20 kyr precession (Fig. 2f). Notably, both the 405 kyr eccentricity and 20 kyr precession cycles are statistically significant in the power spectral analysis, as evidenced by a false discovery rate (FDR) below 5% (Supplementary Fig. S3).

The uncertainty in our age-depth model is influenced by two primary factors: the precision of the CA-ID-TIMS U–Pb dating method (with an uncertainty of 0.12 Myr at the 2σ level^43^) and the methodology of astronomical tuning. The main sources of astrochronologic uncertainty include the choice of tuning strategy, sedimentation rate variations, and possible hiatuses^47^. Specifically, our application of the 405 kyr tuning is constrained by both statistical methods (COCO and TimeOpt) and visual cycle-pattern recognition applied to the 259–197 m interval at Yaoye-1^44^ (Supplementary Fig. S2). The 405 kyr tuning can be influenced by climate proxy record and filtering parameters. However, the consistent results of the 405 kyr tuning for both lithological and MS series, as well as the tuning based on the ~ 100 kyr eccentricity and ~ 20 kyr precession cycles, further reinforce the reliability of our astrochronology (Supplementary Fig. S3). Although independent age constraints for Yaoye-1 are unavailable, a comparable CA-ID-TIMS U–Pb dating result of 4.7 ± 1.4 cm kyr^-1^ (uncertainty calculated by combining errors in quadrature) was obtained from the nearby Yishicun Section^26,48^ (Supplementary Fig. S1). This rate is also similar to the ~ 5 cm kyr^−1^ derived from magnetostratigraphy in the Early Triassic Liujiagou Formation^49^ and the ~ 7 cm kyr^−1^ derived from CA-ID-TIMS U–Pb dating estimates from the Middle Jurassic Yan’an Formation^50^, both of which share a similar environment with the Yanchang Formation in the Ordos Basin. The preservation of orbital cycles across various depositional environments (i.e., deltaic and lacustrine environments) and their transitions^44^ suggests a high degree of stratigraphic completeness at Yaoye-1 with no signs of major disconformities (Fig. 2 and Supplementary Figs. S2 and S3).

**Fig. 3:**
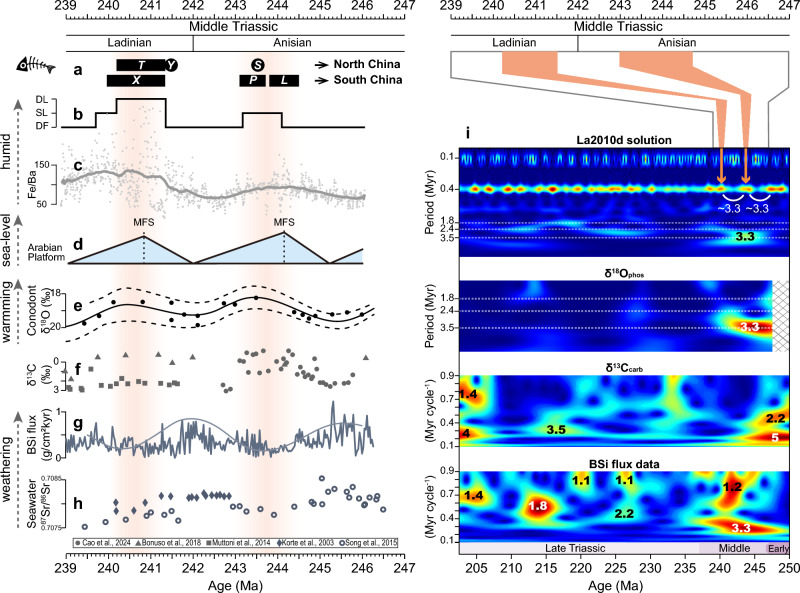
Comparison of fossils and sedimentary environments in the Ordos Basin with global and regional geological records during the Middle Triassic. (**a**) Fossil assemblages found in North China and South China. Names: T = Tongchuan Biota^51^, Y = Yonghesuchus (241.48 ± 0.13 Ma)^52^, S = Sinokannmeyeria (243.53 ± 0.13 Ma)^52^; X = Xingyi Biota (240.65 ± 0.69 Ma)^53^, P = Panxian Biota (243.4 Ma)^54^, L = Luoping Biota (243.8 Ma)^54^. (**b**) depositional environments at Yaoye-1. *DL* deep lacustrine subfacies, *SL* shallow lacustrine subfacies, *DF* delta facies. (**c**) Fe/Ba data series. The thick gray line is the 1,000 kyr “moving” trend of the (gray dots) measured values. (**d**) Sequence stratigraphic cycles from the Arabian Platform^55^. *MFS* maximum flooding surfaces. (**e**) Compilation of sea surface temperature during the Middle Triassic based on conodont apatite δ^18^O record^56^. The solid black line represents the Locfit analysis of δ^18^O data, with dashed lines bracketing the ± 95% confidence interval. (**f**) Integrated carbonate δ^13^C of marine Sections^57–59^. (**g**) Silicate weathering rate from BSi flux in the pelagic Inuyama section, showing a discerned ~ 3.3 Myr cycle^19,20^. (**h**) Conodont apatite ^87^Sr/^86^Sr record^60,61^. (**i**) Comparison of multi-Myr cycles in geological records and astronomical solutions during the Triassic. Wavelet analysis of astronomical solution La2010d eccentricity (top panel)^32^ indicates that the wavelet amplitude (red for peaks; blue for valleys) within the ~ 100 and 405 kyr eccentricity frequency bands is modulated by a period of ~ 3.3 Myr for the Middle Triassic. Wavelet analysis of δ^18^O data series^56^ and evolutionary spectra of δ^13^C and BSi flux data series^20^ reveal significant ~ 3.3 Myr cycles during the Middle Triassic in both the δ^18^O and BSi flux data series.

Based on the newly developed age-depth model, the two lacustrine intervals (~ 244.1–243.2 Ma and ~ 241.3–240 Ma) correspond to the Anisian and Ladinian stages, respectively (Fig. 3b). These lacustrine intervals are also characterized by higher Fe/Ba ratios, which suggest relatively humid conditions during these periods (Fig. 3c). Notably, the Zhangjiatan Shale (~ 241.3–240 Ma) is consistent with U–Pb dating ages (242.1, 240.9, 240.3, and 240.1 Ma) for this interval reported from nearby outcrops (Supplementary Fig. S1) where abundant fossils have been discovered^48,51^. The fossil assemblage in these sediments documents a Mesozoic-type, trophically multileveled lacustrine ecosystem, representing the earliest known complex lacustrine ecosystem that developed following the Permian–Triassic mass extinction (PTME)^51^.

### Hydroclimate simulations

To provide context for the hydroclimate conditions in the Ordos Basin and to explore the driving mechanisms behind the observed changes in the sedimentary environment, we conducted Triassic climate simulations with the Community Earth System Model (CESM). The simulations reveal a spatially extensive monsoon system, particularly over the circum-Tethys region (Fig. 1), which generally aligns with previous modeling results^2,4,62^. The paleogeographic distribution of sedimentary archives of orbital-scale megamonsoon dynamics, extending from the western to eastern and low-to-mid latitude regions of Pangea (e.g., Colorado Plateau^23^, Castile Evaporite Basin^22^, Newark Basin^14^, St. Audrie’s Bay^25^, and Germanic Basin^24^), generally aligns with our simulated monsoon domain (Fig. 1).

**Fig. 4:**
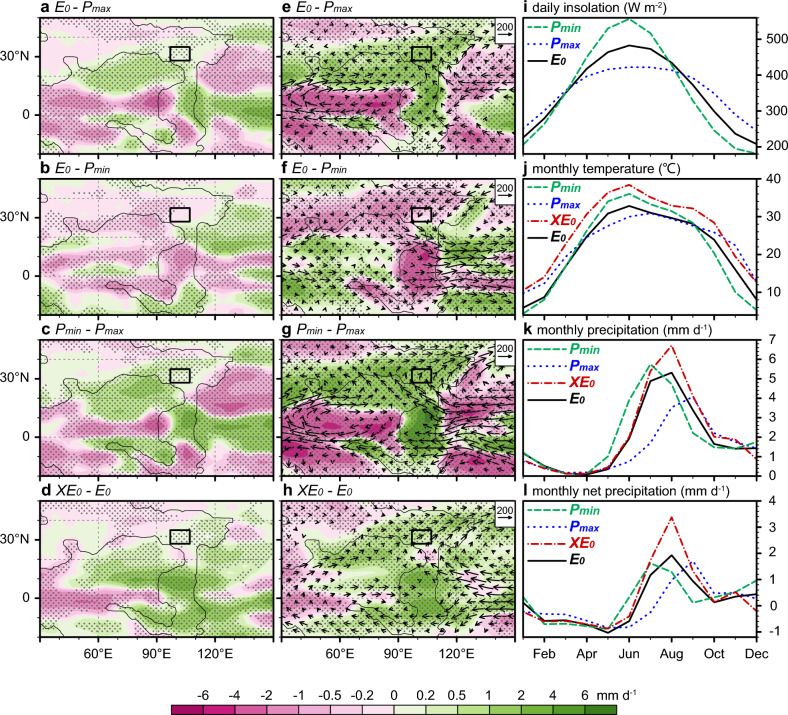
Simulated hydroclimate responses in the northeast Tethys to eccentricity, precession, and CO_2_ variations during the Triassic. Left panels (**a**–**d**) Annual mean net precipitation differences (shaded; units: mm d^−1^) in response to changes in eccentricity, precession, and CO_2_ levels. Regions where differences are statistically significant at the 95% confidence level are indicated by dots. Black rectangles denote the location of the Ordos Basin. Middle panels (**e**–**h**): Summer (June-July-August) precipitation differences (shaded; units: mm d^−1^) and vertically integrated moisture transport differences (vectors, units: kg m^−1^ s^−1^) in response to changes in eccentricity, precession, and CO_2_ levels. Right panels (**i**–**l**): Seasonal cycles of insolation, temperature, precipitation, and net precipitation for four simulation experiments (*E*_*0*_, *XE*_*0*_, *P*_*max*_, and *P*_*min*_) in the Ordos Basin. Note that the seasonal insolation in *E*_*0*_ is identical to that in *XE*_*0*_.

The Ordos Basin was located in the monsoon region in our simulations (Fig. 1). Multiple lines of geological evidence, including paleobotanical fossils, paleosols, and flooding deposits, suggest a humid climatic condition in the Ordos Basin during that time^26,63–68^. Our simulations consistently indicate positive annual net precipitation (precipitation minus evaporation) for the Ordos Basin, regardless of *p*CO₂ and orbital configurations, also indicating a humid climatic condition (Fig. 1b–e). Due to the monsoon characteristics, summer precipitation plays a dominant role in determining the overall annual net precipitation (Fig. 4i–l and Supplementary Fig. S5). This feature is also reflected in the transient climate simulations conducted using the comprehensive Earth system model CLIMBER-X^62^ (Supplementary Fig. S6).

To further investigate the hydrologic response to orbital forcing, we analyzed the effects of end-members of different orbital configurations on precipitation patterns in the Ordos Basin (see “Methods”). When the summer insolation increases due to eccentricity-precession forcing, more moisture is transported to the Tethys region in summer, resulting in an increase in annual/summer precipitation there (Fig. 4a–c,e–g). In addition to the solar insolation changes, global warming due to elevated CO₂ levels also leads to enhanced moisture transport from the Tethys Ocean to the northeastern Tethys region, thereby increasing monsoon rainfall there (Fig. 4d,h). These simulated variations of the net/summer precipitation in response to external forcing (orbital and CO₂ variations) in the Ordos Basin are consistent with earlier transient climate simulations using the CLIMBER-X^62^ (Supplementary Fig. S6).

## Discussion

### Monsoon evolution in the northeast Tethys during the middle Triassic

In line with our observations of rising lake levels and increasing humidity (Fig. 3b–c), a recent study has identified two distinct periods of elevated lake levels during the middle Anisian and early Ladinian stages across much of the Ordos Basin^69^. Concurrently, the adjacent marine environment exhibited comparable significant changes. Specifically, climate proxy records from South China (GR series at Guandao section^70^, MS and Fe/Al series at Yongyue, Pohong, and Yongning Sections^54,71^) suggest heightened precipitation, greater nutrient influx, and boosted productivity in the marine environment during the middle Anisian and early Ladinian. During the Middle Triassic, a transcontinental drainage system flowed from northern North China into the Tethys Ocean across a gradually descending topography^72^ (Supplementary Fig. S1). It is therefore plausible that the concurrent changes observed in both terrestrial and marine environments can be attributed to the intensified hydroclimatic conditions at that time.

These hydrologic changes may induce ecological responses in both terrestrial and marine environments within the Tethys region. The Middle Triassic Lagerstätten, primarily discovered in the Tethys realm, include the Anisian marine Luoping and Panxian biotas, as well as the Ladinian marine Xingyi and lacustrine Tongchuan biotas^39,51–54,73–76^ (Fig. 3a). These exceptionally preserved fossil assemblages signify the complete recovery of marine and terrestrial ecosystems following the PTME. Paleoenvironmental and taphonomic evidence linked to these biotas further suggests common features like elevated lake/sea levels, increased primary productivity, and anoxic conditions during the middle Anisian and early Ladinian^39,71,77,78^. It is conceivable that these hydrologic changes could have regulated ecological niches fostering species diversification, enhanced nutrient fluxes stimulating primary productivity bursts, and created anoxic environments conducive to exceptional fossil preservation. Moreover, although biodiversity data during the Middle Triassic remain limited, existing datasets reveal an increase in marine benthic communities during the middle Anisian^79^ and a rise in conodont diversity during the early Ladinian^80^, suggesting a correlation between hydroclimate and biodiversity worthy of further investigation.

Climate modeling provides mechanistic insights into the observed hydroclimatic changes (Figs. 1,4). Based on the monsoon precipitation index^81^ (see “Method”), the CESM and CLIMBER-X^62^ simulations (Supplementary Fig. S6) collectively highlight the leading role of summer monsoon rainfall in shaping the annual mean net precipitation in the Middle Triassic Ordos Basin. Notably, the paleotopography of the Tethys realm likely influenced regional monsoon evolution and orbital-scale precipitation changes by modulating atmospheric circulation^82^. While uncertainties in the Tethyan microcontinent reconstruction^83,84^, particular regarding spatial resolution and age constraints, the consistent modeling results offer a most plausible hydroclimatic framework for the geological observations discussed above. Specifically, the modeling results support the notion of enhanced monsoon hydrology during the lake expansions of the Ordos Basin in the middle Anisian and early Ladinian.

### Long-term climate cycles

To investigate the potential orbital drivers behind these monsoon hydrologic changes, particularly orbital grand cycles, we compared astronomical solutions (Laskar^31,32^ and Zeebe^85^ solutions) with multiple established and independent geological records. Notably, amplitude modulation analysis of our records is not employed to detect grand cycles because the divergence between ${r}_{spectral}^{2}$ and ${r}_{envelope}^{2}$ in TimeOpt undermines our confidence that astronomical amplitude modulation is well preserved in our records (Supplementary Fig. S4). Alternatively, comparing well-established geological records with astronomical solutions provides a feasible method to determine the orbital origin of paleoclimate changes^15,20,85^. The δ^18^O^56^, δ^13^C^57–59^, and BSi^19,20^ data series compared here represent the most continuous and mutually independent geological compilations available for the Triassic. The relatively diminished influence of tectonic activities during the Middle Triassic increases the likelihood of well-preserved orbitally-forced climate cycles in the geological records^20,56^ (Supplementary Figs. S7 and S8). A conspicuous ~ 3.3 Myr cycle during the Middle Triassic is evident in the La2010d eccentricity solution^32^, the δ^18^O data series^56^, and BSi records^19,20^ (Fig. 3i and Supplementary Fig. S7). However, this cycle is not discernible in the δ^13^C data, possibly due to insufficient sampling resolution or age uncertainties^20^ (Fig. 3i). Moreover, we find a strong coherence between the eccentricity power of the La2010d solution and the δ^18^O time series, revealing a common cycle with a period of ~ 3.3 Myr during the Middle Triassic (Supplementary Fig. S8). La2010d is not only a rigorously constrained model that incorporates major asteroids^32^ but also shows an agreement with geological records during the Triassic^20,86^, thus supporting the orbital origin of the ~ 3.3 Myr cycle during that time.

The ~ 3.3 Myr eccentricity cycles are also well-preserved in the monsoon archives from Pangea and Panthalassa, thus revealing a fundamentally cyclic pattern for the megamonsoon system. The astronomical origin of the ~ 3.3 Myr cycle can be traced to the work of Olsen^87^, who observed ~ 3.5 Myr and ~ 1.75 Myr cycles from the Late Triassic fluviolacustrine sequence in the Newark Basin and proposed that they are related to today’s ~ 4.6 Myr and ~ 2.4 Myr eccentricity cycles, stemming from the Earth-Mars secular resonance (*θ* = 2(*g*₄–*g*₃)–(*s*₄–*s*₃)) and *g*₄–*g*₃, respectively. Here, *g*₃ and *g*₄ represent the precession of the perihelion of the Earth and Mars, respectively, whereas s₃ and s₄ refer to the precession of their corresponding nodes. Despite receiving less attention compared to the *g*₄–*g*₃ cycle (e.g., ~ 2.4 Myr cycle today), the *θ* cycle (e.g., ~ 4.6 Myr cycle today) may have acted as a regulator for sea-level changes^88,89^ and CO₂ fluctuations^90^. Notably, a significant ~ 3.3 Myr cycle was identified in Middle Triassic pelagic sequences in Japan and was interpreted as reflecting the monsoon dynamics during that epoch^17,19,20^. This gives us great confidence that the ~ 3.3 Myr cycle we have identified in the geological records and astronomical solutions represents a grand eccentricity cycle, at least for the Middle Triassic (Fig. 3i). Although much more can be recovered regarding grand cycles, the well-expressed ~ 3.3 Myr cycles during the Middle Triassic provide a unique window to detect both the behavior of these grand cycles and their forcing mechanisms.

### Driving mechanisms on the eccentricity-scale megamonsoon variability

The imprint of the 405 kyr and ~ 3.3 Myr cycles in our hydroclimatic reconstructions provides valuable insights into the monsoon dynamics in the Tethys region. However, variations in the megamonsoon system on eccentricity timescales are inadequately explained by orbital forcing alone, primarily due to the minor influence of eccentricity on insolation compared to precession. Thus, it is essential to consider the feedback mechanisms. Our combined reconstruction and simulation results support the following direct and indirect mechanisms: (1) the nonlinear response of the climate system to insolation changes whereby changes in solar radiation can induce disproportionate responses in precipitation patterns; (2) as shown in Fig. 3, the consistent prevalence of ~ 3.3 Myr cycles in monsoon hydrology, temperature, and sea levels suggests complex interplays among these factors under the influence of orbital forcing.

The nonlinearity in the climate’s response to orbitally-forced insolation and sedimentary systems can impose a smoothing effect (or clipping effect), attenuating high-frequency signals (e.g., precession, the largest contributor to mid- and low-latitude summer insolation variability and monsoon hydrology^30,33^) and amplifying the low-frequency amplitude cycles (e.g., eccentricity)^7,91–93^. Crowley, et al.^7^ proposed that such smoothing effects may arise from the climate’s response to equatorial insolation changes during the (functionally) ice-free Triassic. Our previous study suggests that the similarity between millennial-to-orbital scale climate variability recorded in the sediments of the Yanchang Formation and low-latitude insolation changes suggests that regional climate may be affected by low-latitude processes and their associated smoothing effects^26^. Our simulations indicate the potential for a smoothing effect to emerge in the climate’s response to orbitally-forced insolation changes on a large scale, not confined to the equatorial regions. Specifically, our simulations suggest that the precipitation response in the Ordos Basin does not increase linearly with increasing summer insolation across the orbital configurations (*P*_*max*_, *E*_*0*_, and *P*_*min*_). Both the annual net precipitation and the summer precipitation under high eccentricity (*P*_*min*_) display variability comparable to those under low eccentricity (*E*_*0*_) (Fig. 4b,f). This suggests the existence of a threshold beyond which the precipitation response to increasing insolation becomes constrained. Consequently, the transfer of high-frequency precession signals into lower-frequency eccentricity cycles could occur through this nonlinear filtering process, thereby amplifying eccentricity-induced modulation of monsoon variability^91^. We propose that the occurrence of the clipping effect may have influenced the documentation of astronomical signals, particularly their modulation components, thereby diminishing the effectiveness of modulation analysis methods such as ${r}_{envelope}^{2}$ in TimeOpt. However, this nonlinear response is unlikely to be unique, and alternative forcing mechanisms need considering to fully understand the eccentricity cycle in the monsoon variability, particularly concerning the ~ 3.3 Myr cycle.

The correlation between warming phases inferred by decreased δ^18^O values and intensified monsoon conditions in the Ordos Basin supports the thermally-driven Tethys monsoon, on eccentricity timescales, which is distinct from precession-driven monsoon systems (Fig. 3e and Supplementary Fig. S8). The temperature oscillations observed at eccentricity timescales cannot be attributed to the insolation variations (Supplementary Fig. S8), as eccentricity exerts only a minor influence on insolation. Instead, changes in greenhouse gas concentrations, particularly CO₂ levels, seem to be the predominant factor driving the temperature variations at these timescales. Notably, negative carbon isotope excursions, as a possible consequence of elevated CO₂ concentrations, coincide with the Anisian and Ladinian warming episodes^59,94^, providing evidence for the influence of the carbon cycle on the monsoon hydrologic system of the Ordos Basin. Our simulations demonstrate that the warming caused by elevated CO₂ levels enhances monsoon hydrology in the Ordos Basin (Fig. 4d,h,j–l), aligning with established studies showing the influence of higher temperatures and/or CO₂ levels on the enhanced hydrologic cycle over the Tethys^34,62,94,95^. Unfortunately, continuous proxy-based CO₂ reconstructions are unavailable for the Middle Triassic; nonetheless, the observed out-of-phase relationship between chemical weathering intensity (inferred from BSi flux^19,20^ and Sr isotope^60,61^ datasets) and δ^18^O-inferred temperature reconstructions (Fig. 3e–h) implies that silicate weathering might have regulated atmospheric CO₂ levels during this period. This weathering-induced CO₂ drawdown pattern on eccentricity timescales has been observed in the Late Triassic, where enhanced weatherability paced by long-term eccentricity led to reduced *p*CO₂ levels and consequent climate shifts^19,21,96^. Additional exploration of the specific mechanisms underlying the orbitally-forced temperature changes is warranted, with silicate weathering emerging as a feasible explanation.

Sea level changes during the Middle Triassic seem to have influenced monsoon dynamics (Fig. 3b–d). The lake level changes in the Ordos Basin closely correspond to the eustatic cycles identified through sequence stratigraphy from Tethys^55,97^ (Fig. 3d). This supports previous research identifying the synchronous maximum expansion of the Ordos Basin’s lacustrine system with the early Ladinian maximum flooding surface in eustatic sea level^66^, and reveals another lake expansion of the Ordos Basin corresponding to the sea level rise during the Anisian (Fig. 3). This implies that rising sea levels may have contributed to the monsoon hydrology in the Ordos Basin. In line with the established connection between Cenozoic monsoon variability and sea level changes^98,99^, it is reasonable to infer that rising sea levels could have shortened the pathways of monsoon moisture transport, thereby enhancing regional precipitation. Conversely, lower sea levels could have lengthened these pathways, reducing moisture delivery. The exact mechanism underlying the observed sea level changes on eccentricity timescales during the Triassic remains elusive (Fig. 3d). Analogously, ~ 2.4 Myr and ~ 4.7 Myr eccentricity cycles^100^ are evident in sea level changes during the Cretaceous, a comparable greenhouse period. These fluctuations might be attributed to thermo-eustasy, glacio-eustasy, aquifer-eustasy, or a combination of these processes^101^. Detailed analyses of these fundamental processes underlying the eccentricity-driven rhythm of sea level changes during the Middle Triassic are beyond the scope of the present study; however, our observation highlights a previously unobserved co-variation between lake and sea levels on eccentricity timescales. This finding contrasts with the inverse relationship between lake and sea levels observed during the Early Triassic within ~ 1.2 Myr obliquity cycles^102^. This disparity suggests the intricate regional and temporal responses to disparate orbital influences, pointing to the necessity for further studies to integrate geological records and modeling simulations, aiming at a comprehensive understanding of the spatiotemporal picture of hydrologic changes over orbital timescales.

## Conclusion

Our temporally calibrated, orbitally resolved reconstruction of the lake hydroclimate variations in the Ordos Basin, spanning from ~ 246 to 239 Ma, in tandem with our climate simulations, provides key insights into the behavior of the monsoon hydrologic cycle in the Tethys region during the Middle Triassic. The 405 kyr and ~ 3.3 Myr eccentricity signatures are evident in the reconstructed hydrologic cycles in the Ordos Basin, with the latter representing a distinct expression of the orbital grand cycles during the Middle Triassic, as supported by both geological records and the La2010d astronomical solution. Climate simulations support the leading role of the summer monsoon in the hydroclimate of the Ordos Basin and demonstrate the relative impact of orbit and CO₂ on hydroclimate. Our data-model evidence of monsoon dynamics in the Tethys region supports the idea that the fingerprint of the eccentricity, specifically for the ~ 3.3 Myr cycle, can be attributed to the interplay between external and internal forcings, thus enhancing our understanding of long-term climate changes during the Triassic.

## Methods

### Time series analysis

Magnetic susceptibility (MS) data were collected at 2-cm intervals from the core surface using an MS-30 magnetic susceptibility meter (ZH-Instruments, Czech Republic). The MS series have previously been shown to effectively capture the orbitally-forced redox and detrital input cycles within the interval from 258 to 198 m of the Yaoye-1 core^26,44^. X-ray fluorescence (XRF) measurements were performed with a handheld XRF instrument (NITON XL 2800, Thermo Scientific) at 20-cm intervals. Because Fe is commonly concentrated under humid conditions and Ba is enriched under arid conditions^103^, Fe/Ba variability can reflect changes in paleoclimatic conditions.

The proxy data were detrended by subtracting a 40-m ‘loess’ trend to eliminate long-term trends that might obscure Milankovitch-scale sedimentary cyclicity. The detrended data were then analyzed using the multi-taper method (MTM) spectral estimator, with confidence tests conducted using the smoothed window averaging (SWA) method^104^ to find spectral backgrounds plus false discovery rates (FDR) to reduce the likelihood of false detections of regular cycles. This was followed by Gaussian bandpass filtering to isolate key astronomical signals, such as the 405 kyr eccentricity cycle. The center frequency and the passband of the ~ 16 m cycles, identified as the sedimentary response to the 405 kyr eccentricity cycle, were set to 0.0625 ± 0.03 cycles m^-1^. The proxy data series were transformed from stratigraphic to temporal domain by tuning the 405 kyr cycle. Wavelet power spectral evolution was used to track the cyclical signals and potential orbital cycles in both stratigraphic and temporal domains. One-dimensional continuous wavelet transformation and Morlet wavelet (*k*_0_ = 6) were employed in the wavelet analysis. All these methods of time-series analysis were performed using Acycle^105^.

Statistical tuning was applied to evaluate optimal sedimentation rates using correlation coefficient (COCO)^106^ and TimeOpt^107^ approaches. Six astronomical target periodicities (i.e., 405, 125, 95, 33.0, 20.8, 19.8, and 17.2 kyr) are based on the power spectrum of astronomical target series (La2004 solution from 245 to 240 Ma^31^). Tested sedimentation rates range from 1 to 8 cm kyr^−1^ with a step of 0.1 cm kyr^−1^ for COCO and 0.07 cm kyr^−1^ for TimeOpt (i.e., 100 sedimentation rates between 1 to 8 cm kyr^−1^ were evaluated in the optimization grid of TimeOpt). The null hypothesis that no astronomical forcing drove oscillations of the proxy series was determined through Monte Carlo simulations with 2000 iterations. By incorporating a moving window approach into the COCO and TimeOpt methods, these techniques evolved into the eCOCO and eTimeOpt methods, which were subsequently utilized to investigate variable sedimentation rates within the stratigraphic domain. The sliding window size for both eCOCO and eTimeOpt was set to 100 m, with a step size of 1 m. The (e)COCO and (e)TimeOpt analyses were accomplished by the Acycle^105^ and *astrochron* package in R^108^, respectively.

### Model and experimental setup

This study utilizes the Community Earth System Model version 1.2.2 (CESM1.2.2) to model the Triassic climate and explore the effects of orbital forcing and CO₂-induced warming on hydroclimate variability. The CESM1.2.2 developed by National Center of Atmospheric Research (NCAR) is a fully coupled general circulation model that includes atmosphere, ocean, sea-ice, land, and runoff components, along with a coupler^109^, offering state-of-the-art climate simulations that enable the analysis of past, present, and future climate states^82,110^. The atmospheric component, Community Atmosphere Model version 4 (CAM4)^111^, and the land component, Community Land Model version 4.0 (CLM4.0)^112^, both have a horizontal resolution of 3.75° × 3.75°, with 26 and 10 vertical levels, respectively. The oceanic component, Parallel Ocean Model (POP2)^113^, is configured at a nominal 3-degree horizontal resolution with 60 vertical levels. The sea-ice component, Community Sea Ice Model (CICE4)^114^, shares the same horizontal grid as POP2. The runoff component is executed at a default resolution of 0.5° × 0.5°.

We designed four experimental simulations to assess the sensitivity of the Ordos Basin hydroclimate to different orbital configurations and atmospheric CO₂ levels. These experiments were termed as “*E*_*0*_”, “*XE*_*0*_”, “*P*_*max*_”, and “*P*_*min*_”, respectively. The atmospheric CO₂ concentrations (*p*CO₂) were set to 1,000 ppmv for the “*E*_*0*_”, “*P*_*max*_”, and “*P*_*min*_” experiments, based on proxy data and model estimates for the Middle Triassic^21,94,96,115^ (also see www.planetaryhabitability.org). The “*XE*_*0*_” experiment, however, used a higher *p*CO₂ of 2,500 ppmv to simulate elevated greenhouse conditions. Both the “*E*_*0*_” and “*XE*_*0*_” experiments were conducted with low eccentricity (0). In the “*P*_*min*_” experiment, the eccentricity is fixed at 0.07 with the perihelion occurring in the boreal summer. The “*P*_*max*_” experiment has the same eccentricity with that in “*P*_*min*_” experiment, but the perihelion occurred in the austral summer. Obliquity is fixed at 23.5° across all experiments. The paleogeography was set to ~ 250 Ma using reconstructions by Scotese and Wright^42^ (Fig. 1a), and paleovegetation was adapted from Rees, et al.^116^. The solar constant was adjusted to 1338 W m⁻^1^, approximately 2.1% lower than present-day value^35^. CH₄ and N₂O concentrations were set to 760 ppbv and 270 ppbv, respectively, with no CFCs included. Other atmospheric constituents, such as O₃ and aerosols, were set to preindustrial levels, which are the default values of the CESM1.2.2. The “*E*_*0*_” and “*XE*_*0*_” experiments were run for 2,700 and 2,000 model-years, respectively, with initial conditions from Li, et al.^110^. The “*P*_*min*_” and “*P*_*max*_” experiments have the same *p*CO₂ as in “*E*_*0*_” experiment, and continued for another 800 model years. The atmosphere and surface ocean in all modeling experiment have already achieved equilibrium, and the final 100-model-year data of each simulation were analyzed and presented herein.

The monsoon regions were defined using the monsoon precipitation index^81^, where regions experiencing summer-minus-winter precipitation exceeding 2 mm d⁻^1^ with summer precipitation greater than 55% of the annual total were classified as monsoon regions. Local summer was defined as May through September for the Northern Hemisphere and November through March for the Southern Hemisphere.

## Supplementary Information

Supplementary Information.

## Data Availability

The proxy series of lithological index, magnetic susceptibility, and X-ray fluorescence measurements generated in this study are provided in the Supplementary Information.
