# Supplementary material for: Orbital eccentricity and internal feedbacks drove the Triassic megamonsoon variability
Source: Sci Rep. 2025 Jul 7;15:24190. doi: 10.1038/s41598-025-09295-2 (PMC12234772; doi:10.1038/s41598-025-09295-2)
Supplement: Supplementary file 1 — Supplementary Information. [file 41598_2025_9295_MOESM1_ESM.docx]

**Supporting Information for**

# Orbital eccentricity and internal feedbacks drove the Triassic megamonsoon variability

Runjian Chu^1,2^, Huaichun Wu^1,2,3*^, Jian Zhang^4*^, Qiang Fang^1,2,3^, Christian Zeeden^5^, Peng Chen^1,2^, Rukai Zhu^6^, Jingwei Cui^6^, Shihong Zhang^1,2^, Tianshui Yang^1,2^, Chengshan Wang^1,2^

^1^ State Key Laboratory of Geomicrobiology and Environmental Changes, China University of Geosciences, Beijing 100083, China.

^2^ Frontiers Science Center for Deep-time Digital Earth, China University of Geosciences (Beijing), Beijing 100083, China.

^3^ Key Laboratory of Polar Geology and Marine Mineral Resources, School of Ocean Science, China University of Geosciences (Beijing), Beijing 100083, China.

^4^ State Key Laboratory of Tibetan Plateau Earth System, Environment and Resources (TPESER), Institute of Tibetan Plateau Research, Chinese Academy of Sciences, Beijing 100101, China.

^5^ LIAG-Leibniz Institute for Applied Geophysics, Stilleweg 2, 30655 Hannover, Germany.

^6^ Research Institute of Petroleum Exploration & Development, PetroChina, Beijing 100083, China.

**The File includes:**

**1. Figures S1 to S8**

**2. Tables S1 to S3**

**3. SI References**


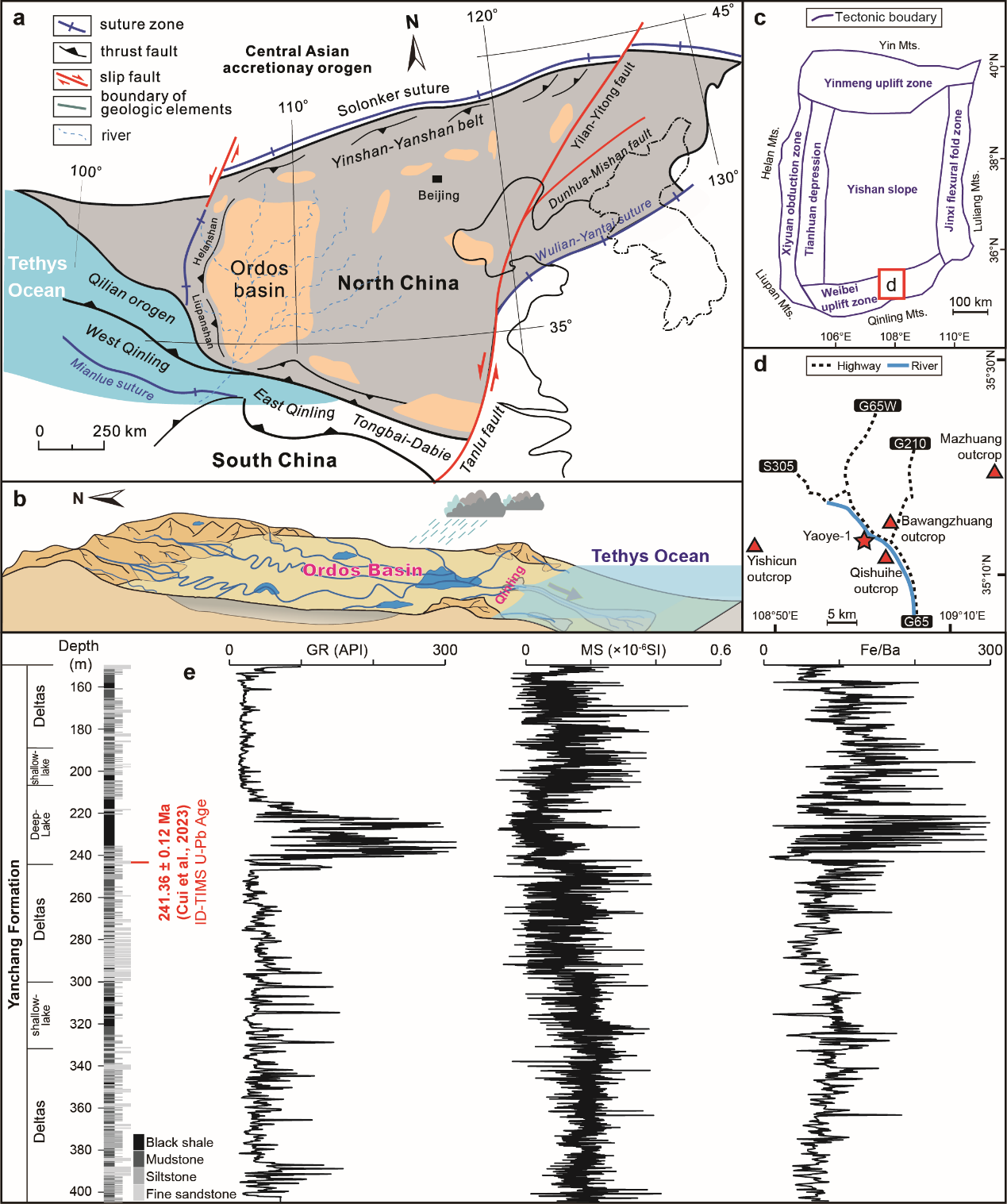
**Supplementary Fig. S1 Paleogeographic framework of the Mesozoic Ordos Basin,** **North China Craton, and location, lithology, and paleoclimate proxies of the Yaoye-1 core. a** North China paleogeography during the Middle Triassic, adapted from Meng, et al. ^1^. **b** Schematic model illustrating the stream drainage systems in the Ordos Basin during the Middle Triassic, adapted from Peng, et al. ^2^. **c** Tectonic unit of the Ordos Basin and location of the study area. **d** Locations of the Yaoye-1 core and four outcrops mentioned in the text^3,4^. **e** Stratigraphic column for the Yanchang Formation of the Yaoye-1 core in the Ordos Basin, illustrating the lithostratigraphy, depositional environments, U-Pb geochronology, and proxy data series. The U-Pb age of 241.36 ± 0.12 Ma was reported by Cui, et al. ^5^.


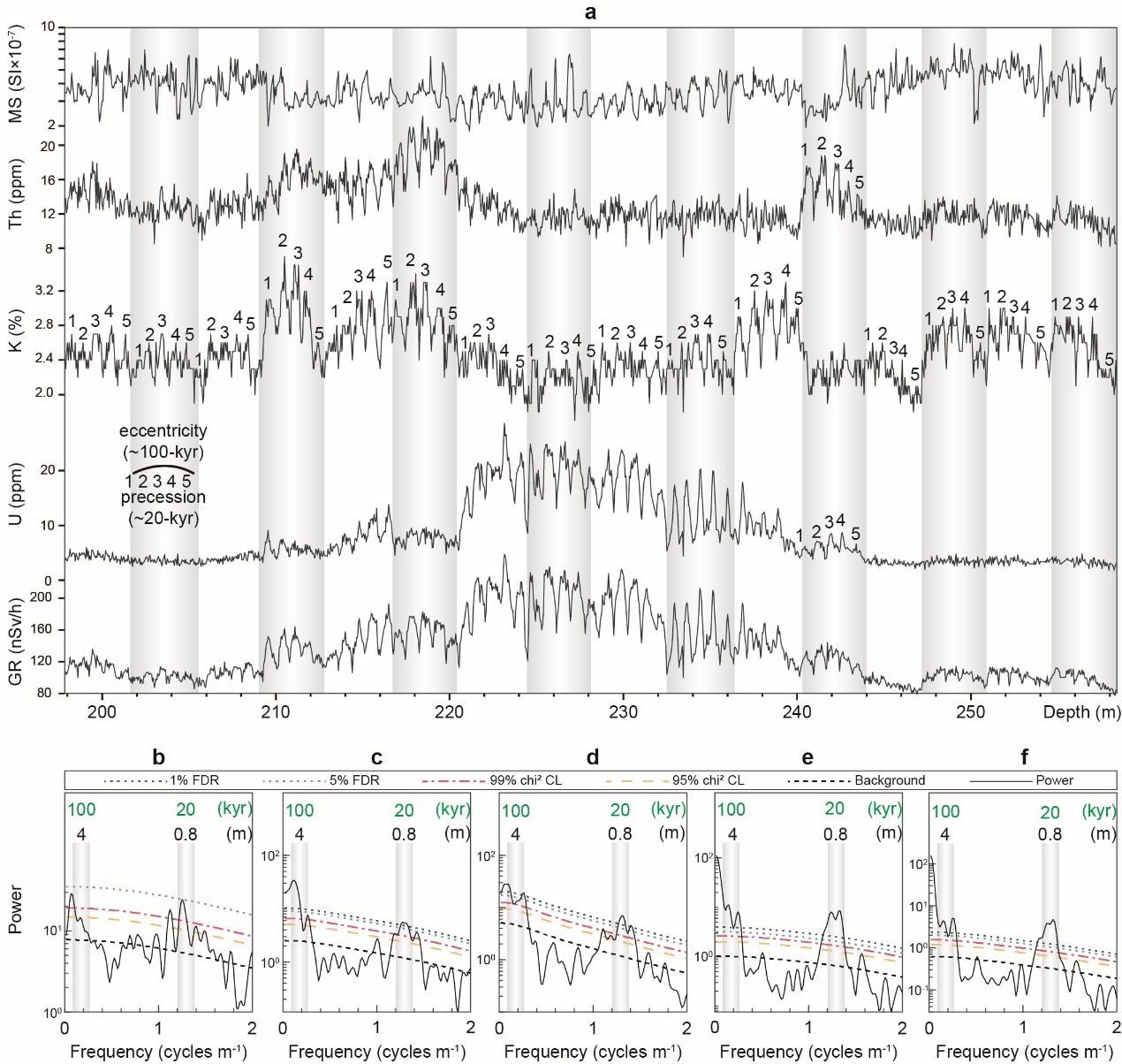


**Supplementary Fig. S2 Evident ~100 kyr eccentricity and ~20 kyr precession cycles illustrated by multi-proxy data series across the interval of 258.4–197.85 m at Yaoye-1 (see Chu, et al. ^6^ for details). a** Magnetic susceptibility (MS), thorium (Th), potassium (K), uranium (U), and gamma-ray (GR) data series showing the ~100 kyr eccentricity and ~20 kyr precession cycles. A grouping of five precession cycles (labeled as cycles 1 through 5) constitutes one eccentricity cycle in the stratigraphic domain. **b–f** Power spectra of the proxy data series (MS in panel **b**, Th in panel **c**, K in panel **d**, U in panel **e**, and GR in panel **f**) after removing the 20 m ‘loess’ trend. The confidence levels (CLs) achieved by the smoothed window average (SWA) method include the 95% and 99% Chi-squared CLs as well as the 5% and 1% false discovery rate (FDR) CLs. Significant peaks (e: short eccentricity; O: obliquity; P: precession) are indicated in both the depth (in meters) and time (in kiloyears) domains, using a sedimentation rate of 4 cm kyr^-1^.


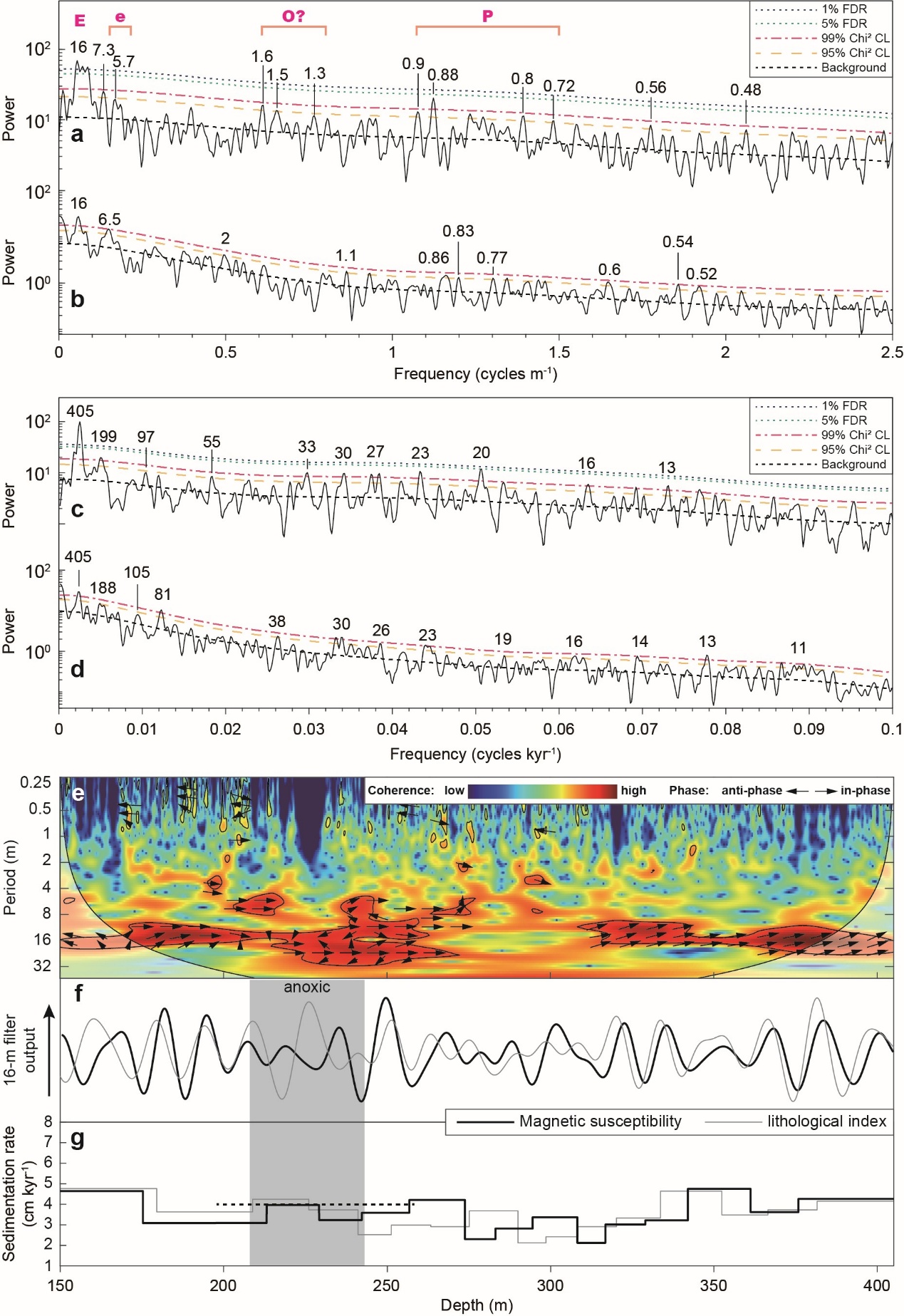


**Supplementary Fig. S3 Cyclostratigraphic analysis of the Magnetic susceptibility (MS) and lithological series of the Yaoye-1 core. a** Power spectrum of the MS series (after subtracting a 40-m ‘loess’ trend) in the stratigraphic domain. The spectrum illustrates the power associated with long eccentricity (E), short eccentricity (e), obliquity (O), and precession (P) cycles. Significant peaks are labeled in the meter. **b** Power spectrum of the lithological index series in the stratigraphic domain. Significant peaks are labeled in the meter. **c** Power spectrum of the tuned MS series (after subtracting a 1000-kyr ‘loess’ trend) in the time domain. Significant peaks are labeled in the kiloyear. **d** Power spectrum of the tuned lithological index series in the time domain. Significant peaks are labeled in the kiloyear. **e** Cross wavelet spectra between lithological and MS data series in the stratigraphic domain, showing a coherent cyclicity at ~16 m. **f** The 16 m filtered cycles derived from both the MS and lithological series (passbands: 0.0625 ± 0.03 cycles m^-1^). **g** The sedimentation rate curves derived from 405 kyr tuning for both the MS and lithological series. The dotted line indicates a stable sedimentation rate of 4 cm kyr^-1^ based on the 0.8 m cycle corresponding to the 20 kyr precession cycles across the interval of 258.4–197.85 m (Fig. S2; see Chu, et al. ^6^ for details). Note that the transition from in-phase to anti-phase of the long eccentricity cycle (~16 m cycles in the stratigraphic domain) between the MS and lithological series corresponds to the depositional environment transition from oxic to anoxic. This can be attributed to the preservation and dissolution cycles of detrital magnetic minerals in oxic and anoxic environments, respectively, as recorded in the MS data.


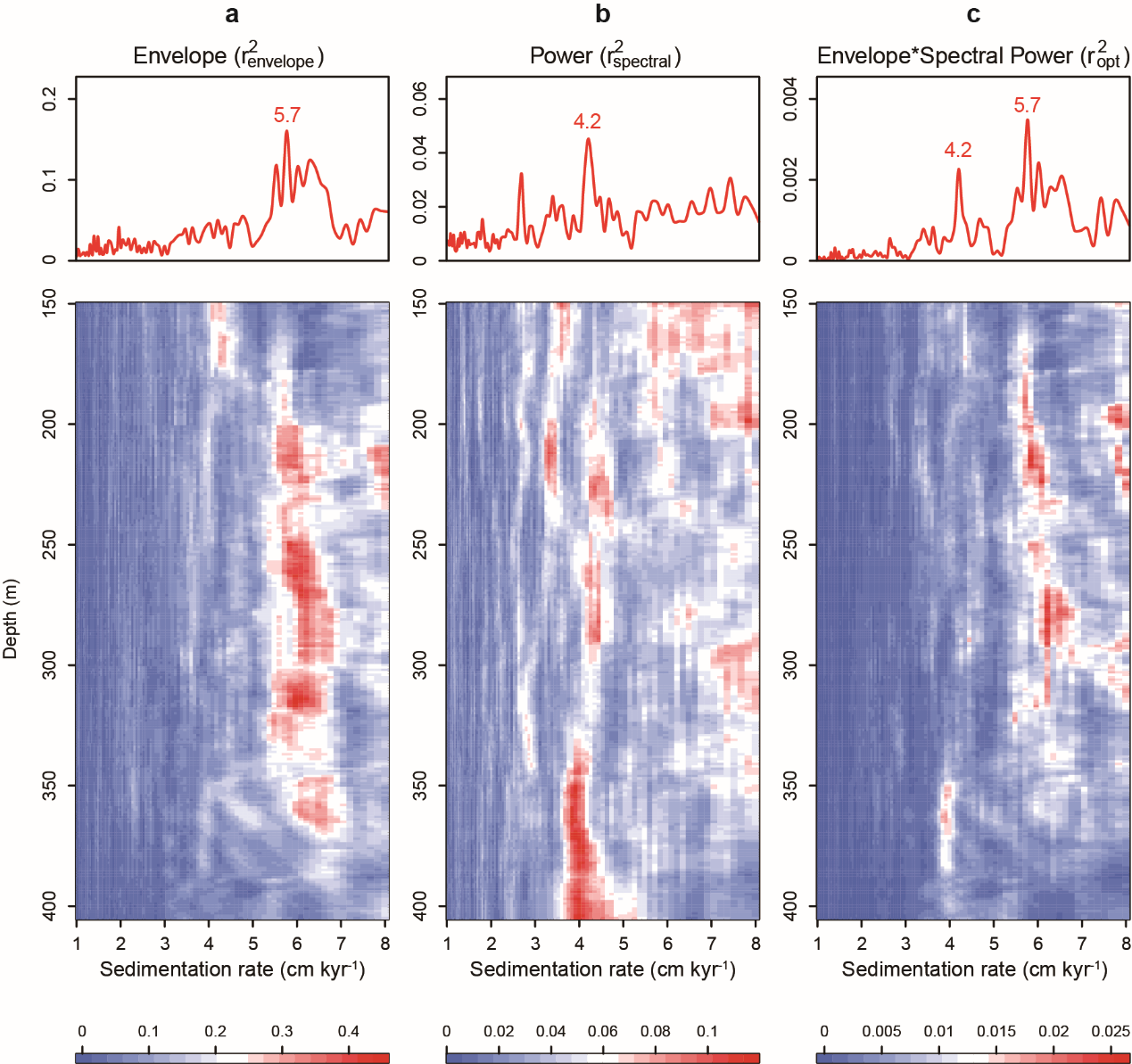


**Supplementary Fig. S4 TimeOpt and eTimeOpt of the Magnetic susceptibility (MS) series of the Yaoye-1 core. a** Squared Pearson correlation coefficient for the amplitude envelope fit ($r_{envelope}^{2}$) at each evaluated sedimentation rate. **b** Squared Pearson correlation coefficient for the spectral power fit ($r_{spectral}^{2}$) at each evaluated sedimentation rate. **c** Combined envelope and spectral power fit ($r_{opt}^{2}$) at each evaluated sedimentation rate.


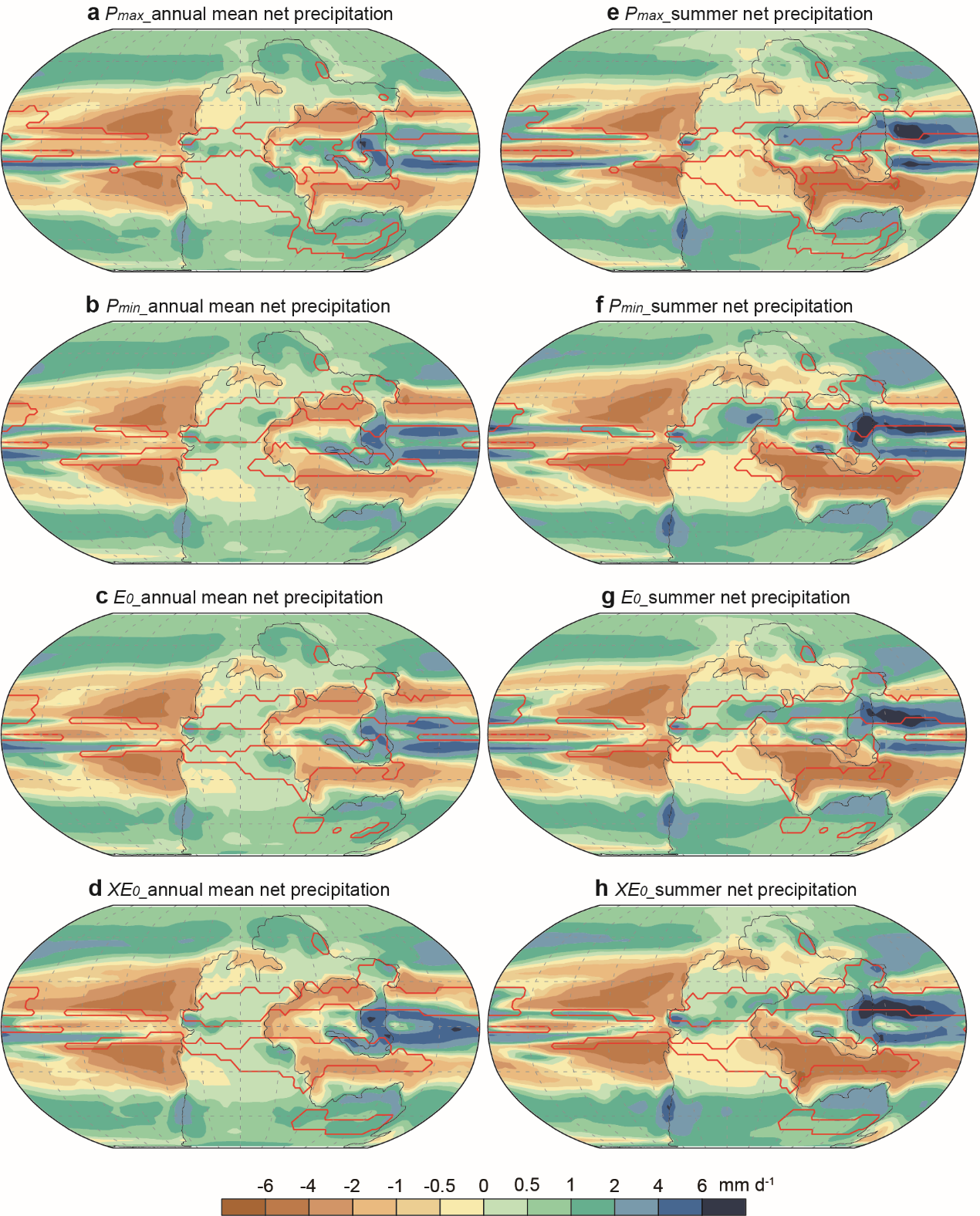


**Supplementary Fig. S5 Comparison between annual mean net precipitation and summer net precipitation.** Annual mean net precipitation (shaded; units: mm d^-1^) for the simulations of *P_max_* (**a**), *P_min_* (**b**), *E_0_* (**c**), and *XE_0_* (**d**). Summer net precipitation (shaded; units: mm d^-1^) for the simulations of *P_max_* (**e**), *P_min_* (**f**), *E_0_* (**g**), and *XE_0_* (**h**). The monsoon domains, outlined in red lines, are defined by the monsoon precipitation index^7^.


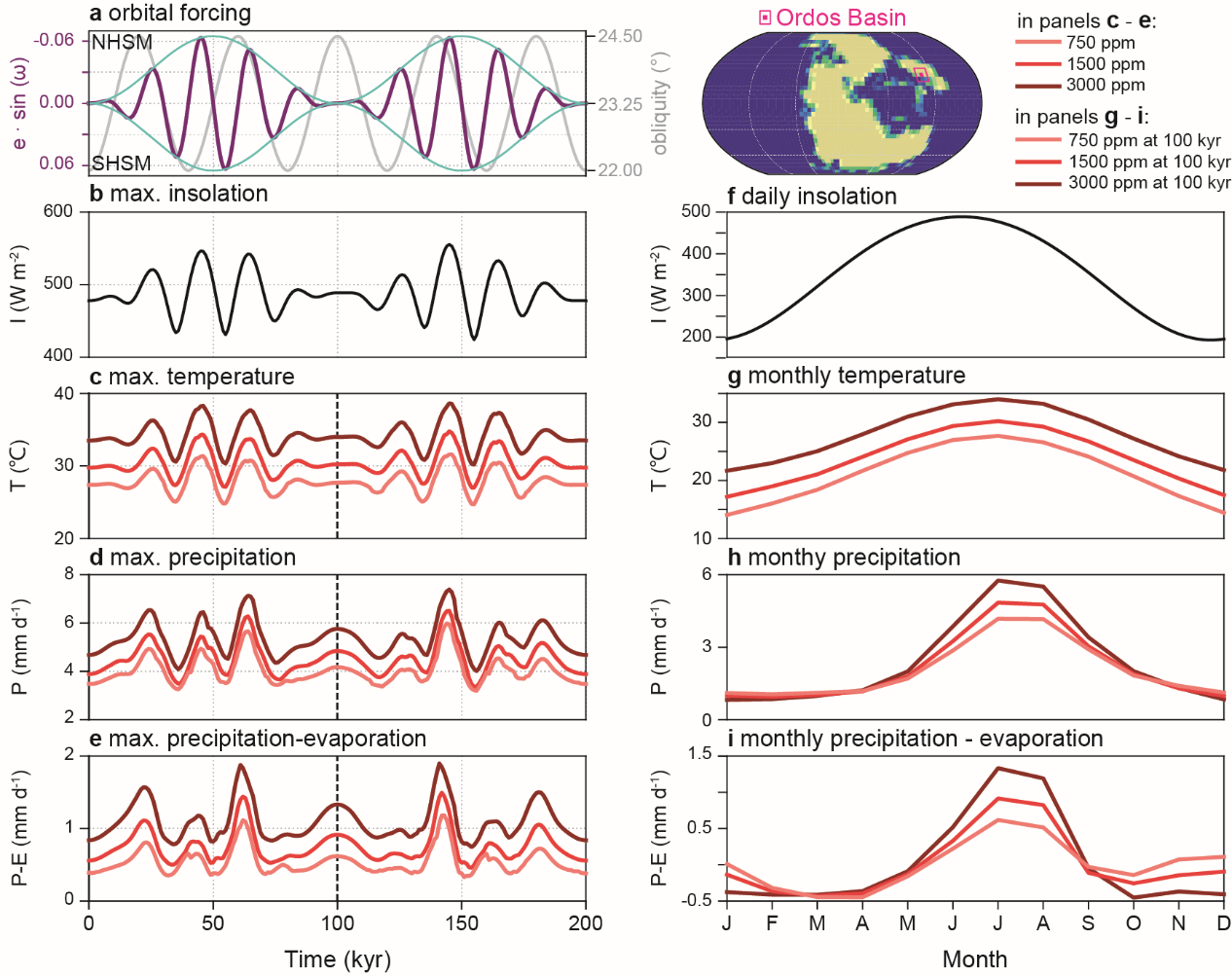
**Supplementary Fig. S6 Simulated effects of the imposed orbital forcing and CO₂ on the regional climate conditions in the Ordos Basin using CLIMBER-X^8^. a** Orbital forcing expressed by the precession index e · sin (ω) (e, perihelion angle; ω, eccentricity) and obliquity ε. The left axis is inverted so that maximum Northern Hemisphere summer insolation (NHSM) occurs at the top of the plot, while SHSM denotes maximum Southern Hemisphere summer insolation. **b–e** Maximum monthly values of regional climate quantities for three simulations at different *p*CO₂ levels of 750, 1,500, and 3,000 ppmv, using 230 Ma Marcilly paleogeography. Vertical lines indicate three points in time for which monthly data are shown on the right side. **f–i** Monthly values of the same quantities at minimum ω and ε (at 100 kyr; dashed lines).
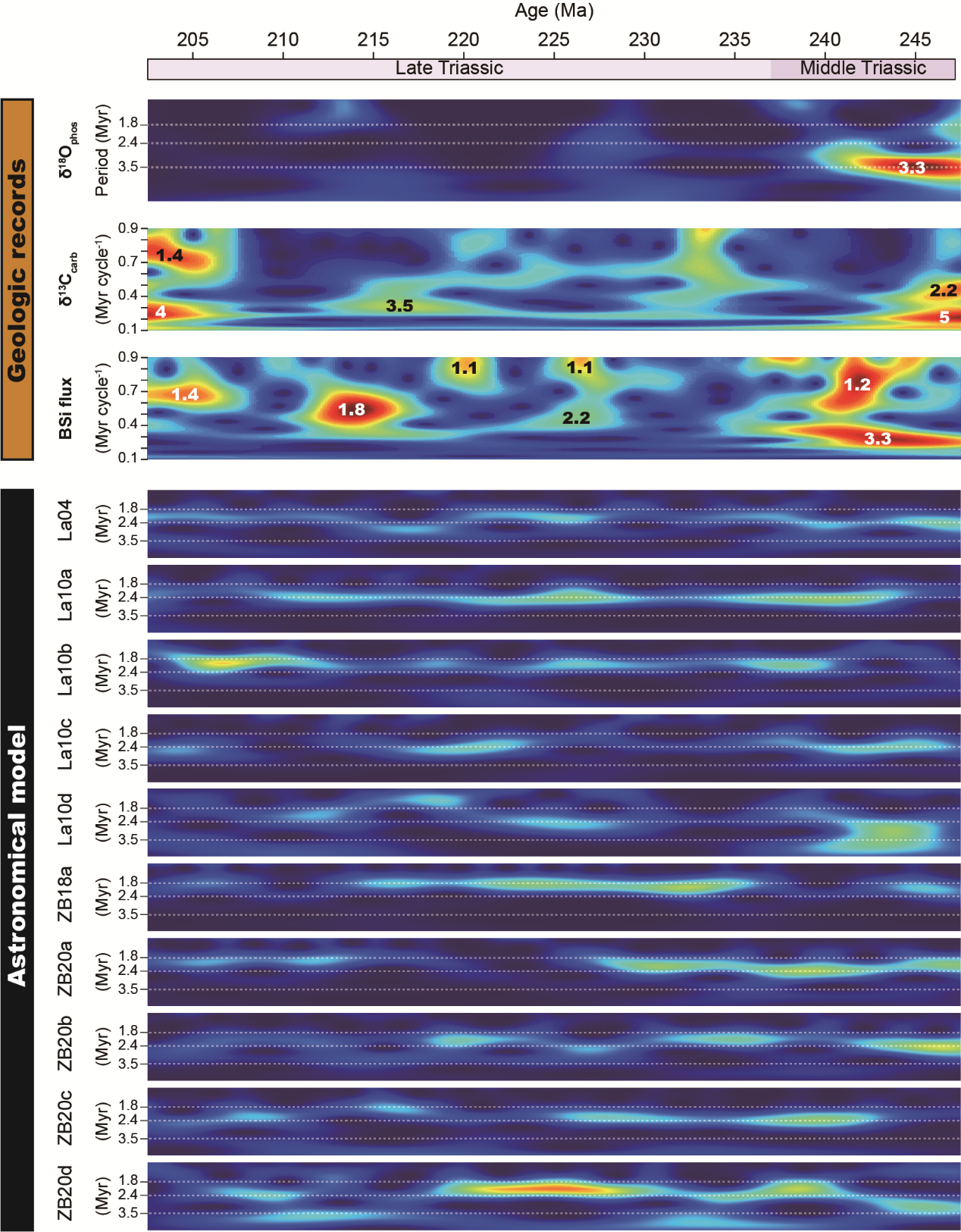


**Supplementary Fig. S7 Comparison of multi-Myr cycles in geologic records and astronomical solutions during the Middle to Late Triassic (see Ikeda and Tada ^9^ for details).** Wavelet analysis and evolutionary spectra of oxygen isotope (δ¹⁸O)^10^, carbonate carbon isotope (δ¹³C)^9^, biogenic silica (BSi) burial flux data^9^, and astronomical solutions (Laskar and Zeebe solutions)^11-13^.


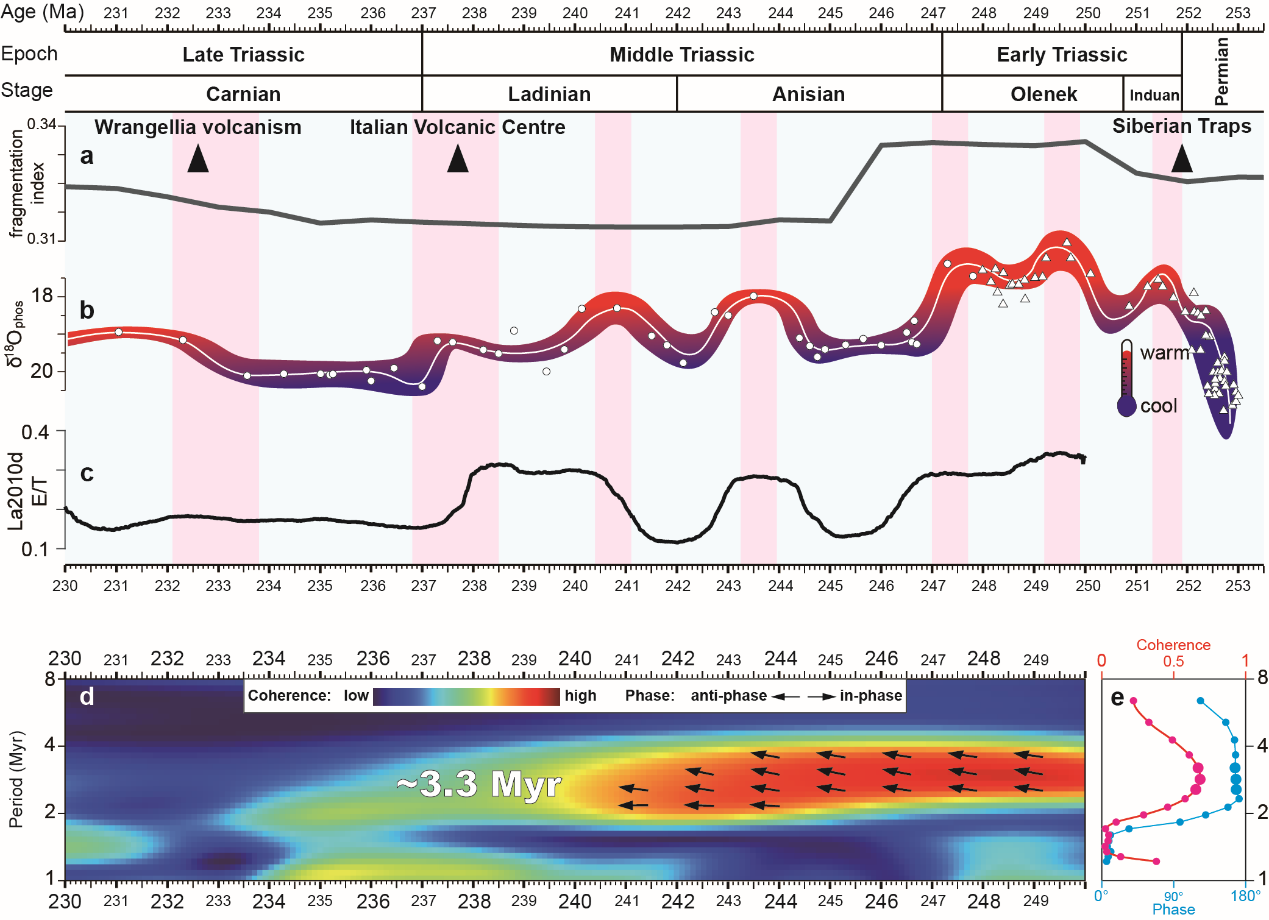


**Supplementary Fig. S8 Temperature oscillations indicated by the oxygen isotope compositions of conodont apatite (δ¹⁸O) during the Triassic, which were triggered by orbitally forced and/or volcanism-related mechanisms.** **a** An index of continental block fragmentation^14^ and major volcanic events^10^. **b** The δ¹⁸O composite curve during the Triassic^10^. **c** The ratio of 405 kyr eccentricity variance to total variance (E/T) of the La2010d eccentricity series^12^ with a sliding window of 1,500 kyr. **d** Cross wavelet spectra between δ¹⁸O (from panel **b**) and the E/T ratio (from panel **c**). **e** Coherence and cross-phase analyses between δ¹⁸O (from panel **b**) and the E/T ratio (from panel **c**).

**Supplementary Table S1. Lithological index**

| Depth  (m) | Lith  index | Depth  (m) | Lith  index | Depth  (m) | Lith  index | Depth  (m) | Lith  index | Depth  (m) | Lith  index | Depth  (m) | Lith  index |
| --- | --- | --- | --- | --- | --- | --- | --- | --- | --- | --- | --- |
| 150 | 1 | 200 | 4 | 250 | 4 | 300 | 3 | 350 | 2 | 400 | 4 |
| 150.1 | 1 | 200.1 | 4 | 250.1 | 4 | 300.1 | 3 | 350.1 | 2 | 400.1 | 3 |
| 150.2 | 1 | 200.2 | 4 | 250.2 | 4 | 300.2 | 3 | 350.2 | 2 | 400.2 | 3 |
| 150.3 | 2 | 200.3 | 4 | 250.3 | 4 | 300.3 | 3 | 350.3 | 2 | 400.3 | 4 |
| 150.4 | 1 | 200.4 | 4 | 250.4 | 4 | 300.4 | 3 | 350.4 | 2 | 400.4 | 2 |
| 150.5 | 1 | 200.5 | 4 | 250.5 | 4 | 300.5 | 1 | 350.5 | 2 | 400.5 | 2 |
| 150.6 | 1 | 200.6 | 3 | 250.6 | 4 | 300.6 | 3 | 350.6 | 4 | 400.6 | 2 |
| 150.7 | 1 | 200.7 | 3 | 250.7 | 4 | 300.7 | 3 | 350.7 | 4 | 400.7 | 2 |
| 150.8 | 1 | 200.8 | 1 | 250.8 | 4 | 300.8 | 3 | 350.8 | 4 | 400.8 | 2 |
| 150.9 | 1 | 200.9 | 1 | 250.9 | 2 | 300.9 | 3 | 350.9 | 2 | 400.9 | 2 |
| 151 | 1 | 201 | 1 | 251 | 3 | 301 | 3 | 351 | 2 | 401 | 2 |
| 151.1 | 1 | 201.1 | 1 | 251.1 | 4 | 301.1 | 3 | 351.1 | 4 | 401.1 | 4 |
| 151.2 | 1 | 201.2 | 4 | 251.2 | 4 | 301.2 | 4 | 351.2 | 2 | 401.2 | 4 |
| 151.3 | 1 | 201.3 | 4 | 251.3 | 4 | 301.3 | 1 | 351.3 | 2 | 401.3 | 4 |
| 151.4 | 1 | 201.4 | 1 | 251.4 | 4 | 301.4 | 1 | 351.4 | 2 | 401.4 | 4 |
| 151.5 | 1 | 201.5 | 4 | 251.5 | 4 | 301.5 | 1 | 351.5 | 4 | 401.5 | 4 |
| 151.6 | 1 | 201.6 | 4 | 251.6 | 4 | 301.6 | 1 | 351.6 | 4 | 401.6 | 4 |
| 151.7 | 1 | 201.7 | 4 | 251.7 | 4 | 301.7 | 3 | 351.7 | 4 | 401.7 | 4 |
| 151.8 | 1 | 201.8 | 3 | 251.8 | 4 | 301.8 | 3 | 351.8 | 2 | 401.8 | 4 |
| 151.9 | 4 | 201.9 | 3 | 251.9 | 4 | 301.9 | 3 | 351.9 | 1 | 401.9 | 4 |
| 152 | 1 | 202 | 4 | 252 | 4 | 302 | 3 | 352 | 1 | 402 | 4 |
| 152.1 | 1 | 202.1 | 4 | 252.1 | 4 | 302.1 | 4 | 352.1 | 1 | 402.1 | 4 |
| 152.2 | 3 | 202.2 | 4 | 252.2 | 4 | 302.2 | 4 | 352.2 | 4 | 402.2 | 4 |
| 152.3 | 4 | 202.3 | 4 | 252.3 | 4 | 302.3 | 4 | 352.3 | 4 | 402.3 | 4 |
| 152.4 | 1 | 202.4 | 4 | 252.4 | 4 | 302.4 | 1 | 352.4 | 4 | 402.4 | 4 |
| 152.5 | 4 | 202.5 | 1 | 252.5 | 4 | 302.5 | 1 | 352.5 | 4 | 402.5 | 4 |
| 152.6 | 1 | 202.6 | 1 | 252.6 | 4 | 302.6 | 1 | 352.6 | 4 | 402.6 | 4 |
| 152.7 | 4 | 202.7 | 1 | 252.7 | 4 | 302.7 | 1 | 352.7 | 4 | 402.7 | 4 |
| 152.8 | 2 | 202.8 | 2 | 252.8 | 1 | 302.8 | 1 | 352.8 | 4 | 402.8 | 3 |
| 152.9 | 2 | 202.9 | 2 | 252.9 | 3 | 302.9 | 1 | 352.9 | 4 | 402.9 | 3 |
| 153 | 2 | 203 | 2 | 253 | 3 | 303 | 1 | 353 | 4 | 403 | 3 |
| 153.1 | 2 | 203.1 | 3 | 253.1 | 3 | 303.1 | 1 | 353.1 | 4 | 403.1 | 4 |
| 153.2 | 2 | 203.2 | 3 | 253.2 | 3 | 303.2 | 1 | 353.2 | 4 | 403.2 | 2 |
| 153.3 | 2 | 203.3 | 2 | 253.3 | 3 | 303.3 | 1 | 353.3 | 4 | 403.3 | 2 |
| 153.4 | 2 | 203.4 | 1 | 253.4 | 3 | 303.4 | 4 | 353.4 | 4 | 403.4 | 2 |
| 153.5 | 2 | 203.5 | 1 | 253.5 | 3 | 303.5 | 2 | 353.5 | 4 | 403.5 | 2 |
| 153.6 | 2 | 203.6 | 4 | 253.6 | 4 | 303.6 | 2 | 353.6 | 4 | 403.6 | 2 |
| 153.7 | 2 | 203.7 | 4 | 253.7 | 4 | 303.7 | 2 | 353.7 | 4 | 403.7 | 2 |
| 153.8 | 2 | 203.8 | 4 | 253.8 | 4 | 303.8 | 2 | 353.8 | 2 | 403.8 | 2 |
| 153.9 | 4 | 203.9 | 4 | 253.9 | 4 | 303.9 | 3 | 353.9 | 2 | 403.9 | 3 |
| 154 | 4 | 204 | 2 | 254 | 3 | 304 | 3 | 354 | 4 | 404 | 3 |
| 154.1 | 4 | 204.1 | 4 | 254.1 | 3 | 304.1 | 3 | 354.1 | 4 | 404.1 | 4 |
| 154.2 | 4 | 204.2 | 4 | 254.2 | 3 | 304.2 | 3 | 354.2 | 4 | 404.2 | 4 |
| 154.3 | 2 | 204.3 | 2 | 254.3 | 4 | 304.3 | 3 | 354.3 | 4 | 404.3 | 4 |
| 154.4 | 2 | 204.4 | 2 | 254.4 | 4 | 304.4 | 3 | 354.4 | 4 | 404.4 | 4 |
| 154.5 | 2 | 204.5 | 2 | 254.5 | 4 | 304.5 | 3 | 354.5 | 4 | 404.5 | 2 |
| 154.6 | 2 | 204.6 | 4 | 254.6 | 4 | 304.6 | 2 | 354.6 | 4 | 404.6 | 4 |
| 154.7 | 3 | 204.7 | 4 | 254.7 | 3 | 304.7 | 2 | 354.7 | 4 | 404.7 | 3 |
| 154.8 | 3 | 204.8 | 2 | 254.8 | 3 | 304.8 | 3 | 354.8 | 4 | 404.8 | 4 |
| 154.9 | 3 | 204.9 | 4 | 254.9 | 3 | 304.9 | 3 | 354.9 | 4 | 404.9 | 4 |
| 155 | 3 | 205 | 4 | 255 | 3 | 305 | 4 | 355 | 4 | 405 | 4 |
| 155.1 | 3 | 205.1 | 4 | 255.1 | 2 | 305.1 | 3 | 355.1 | 2 | 405.1 | 4 |
| 155.2 | 3 | 205.2 | 4 | 255.2 | 2 | 305.2 | 3 | 355.2 | 2 | 405.2 | 4 |
| 155.3 | 3 | 205.3 | 4 | 255.3 | 2 | 305.3 | 3 | 355.3 | 2 | 405.3 | 4 |
| 155.4 | 3 | 205.4 | 1 | 255.4 | 2 | 305.4 | 3 | 355.4 | 4 | 405.4 | 3 |
| 155.5 | 3 | 205.5 | 1 | 255.5 | 4 | 305.5 | 3 | 355.5 | 4 | 405.5 | 3 |
| 155.6 | 3 | 205.6 | 1 | 255.6 | 4 | 305.6 | 3 | 355.6 | 3 |  |  |
| 155.7 | 3 | 205.7 | 1 | 255.7 | 4 | 305.7 | 3 | 355.7 | 3 |  |  |
| 155.8 | 3 | 205.8 | 4 | 255.8 | 4 | 305.8 | 2 | 355.8 | 4 |  |  |
| 155.9 | 3 | 205.9 | 2 | 255.9 | 2 | 305.9 | 3 | 355.9 | 4 |  |  |
| 156 | 3 | 206 | 4 | 256 | 2 | 306 | 3 | 356 | 4 |  |  |
| 156.1 | 3 | 206.1 | 4 | 256.1 | 4 | 306.1 | 3 | 356.1 | 4 |  |  |
| 156.2 | 3 | 206.2 | 2 | 256.2 | 4 | 306.2 | 3 | 356.2 | 4 |  |  |
| 156.3 | 3 | 206.3 | 2 | 256.3 | 4 | 306.3 | 3 | 356.3 | 4 |  |  |
| 156.4 | 3 | 206.4 | 2 | 256.4 | 3 | 306.4 | 2 | 356.4 | 4 |  |  |
| 156.5 | 3 | 206.5 | 4 | 256.5 | 3 | 306.5 | 2 | 356.5 | 4 |  |  |
| 156.6 | 3 | 206.6 | 4 | 256.6 | 2 | 306.6 | 4 | 356.6 | 4 |  |  |
| 156.7 | 3 | 206.7 | 4 | 256.7 | 2 | 306.7 | 4 | 356.7 | 4 |  |  |
| 156.8 | 3 | 206.8 | 2 | 256.8 | 2 | 306.8 | 4 | 356.8 | 4 |  |  |
| 156.9 | 3 | 206.9 | 2 | 256.9 | 2 | 306.9 | 4 | 356.9 | 4 |  |  |
| 157 | 3 | 207 | 2 | 257 | 1 | 307 | 4 | 357 | 4 |  |  |
| 157.1 | 3 | 207.1 | 4 | 257.1 | 1 | 307.1 | 4 | 357.1 | 2 |  |  |
| 157.2 | 3 | 207.2 | 4 | 257.2 | 1 | 307.2 | 4 | 357.2 | 2 |  |  |
| 157.3 | 3 | 207.3 | 4 | 257.3 | 1 | 307.3 | 3 | 357.3 | 1 |  |  |
| 157.4 | 3 | 207.4 | 4 | 257.4 | 1 | 307.4 | 3 | 357.4 | 1 |  |  |
| 157.5 | 3 | 207.5 | 4 | 257.5 | 1 | 307.5 | 1 | 357.5 | 1 |  |  |
| 157.6 | 4 | 207.6 | 4 | 257.6 | 1 | 307.6 | 1 | 357.6 | 1 |  |  |
| 157.7 | 4 | 207.7 | 2 | 257.7 | 1 | 307.7 | 1 | 357.7 | 1 |  |  |
| 157.8 | 4 | 207.8 | 2 | 257.8 | 1 | 307.8 | 1 | 357.8 | 1 |  |  |
| 157.9 | 4 | 207.9 | 4 | 257.9 | 1 | 307.9 | 4 | 357.9 | 1 |  |  |
| 158 | 4 | 208 | 4 | 258 | 1 | 308 | 4 | 358 | 1 |  |  |
| 158.1 | 4 | 208.1 | 4 | 258.1 | 1 | 308.1 | 4 | 358.1 | 2 |  |  |
| 158.2 | 4 | 208.2 | 4 | 258.2 | 1 | 308.2 | 1 | 358.2 | 2 |  |  |
| 158.3 | 4 | 208.3 | 4 | 258.3 | 1 | 308.3 | 1 | 358.3 | 2 |  |  |
| 158.4 | 4 | 208.4 | 4 | 258.4 | 1 | 308.4 | 1 | 358.4 | 2 |  |  |
| 158.5 | 4 | 208.5 | 4 | 258.5 | 1 | 308.5 | 4 | 358.5 | 2 |  |  |
| 158.6 | 4 | 208.6 | 4 | 258.6 | 2 | 308.6 | 4 | 358.6 | 2 |  |  |
| 158.7 | 4 | 208.7 | 4 | 258.7 | 3 | 308.7 | 4 | 358.7 | 3 |  |  |
| 158.8 | 4 | 208.8 | 4 | 258.8 | 4 | 308.8 | 4 | 358.8 | 4 |  |  |
| 158.9 | 4 | 208.9 | 4 | 258.9 | 4 | 308.9 | 4 | 358.9 | 4 |  |  |
| 159 | 4 | 209 | 4 | 259 | 2 | 309 | 4 | 359 | 2 |  |  |
| 159.1 | 4 | 209.1 | 4 | 259.1 | 2 | 309.1 | 4 | 359.1 | 2 |  |  |
| 159.2 | 4 | 209.2 | 4 | 259.2 | 2 | 309.2 | 4 | 359.2 | 3 |  |  |
| 159.3 | 4 | 209.3 | 4 | 259.3 | 4 | 309.3 | 4 | 359.3 | 2 |  |  |
| 159.4 | 4 | 209.4 | 4 | 259.4 | 4 | 309.4 | 4 | 359.4 | 4 |  |  |
| 159.5 | 4 | 209.5 | 4 | 259.5 | 4 | 309.5 | 4 | 359.5 | 4 |  |  |
| 159.6 | 4 | 209.6 | 4 | 259.6 | 4 | 309.6 | 4 | 359.6 | 2 |  |  |
| 159.7 | 4 | 209.7 | 4 | 259.7 | 4 | 309.7 | 2 | 359.7 | 2 |  |  |
| 159.8 | 4 | 209.8 | 4 | 259.8 | 4 | 309.8 | 4 | 359.8 | 2 |  |  |
| 159.9 | 4 | 209.9 | 4 | 259.9 | 4 | 309.9 | 4 | 359.9 | 4 |  |  |
| 160 | 4 | 210 | 4 | 260 | 4 | 310 | 2 | 360 | 4 |  |  |
| 160.1 | 4 | 210.1 | 4 | 260.1 | 2 | 310.1 | 2 | 360.1 | 2 |  |  |
| 160.2 | 4 | 210.2 | 4 | 260.2 | 2 | 310.2 | 1 | 360.2 | 2 |  |  |
| 160.3 | 4 | 210.3 | 4 | 260.3 | 4 | 310.3 | 4 | 360.3 | 3 |  |  |
| 160.4 | 4 | 210.4 | 4 | 260.4 | 4 | 310.4 | 4 | 360.4 | 3 |  |  |
| 160.5 | 4 | 210.5 | 4 | 260.5 | 4 | 310.5 | 2 | 360.5 | 1 |  |  |
| 160.6 | 3 | 210.6 | 4 | 260.6 | 4 | 310.6 | 2 | 360.6 | 4 |  |  |
| 160.7 | 3 | 210.7 | 4 | 260.7 | 4 | 310.7 | 3 | 360.7 | 4 |  |  |
| 160.8 | 3 | 210.8 | 4 | 260.8 | 2 | 310.8 | 3 | 360.8 | 4 |  |  |
| 160.9 | 3 | 210.9 | 4 | 260.9 | 2 | 310.9 | 3 | 360.9 | 4 |  |  |
| 161 | 3 | 211 | 4 | 261 | 2 | 311 | 4 | 361 | 4 |  |  |
| 161.1 | 3 | 211.1 | 3 | 261.1 | 2 | 311.1 | 4 | 361.1 | 4 |  |  |
| 161.2 | 3 | 211.2 | 3 | 261.2 | 2 | 311.2 | 4 | 361.2 | 4 |  |  |
| 161.3 | 3 | 211.3 | 3 | 261.3 | 2 | 311.3 | 1 | 361.3 | 4 |  |  |
| 161.4 | 3 | 211.4 | 3 | 261.4 | 4 | 311.4 | 4 | 361.4 | 4 |  |  |
| 161.5 | 3 | 211.5 | 3 | 261.5 | 4 | 311.5 | 4 | 361.5 | 4 |  |  |
| 161.6 | 3 | 211.6 | 2 | 261.6 | 4 | 311.6 | 1 | 361.6 | 1 |  |  |
| 161.7 | 4 | 211.7 | 2 | 261.7 | 4 | 311.7 | 1 | 361.7 | 1 |  |  |
| 161.8 | 4 | 211.8 | 2 | 261.8 | 4 | 311.8 | 1 | 361.8 | 1 |  |  |
| 161.9 | 4 | 211.9 | 2 | 261.9 | 4 | 311.9 | 1 | 361.9 | 1 |  |  |
| 162 | 4 | 212 | 2 | 262 | 4 | 312 | 4 | 362 | 1 |  |  |
| 162.1 | 4 | 212.1 | 2 | 262.1 | 4 | 312.1 | 4 | 362.1 | 1 |  |  |
| 162.2 | 4 | 212.2 | 2 | 262.2 | 4 | 312.2 | 4 | 362.2 | 1 |  |  |
| 162.3 | 4 | 212.3 | 1 | 262.3 | 4 | 312.3 | 2 | 362.3 | 1 |  |  |
| 162.4 | 4 | 212.4 | 1 | 262.4 | 4 | 312.4 | 2 | 362.4 | 1 |  |  |
| 162.5 | 4 | 212.5 | 1 | 262.5 | 4 | 312.5 | 3 | 362.5 | 1 |  |  |
| 162.6 | 4 | 212.6 | 1 | 262.6 | 4 | 312.6 | 3 | 362.6 | 4 |  |  |
| 162.7 | 4 | 212.7 | 1 | 262.7 | 4 | 312.7 | 3 | 362.7 | 4 |  |  |
| 162.8 | 4 | 212.8 | 1 | 262.8 | 4 | 312.8 | 3 | 362.8 | 4 |  |  |
| 162.9 | 4 | 212.9 | 1 | 262.9 | 4 | 312.9 | 3 | 362.9 | 2 |  |  |
| 163 | 4 | 213 | 1 | 263 | 4 | 313 | 3 | 363 | 4 |  |  |
| 163.1 | 4 | 213.1 | 1 | 263.1 | 4 | 313.1 | 3 | 363.1 | 4 |  |  |
| 163.2 | 4 | 213.2 | 1 | 263.2 | 1 | 313.2 | 3 | 363.2 | 4 |  |  |
| 163.3 | 4 | 213.3 | 1 | 263.3 | 1 | 313.3 | 3 | 363.3 | 2 |  |  |
| 163.4 | 4 | 213.4 | 1 | 263.4 | 1 | 313.4 | 3 | 363.4 | 2 |  |  |
| 163.5 | 4 | 213.5 | 1 | 263.5 | 4 | 313.5 | 3 | 363.5 | 4 |  |  |
| 163.6 | 4 | 213.6 | 1 | 263.6 | 1 | 313.6 | 3 | 363.6 | 4 |  |  |
| 163.7 | 4 | 213.7 | 4 | 263.7 | 4 | 313.7 | 3 | 363.7 | 4 |  |  |
| 163.8 | 4 | 213.8 | 1 | 263.8 | 1 | 313.8 | 3 | 363.8 | 4 |  |  |
| 163.9 | 4 | 213.9 | 1 | 263.9 | 4 | 313.9 | 3 | 363.9 | 2 |  |  |
| 164 | 4 | 214 | 1 | 264 | 4 | 314 | 3 | 364 | 2 |  |  |
| 164.1 | 4 | 214.1 | 4 | 264.1 | 4 | 314.1 | 3 | 364.1 | 4 |  |  |
| 164.2 | 4 | 214.2 | 4 | 264.2 | 4 | 314.2 | 3 | 364.2 | 4 |  |  |
| 164.3 | 3 | 214.3 | 4 | 264.3 | 3 | 314.3 | 4 | 364.3 | 4 |  |  |
| 164.4 | 3 | 214.4 | 4 | 264.4 | 2 | 314.4 | 4 | 364.4 | 2 |  |  |
| 164.5 | 3 | 214.5 | 4 | 264.5 | 1 | 314.5 | 4 | 364.5 | 2 |  |  |
| 164.6 | 3 | 214.6 | 4 | 264.6 | 1 | 314.6 | 3 | 364.6 | 4 |  |  |
| 164.7 | 3 | 214.7 | 4 | 264.7 | 1 | 314.7 | 3 | 364.7 | 4 |  |  |
| 164.8 | 3 | 214.8 | 4 | 264.8 | 1 | 314.8 | 3 | 364.8 | 4 |  |  |
| 164.9 | 3 | 214.9 | 4 | 264.9 | 4 | 314.9 | 3 | 364.9 | 4 |  |  |
| 165 | 3 | 215 | 2 | 265 | 1 | 315 | 4 | 365 | 2 |  |  |
| 165.1 | 3 | 215.1 | 2 | 265.1 | 4 | 315.1 | 4 | 365.1 | 2 |  |  |
| 165.2 | 3 | 215.2 | 4 | 265.2 | 4 | 315.2 | 4 | 365.2 | 4 |  |  |
| 165.3 | 3 | 215.3 | 2 | 265.3 | 1 | 315.3 | 4 | 365.3 | 2 |  |  |
| 165.4 | 3 | 215.4 | 2 | 265.4 | 1 | 315.4 | 4 | 365.4 | 4 |  |  |
| 165.5 | 3 | 215.5 | 4 | 265.5 | 1 | 315.5 | 4 | 365.5 | 4 |  |  |
| 165.6 | 3 | 215.6 | 4 | 265.6 | 4 | 315.6 | 4 | 365.6 | 4 |  |  |
| 165.7 | 3 | 215.7 | 4 | 265.7 | 1 | 315.7 | 3 | 365.7 | 4 |  |  |
| 165.8 | 3 | 215.8 | 4 | 265.8 | 4 | 315.8 | 3 | 365.8 | 4 |  |  |
| 165.9 | 3 | 215.9 | 4 | 265.9 | 1 | 315.9 | 3 | 365.9 | 4 |  |  |
| 166 | 3 | 216 | 4 | 266 | 4 | 316 | 3 | 366 | 4 |  |  |
| 166.1 | 3 | 216.1 | 4 | 266.1 | 4 | 316.1 | 1 | 366.1 | 4 |  |  |
| 166.2 | 3 | 216.2 | 4 | 266.2 | 4 | 316.2 | 1 | 366.2 | 4 |  |  |
| 166.3 | 3 | 216.3 | 4 | 266.3 | 4 | 316.3 | 1 | 366.3 | 4 |  |  |
| 166.4 | 3 | 216.4 | 4 | 266.4 | 4 | 316.4 | 1 | 366.4 | 4 |  |  |
| 166.5 | 3 | 216.5 | 4 | 266.5 | 4 | 316.5 | 1 | 366.5 | 4 |  |  |
| 166.6 | 3 | 216.6 | 1 | 266.6 | 4 | 316.6 | 1 | 366.6 | 3 |  |  |
| 166.7 | 3 | 216.7 | 1 | 266.7 | 1 | 316.7 | 1 | 366.7 | 3 |  |  |
| 166.8 | 3 | 216.8 | 1 | 266.8 | 1 | 316.8 | 1 | 366.8 | 3 |  |  |
| 166.9 | 3 | 216.9 | 1 | 266.9 | 1 | 316.9 | 1 | 366.9 | 3 |  |  |
| 167 | 3 | 217 | 1 | 267 | 2 | 317 | 3 | 367 | 2 |  |  |
| 167.1 | 3 | 217.1 | 1 | 267.1 | 2 | 317.1 | 3 | 367.1 | 2 |  |  |
| 167.2 | 3 | 217.2 | 1 | 267.2 | 2 | 317.2 | 3 | 367.2 | 2 |  |  |
| 167.3 | 3 | 217.3 | 1 | 267.3 | 1 | 317.3 | 3 | 367.3 | 2 |  |  |
| 167.4 | 3 | 217.4 | 1 | 267.4 | 1 | 317.4 | 3 | 367.4 | 3 |  |  |
| 167.5 | 3 | 217.5 | 1 | 267.5 | 1 | 317.5 | 3 | 367.5 | 3 |  |  |
| 167.6 | 3 | 217.6 | 1 | 267.6 | 2 | 317.6 | 3 | 367.6 | 3 |  |  |
| 167.7 | 3 | 217.7 | 1 | 267.7 | 4 | 317.7 | 3 | 367.7 | 3 |  |  |
| 167.8 | 3 | 217.8 | 1 | 267.8 | 4 | 317.8 | 3 | 367.8 | 4 |  |  |
| 167.9 | 3 | 217.9 | 1 | 267.9 | 4 | 317.9 | 4 | 367.9 | 4 |  |  |
| 168 | 3 | 218 | 1 | 268 | 3 | 318 | 4 | 368 | 4 |  |  |
| 168.1 | 4 | 218.1 | 1 | 268.1 | 2 | 318.1 | 4 | 368.1 | 4 |  |  |
| 168.2 | 2 | 218.2 | 1 | 268.2 | 2 | 318.2 | 4 | 368.2 | 4 |  |  |
| 168.3 | 4 | 218.3 | 1 | 268.3 | 2 | 318.3 | 4 | 368.3 | 4 |  |  |
| 168.4 | 2 | 218.4 | 1 | 268.4 | 4 | 318.4 | 4 | 368.4 | 4 |  |  |
| 168.5 | 4 | 218.5 | 1 | 268.5 | 4 | 318.5 | 4 | 368.5 | 3 |  |  |
| 168.6 | 2 | 218.6 | 1 | 268.6 | 4 | 318.6 | 4 | 368.6 | 3 |  |  |
| 168.7 | 4 | 218.7 | 1 | 268.7 | 4 | 318.7 | 4 | 368.7 | 3 |  |  |
| 168.8 | 4 | 218.8 | 1 | 268.8 | 4 | 318.8 | 4 | 368.8 | 4 |  |  |
| 168.9 | 4 | 218.9 | 1 | 268.9 | 4 | 318.9 | 4 | 368.9 | 1 |  |  |
| 169 | 2 | 219 | 1 | 269 | 4 | 319 | 4 | 369 | 1 |  |  |
| 169.1 | 4 | 219.1 | 1 | 269.1 | 3 | 319.1 | 4 | 369.1 | 1 |  |  |
| 169.2 | 2 | 219.2 | 1 | 269.2 | 3 | 319.2 | 4 | 369.2 | 3 |  |  |
| 169.3 | 2 | 219.3 | 1 | 269.3 | 3 | 319.3 | 4 | 369.3 | 3 |  |  |
| 169.4 | 2 | 219.4 | 1 | 269.4 | 3 | 319.4 | 4 | 369.4 | 3 |  |  |
| 169.5 | 4 | 219.5 | 1 | 269.5 | 3 | 319.5 | 4 | 369.5 | 3 |  |  |
| 169.6 | 4 | 219.6 | 1 | 269.6 | 2 | 319.6 | 4 | 369.6 | 3 |  |  |
| 169.7 | 4 | 219.7 | 1 | 269.7 | 2 | 319.7 | 4 | 369.7 | 3 |  |  |
| 169.8 | 4 | 219.8 | 1 | 269.8 | 2 | 319.8 | 4 | 369.8 | 3 |  |  |
| 169.9 | 4 | 219.9 | 1 | 269.9 | 2 | 319.9 | 4 | 369.9 | 3 |  |  |
| 170 | 1 | 220 | 1 | 270 | 2 | 320 | 4 | 370 | 3 |  |  |
| 170.1 | 1 | 220.1 | 1 | 270.1 | 2 | 320.1 | 4 | 370.1 | 3 |  |  |
| 170.2 | 1 | 220.2 | 1 | 270.2 | 2 | 320.2 | 4 | 370.2 | 2 |  |  |
| 170.3 | 1 | 220.3 | 1 | 270.3 | 2 | 320.3 | 4 | 370.3 | 2 |  |  |
| 170.4 | 4 | 220.4 | 1 | 270.4 | 2 | 320.4 | 4 | 370.4 | 2 |  |  |
| 170.5 | 4 | 220.5 | 1 | 270.5 | 2 | 320.5 | 4 | 370.5 | 2 |  |  |
| 170.6 | 4 | 220.6 | 1 | 270.6 | 2 | 320.6 | 4 | 370.6 | 2 |  |  |
| 170.7 | 1 | 220.7 | 1 | 270.7 | 1 | 320.7 | 4 | 370.7 | 4 |  |  |
| 170.8 | 1 | 220.8 | 1 | 270.8 | 1 | 320.8 | 4 | 370.8 | 4 |  |  |
| 170.9 | 1 | 220.9 | 1 | 270.9 | 1 | 320.9 | 4 | 370.9 | 2 |  |  |
| 171 | 4 | 221 | 4 | 271 | 1 | 321 | 4 | 371 | 2 |  |  |
| 171.1 | 4 | 221.1 | 4 | 271.1 | 1 | 321.1 | 4 | 371.1 | 2 |  |  |
| 171.2 | 4 | 221.2 | 4 | 271.2 | 1 | 321.2 | 4 | 371.2 | 2 |  |  |
| 171.3 | 1 | 221.3 | 4 | 271.3 | 1 | 321.3 | 4 | 371.3 | 4 |  |  |
| 171.4 | 1 | 221.4 | 4 | 271.4 | 1 | 321.4 | 4 | 371.4 | 1 |  |  |
| 171.5 | 1 | 221.5 | 4 | 271.5 | 1 | 321.5 | 4 | 371.5 | 1 |  |  |
| 171.6 | 1 | 221.6 | 4 | 271.6 | 1 | 321.6 | 4 | 371.6 | 1 |  |  |
| 171.7 | 1 | 221.7 | 4 | 271.7 | 1 | 321.7 | 4 | 371.7 | 4 |  |  |
| 171.8 | 1 | 221.8 | 4 | 271.8 | 1 | 321.8 | 4 | 371.8 | 1 |  |  |
| 171.9 | 1 | 221.9 | 4 | 271.9 | 1 | 321.9 | 4 | 371.9 | 1 |  |  |
| 172 | 1 | 222 | 4 | 272 | 1 | 322 | 4 | 372 | 1 |  |  |
| 172.1 | 1 | 222.1 | 4 | 272.1 | 1 | 322.1 | 4 | 372.1 | 1 |  |  |
| 172.2 | 1 | 222.2 | 4 | 272.2 | 1 | 322.2 | 4 | 372.2 | 4 |  |  |
| 172.3 | 1 | 222.3 | 4 | 272.3 | 2 | 322.3 | 4 | 372.3 | 3 |  |  |
| 172.4 | 1 | 222.4 | 4 | 272.4 | 3 | 322.4 | 4 | 372.4 | 4 |  |  |
| 172.5 | 1 | 222.5 | 4 | 272.5 | 4 | 322.5 | 4 | 372.5 | 4 |  |  |
| 172.6 | 4 | 222.6 | 4 | 272.6 | 4 | 322.6 | 4 | 372.6 | 4 |  |  |
| 172.7 | 2 | 222.7 | 4 | 272.7 | 4 | 322.7 | 4 | 372.7 | 1 |  |  |
| 172.8 | 4 | 222.8 | 4 | 272.8 | 4 | 322.8 | 4 | 372.8 | 1 |  |  |
| 172.9 | 2 | 222.9 | 4 | 272.9 | 4 | 322.9 | 3 | 372.9 | 4 |  |  |
| 173 | 2 | 223 | 4 | 273 | 4 | 323 | 3 | 373 | 2 |  |  |
| 173.1 | 4 | 223.1 | 4 | 273.1 | 3 | 323.1 | 3 | 373.1 | 2 |  |  |
| 173.2 | 4 | 223.2 | 4 | 273.2 | 2 | 323.2 | 3 | 373.2 | 2 |  |  |
| 173.3 | 2 | 223.3 | 4 | 273.3 | 1 | 323.3 | 3 | 373.3 | 2 |  |  |
| 173.4 | 2 | 223.4 | 4 | 273.4 | 1 | 323.4 | 2 | 373.4 | 2 |  |  |
| 173.5 | 2 | 223.5 | 4 | 273.5 | 1 | 323.5 | 2 | 373.5 | 2 |  |  |
| 173.6 | 2 | 223.6 | 4 | 273.6 | 1 | 323.6 | 2 | 373.6 | 2 |  |  |
| 173.7 | 4 | 223.7 | 4 | 273.7 | 1 | 323.7 | 2 | 373.7 | 2 |  |  |
| 173.8 | 4 | 223.8 | 4 | 273.8 | 1 | 323.8 | 2 | 373.8 | 4 |  |  |
| 173.9 | 4 | 223.9 | 4 | 273.9 | 1 | 323.9 | 2 | 373.9 | 1 |  |  |
| 174 | 4 | 224 | 4 | 274 | 1 | 324 | 2 | 374 | 1 |  |  |
| 174.1 | 2 | 224.1 | 4 | 274.1 | 1 | 324.1 | 2 | 374.1 | 1 |  |  |
| 174.2 | 2 | 224.2 | 4 | 274.2 | 1 | 324.2 | 2 | 374.2 | 1 |  |  |
| 174.3 | 2 | 224.3 | 4 | 274.3 | 1 | 324.3 | 2 | 374.3 | 1 |  |  |
| 174.4 | 2 | 224.4 | 4 | 274.4 | 4 | 324.4 | 2 | 374.4 | 1 |  |  |
| 174.5 | 2 | 224.5 | 4 | 274.5 | 4 | 324.5 | 3 | 374.5 | 1 |  |  |
| 174.6 | 2 | 224.6 | 4 | 274.6 | 4 | 324.6 | 3 | 374.6 | 1 |  |  |
| 174.7 | 3 | 224.7 | 4 | 274.7 | 4 | 324.7 | 3 | 374.7 | 1 |  |  |
| 174.8 | 3 | 224.8 | 4 | 274.8 | 4 | 324.8 | 3 | 374.8 | 1 |  |  |
| 174.9 | 3 | 224.9 | 4 | 274.9 | 4 | 324.9 | 3 | 374.9 | 1 |  |  |
| 175 | 3 | 225 | 4 | 275 | 4 | 325 | 3 | 375 | 1 |  |  |
| 175.1 | 3 | 225.1 | 4 | 275.1 | 4 | 325.1 | 3 | 375.1 | 1 |  |  |
| 175.2 | 3 | 225.2 | 4 | 275.2 | 4 | 325.2 | 3 | 375.2 | 1 |  |  |
| 175.3 | 3 | 225.3 | 4 | 275.3 | 4 | 325.3 | 3 | 375.3 | 1 |  |  |
| 175.4 | 3 | 225.4 | 4 | 275.4 | 4 | 325.4 | 3 | 375.4 | 1 |  |  |
| 175.5 | 3 | 225.5 | 4 | 275.5 | 3 | 325.5 | 3 | 375.5 | 1 |  |  |
| 175.6 | 2 | 225.6 | 4 | 275.6 | 3 | 325.6 | 3 | 375.6 | 1 |  |  |
| 175.7 | 2 | 225.7 | 4 | 275.7 | 2 | 325.7 | 3 | 375.7 | 1 |  |  |
| 175.8 | 2 | 225.8 | 4 | 275.8 | 1 | 325.8 | 2 | 375.8 | 1 |  |  |
| 175.9 | 2 | 225.9 | 4 | 275.9 | 1 | 325.9 | 2 | 375.9 | 1 |  |  |
| 176 | 2 | 226 | 4 | 276 | 4 | 326 | 1 | 376 | 1 |  |  |
| 176.1 | 2 | 226.1 | 4 | 276.1 | 4 | 326.1 | 1 | 376.1 | 1 |  |  |
| 176.2 | 2 | 226.2 | 4 | 276.2 | 4 | 326.2 | 1 | 376.2 | 1 |  |  |
| 176.3 | 2 | 226.3 | 4 | 276.3 | 4 | 326.3 | 1 | 376.3 | 1 |  |  |
| 176.4 | 2 | 226.4 | 4 | 276.4 | 4 | 326.4 | 1 | 376.4 | 1 |  |  |
| 176.5 | 2 | 226.5 | 4 | 276.5 | 4 | 326.5 | 2 | 376.5 | 1 |  |  |
| 176.6 | 2 | 226.6 | 4 | 276.6 | 1 | 326.6 | 2 | 376.6 | 1 |  |  |
| 176.7 | 2 | 226.7 | 4 | 276.7 | 1 | 326.7 | 2 | 376.7 | 1 |  |  |
| 176.8 | 2 | 226.8 | 4 | 276.8 | 1 | 326.8 | 2 | 376.8 | 1 |  |  |
| 176.9 | 2 | 226.9 | 4 | 276.9 | 1 | 326.9 | 2 | 376.9 | 1 |  |  |
| 177 | 2 | 227 | 4 | 277 | 1 | 327 | 2 | 377 | 1 |  |  |
| 177.1 | 4 | 227.1 | 4 | 277.1 | 1 | 327.1 | 4 | 377.1 | 1 |  |  |
| 177.2 | 2 | 227.2 | 4 | 277.2 | 1 | 327.2 | 4 | 377.2 | 1 |  |  |
| 177.3 | 4 | 227.3 | 4 | 277.3 | 1 | 327.3 | 2 | 377.3 | 1 |  |  |
| 177.4 | 4 | 227.4 | 4 | 277.4 | 1 | 327.4 | 4 | 377.4 | 1 |  |  |
| 177.5 | 4 | 227.5 | 4 | 277.5 | 1 | 327.5 | 4 | 377.5 | 1 |  |  |
| 177.6 | 4 | 227.6 | 4 | 277.6 | 1 | 327.6 | 1 | 377.6 | 1 |  |  |
| 177.7 | 4 | 227.7 | 4 | 277.7 | 1 | 327.7 | 1 | 377.7 | 1 |  |  |
| 177.8 | 4 | 227.8 | 4 | 277.8 | 1 | 327.8 | 1 | 377.8 | 1 |  |  |
| 177.9 | 4 | 227.9 | 4 | 277.9 | 1 | 327.9 | 1 | 377.9 | 2 |  |  |
| 178 | 4 | 228 | 4 | 278 | 4 | 328 | 1 | 378 | 2 |  |  |
| 178.1 | 4 | 228.1 | 4 | 278.1 | 1 | 328.1 | 1 | 378.1 | 2 |  |  |
| 178.2 | 4 | 228.2 | 4 | 278.2 | 1 | 328.2 | 4 | 378.2 | 2 |  |  |
| 178.3 | 4 | 228.3 | 4 | 278.3 | 1 | 328.3 | 1 | 378.3 | 2 |  |  |
| 178.4 | 4 | 228.4 | 4 | 278.4 | 1 | 328.4 | 1 | 378.4 | 4 |  |  |
| 178.5 | 4 | 228.5 | 4 | 278.5 | 1 | 328.5 | 1 | 378.5 | 4 |  |  |
| 178.6 | 2 | 228.6 | 4 | 278.6 | 1 | 328.6 | 3 | 378.6 | 4 |  |  |
| 178.7 | 2 | 228.7 | 4 | 278.7 | 1 | 328.7 | 1 | 378.7 | 4 |  |  |
| 178.8 | 2 | 228.8 | 4 | 278.8 | 1 | 328.8 | 1 | 378.8 | 4 |  |  |
| 178.9 | 4 | 228.9 | 4 | 278.9 | 1 | 328.9 | 1 | 378.9 | 3 |  |  |
| 179 | 4 | 229 | 4 | 279 | 1 | 329 | 1 | 379 | 3 |  |  |
| 179.1 | 4 | 229.1 | 4 | 279.1 | 1 | 329.1 | 1 | 379.1 | 4 |  |  |
| 179.2 | 4 | 229.2 | 4 | 279.2 | 1 | 329.2 | 1 | 379.2 | 4 |  |  |
| 179.3 | 4 | 229.3 | 4 | 279.3 | 1 | 329.3 | 1 | 379.3 | 4 |  |  |
| 179.4 | 4 | 229.4 | 4 | 279.4 | 1 | 329.4 | 1 | 379.4 | 4 |  |  |
| 179.5 | 4 | 229.5 | 4 | 279.5 | 1 | 329.5 | 1 | 379.5 | 4 |  |  |
| 179.6 | 2 | 229.6 | 4 | 279.6 | 1 | 329.6 | 2 | 379.6 | 3 |  |  |
| 179.7 | 4 | 229.7 | 4 | 279.7 | 1 | 329.7 | 2 | 379.7 | 4 |  |  |
| 179.8 | 2 | 229.8 | 4 | 279.8 | 1 | 329.8 | 2 | 379.8 | 4 |  |  |
| 179.9 | 4 | 229.9 | 4 | 279.9 | 1 | 329.9 | 2 | 379.9 | 4 |  |  |
| 180 | 4 | 230 | 4 | 280 | 1 | 330 | 2 | 380 | 4 |  |  |
| 180.1 | 4 | 230.1 | 4 | 280.1 | 4 | 330.1 | 3 | 380.1 | 4 |  |  |
| 180.2 | 4 | 230.2 | 4 | 280.2 | 4 | 330.2 | 4 | 380.2 | 4 |  |  |
| 180.3 | 4 | 230.3 | 4 | 280.3 | 4 | 330.3 | 4 | 380.3 | 4 |  |  |
| 180.4 | 4 | 230.4 | 4 | 280.4 | 4 | 330.4 | 4 | 380.4 | 4 |  |  |
| 180.5 | 4 | 230.5 | 4 | 280.5 | 1 | 330.5 | 4 | 380.5 | 4 |  |  |
| 180.6 | 4 | 230.6 | 4 | 280.6 | 1 | 330.6 | 4 | 380.6 | 4 |  |  |
| 180.7 | 4 | 230.7 | 4 | 280.7 | 1 | 330.7 | 4 | 380.7 | 4 |  |  |
| 180.8 | 4 | 230.8 | 4 | 280.8 | 1 | 330.8 | 4 | 380.8 | 3 |  |  |
| 180.9 | 4 | 230.9 | 4 | 280.9 | 1 | 330.9 | 4 | 380.9 | 4 |  |  |
| 181 | 4 | 231 | 4 | 281 | 1 | 331 | 4 | 381 | 4 |  |  |
| 181.1 | 4 | 231.1 | 4 | 281.1 | 1 | 331.1 | 3 | 381.1 | 4 |  |  |
| 181.2 | 4 | 231.2 | 4 | 281.2 | 1 | 331.2 | 2 | 381.2 | 4 |  |  |
| 181.3 | 4 | 231.3 | 4 | 281.3 | 1 | 331.3 | 2 | 381.3 | 4 |  |  |
| 181.4 | 4 | 231.4 | 4 | 281.4 | 1 | 331.4 | 2 | 381.4 | 4 |  |  |
| 181.5 | 4 | 231.5 | 4 | 281.5 | 1 | 331.5 | 2 | 381.5 | 4 |  |  |
| 181.6 | 2 | 231.6 | 2 | 281.6 | 1 | 331.6 | 2 | 381.6 | 4 |  |  |
| 181.7 | 2 | 231.7 | 2 | 281.7 | 1 | 331.7 | 2 | 381.7 | 4 |  |  |
| 181.8 | 4 | 231.8 | 2 | 281.8 | 1 | 331.8 | 2 | 381.8 | 4 |  |  |
| 181.9 | 4 | 231.9 | 4 | 281.9 | 1 | 331.9 | 2 | 381.9 | 4 |  |  |
| 182 | 4 | 232 | 4 | 282 | 1 | 332 | 2 | 382 | 4 |  |  |
| 182.1 | 4 | 232.1 | 4 | 282.1 | 1 | 332.1 | 4 | 382.1 | 4 |  |  |
| 182.2 | 4 | 232.2 | 4 | 282.2 | 1 | 332.2 | 4 | 382.2 | 4 |  |  |
| 182.3 | 3 | 232.3 | 4 | 282.3 | 1 | 332.3 | 4 | 382.3 | 4 |  |  |
| 182.4 | 2 | 232.4 | 2 | 282.4 | 3 | 332.4 | 4 | 382.4 | 3 |  |  |
| 182.5 | 1 | 232.5 | 2 | 282.5 | 1 | 332.5 | 4 | 382.5 | 3 |  |  |
| 182.6 | 1 | 232.6 | 4 | 282.6 | 1 | 332.6 | 4 | 382.6 | 4 |  |  |
| 182.7 | 4 | 232.7 | 4 | 282.7 | 1 | 332.7 | 4 | 382.7 | 4 |  |  |
| 182.8 | 1 | 232.8 | 2 | 282.8 | 1 | 332.8 | 4 | 382.8 | 4 |  |  |
| 182.9 | 1 | 232.9 | 2 | 282.9 | 1 | 332.9 | 4 | 382.9 | 4 |  |  |
| 183 | 1 | 233 | 2 | 283 | 1 | 333 | 4 | 383 | 4 |  |  |
| 183.1 | 1 | 233.1 | 2 | 283.1 | 1 | 333.1 | 4 | 383.1 | 2 |  |  |
| 183.2 | 1 | 233.2 | 2 | 283.2 | 1 | 333.2 | 4 | 383.2 | 2 |  |  |
| 183.3 | 1 | 233.3 | 2 | 283.3 | 2 | 333.3 | 4 | 383.3 | 2 |  |  |
| 183.4 | 1 | 233.4 | 2 | 283.4 | 1 | 333.4 | 4 | 383.4 | 2 |  |  |
| 183.5 | 1 | 233.5 | 2 | 283.5 | 1 | 333.5 | 2 | 383.5 | 2 |  |  |
| 183.6 | 1 | 233.6 | 2 | 283.6 | 1 | 333.6 | 2 | 383.6 | 2 |  |  |
| 183.7 | 1 | 233.7 | 2 | 283.7 | 1 | 333.7 | 2 | 383.7 | 2 |  |  |
| 183.8 | 4 | 233.8 | 2 | 283.8 | 1 | 333.8 | 2 | 383.8 | 2 |  |  |
| 183.9 | 4 | 233.9 | 2 | 283.9 | 1 | 333.9 | 2 | 383.9 | 2 |  |  |
| 184 | 4 | 234 | 2 | 284 | 1 | 334 | 4 | 384 | 2 |  |  |
| 184.1 | 4 | 234.1 | 2 | 284.1 | 1 | 334.1 | 1 | 384.1 | 2 |  |  |
| 184.2 | 4 | 234.2 | 2 | 284.2 | 1 | 334.2 | 1 | 384.2 | 2 |  |  |
| 184.3 | 4 | 234.3 | 2 | 284.3 | 1 | 334.3 | 1 | 384.3 | 2 |  |  |
| 184.4 | 4 | 234.4 | 2 | 284.4 | 1 | 334.4 | 4 | 384.4 | 2 |  |  |
| 184.5 | 4 | 234.5 | 2 | 284.5 | 1 | 334.5 | 4 | 384.5 | 2 |  |  |
| 184.6 | 4 | 234.6 | 2 | 284.6 | 2 | 334.6 | 4 | 384.6 | 4 |  |  |
| 184.7 | 4 | 234.7 | 2 | 284.7 | 1 | 334.7 | 3 | 384.7 | 4 |  |  |
| 184.8 | 4 | 234.8 | 4 | 284.8 | 1 | 334.8 | 3 | 384.8 | 4 |  |  |
| 184.9 | 4 | 234.9 | 4 | 284.9 | 1 | 334.9 | 4 | 384.9 | 4 |  |  |
| 185 | 3 | 235 | 4 | 285 | 1 | 335 | 3 | 385 | 1 |  |  |
| 185.1 | 3 | 235.1 | 4 | 285.1 | 1 | 335.1 | 3 | 385.1 | 1 |  |  |
| 185.2 | 2 | 235.2 | 4 | 285.2 | 1 | 335.2 | 4 | 385.2 | 3 |  |  |
| 185.3 | 2 | 235.3 | 4 | 285.3 | 1 | 335.3 | 4 | 385.3 | 3 |  |  |
| 185.4 | 1 | 235.4 | 2 | 285.4 | 1 | 335.4 | 4 | 385.4 | 3 |  |  |
| 185.5 | 1 | 235.5 | 2 | 285.5 | 1 | 335.5 | 4 | 385.5 | 3 |  |  |
| 185.6 | 1 | 235.6 | 4 | 285.6 | 2 | 335.6 | 4 | 385.6 | 3 |  |  |
| 185.7 | 1 | 235.7 | 4 | 285.7 | 2 | 335.7 | 4 | 385.7 | 3 |  |  |
| 185.8 | 1 | 235.8 | 2 | 285.8 | 2 | 335.8 | 4 | 385.8 | 3 |  |  |
| 185.9 | 1 | 235.9 | 2 | 285.9 | 2 | 335.9 | 4 | 385.9 | 3 |  |  |
| 186 | 1 | 236 | 4 | 286 | 1 | 336 | 4 | 386 | 3 |  |  |
| 186.1 | 1 | 236.1 | 2 | 286.1 | 1 | 336.1 | 4 | 386.1 | 3 |  |  |
| 186.2 | 1 | 236.2 | 2 | 286.2 | 1 | 336.2 | 4 | 386.2 | 3 |  |  |
| 186.3 | 1 | 236.3 | 4 | 286.3 | 1 | 336.3 | 4 | 386.3 | 3 |  |  |
| 186.4 | 1 | 236.4 | 4 | 286.4 | 1 | 336.4 | 4 | 386.4 | 3 |  |  |
| 186.5 | 1 | 236.5 | 2 | 286.5 | 1 | 336.5 | 4 | 386.5 | 3 |  |  |
| 186.6 | 4 | 236.6 | 2 | 286.6 | 1 | 336.6 | 4 | 386.6 | 3 |  |  |
| 186.7 | 4 | 236.7 | 4 | 286.7 | 1 | 336.7 | 4 | 386.7 | 3 |  |  |
| 186.8 | 4 | 236.8 | 2 | 286.8 | 1 | 336.8 | 4 | 386.8 | 3 |  |  |
| 186.9 | 4 | 236.9 | 2 | 286.9 | 1 | 336.9 | 4 | 386.9 | 3 |  |  |
| 187 | 4 | 237 | 4 | 287 | 1 | 337 | 3 | 387 | 3 |  |  |
| 187.1 | 4 | 237.1 | 4 | 287.1 | 2 | 337.1 | 2 | 387.1 | 3 |  |  |
| 187.2 | 4 | 237.2 | 4 | 287.2 | 2 | 337.2 | 2 | 387.2 | 3 |  |  |
| 187.3 | 4 | 237.3 | 4 | 287.3 | 1 | 337.3 | 2 | 387.3 | 3 |  |  |
| 187.4 | 4 | 237.4 | 4 | 287.4 | 1 | 337.4 | 2 | 387.4 | 3 |  |  |
| 187.5 | 3 | 237.5 | 3 | 287.5 | 1 | 337.5 | 3 | 387.5 | 3 |  |  |
| 187.6 | 2 | 237.6 | 2 | 287.6 | 1 | 337.6 | 3 | 387.6 | 2 |  |  |
| 187.7 | 1 | 237.7 | 1 | 287.7 | 1 | 337.7 | 2 | 387.7 | 2 |  |  |
| 187.8 | 1 | 237.8 | 1 | 287.8 | 1 | 337.8 | 2 | 387.8 | 1 |  |  |
| 187.9 | 1 | 237.9 | 1 | 287.9 | 1 | 337.9 | 2 | 387.9 | 1 |  |  |
| 188 | 4 | 238 | 4 | 288 | 1 | 338 | 2 | 388 | 1 |  |  |
| 188.1 | 1 | 238.1 | 3 | 288.1 | 1 | 338.1 | 2 | 388.1 | 1 |  |  |
| 188.2 | 1 | 238.2 | 2 | 288.2 | 1 | 338.2 | 2 | 388.2 | 1 |  |  |
| 188.3 | 1 | 238.3 | 2 | 288.3 | 1 | 338.3 | 2 | 388.3 | 1 |  |  |
| 188.4 | 4 | 238.4 | 2 | 288.4 | 2 | 338.4 | 1 | 388.4 | 1 |  |  |
| 188.5 | 1 | 238.5 | 4 | 288.5 | 2 | 338.5 | 1 | 388.5 | 1 |  |  |
| 188.6 | 1 | 238.6 | 4 | 288.6 | 1 | 338.6 | 1 | 388.6 | 1 |  |  |
| 188.7 | 4 | 238.7 | 4 | 288.7 | 1 | 338.7 | 1 | 388.7 | 1 |  |  |
| 188.8 | 1 | 238.8 | 4 | 288.8 | 1 | 338.8 | 1 | 388.8 | 1 |  |  |
| 188.9 | 1 | 238.9 | 4 | 288.9 | 1 | 338.9 | 1 | 388.9 | 1 |  |  |
| 189 | 1 | 239 | 1 | 289 | 1 | 339 | 1 | 389 | 1 |  |  |
| 189.1 | 1 | 239.1 | 1 | 289.1 | 1 | 339.1 | 1 | 389.1 | 1 |  |  |
| 189.2 | 1 | 239.2 | 1 | 289.2 | 2 | 339.2 | 1 | 389.2 | 1 |  |  |
| 189.3 | 4 | 239.3 | 1 | 289.3 | 1 | 339.3 | 1 | 389.3 | 1 |  |  |
| 189.4 | 4 | 239.4 | 1 | 289.4 | 1 | 339.4 | 1 | 389.4 | 1 |  |  |
| 189.5 | 1 | 239.5 | 1 | 289.5 | 1 | 339.5 | 1 | 389.5 | 1 |  |  |
| 189.6 | 4 | 239.6 | 1 | 289.6 | 1 | 339.6 | 1 | 389.6 | 1 |  |  |
| 189.7 | 4 | 239.7 | 1 | 289.7 | 1 | 339.7 | 1 | 389.7 | 1 |  |  |
| 189.8 | 4 | 239.8 | 1 | 289.8 | 1 | 339.8 | 1 | 389.8 | 1 |  |  |
| 189.9 | 1 | 239.9 | 1 | 289.9 | 2 | 339.9 | 1 | 389.9 | 1 |  |  |
| 190 | 4 | 240 | 1 | 290 | 1 | 340 | 1 | 390 | 1 |  |  |
| 190.1 | 4 | 240.1 | 1 | 290.1 | 1 | 340.1 | 3 | 390.1 | 1 |  |  |
| 190.2 | 4 | 240.2 | 1 | 290.2 | 1 | 340.2 | 3 | 390.2 | 1 |  |  |
| 190.3 | 4 | 240.3 | 1 | 290.3 | 1 | 340.3 | 3 | 390.3 | 1 |  |  |
| 190.4 | 4 | 240.4 | 1 | 290.4 | 1 | 340.4 | 3 | 390.4 | 1 |  |  |
| 190.5 | 4 | 240.5 | 1 | 290.5 | 4 | 340.5 | 2 | 390.5 | 1 |  |  |
| 190.6 | 1 | 240.6 | 4 | 290.6 | 4 | 340.6 | 2 | 390.6 | 1 |  |  |
| 190.7 | 1 | 240.7 | 4 | 290.7 | 4 | 340.7 | 2 | 390.7 | 1 |  |  |
| 190.8 | 1 | 240.8 | 3 | 290.8 | 4 | 340.8 | 3 | 390.8 | 1 |  |  |
| 190.9 | 1 | 240.9 | 2 | 290.9 | 4 | 340.9 | 3 | 390.9 | 1 |  |  |
| 191 | 4 | 241 | 2 | 291 | 4 | 341 | 4 | 391 | 1 |  |  |
| 191.1 | 4 | 241.1 | 2 | 291.1 | 4 | 341.1 | 4 | 391.1 | 1 |  |  |
| 191.2 | 1 | 241.2 | 2 | 291.2 | 4 | 341.2 | 3 | 391.2 | 1 |  |  |
| 191.3 | 1 | 241.3 | 2 | 291.3 | 4 | 341.3 | 3 | 391.3 | 1 |  |  |
| 191.4 | 4 | 241.4 | 2 | 291.4 | 4 | 341.4 | 3 | 391.4 | 1 |  |  |
| 191.5 | 2 | 241.5 | 2 | 291.5 | 4 | 341.5 | 3 | 391.5 | 1 |  |  |
| 191.6 | 2 | 241.6 | 4 | 291.6 | 3 | 341.6 | 3 | 391.6 | 2 |  |  |
| 191.7 | 3 | 241.7 | 4 | 291.7 | 2 | 341.7 | 3 | 391.7 | 2 |  |  |
| 191.8 | 3 | 241.8 | 4 | 291.8 | 2 | 341.8 | 4 | 391.8 | 2 |  |  |
| 191.9 | 4 | 241.9 | 4 | 291.9 | 4 | 341.9 | 2 | 391.9 | 2 |  |  |
| 192 | 4 | 242 | 4 | 292 | 2 | 342 | 2 | 392 | 2 |  |  |
| 192.1 | 4 | 242.1 | 4 | 292.1 | 1 | 342.1 | 2 | 392.1 | 3 |  |  |
| 192.2 | 4 | 242.2 | 4 | 292.2 | 1 | 342.2 | 1 | 392.2 | 3 |  |  |
| 192.3 | 4 | 242.3 | 4 | 292.3 | 1 | 342.3 | 1 | 392.3 | 3 |  |  |
| 192.4 | 4 | 242.4 | 4 | 292.4 | 1 | 342.4 | 1 | 392.4 | 2 |  |  |
| 192.5 | 4 | 242.5 | 4 | 292.5 | 1 | 342.5 | 1 | 392.5 | 2 |  |  |
| 192.6 | 4 | 242.6 | 4 | 292.6 | 1 | 342.6 | 2 | 392.6 | 2 |  |  |
| 192.7 | 2 | 242.7 | 4 | 292.7 | 1 | 342.7 | 2 | 392.7 | 2 |  |  |
| 192.8 | 4 | 242.8 | 4 | 292.8 | 1 | 342.8 | 2 | 392.8 | 2 |  |  |
| 192.9 | 4 | 242.9 | 4 | 292.9 | 1 | 342.9 | 2 | 392.9 | 2 |  |  |
| 193 | 4 | 243 | 4 | 293 | 1 | 343 | 4 | 393 | 2 |  |  |
| 193.1 | 4 | 243.1 | 4 | 293.1 | 1 | 343.1 | 4 | 393.1 | 4 |  |  |
| 193.2 | 4 | 243.2 | 4 | 293.2 | 1 | 343.2 | 4 | 393.2 | 4 |  |  |
| 193.3 | 4 | 243.3 | 4 | 293.3 | 1 | 343.3 | 4 | 393.3 | 2 |  |  |
| 193.4 | 4 | 243.4 | 4 | 293.4 | 1 | 343.4 | 4 | 393.4 | 2 |  |  |
| 193.5 | 4 | 243.5 | 4 | 293.5 | 1 | 343.5 | 3 | 393.5 | 4 |  |  |
| 193.6 | 4 | 243.6 | 4 | 293.6 | 1 | 343.6 | 3 | 393.6 | 4 |  |  |
| 193.7 | 4 | 243.7 | 4 | 293.7 | 1 | 343.7 | 4 | 393.7 | 4 |  |  |
| 193.8 | 4 | 243.8 | 3 | 293.8 | 1 | 343.8 | 4 | 393.8 | 4 |  |  |
| 193.9 | 2 | 243.9 | 3 | 293.9 | 1 | 343.9 | 1 | 393.9 | 4 |  |  |
| 194 | 4 | 244 | 3 | 294 | 1 | 344 | 1 | 394 | 4 |  |  |
| 194.1 | 4 | 244.1 | 3 | 294.1 | 1 | 344.1 | 1 | 394.1 | 2 |  |  |
| 194.2 | 4 | 244.2 | 3 | 294.2 | 1 | 344.2 | 1 | 394.2 | 2 |  |  |
| 194.3 | 2 | 244.3 | 2 | 294.3 | 1 | 344.3 | 4 | 394.3 | 2 |  |  |
| 194.4 | 4 | 244.4 | 2 | 294.4 | 1 | 344.4 | 3 | 394.4 | 2 |  |  |
| 194.5 | 4 | 244.5 | 2 | 294.5 | 1 | 344.5 | 3 | 394.5 | 2 |  |  |
| 194.6 | 2 | 244.6 | 4 | 294.6 | 1 | 344.6 | 3 | 394.6 | 3 |  |  |
| 194.7 | 2 | 244.7 | 4 | 294.7 | 1 | 344.7 | 3 | 394.7 | 2 |  |  |
| 194.8 | 4 | 244.8 | 4 | 294.8 | 1 | 344.8 | 4 | 394.8 | 3 |  |  |
| 194.9 | 2 | 244.9 | 4 | 294.9 | 4 | 344.9 | 4 | 394.9 | 2 |  |  |
| 195 | 2 | 245 | 4 | 295 | 4 | 345 | 4 | 395 | 1 |  |  |
| 195.1 | 2 | 245.1 | 3 | 295.1 | 4 | 345.1 | 2 | 395.1 | 1 |  |  |
| 195.2 | 2 | 245.2 | 2 | 295.2 | 4 | 345.2 | 2 | 395.2 | 1 |  |  |
| 195.3 | 3 | 245.3 | 1 | 295.3 | 4 | 345.3 | 2 | 395.3 | 1 |  |  |
| 195.4 | 4 | 245.4 | 1 | 295.4 | 1 | 345.4 | 4 | 395.4 | 1 |  |  |
| 195.5 | 4 | 245.5 | 1 | 295.5 | 1 | 345.5 | 3 | 395.5 | 1 |  |  |
| 195.6 | 4 | 245.6 | 1 | 295.6 | 1 | 345.6 | 2 | 395.6 | 2 |  |  |
| 195.7 | 4 | 245.7 | 1 | 295.7 | 1 | 345.7 | 2 | 395.7 | 2 |  |  |
| 195.8 | 2 | 245.8 | 1 | 295.8 | 1 | 345.8 | 4 | 395.8 | 2 |  |  |
| 195.9 | 2 | 245.9 | 1 | 295.9 | 1 | 345.9 | 4 | 395.9 | 4 |  |  |
| 196 | 2 | 246 | 1 | 296 | 1 | 346 | 4 | 396 | 4 |  |  |
| 196.1 | 4 | 246.1 | 1 | 296.1 | 1 | 346.1 | 4 | 396.1 | 3 |  |  |
| 196.2 | 4 | 246.2 | 1 | 296.2 | 1 | 346.2 | 4 | 396.2 | 3 |  |  |
| 196.3 | 4 | 246.3 | 1 | 296.3 | 1 | 346.3 | 2 | 396.3 | 4 |  |  |
| 196.4 | 4 | 246.4 | 1 | 296.4 | 1 | 346.4 | 2 | 396.4 | 4 |  |  |
| 196.5 | 4 | 246.5 | 1 | 296.5 | 1 | 346.5 | 2 | 396.5 | 4 |  |  |
| 196.6 | 4 | 246.6 | 1 | 296.6 | 1 | 346.6 | 3 | 396.6 | 4 |  |  |
| 196.7 | 4 | 246.7 | 1 | 296.7 | 1 | 346.7 | 3 | 396.7 | 4 |  |  |
| 196.8 | 4 | 246.8 | 1 | 296.8 | 1 | 346.8 | 3 | 396.8 | 4 |  |  |
| 196.9 | 4 | 246.9 | 1 | 296.9 | 4 | 346.9 | 4 | 396.9 | 3 |  |  |
| 197 | 4 | 247 | 1 | 297 | 4 | 347 | 4 | 397 | 3 |  |  |
| 197.1 | 4 | 247.1 | 1 | 297.1 | 1 | 347.1 | 4 | 397.1 | 3 |  |  |
| 197.2 | 4 | 247.2 | 1 | 297.2 | 4 | 347.2 | 4 | 397.2 | 3 |  |  |
| 197.3 | 4 | 247.3 | 1 | 297.3 | 4 | 347.3 | 4 | 397.3 | 3 |  |  |
| 197.4 | 4 | 247.4 | 1 | 297.4 | 4 | 347.4 | 2 | 397.4 | 3 |  |  |
| 197.5 | 4 | 247.5 | 1 | 297.5 | 3 | 347.5 | 2 | 397.5 | 3 |  |  |
| 197.6 | 4 | 247.6 | 1 | 297.6 | 3 | 347.6 | 2 | 397.6 | 4 |  |  |
| 197.7 | 4 | 247.7 | 1 | 297.7 | 3 | 347.7 | 2 | 397.7 | 4 |  |  |
| 197.8 | 4 | 247.8 | 4 | 297.8 | 4 | 347.8 | 2 | 397.8 | 3 |  |  |
| 197.9 | 4 | 247.9 | 4 | 297.9 | 4 | 347.9 | 2 | 397.9 | 3 |  |  |
| 198 | 4 | 248 | 4 | 298 | 4 | 348 | 4 | 398 | 3 |  |  |
| 198.1 | 4 | 248.1 | 4 | 298.1 | 4 | 348.1 | 4 | 398.1 | 3 |  |  |
| 198.2 | 4 | 248.2 | 4 | 298.2 | 4 | 348.2 | 3 | 398.2 | 3 |  |  |
| 198.3 | 1 | 248.3 | 4 | 298.3 | 4 | 348.3 | 3 | 398.3 | 4 |  |  |
| 198.4 | 1 | 248.4 | 4 | 298.4 | 3 | 348.4 | 4 | 398.4 | 4 |  |  |
| 198.5 | 1 | 248.5 | 4 | 298.5 | 3 | 348.5 | 4 | 398.5 | 4 |  |  |
| 198.6 | 1 | 248.6 | 4 | 298.6 | 3 | 348.6 | 4 | 398.6 | 4 |  |  |
| 198.7 | 1 | 248.7 | 4 | 298.7 | 3 | 348.7 | 4 | 398.7 | 2 |  |  |
| 198.8 | 1 | 248.8 | 4 | 298.8 | 4 | 348.8 | 4 | 398.8 | 2 |  |  |
| 198.9 | 1 | 248.9 | 4 | 298.9 | 4 | 348.9 | 4 | 398.9 | 2 |  |  |
| 199 | 1 | 249 | 4 | 299 | 4 | 349 | 4 | 399 | 2 |  |  |
| 199.1 | 1 | 249.1 | 4 | 299.1 | 3 | 349.1 | 4 | 399.1 | 4 |  |  |
| 199.2 | 1 | 249.2 | 4 | 299.2 | 2 | 349.2 | 4 | 399.2 | 4 |  |  |
| 199.3 | 1 | 249.3 | 4 | 299.3 | 2 | 349.3 | 3 | 399.3 | 3 |  |  |
| 199.4 | 1 | 249.4 | 4 | 299.4 | 2 | 349.4 | 3 | 399.4 | 3 |  |  |
| 199.5 | 1 | 249.5 | 4 | 299.5 | 2 | 349.5 | 4 | 399.5 | 3 |  |  |
| 199.6 | 4 | 249.6 | 4 | 299.6 | 4 | 349.6 | 4 | 399.6 | 3 |  |  |
| 199.7 | 4 | 249.7 | 4 | 299.7 | 1 | 349.7 | 2 | 399.7 | 3 |  |  |
| 199.8 | 4 | 249.8 | 4 | 299.8 | 3 | 349.8 | 2 | 399.8 | 3 |  |  |
| 199.9 | 4 | 249.9 | 4 | 299.9 | 3 | 349.9 | 2 | 399.9 | 3 |  |  |

*1 = “Fine stone”, 2 = “Siltstone”, 3 = “Muddy Silstone”, and 4 = “Mudstone/Shales”

**Supplementary Table S2. Magnetic susceptibility**

| Depth (m) | MS | Depth (m) | MS | Depth (m) | MS |  | Depth (m) | MS | Depth (m) | MS |  | Depth (m) | MS |
| --- | --- | --- | --- | --- | --- | --- | --- | --- | --- | --- | --- | --- | --- |
| 150 | 0.132 | 200 | 0.027 | 250 | 0.378 |  | 300 | 0.148 | 350 | 0.174 |  | 400 | 0.216 |
| 150.02 | 0.14 | 200.02 | 0.032 | 250.02 | 0.359 |  | 300.02 | 0.14 | 350.02 | 0.185 |  | 400.02 | 0.211 |
| 150.04 | 0.138 | 200.04 | 0.032 | 250.04 | 0.352 |  | 300.04 | 0.131 | 350.04 | 0.195 |  | 400.04 | 0.193 |
| 150.06 | 0.129 | 200.06 | 0.021 | 250.06 | 0.353 |  | 300.06 | 0.129 | 350.06 | 0.201 |  | 400.06 | 0.178 |
| 150.08 | 0.118 | 200.08 | 0.074 | 250.08 | 0.327 |  | 300.08 | 0.123 | 350.08 | 0.203 |  | 400.08 | 0.187 |
| 150.1 | 0.107 | 200.1 | 0.151 | 250.1 | 0.302 |  | 300.1 | 0.109 | 350.1 | 0.208 |  | 400.1 | 0.197 |
| 150.12 | 0.105 | 200.12 | 0.205 | 250.12 | 0.342 |  | 300.12 | 0.128 | 350.12 | 0.219 |  | 400.12 | 0.224 |
| 150.14 | 0.086 | 200.14 | 0.228 | 250.14 | 0.2778 |  | 300.14 | 0.124 | 350.14 | 0.217 |  | 400.14 | 0.251 |
| 150.16 | 0.065 | 200.16 | 0.169 | 250.16 | 0.04 |  | 300.16 | 0.206 | 350.16 | 0.215 |  | 400.16 | 0.28 |
| 150.18 | 0.034 | 200.18 | 0.213 | 250.18 | -0.0205 |  | 300.18 | 0.192 | 350.18 | 0.221 |  | 400.18 | 0.284 |
| 150.2 | 0.002 | 200.2 | 0.179 | 250.2 | -0.0163 |  | 300.2 | 0.166 | 350.2 | 0.226 |  | 400.2 | 0.264 |
| 150.22 | -0.01 | 200.22 | 0.155 | 250.22 | -0.0135 |  | 300.22 | 0.101 | 350.22 | 0.213 |  | 400.22 | 0.238 |
| 150.24 | -0.019 | 200.24 | 0.144 | 250.24 | -0.0113 |  | 300.24 | 0.092 | 350.24 | 0.211 |  | 400.24 | 0.224 |
| 150.26 | -0.032 | 200.26 | 0.149 | 250.26 | -0.096 |  | 300.26 | 0.101 | 350.26 | 0.214 |  | 400.26 | 0.198 |
| 150.28 | -0.035 | 200.28 | 0.177 | 250.28 | -0.061 |  | 300.28 | 0.097 | 350.28 | 0.21 |  | 400.28 | 0.195 |
| 150.3 | -0.013 | 200.3 | 0.244 | 250.3 | -0.04 |  | 300.3 | 0.095 | 350.3 | 0.186 |  | 400.3 | 0.203 |
| 150.32 | -0.01 | 200.32 | 0.348 | 250.32 | 0.028 |  | 300.32 | 0.096 | 350.32 | 0.183 |  | 400.32 | 0.22 |
| 150.34 | -0.031 | 200.34 | 0.349 | 250.34 | 0.019 |  | 300.34 | 0.093 | 350.34 | 0.165 |  | 400.34 | 0.217 |
| 150.36 | -0.059 | 200.36 | 0.255 | 250.36 | -0.056 |  | 300.36 | 0.083 | 350.36 | 0.154 |  | 400.36 | 0.209 |
| 150.38 | -0.086 | 200.38 | 0.141 | 250.38 | -0.071 |  | 300.38 | 0.088 | 350.38 | 0.182 |  | 400.38 | 0.227 |
| 150.4 | -0.087 | 200.4 | 0.142 | 250.4 | -0.064 |  | 300.4 | 0.114 | 350.4 | 0.219 |  | 400.4 | 0.218 |
| 150.42 | -0.079 | 200.42 | 0.155 | 250.42 | -0.008 |  | 300.42 | 0.166 | 350.42 | 0.223 |  | 400.42 | 0.215 |
| 150.44 | -0.069 | 200.44 | 0.194 | 250.44 | 0.052 |  | 300.44 | 0.173 | 350.44 | 0.227 |  | 400.44 | 0.219 |
| 150.46 | -0.056 | 200.46 | 0.21 | 250.46 | 0.095 |  | 300.46 | 0.182 | 350.46 | 0.226 |  | 400.46 | 0.223 |
| 150.48 | -0.038 | 200.48 | 0.193 | 250.48 | 0.119 |  | 300.48 | 0.189 | 350.48 | 0.226 |  | 400.48 | 0.219 |
| 150.5 | 0.012 | 200.5 | 0.16 | 250.5 | 0.134 |  | 300.5 | 0.19 | 350.5 | 0.185 |  | 400.5 | 0.217 |
| 150.52 | 0.074 | 200.52 | 0.122 | 250.52 | 0.154 |  | 300.52 | 0.197 | 350.52 | 0.171 |  | 400.52 | 0.182 |
| 150.54 | 0.109 | 200.54 | 0.107 | 250.54 | 0.172 |  | 300.54 | 0.202 | 350.54 | 0.172 |  | 400.54 | 0.183 |
| 150.56 | 0.133 | 200.56 | 0.092 | 250.56 | 0.17 |  | 300.56 | 0.253 | 350.56 | 0.219 |  | 400.56 | 0.186 |
| 150.58 | 0.151 | 200.58 | 0.083 | 250.58 | 0.185 |  | 300.58 | 0.297 | 350.58 | 0.222 |  | 400.58 | 0.163 |
| 150.6 | 0.128 | 200.6 | 0.08 | 250.6 | 0.149 |  | 300.6 | 0.307 | 350.6 | 0.216 |  | 400.6 | 0.146 |
| 150.62 | 0.109 | 200.62 | 0.079 | 250.62 | 0.216 |  | 300.62 | 0.271 | 350.62 | 0.209 |  | 400.62 | 0.14 |
| 150.64 | 0.102 | 200.64 | 0.078 | 250.64 | 0.226 |  | 300.64 | 0.225 | 350.64 | 0.204 |  | 400.64 | 0.155 |
| 150.66 | 0.099 | 200.66 | 0.083 | 250.66 | 0.185 |  | 300.66 | 0.2 | 350.66 | 0.21 |  | 400.66 | 0.173 |
| 150.68 | 0.104 | 200.68 | 0.087 | 250.68 | 0.186 |  | 300.68 | 0.175 | 350.68 | 0.199 |  | 400.68 | 0.192 |
| 150.7 | 0.106 | 200.7 | 0.092 | 250.7 | 0.192 |  | 300.7 | 0.172 | 350.7 | 0.124 |  | 400.7 | 0.15 |
| 150.72 | 0.18 | 200.72 | 0.101 | 250.72 | 0.177 |  | 300.72 | 0.191 | 350.72 | 0.11 |  | 400.72 | 0.111 |
| 150.74 | 0.204 | 200.74 | 0.108 | 250.74 | 0.14 |  | 300.74 | 0.221 | 350.74 | 0.149 |  | 400.74 | 0.248 |
| 150.76 | 0.227 | 200.76 | 0.109 | 250.76 | 0.091 |  | 300.76 | 0.241 | 350.76 | 0.182 |  | 400.76 | 0.225 |
| 150.78 | 0.246 | 200.78 | 0.113 | 250.78 | 0.082 |  | 300.78 | 0.265 | 350.78 | 0.189 |  | 400.78 | 0.209 |
| 150.8 | 0.254 | 200.8 | 0.101 | 250.8 | 0.093 |  | 300.8 | 0.265 | 350.8 | 0.188 |  | 400.8 | 0.246 |
| 150.82 | 0.238 | 200.82 | 0.129 | 250.82 | 0.1 |  | 300.82 | 0.245 | 350.82 | 0.189 |  | 400.82 | 0.219 |
| 150.84 | 0.204 | 200.84 | 0.146 | 250.84 | 0.142 |  | 300.84 | 0.197 | 350.84 | 0.183 |  | 400.84 | 0.185 |
| 150.86 | 0.167 | 200.86 | 0.137 | 250.86 | 0.173 |  | 300.86 | 0.183 | 350.86 | 0.171 |  | 400.86 | 0.159 |
| 150.88 | 0.143 | 200.88 | 0.135 | 250.88 | 0.202 |  | 300.88 | 0.185 | 350.88 | 0.166 |  | 400.88 | 0.159 |
| 150.9 | 0.136 | 200.9 | 0.14 | 250.9 | 0.216 |  | 300.9 | 0.201 | 350.9 | 0.163 |  | 400.9 | 0.198 |
| 150.92 | 0.133 | 200.92 | 0.137 | 250.92 | 0.23 |  | 300.92 | 0.222 | 350.92 | 0.162 |  | 400.92 | 0.163 |
| 150.94 | 0.133 | 200.94 | 0.131 | 250.94 | 0.205 |  | 300.94 | 0.231 | 350.94 | 0.149 |  | 400.94 | 0.142 |
| 150.96 | 0.135 | 200.96 | 0.119 | 250.96 | 0.151 |  | 300.96 | 0.232 | 350.96 | 0.131 |  | 400.96 | 0.115 |
| 150.98 | 0.135 | 200.98 | 0.118 | 250.98 | 0.163 |  | 300.98 | 0.234 | 350.98 | 0.135 |  | 400.98 | 0.117 |
| 151 | 0.129 | 201 | 0.116 | 251 | 0.166 |  | 301 | 0.245 | 351 | 0.14 |  | 401 | 0.254 |
| 151.02 | 0.122 | 201.02 | 0.113 | 251.02 | 0.177 |  | 301.02 | 0.236 | 351.02 | 0.132 |  | 401.02 | 0.2629 |
| 151.04 | 0.12 | 201.04 | 0.111 | 251.04 | 0.17 |  | 301.04 | 0.164 | 351.04 | 0.141 |  | 401.04 | 0.268 |
| 151.06 | 0.133 | 201.06 | 0.106 | 251.06 | 0.162 |  | 301.06 | 0.164 | 351.06 | 0.139 |  | 401.06 | 0.282 |
| 151.08 | 0.128 | 201.08 | 0.106 | 251.08 | 0.164 |  | 301.08 | 0.163 | 351.08 | 0.134 |  | 401.08 | 0.211 |
| 151.1 | 0.121 | 201.1 | 0.106 | 251.1 | 0.172 |  | 301.1 | 0.166 | 351.1 | 0.147 |  | 401.1 | 0.282 |
| 151.12 | 0.12 | 201.12 | 0.111 | 251.12 | 0.17 |  | 301.12 | 0.16 | 351.12 | 0.174 |  | 401.12 | 0.232 |
| 151.14 | 0.12 | 201.14 | 0.135 | 251.14 | 0.161 |  | 301.14 | 0.148 | 351.14 | 0.184 |  | 401.14 | 0.223 |
| 151.16 | 0.119 | 201.16 | 0.184 | 251.16 | 0.158 |  | 301.16 | 0.165 | 351.16 | 0.17 |  | 401.16 | 0.284 |
| 151.18 | 0.12 | 201.18 | 0.012 | 251.18 | 0.161 |  | 301.18 | 0.164 | 351.18 | 0.164 |  | 401.18 | 0.213 |
| 151.2 | 0.126 | 201.2 | -0.008 | 251.2 | 0.158 |  | 301.2 | 0.173 | 351.2 | 0.16 |  | 401.2 | 0.414 |
| 151.22 | 0.135 | 201.22 | -0.006 | 251.22 | 0.117 |  | 301.22 | 0.18 | 351.22 | 0.172 |  | 401.22 | 0.3492 |
| 151.24 | 0.137 | 201.24 | 0.044 | 251.24 | 0.115 |  | 301.24 | 0.183 | 351.24 | 0.194 |  | 401.24 | 0.07 |
| 151.26 | 0.134 | 201.26 | 0.091 | 251.26 | 0.092 |  | 301.26 | 0.18 | 351.26 | 0.21 |  | 401.26 | 0.18 |
| 151.28 | 0.143 | 201.28 | 0.096 | 251.28 | 0.124 |  | 301.28 | 0.165 | 351.28 | 0.235 |  | 401.28 | 0.02 |
| 151.3 | 0.171 | 201.3 | 0.09 | 251.3 | 0.169 |  | 301.3 | 0.158 | 351.3 | 0.248 |  | 401.3 | 0.06 |
| 151.32 | 0.186 | 201.32 | 0.076 | 251.32 | 0.169 |  | 301.32 | 0.151 | 351.32 | 0.234 |  | 401.32 | 0.06 |
| 151.34 | 0.189 | 201.34 | 0.063 | 251.34 | 0.159 |  | 301.34 | 0.133 | 351.34 | 0.215 |  | 401.34 | 0.29 |
| 151.36 | 0.194 | 201.36 | 0.049 | 251.36 | 0.151 |  | 301.36 | 0.09 | 351.36 | 0.194 |  | 401.36 | 0.39 |
| 151.38 | 0.199 | 201.38 | 0.043 | 251.38 | 0.153 |  | 301.38 | 0.146 | 351.38 | 0.187 |  | 401.38 | 0.252 |
| 151.4 | 0.2 | 201.4 | 0.037 | 251.4 | 0.156 |  | 301.4 | 0.163 | 351.4 | 0.186 |  | 401.4 | 0.115 |
| 151.42 | 0.183 | 201.42 | 0.041 | 251.42 | 0.162 |  | 301.42 | 0.176 | 351.42 | 0.184 |  | 401.42 | 0.239 |
| 151.44 | 0.162 | 201.44 | 0.065 | 251.44 | 0.157 |  | 301.44 | 0.181 | 351.44 | 0.192 |  | 401.44 | 0.192 |
| 151.46 | 0.161 | 201.46 | 0.064 | 251.46 | 0.116 |  | 301.46 | 0.165 | 351.46 | 0.218 |  | 401.46 | 0.21 |
| 151.48 | 0.16 | 201.48 | 0.082 | 251.48 | 0.168 |  | 301.48 | 0.15 | 351.48 | 0.231 |  | 401.48 | 0.187 |
| 151.5 | 0.164 | 201.5 | 0.099 | 251.5 | 0.169 |  | 301.5 | 0.184 | 351.5 | 0.207 |  | 401.5 | 0.141 |
| 151.52 | 0.161 | 201.52 | 0.164 | 251.52 | 0.158 |  | 301.52 | 0.192 | 351.52 | 0.191 |  | 401.52 | 0.136 |
| 151.54 | 0.167 | 201.54 | 0.314 | 251.54 | 0.237 |  | 301.54 | 0.18 | 351.54 | 0.195 |  | 401.54 | 0.17 |
| 151.56 | 0.176 | 201.56 | 0.171 | 251.56 | 0.251 |  | 301.56 | 0.178 | 351.56 | 0.19 |  | 401.56 | 0.2 |
| 151.58 | 0.184 | 201.58 | 0.127 | 251.58 | 0.212 |  | 301.58 | 0.18 | 351.58 | 0.187 |  | 401.58 | 0.221 |
| 151.6 | 0.192 | 201.6 | 0.11 | 251.6 | 0.152 |  | 301.6 | 0.192 | 351.6 | 0.195 |  | 401.6 | 0.213 |
| 151.62 | 0.191 | 201.62 | 0.055 | 251.62 | 0.11 |  | 301.62 | 0.181 | 351.62 | 0.211 |  | 401.62 | 0.212 |
| 151.64 | 0.194 | 201.64 | 0.075 | 251.64 | 0.131 |  | 301.64 | 0.186 | 351.64 | 0.235 |  | 401.64 | 0.206 |
| 151.66 | 0.186 | 201.66 | 0.09 | 251.66 | 0.105 |  | 301.66 | 0.203 | 351.66 | 0.22 |  | 401.66 | 0.191 |
| 151.68 | 0.185 | 201.68 | 0.093 | 251.68 | 0.147 |  | 301.68 | 0.187 | 351.68 | 0.194 |  | 401.68 | 0.207 |
| 151.7 | 0.182 | 201.7 | 0.097 | 251.7 | 0.144 |  | 301.7 | 0.139 | 351.7 | 0.192 |  | 401.7 | 0.229 |
| 151.72 | 0.163 | 201.72 | 0.103 | 251.72 | 0.158 |  | 301.72 | 0.159 | 351.72 | 0.191 |  | 401.72 | 0.237 |
| 151.74 | 0.17 | 201.74 | 0.115 | 251.74 | 0.158 |  | 301.74 | 0.171 | 351.74 | 0.175 |  | 401.74 | 0.198 |
| 151.76 | 0.194 | 201.76 | 0.117 | 251.76 | 0.185 |  | 301.76 | 0.155 | 351.76 | 0.179 |  | 401.76 | 0.177 |
| 151.78 | 0.2 | 201.78 | 0.119 | 251.78 | 0.185 |  | 301.78 | 0.137 | 351.78 | 0.192 |  | 401.78 | 0.227 |
| 151.8 | 0.199 | 201.8 | 0.114 | 251.8 | 0.187 |  | 301.8 | 0.132 | 351.8 | 0.182 |  | 401.8 | 0.22 |
| 151.82 | 0.174 | 201.82 | 0.116 | 251.82 | 0.178 |  | 301.82 | 0.125 | 351.82 | 0.211 |  | 401.82 | 0.189 |
| 151.84 | 0.142 | 201.84 | 0.11 | 251.84 | 0.229 |  | 301.84 | 0.103 | 351.84 | 0.242 |  | 401.84 | 0.18 |
| 151.86 | 0.12 | 201.86 | 0.108 | 251.86 | 0.225 |  | 301.86 | 0.143 | 351.86 | 0.233 |  | 401.86 | 0.202 |
| 151.88 | 0.106 | 201.88 | 0.121 | 251.88 | 0.216 |  | 301.88 | 0.168 | 351.88 | 0.225 |  | 401.88 | 0.232 |
| 151.9 | 0.102 | 201.9 | 0.147 | 251.9 | 0.221 |  | 301.9 | 0.217 | 351.9 | 0.212 |  | 401.9 | 0.146 |
| 151.92 | 0.1 | 201.92 | 0.257 | 251.92 | 0.227 |  | 301.92 | 0.218 | 351.92 | 0.203 |  | 401.92 | 0.125 |
| 151.94 | 0.125 | 201.94 | 0.151 | 251.94 | 0.237 |  | 301.94 | 0.215 | 351.94 | 0.212 |  | 401.94 | 0.172 |
| 151.96 | 0.155 | 201.96 | 0.129 | 251.96 | 0.244 |  | 301.96 | 0.215 | 351.96 | 0.215 |  | 401.96 | 0.173 |
| 151.98 | 0.186 | 201.98 | 0.135 | 251.98 | 0.24 |  | 301.98 | 0.203 | 351.98 | 0.212 |  | 401.98 | 0.18 |
| 152 | 0.209 | 202 | 0.122 | 252 | 0.228 |  | 302 | 0.185 | 352 | 0.21 |  | 402 | 0.164 |
| 152.02 | 0.212 | 202.02 | 0.113 | 252.02 | 0.209 |  | 302.02 | 0.175 | 352.02 | 0.216 |  | 402.02 | 0.203 |
| 152.04 | 0.197 | 202.04 | 0.105 | 252.04 | 0.196 |  | 302.04 | 0.188 | 352.04 | 0.221 |  | 402.04 | 0.201 |
| 152.06 | 0.176 | 202.06 | 0.1 | 252.06 | 0.123 |  | 302.06 | 0.188 | 352.06 | 0.236 |  | 402.06 | 0.191 |
| 152.08 | 0.151 | 202.08 | 0.098 | 252.08 | 0.147 |  | 302.08 | 0.186 | 352.08 | 0.234 |  | 402.08 | 0.169 |
| 152.1 | 0.138 | 202.1 | 0.098 | 252.1 | 0.17 |  | 302.1 | 0.178 | 352.1 | 0.222 |  | 402.1 | 0.19 |
| 152.12 | 0.142 | 202.12 | 0.102 | 252.12 | 0.181 |  | 302.12 | 0.179 | 352.12 | 0.202 |  | 402.12 | 0.193 |
| 152.14 | 0.153 | 202.14 | 0.118 | 252.14 | 0.158 |  | 302.14 | 0.185 | 352.14 | 0.196 |  | 402.14 | 0.197 |
| 152.16 | 0.167 | 202.16 | 0.137 | 252.16 | 0.122 |  | 302.16 | 0.192 | 352.16 | 0.179 |  | 402.16 | 0.162 |
| 152.18 | 0.177 | 202.18 | 0.139 | 252.18 | 0.106 |  | 302.18 | 0.171 | 352.18 | 0.188 |  | 402.18 | 0.164 |
| 152.2 | 0.188 | 202.2 | 0.156 | 252.2 | 0.125 |  | 302.2 | 0.171 | 352.2 | 0.207 |  | 402.2 | 0.167 |
| 152.22 | 0.178 | 202.22 | 0.185 | 252.22 | 0.172 |  | 302.22 | 0.253 | 352.22 | 0.215 |  | 402.22 | 0.166 |
| 152.24 | 0.169 | 202.24 | 0.188 | 252.24 | 0.138 |  | 302.24 | 0.157 | 352.24 | 0.18 |  | 402.24 | 0.157 |
| 152.26 | 0.185 | 202.26 | 0.22 | 252.26 | 0.12 |  | 302.26 | 0.156 | 352.26 | 0.183 |  | 402.26 | 0.157 |
| 152.28 | 0.191 | 202.28 | 0.231 | 252.28 | 0.166 |  | 302.28 | 0.3732 | 352.28 | 0.199 |  | 402.28 | 0.094 |
| 152.3 | 0.193 | 202.3 | 0.182 | 252.3 | 0.198 |  | 302.3 | 0.365 | 352.3 | 0.2 |  | 402.3 | 0.058 |
| 152.32 | 0.192 | 202.32 | 0.165 | 252.32 | 0.231 |  | 302.32 | 0.241 | 352.32 | 0.204 |  | 402.32 | 0.043 |
| 152.34 | 0.189 | 202.34 | 0.149 | 252.34 | 0.346 |  | 302.34 | 0.179 | 352.34 | 0.201 |  | 402.34 | 0.109 |
| 152.36 | 0.182 | 202.36 | 0.122 | 252.36 | 0.252 |  | 302.36 | 0.146 | 352.36 | 0.202 |  | 402.36 | 0.087 |
| 152.38 | 0.171 | 202.38 | 0.144 | 252.38 | 0.233 |  | 302.38 | 0.161 | 352.38 | 0.199 |  | 402.38 | 0.081 |
| 152.4 | 0.169 | 202.4 | 0.07 | 252.4 | 0.215 |  | 302.4 | 0.195 | 352.4 | 0.198 |  | 402.4 | 0.078 |
| 152.42 | 0.165 | 202.42 | 0.137 | 252.42 | 0.168 |  | 302.42 | 0.196 | 352.42 | 0.192 |  | 402.42 | 0.154 |
| 152.44 | 0.179 | 202.44 | 0.324 | 252.44 | 0.116 |  | 302.44 | 0.193 | 352.44 | 0.144 |  | 402.44 | 0.165 |
| 152.46 | 0.188 | 202.46 | 0.302 | 252.46 | 0.265 |  | 302.46 | 0.182 | 352.46 | 0.161 |  | 402.46 | 0.081 |
| 152.48 | 0.191 | 202.48 | 0.293 | 252.48 | 0.282 |  | 302.48 | 0.17 | 352.48 | 0.17 |  | 402.48 | 0.046 |
| 152.5 | 0.185 | 202.5 | 0.183 | 252.5 | 0.26 |  | 302.5 | 0.173 | 352.5 | 0.16 |  | 402.5 | 0.138 |
| 152.52 | 0.185 | 202.52 | 0.144 | 252.52 | 0.229 |  | 302.52 | 0.182 | 352.52 | 0.186 |  | 402.52 | 0.136 |
| 152.54 | 0.181 | 202.54 | 0.132 | 252.54 | 0.185 |  | 302.54 | 0.189 | 352.54 | 0.212 |  | 402.54 | 0.138 |
| 152.56 | 0.168 | 202.56 | 0.14 | 252.56 | 0.162 |  | 302.56 | 0.186 | 352.56 | 0.192 |  | 402.56 | 0.138 |
| 152.58 | 0.107 | 202.58 | 0.153 | 252.58 | 0.178 |  | 302.58 | 0.175 | 352.58 | 0.182 |  | 402.58 | 0.179 |
| 152.6 | 0.135 | 202.6 | 0.171 | 252.6 | 0.195 |  | 302.6 | 0.162 | 352.6 | 0.179 |  | 402.6 | 0.184 |
| 152.62 | 0.18 | 202.62 | 0.181 | 252.62 | 0.204 |  | 302.62 | 0.184 | 352.62 | 0.196 |  | 402.62 | 0.17 |
| 152.64 | 0.184 | 202.64 | 0.169 | 252.64 | 0.207 |  | 302.64 | 0.197 | 352.64 | 0.236 |  | 402.64 | 0.16 |
| 152.66 | 0.191 | 202.66 | 0.148 | 252.66 | 0.211 |  | 302.66 | 0.207 | 352.66 | 0.217 |  | 402.66 | 0.16 |
| 152.68 | 0.185 | 202.68 | 0.12 | 252.68 | 0.234 |  | 302.68 | 0.203 | 352.68 | 0.23 |  | 402.68 | 0.175 |
| 152.7 | 0.159 | 202.7 | 0.121 | 252.7 | 0.286 |  | 302.7 | 0.195 | 352.7 | 0.233 |  | 402.7 | 0.165 |
| 152.72 | 0.097 | 202.72 | 0.116 | 252.72 | 0.302 |  | 302.72 | 0.193 | 352.72 | 0.225 |  | 402.72 | 0.159 |
| 152.74 | 0.074 | 202.74 | 0.117 | 252.74 | 0.262 |  | 302.74 | 0.179 | 352.74 | 0.208 |  | 402.74 | 0.184 |
| 152.76 | 0.089 | 202.76 | 0.119 | 252.76 | 0.211 |  | 302.76 | 0.171 | 352.76 | 0.2 |  | 402.76 | 0.181 |
| 152.78 | 0.074 | 202.78 | 0.112 | 252.78 | 0.16 |  | 302.78 | 0.191 | 352.78 | 0.181 |  | 402.78 | 0.143 |
| 152.8 | 0.079 | 202.8 | 0.091 | 252.8 | 0.132 |  | 302.8 | 0.198 | 352.8 | 0.2 |  | 402.8 | 0.157 |
| 152.82 | 0.114 | 202.82 | 0.093 | 252.82 | 0.14 |  | 302.82 | 0.205 | 352.82 | 0.213 |  | 402.82 | 0.177 |
| 152.84 | 0.151 | 202.84 | 0.119 | 252.84 | 0.154 |  | 302.84 | 0.202 | 352.84 | 0.225 |  | 402.84 | 0.185 |
| 152.86 | 0.18 | 202.86 | 0.175 | 252.86 | 0.164 |  | 302.86 | 0.215 | 352.86 | 0.204 |  | 402.86 | 0.187 |
| 152.88 | 0.233 | 202.88 | 0.199 | 252.88 | 0.169 |  | 302.88 | 0.223 | 352.88 | 0.194 |  | 402.88 | 0.219 |
| 152.9 | 0.236 | 202.9 | 0.158 | 252.9 | 0.168 |  | 302.9 | 0.214 | 352.9 | 0.201 |  | 402.9 | 0.229 |
| 152.92 | 0.088 | 202.92 | 0.132 | 252.92 | 0.164 |  | 302.92 | 0.209 | 352.92 | 0.207 |  | 402.92 | 0.206 |
| 152.94 | 0.11 | 202.94 | 0.134 | 252.94 | 0.175 |  | 302.94 | 0.189 | 352.94 | 0.207 |  | 402.94 | 0.242 |
| 152.96 | 0.145 | 202.96 | 0.153 | 252.96 | 0.213 |  | 302.96 | 0.17 | 352.96 | 0.204 |  | 402.96 | 0.228 |
| 152.98 | 0.162 | 202.98 | 0.174 | 252.98 | 0.223 |  | 302.98 | 0.197 | 352.98 | 0.204 |  | 402.98 | 0.207 |
| 153 | 0.161 | 203 | 0.187 | 253 | 0.184 |  | 303 | 0.175 | 353 | 0.212 |  | 403 | 0.19 |
| 153.02 | 0.134 | 203.02 | 0.189 | 253.02 | 0.131 |  | 303.02 | 0.158 | 353.02 | 0.193 |  | 403.02 | 0.185 |
| 153.04 | 0.135 | 203.04 | 0.168 | 253.04 | 0.11 |  | 303.04 | 0.18 | 353.04 | 0.201 |  | 403.04 | 0.18 |
| 153.06 | 0.143 | 203.06 | 0.159 | 253.06 | 0.088 |  | 303.06 | 0.177 | 353.06 | 0.199 |  | 403.06 | 0.178 |
| 153.08 | 0.154 | 203.08 | 0.015 | 253.08 | 0.073 |  | 303.08 | 0.155 | 353.08 | 0.154 |  | 403.08 | 0.181 |
| 153.1 | 0.163 | 203.1 | 0.02 | 253.1 | 0.068 |  | 303.1 | 0.156 | 353.1 | 0.149 |  | 403.1 | 0.179 |
| 153.12 | 0.14 | 203.12 | 0.019 | 253.12 | 0.057 |  | 303.12 | 0.125 | 353.12 | 0.169 |  | 403.12 | 0.165 |
| 153.14 | 0.127 | 203.14 | -0.024 | 253.14 | 0.038 |  | 303.14 | 0.088 | 353.14 | 0.171 |  | 403.14 | 0.182 |
| 153.16 | 0.118 | 203.16 | 0 | 253.16 | 0.083 |  | 303.16 | 0.092 | 353.16 | 0.166 |  | 403.16 | 0.185 |
| 153.18 | 0.123 | 203.18 | 0.017 | 253.18 | 0.11 |  | 303.18 | 0.099 | 353.18 | 0.19 |  | 403.18 | 0.188 |
| 153.2 | 0.122 | 203.2 | 0.023 | 253.2 | 0.12 |  | 303.2 | 0.173 | 353.2 | 0.211 |  | 403.2 | 0.187 |
| 153.22 | 0.123 | 203.22 | 0.053 | 253.22 | 0.121 |  | 303.22 | 0.162 | 353.22 | 0.189 |  | 403.22 | 0.169 |
| 153.24 | 0.129 | 203.24 | 0.141 | 253.24 | 0.13 |  | 303.24 | 0.14 | 353.24 | 0.18 |  | 403.24 | 0.177 |
| 153.26 | 0.123 | 203.26 | 0.137 | 253.26 | 0.055 |  | 303.26 | 0.156 | 353.26 | 0.164 |  | 403.26 | 0.226 |
| 153.28 | 0.12 | 203.28 | 0.098 | 253.28 | 0.068 |  | 303.28 | 0.173 | 353.28 | 0.171 |  | 403.28 | 0.271 |
| 153.3 | 0.109 | 203.3 | 0.087 | 253.3 | 0.203 |  | 303.3 | 0.14 | 353.3 | 0.2 |  | 403.3 | 0.33 |
| 153.32 | 0.105 | 203.32 | 0.091 | 253.32 | 0.254 |  | 303.32 | 0.088 | 353.32 | 0.224 |  | 403.32 | 0.3438 |
| 153.34 | 0.101 | 203.34 | 0.086 | 253.34 | 0.241 |  | 303.34 | 0.069 | 353.34 | 0.226 |  | 403.34 | 0.3342 |
| 153.36 | 0.098 | 203.36 | 0.093 | 253.36 | 0.171 |  | 303.36 | 0.146 | 353.36 | 0.222 |  | 403.36 | 0.251 |
| 153.38 | 0.092 | 203.38 | 0.092 | 253.38 | 0.143 |  | 303.38 | 0.147 | 353.38 | 0.219 |  | 403.38 | 0.3 |
| 153.4 | 0.079 | 203.4 | 0.1 | 253.4 | 0.114 |  | 303.4 | 0.174 | 353.4 | 0.205 |  | 403.4 | 0.323 |
| 153.42 | 0.084 | 203.42 | 0.093 | 253.42 | 0.108 |  | 303.42 | 0.206 | 353.42 | 0.194 |  | 403.42 | 0.301 |
| 153.44 | 0.086 | 203.44 | 0.099 | 253.44 | 0.1 |  | 303.44 | 0.204 | 353.44 | 0.166 |  | 403.44 | 0.258 |
| 153.46 | 0.086 | 203.46 | 0.113 | 253.46 | 0.109 |  | 303.46 | 0.207 | 353.46 | 0.185 |  | 403.46 | 0.232 |
| 153.48 | 0.088 | 203.48 | 0.112 | 253.48 | 0.122 |  | 303.48 | 0.21 | 353.48 | 0.2 |  | 403.48 | 0.215 |
| 153.5 | 0.092 | 203.5 | 0.138 | 253.5 | 0.128 |  | 303.5 | 0.21 | 353.5 | 0.199 |  | 403.5 | 0.198 |
| 153.52 | 0.019 | 203.52 | 0.132 | 253.52 | 0.115 |  | 303.52 | 0.199 | 353.52 | 0.211 |  | 403.52 | 0.188 |
| 153.54 | 0.027 | 203.54 | 0.1 | 253.54 | 0.117 |  | 303.54 | 0.195 | 353.54 | 0.216 |  | 403.54 | 0.176 |
| 153.56 | 0.038 | 203.56 | 0.102 | 253.56 | 0.133 |  | 303.56 | 0.201 | 353.56 | 0.228 |  | 403.56 | 0.164 |
| 153.58 | 0.051 | 203.58 | 0.1 | 253.58 | 0.139 |  | 303.58 | 0.186 | 353.58 | 0.216 |  | 403.58 | 0.153 |
| 153.6 | 0.058 | 203.6 | 0.12 | 253.6 | 0.141 |  | 303.6 | 0.176 | 353.6 | 0.209 |  | 403.6 | 0.131 |
| 153.62 | 0.065 | 203.62 | 0.147 | 253.62 | 0.138 |  | 303.62 | 0.166 | 353.62 | 0.21 |  | 403.62 | 0.126 |
| 153.64 | 0.07 | 203.64 | 0.149 | 253.64 | 0.138 |  | 303.64 | 0.18 | 353.64 | 0.228 |  | 403.64 | 0.146 |
| 153.66 | 0.072 | 203.66 | 0.125 | 253.66 | 0.018 |  | 303.66 | 0.18 | 353.66 | 0.219 |  | 403.66 | 0.151 |
| 153.68 | 0.069 | 203.68 | 0.095 | 253.68 | 0.03 |  | 303.68 | 0.179 | 353.68 | 0.23 |  | 403.68 | 0.174 |
| 153.7 | -0.036 | 203.7 | 0.109 | 253.7 | 0.038 |  | 303.7 | 0.15 | 353.7 | 0.247 |  | 403.7 | 0.188 |
| 153.72 | -0.018 | 203.72 | 0.156 | 253.72 | 0.04 |  | 303.72 | 0.132 | 353.72 | 0.235 |  | 403.72 | 0.184 |
| 153.74 | -0.015 | 203.74 | 0.202 | 253.74 | 0.046 |  | 303.74 | 0.13 | 353.74 | 0.225 |  | 403.74 | 0.178 |
| 153.76 | -0.01 | 203.76 | 0.207 | 253.76 | 0.052 |  | 303.76 | 0.153 | 353.76 | 0.195 |  | 403.76 | 0.165 |
| 153.78 | 0 | 203.78 | 0.138 | 253.78 | 0.06 |  | 303.78 | 0.18 | 353.78 | 0.135 |  | 403.78 | 0.208 |
| 153.8 | 0.022 | 203.8 | 0.087 | 253.8 | 0.057 |  | 303.8 | 0.174 | 353.8 | 0.102 |  | 403.8 | 0.212 |
| 153.82 | 0.034 | 203.82 | 0.096 | 253.82 | 0.062 |  | 303.82 | 0.209 | 353.82 | 0.136 |  | 403.82 | 0.212 |
| 153.84 | 0.043 | 203.84 | 0.094 | 253.84 | 0.06 |  | 303.84 | 0.184 | 353.84 | 0.152 |  | 403.84 | 0.211 |
| 153.86 | 0.053 | 203.86 | 0.089 | 253.86 | 0.082 |  | 303.86 | 0.17 | 353.86 | 0.155 |  | 403.86 | 0.203 |
| 153.88 | 0.056 | 203.88 | 0.082 | 253.88 | 0.115 |  | 303.88 | 0.125 | 353.88 | 0.138 |  | 403.88 | 0.215 |
| 153.9 | 0.062 | 203.9 | 0.064 | 253.9 | 0.136 |  | 303.9 | 0.09 | 353.9 | 0.089 |  | 403.9 | 0.217 |
| 153.92 | 0.057 | 203.92 | 0.236 | 253.92 | 0.151 |  | 303.92 | 0.099 | 353.92 | 0.127 |  | 403.92 | 0.204 |
| 153.94 | 0.034 | 203.94 | 0.292 | 253.94 | 0.2 |  | 303.94 | 0.216 | 353.94 | 0.21 |  | 403.94 | 0.201 |
| 153.96 | 0.015 | 203.96 | 0.162 | 253.96 | 0.262 |  | 303.96 | 0.221 | 353.96 | 0.212 |  | 403.96 | 0.201 |
| 153.98 | 0.036 | 203.98 | 0.162 | 253.98 | 0.317 |  | 303.98 | 0.226 | 353.98 | 0.247 |  | 403.98 | 0.221 |
| 154 | 0.06 | 204 | 0.208 | 254 | 0.357 |  | 304 | 0.229 | 354 | 0.251 |  | 404 | 0.196 |
| 154.02 | 0.074 | 204.02 | 0.147 | 254.02 | 0.38 |  | 304.02 | 0.228 | 354.02 | 0.251 |  | 404.02 | 0.168 |
| 154.04 | 0.089 | 204.04 | 0.118 | 254.04 | 0.386 |  | 304.04 | 0.223 | 354.04 | 0.243 |  | 404.04 | 0.163 |
| 154.06 | 0.098 | 204.06 | 0.117 | 254.06 | 0.242 |  | 304.06 | 0.224 | 354.06 | 0.234 |  | 404.06 | 0.168 |
| 154.08 | 0.117 | 204.08 | 0.167 | 254.08 | 0.212 |  | 304.08 | 0.228 | 354.08 | 0.221 |  | 404.08 | 0.212 |
| 154.1 | 0.126 | 204.1 | 0.162 | 254.1 | 0.161 |  | 304.1 | 0.228 | 354.1 | 0.233 |  | 404.1 | 0.201 |
| 154.12 | 0.121 | 204.12 | 0.084 | 254.12 | 0.131 |  | 304.12 | 0.227 | 354.12 | 0.234 |  | 404.12 | 0.178 |
| 154.14 | 0.108 | 204.14 | 0.051 | 254.14 | 0.109 |  | 304.14 | 0.221 | 354.14 | 0.245 |  | 404.14 | 0.157 |
| 154.16 | 0.106 | 204.16 | 0.046 | 254.16 | 0.088 |  | 304.16 | 0.221 | 354.16 | 0.227 |  | 404.16 | 0.165 |
| 154.18 | 0.105 | 204.18 | 0.062 | 254.18 | 0.035 |  | 304.18 | 0.223 | 354.18 | 0.205 |  | 404.18 | 0.176 |
| 154.2 | 0.108 | 204.2 | 0.086 | 254.2 | -0.024 |  | 304.2 | 0.229 | 354.2 | 0.174 |  | 404.2 | 0.18 |
| 154.22 | 0.113 | 204.22 | 0.142 | 254.22 | -0.004 |  | 304.22 | 0.198 | 354.22 | 0.172 |  | 404.22 | 0.176 |
| 154.24 | 0.108 | 204.24 | 0.181 | 254.24 | 0.003 |  | 304.24 | 0.197 | 354.24 | 0.143 |  | 404.24 | 0.161 |
| 154.26 | 0.1 | 204.26 | 0.181 | 254.26 | 0.017 |  | 304.26 | 0.2 | 354.26 | 0.205 |  | 404.26 | 0.136 |
| 154.28 | 0.098 | 204.28 | 0.018 | 254.28 | 0.028 |  | 304.28 | 0.172 | 354.28 | 0.227 |  | 404.28 | 0.129 |
| 154.3 | 0.088 | 204.3 | -0.043 | 254.3 | 0.045 |  | 304.3 | 0.147 | 354.3 | 0.221 |  | 404.3 | 0.162 |
| 154.32 | 0.084 | 204.32 | -0.09 | 254.32 | 0.056 |  | 304.32 | 0.138 | 354.32 | 0.204 |  | 404.32 | 0.193 |
| 154.34 | 0.089 | 204.34 | -0.091 | 254.34 | 0.069 |  | 304.34 | 0.153 | 354.34 | 0.193 |  | 404.34 | 0.174 |
| 154.36 | 0.085 | 204.36 | -0.091 | 254.36 | 0.081 |  | 304.36 | 0.178 | 354.36 | 0.183 |  | 404.36 | 0.173 |
| 154.38 | 0.078 | 204.38 | -0.071 | 254.38 | 0.078 |  | 304.38 | 0.19 | 354.38 | 0.208 |  | 404.38 | 0.196 |
| 154.4 | 0.045 | 204.4 | -0.033 | 254.4 | 0.086 |  | 304.4 | 0.188 | 354.4 | 0.198 |  | 404.4 | 0.205 |
| 154.42 | 0.067 | 204.42 | 0.01 | 254.42 | 0.043 |  | 304.42 | 0.181 | 354.42 | 0.191 |  | 404.42 | 0.193 |
| 154.44 | 0.066 | 204.44 | -0.041 | 254.44 | 0.037 |  | 304.44 | 0.156 | 354.44 | 0.213 |  | 404.44 | 0.183 |
| 154.46 | 0.07 | 204.46 | -0.036 | 254.46 | 0.003 |  | 304.46 | 0.112 | 354.46 | 0.214 |  | 404.46 | 0.172 |
| 154.48 | 0.073 | 204.48 | -0.008 | 254.48 | 0.024 |  | 304.48 | 0.104 | 354.48 | 0.191 |  | 404.48 | 0.173 |
| 154.5 | 0.075 | 204.5 | 0.03 | 254.5 | 0.053 |  | 304.5 | 0.124 | 354.5 | 0.182 |  | 404.5 | 0.199 |
| 154.52 | 0.076 | 204.52 | 0.05 | 254.52 | 0.08 |  | 304.52 | 0.169 | 354.52 | 0.186 |  | 404.52 | 0.207 |
| 154.54 | 0.077 | 204.54 | 0.074 | 254.54 | 0.109 |  | 304.54 | 0.175 | 354.54 | 0.22 |  | 404.54 | 0.187 |
| 154.56 | 0.078 | 204.56 | 0.078 | 254.56 | 0.141 |  | 304.56 | 0.187 | 354.56 | 0.204 |  | 404.56 | 0.212 |
| 154.58 | 0.084 | 204.58 | 0.069 | 254.58 | 0.162 |  | 304.58 | 0.193 | 354.58 | 0.163 |  | 404.58 | 0.186 |
| 154.6 | 0.095 | 204.6 | 0.101 | 254.6 | 0.146 |  | 304.6 | 0.2 | 354.6 | 0.152 |  | 404.6 | 0.195 |
| 154.62 | 0.089 | 204.62 | 0.111 | 254.62 | 0.155 |  | 304.62 | 0.188 | 354.62 | 0.159 |  | 404.62 | 0.221 |
| 154.64 | 0.015 | 204.64 | 0.096 | 254.64 | 0.167 |  | 304.64 | 0.168 | 354.64 | 0.167 |  | 404.64 | 0.193 |
| 154.66 | 0.023 | 204.66 | 0.112 | 254.66 | 0.164 |  | 304.66 | 0.188 | 354.66 | 0.196 |  | 404.66 | 0.159 |
| 154.68 | 0.018 | 204.68 | 0.086 | 254.68 | 0.118 |  | 304.68 | 0.224 | 354.68 | 0.144 |  | 404.68 | 0.146 |
| 154.7 | 0.006 | 204.7 | 0.092 | 254.7 | 0.082 |  | 304.7 | 0.214 | 354.7 | 0.233 |  | 404.7 | 0.127 |
| 154.72 | 0.017 | 204.72 | 0.076 | 254.72 | 0.071 |  | 304.72 | 0.172 | 354.72 | 0.24 |  | 404.72 | 0.097 |
| 154.74 | 0.026 | 204.74 | 0.066 | 254.74 | 0.1 |  | 304.74 | 0.167 | 354.74 | 0.219 |  | 404.74 | 0.099 |
| 154.76 | 0.029 | 204.76 | 0.059 | 254.76 | 0.112 |  | 304.76 | 0.152 | 354.76 | 0.2 |  | 404.76 | 0.102 |
| 154.78 | 0.015 | 204.78 | 0.056 | 254.78 | 0.105 |  | 304.78 | 0.142 | 354.78 | 0.21 |  | 404.78 | 0.129 |
| 154.8 | 0.008 | 204.8 | 0.039 | 254.8 | 0.114 |  | 304.8 | 0.154 | 354.8 | 0.238 |  | 404.8 | 0.137 |
| 154.82 | 0.025 | 204.82 | 0.051 | 254.82 | 0.138 |  | 304.82 | 0.146 | 354.82 | 0.247 |  | 404.82 | 0.147 |
| 154.84 | 0.026 | 204.84 | 0.051 | 254.84 | 0.133 |  | 304.84 | 0.146 | 354.84 | 0.245 |  | 404.84 | 0.163 |
| 154.86 | 0.028 | 204.86 | 0.058 | 254.86 | 0.001 |  | 304.86 | 0.148 | 354.86 | 0.245 |  | 404.86 | 0.201 |
| 154.88 | 0.018 | 204.88 | 0.073 | 254.88 | 0.03 |  | 304.88 | 0.164 | 354.88 | 0.249 |  | 404.88 | 0.218 |
| 154.9 | 0.008 | 204.9 | 0.125 | 254.9 | 0.052 |  | 304.9 | 0.152 | 354.9 | 0.243 |  | 404.9 | 0.216 |
| 154.92 | 0 | 204.92 | 0.162 | 254.92 | 0.052 |  | 304.92 | 0.152 | 354.92 | 0.211 |  | 404.92 | 0.198 |
| 154.94 | 0.005 | 204.94 | 0.228 | 254.94 | 0.047 |  | 304.94 | 0.159 | 354.94 | 0.177 |  | 404.94 | 0.196 |
| 154.96 | 0 | 204.96 | 0.27 | 254.96 | 0.029 |  | 304.96 | 0.177 | 354.96 | 0.187 |  | 404.96 | 0.214 |
| 154.98 | -0.002 | 204.98 | 0.298 | 254.98 | 0.089 |  | 304.98 | 0.197 | 354.98 | 0.24 |  | 404.98 | 0.225 |
| 155 | 0.001 | 205 | 0.272 | 255 | 0.09 |  | 305 | 0.202 | 355 | 0.241 |  | 405 | 0.223 |
| 155.02 | 0.011 | 205.02 | 0.154 | 255.02 | 0.104 |  | 305.02 | 0.207 | 355.02 | 0.237 |  | 405.02 | 0.215 |
| 155.04 | 0.012 | 205.04 | 0.102 | 255.04 | 0.131 |  | 305.04 | 0.217 | 355.04 | 0.238 |  | 405.04 | 0.206 |
| 155.06 | 0.017 | 205.06 | 0.1 | 255.06 | 0.148 |  | 305.06 | 0.226 | 355.06 | 0.134 |  | 405.06 | 0.214 |
| 155.08 | 0.006 | 205.08 | 0.007 | 255.08 | 0.144 |  | 305.08 | 0.222 | 355.08 | 0.176 |  |  |  |
| 155.1 | 0.005 | 205.1 | 0 | 255.1 | 0.135 |  | 305.1 | 0.214 | 355.1 | 0.215 |  |  |  |
| 155.12 | 0.043 | 205.12 | -0.007 | 255.12 | 0.132 |  | 305.12 | 0.212 | 355.12 | 0.22 |  |  |  |
| 155.14 | 0.046 | 205.14 | -0.021 | 255.14 | 0.133 |  | 305.14 | 0.207 | 355.14 | 0.228 |  |  |  |
| 155.16 | 0.031 | 205.16 | -0.008 | 255.16 | 0.137 |  | 305.16 | 0.21 | 355.16 | 0.204 |  |  |  |
| 155.18 | 0.029 | 205.18 | 0.011 | 255.18 | 0.13 |  | 305.18 | 0.158 | 355.18 | 0.17 |  |  |  |
| 155.2 | 0.036 | 205.2 | -0.007 | 255.2 | 0.112 |  | 305.2 | 0.166 | 355.2 | 0.228 |  |  |  |
| 155.22 | 0.037 | 205.22 | 0.005 | 255.22 | 0.106 |  | 305.22 | 0.178 | 355.22 | 0.195 |  |  |  |
| 155.24 | 0.036 | 205.24 | 0.001 | 255.24 | 0.094 |  | 305.24 | 0.212 | 355.24 | 0.182 |  |  |  |
| 155.26 | 0.027 | 205.26 | 0.034 | 255.26 | 0.141 |  | 305.26 | 0.23 | 355.26 | 0.19 |  |  |  |
| 155.28 | 0.024 | 205.28 | 0.048 | 255.28 | 0.158 |  | 305.28 | 0.238 | 355.28 | 0.228 |  |  |  |
| 155.3 | 0.027 | 205.3 | 0.039 | 255.3 | 0.165 |  | 305.3 | 0.248 | 355.3 | 0.067 |  |  |  |
| 155.32 | 0.037 | 205.32 | 0.063 | 255.32 | 0.171 |  | 305.32 | 0.247 | 355.32 | 0.088 |  |  |  |
| 155.34 | 0.031 | 205.34 | 0.07 | 255.34 | 0.181 |  | 305.34 | 0.242 | 355.34 | 0.139 |  |  |  |
| 155.36 | 0.03 | 205.36 | 0.087 | 255.36 | 0.177 |  | 305.36 | 0.226 | 355.36 | 0.215 |  |  |  |
| 155.38 | 0.041 | 205.38 | 0.13 | 255.38 | 0.186 |  | 305.38 | 0.219 | 355.38 | 0.221 |  |  |  |
| 155.4 | 0.043 | 205.4 | 0.254 | 255.4 | 0.2 |  | 305.4 | 0.205 | 355.4 | 0.278 |  |  |  |
| 155.42 | 0.045 | 205.42 | 0.353 | 255.42 | 0.199 |  | 305.42 | 0.213 | 355.42 | 0.282 |  |  |  |
| 155.44 | 0.049 | 205.44 | 0.297 | 255.44 | 0.211 |  | 305.44 | 0.2 | 355.44 | 0.261 |  |  |  |
| 155.46 | 0.046 | 205.46 | 0.159 | 255.46 | 0.188 |  | 305.46 | 0.21 | 355.46 | 0.245 |  |  |  |
| 155.48 | 0.039 | 205.48 | 0.001 | 255.48 | 0.216 |  | 305.48 | 0.21 | 355.48 | 0.202 |  |  |  |
| 155.5 | 0.017 | 205.5 | 0.022 | 255.5 | 0.232 |  | 305.5 | 0.21 | 355.5 | 0.181 |  |  |  |
| 155.52 | 0.017 | 205.52 | 0.019 | 255.52 | 0.219 |  | 305.52 | 0.206 | 355.52 | 0.157 |  |  |  |
| 155.54 | 0.017 | 205.54 | 0.024 | 255.54 | 0.201 |  | 305.54 | 0.203 | 355.54 | 0.163 |  |  |  |
| 155.56 | 0.024 | 205.56 | 0.018 | 255.56 | 0.183 |  | 305.56 | 0.203 | 355.56 | 0.133 |  |  |  |
| 155.58 | 0.026 | 205.58 | 0.011 | 255.58 | 0.182 |  | 305.58 | 0.205 | 355.58 | 0.132 |  |  |  |
| 155.6 | 0.025 | 205.6 | 0.004 | 255.6 | 0.183 |  | 305.6 | 0.214 | 355.6 | 0.185 |  |  |  |
| 155.62 | 0.02 | 205.62 | 0.008 | 255.62 | 0.176 |  | 305.62 | 0.217 | 355.62 | 0.215 |  |  |  |
| 155.64 | 0.034 | 205.64 | 0.006 | 255.64 | 0.162 |  | 305.64 | 0.216 | 355.64 | 0.203 |  |  |  |
| 155.66 | 0.037 | 205.66 | 0.009 | 255.66 | 0.048 |  | 305.66 | 0.19 | 355.66 | 0.179 |  |  |  |
| 155.68 | 0.034 | 205.68 | 0.01 | 255.68 | 0.032 |  | 305.68 | 0.173 | 355.68 | 0.17 |  |  |  |
| 155.7 | 0.027 | 205.7 | 0.014 | 255.7 | 0.009 |  | 305.7 | 0.165 | 355.7 | 0.141 |  |  |  |
| 155.72 | 0.027 | 205.72 | 0.022 | 255.72 | 0.026 |  | 305.72 | 0.126 | 355.72 | 0.118 |  |  |  |
| 155.74 | 0.027 | 205.74 | 0.041 | 255.74 | 0.04 |  | 305.74 | 0.187 | 355.74 | 0.131 |  |  |  |
| 155.76 | 0.029 | 205.76 | 0.062 | 255.76 | 0.05 |  | 305.76 | 0.247 | 355.76 | 0.158 |  |  |  |
| 155.78 | 0.031 | 205.78 | 0.049 | 255.78 | 0.057 |  | 305.78 | 0.268 | 355.78 | 0.188 |  |  |  |
| 155.8 | 0.034 | 205.8 | 0.066 | 255.8 | 0.074 |  | 305.8 | 0.233 | 355.8 | 0.161 |  |  |  |
| 155.82 | 0.036 | 205.82 | 0.061 | 255.82 | 0.152 |  | 305.82 | 0.224 | 355.82 | 0.162 |  |  |  |
| 155.84 | 0.04 | 205.84 | 0.074 | 255.84 | 0.203 |  | 305.84 | 0.21 | 355.84 | 0.204 |  |  |  |
| 155.86 | 0.043 | 205.86 | 0.073 | 255.86 | 0.215 |  | 305.86 | 0.195 | 355.86 | 0.192 |  |  |  |
| 155.88 | 0.048 | 205.88 | 0.105 | 255.88 | 0.17 |  | 305.88 | 0.188 | 355.88 | 0.159 |  |  |  |
| 155.9 | 0.055 | 205.9 | 0.125 | 255.9 | 0.153 |  | 305.9 | 0.198 | 355.9 | 0.149 |  |  |  |
| 155.92 | 0.002 | 205.92 | 0.107 | 255.92 | 0.169 |  | 305.92 | 0.216 | 355.92 | 0.16 |  |  |  |
| 155.94 | -0.001 | 205.94 | 0.075 | 255.94 | 0.176 |  | 305.94 | 0.224 | 355.94 | 0.183 |  |  |  |
| 155.96 | -0.044 | 205.96 | 0.127 | 255.96 | 0.182 |  | 305.96 | 0.218 | 355.96 | 0.105 |  |  |  |
| 155.98 | -0.029 | 205.98 | 0.123 | 255.98 | 0.185 |  | 305.98 | 0.215 | 355.98 | 0.127 |  |  |  |
| 156 | -0.024 | 206 | 0.107 | 256 | 0.167 |  | 306 | 0.216 | 356 | 0.11 |  |  |  |
| 156.02 | -0.024 | 206.02 | 0.135 | 256.02 | 0.157 |  | 306.02 | 0.218 | 356.02 | 0.109 |  |  |  |
| 156.04 | -0.024 | 206.04 | 0.129 | 256.04 | 0.141 |  | 306.04 | 0.216 | 356.04 | 0.126 |  |  |  |
| 156.06 | -0.022 | 206.06 | 0.1 | 256.06 | 0.017 |  | 306.06 | 0.226 | 356.06 | 0.133 |  |  |  |
| 156.08 | -0.016 | 206.08 | 0.124 | 256.08 | 0.027 |  | 306.08 | 0.215 | 356.08 | 0.138 |  |  |  |
| 156.1 | -0.003 | 206.1 | 0.128 | 256.1 | 0.026 |  | 306.1 | 0.21 | 356.1 | 0.1 |  |  |  |
| 156.12 | -0.002 | 206.12 | 0.127 | 256.12 | 0.029 |  | 306.12 | 0.221 | 356.12 | 0.184 |  |  |  |
| 156.14 | 0.008 | 206.14 | 0.106 | 256.14 | 0.022 |  | 306.14 | 0.174 | 356.14 | 0.164 |  |  |  |
| 156.16 | -0.015 | 206.16 | 0.101 | 256.16 | 0.017 |  | 306.16 | 0.177 | 356.16 | 0.076 |  |  |  |
| 156.18 | -0.04 | 206.18 | 0.109 | 256.18 | 0.029 |  | 306.18 | 0.203 | 356.18 | 0.103 |  |  |  |
| 156.2 | -0.016 | 206.2 | 0.101 | 256.2 | 0.04 |  | 306.2 | 0.208 | 356.2 | 0.132 |  |  |  |
| 156.22 | -0.001 | 206.22 | 0.106 | 256.22 | 0.049 |  | 306.22 | 0.002 | 356.22 | 0.157 |  |  |  |
| 156.24 | 0.008 | 206.24 | 0.137 | 256.24 | 0.047 |  | 306.24 | 0.021 | 356.24 | 0.133 |  |  |  |
| 156.26 | 0.015 | 206.26 | 0.14 | 256.26 | 0 |  | 306.26 | 0.004 | 356.26 | 0.147 |  |  |  |
| 156.28 | 0.015 | 206.28 | 0.137 | 256.28 | 0.151 |  | 306.28 | 0.037 | 356.28 | 0.153 |  |  |  |
| 156.3 | 0.047 | 206.3 | 0.113 | 256.3 | 0.256 |  | 306.3 | 0.061 | 356.3 | 0.174 |  |  |  |
| 156.32 | 0.031 | 206.32 | 0.111 | 256.32 | 0.211 |  | 306.32 | 0.068 | 356.32 | 0.17 |  |  |  |
| 156.34 | 0.022 | 206.34 | 0.11 | 256.34 | 0.119 |  | 306.34 | 0.089 | 356.34 | 0.176 |  |  |  |
| 156.36 | -0.007 | 206.36 | 0.107 | 256.36 | 0.104 |  | 306.36 | 0.106 | 356.36 | 0.183 |  |  |  |
| 156.38 | 0.017 | 206.38 | 0.104 | 256.38 | 0.123 |  | 306.38 | 0.11 | 356.38 | 0.225 |  |  |  |
| 156.4 | 0.02 | 206.4 | 0.092 | 256.4 | 0.134 |  | 306.4 | 0.115 | 356.4 | 0.254 |  |  |  |
| 156.42 | 0.026 | 206.42 | 0.117 | 256.42 | 0.138 |  | 306.42 | 0.13 | 356.42 | 0.264 |  |  |  |
| 156.44 | 0.015 | 206.44 | 0.11 | 256.44 | 0.139 |  | 306.44 | 0.145 | 356.44 | 0.261 |  |  |  |
| 156.46 | 0.025 | 206.46 | 0.109 | 256.46 | 0.074 |  | 306.46 | 0.162 | 356.46 | 0.255 |  |  |  |
| 156.48 | 0.035 | 206.48 | 0.03 | 256.48 | 0.069 |  | 306.48 | 0.164 | 356.48 | 0.252 |  |  |  |
| 156.5 | 0.039 | 206.5 | 0.1 | 256.5 | 0.086 |  | 306.5 | 0.165 | 356.5 | 0.25 |  |  |  |
| 156.52 | 0.042 | 206.52 | 0.06 | 256.52 | 0.103 |  | 306.52 | 0.175 | 356.52 | 0.235 |  |  |  |
| 156.54 | 0.042 | 206.54 | 0.089 | 256.54 | 0.099 |  | 306.54 | 0.177 | 356.54 | 0.227 |  |  |  |
| 156.56 | 0.044 | 206.56 | 0.068 | 256.56 | 0.103 |  | 306.56 | 0.181 | 356.56 | 0.216 |  |  |  |
| 156.58 | 0.027 | 206.58 | -0.004 | 256.58 | 0.1 |  | 306.58 | 0.186 | 356.58 | 0.205 |  |  |  |
| 156.6 | 0.026 | 206.6 | 0.01 | 256.6 | 0.104 |  | 306.6 | 0.19 | 356.6 | 0.222 |  |  |  |
| 156.62 | 0.032 | 206.62 | 0.024 | 256.62 | 0.103 |  | 306.62 | 0.216 | 356.62 | 0.229 |  |  |  |
| 156.64 | 0.039 | 206.64 | 0.041 | 256.64 | 0.091 |  | 306.64 | 0.212 | 356.64 | 0.215 |  |  |  |
| 156.66 | 0.002 | 206.66 | 0.054 | 256.66 | 0.092 |  | 306.66 | 0.215 | 356.66 | 0.209 |  |  |  |
| 156.68 | 0.003 | 206.68 | 0.145 | 256.68 | 0.094 |  | 306.68 | 0.209 | 356.68 | 0.215 |  |  |  |
| 156.7 | 0.005 | 206.7 | 0.197 | 256.7 | 0.093 |  | 306.7 | 0.203 | 356.7 | 0.222 |  |  |  |
| 156.72 | 0.001 | 206.72 | 0.127 | 256.72 | 0.08 |  | 306.72 | 0.212 | 356.72 | 0.22 |  |  |  |
| 156.74 | -0.004 | 206.74 | 0.125 | 256.74 | 0.05 |  | 306.74 | 0.213 | 356.74 | 0.207 |  |  |  |
| 156.76 | 0.033 | 206.76 | 0.128 | 256.76 | 0.087 |  | 306.76 | 0.227 | 356.76 | 0.196 |  |  |  |
| 156.78 | 0.045 | 206.78 | 0.082 | 256.78 | 0.096 |  | 306.78 | 0.2 | 356.78 | 0.183 |  |  |  |
| 156.8 | 0.039 | 206.8 | 0.071 | 256.8 | 0.094 |  | 306.8 | 0.224 | 356.8 | 0.181 |  |  |  |
| 156.82 | 0.036 | 206.82 | 0.073 | 256.82 | 0.109 |  | 306.82 | 0.228 | 356.82 | 0.188 |  |  |  |
| 156.84 | 0.031 | 206.84 | 0.073 | 256.84 | 0.131 |  | 306.84 | 0.232 | 356.84 | 0.173 |  |  |  |
| 156.86 | 0.031 | 206.86 | 0.06 | 256.86 | 0.012 |  | 306.86 | 0.234 | 356.86 | 0.181 |  |  |  |
| 156.88 | 0.027 | 206.88 | 0.123 | 256.88 | 0.009 |  | 306.88 | 0.217 | 356.88 | 0.172 |  |  |  |
| 156.9 | 0.03 | 206.9 | 0.16 | 256.9 | -0.006 |  | 306.9 | 0.207 | 356.9 | 0.168 |  |  |  |
| 156.92 | 0.021 | 206.92 | 0.163 | 256.92 | 0.064 |  | 306.92 | 0.196 | 356.92 | 0.183 |  |  |  |
| 156.94 | 0.009 | 206.94 | 0.116 | 256.94 | 0.058 |  | 306.94 | 0.206 | 356.94 | 0.19 |  |  |  |
| 156.96 | 0.005 | 206.96 | 0.096 | 256.96 | 0.044 |  | 306.96 | 0.214 | 356.96 | 0.183 |  |  |  |
| 156.98 | 0.008 | 206.98 | 0.058 | 256.98 | 0.043 |  | 306.98 | 0.223 | 356.98 | 0.181 |  |  |  |
| 157 | 0.009 | 207 | 0.053 | 257 | 0.034 |  | 307 | 0.187 | 357 | 0.185 |  |  |  |
| 157.02 | 0.024 | 207.02 | 0.044 | 257.02 | 0.044 |  | 307.02 | 0.181 | 357.02 | 0.184 |  |  |  |
| 157.04 | 0.032 | 207.04 | 0.057 | 257.04 | 0.052 |  | 307.04 | 0.224 | 357.04 | 0.191 |  |  |  |
| 157.06 | 0.026 | 207.06 | 0.04 | 257.06 | 0.063 |  | 307.06 | 0.227 | 357.06 | 0.184 |  |  |  |
| 157.08 | 0.024 | 207.08 | 0.034 | 257.08 | 0.077 |  | 307.08 | 0.204 | 357.08 | 0.174 |  |  |  |
| 157.1 | 0.023 | 207.1 | 0.042 | 257.1 | 0.079 |  | 307.1 | 0.181 | 357.1 | 0.17 |  |  |  |
| 157.12 | 0 | 207.12 | 0.061 | 257.12 | 0.067 |  | 307.12 | 0.175 | 357.12 | 0.166 |  |  |  |
| 157.14 | 0.011 | 207.14 | 0.067 | 257.14 | 0.058 |  | 307.14 | 0.188 | 357.14 | 0.185 |  |  |  |
| 157.16 | 0.011 | 207.16 | 0.057 | 257.16 | 0.069 |  | 307.16 | 0.195 | 357.16 | 0.202 |  |  |  |
| 157.18 | -0.001 | 207.18 | 0.05 | 257.18 | 0.086 |  | 307.18 | 0.185 | 357.18 | 0.2 |  |  |  |
| 157.2 | 0.012 | 207.2 | 0.037 | 257.2 | 0.105 |  | 307.2 | 0.198 | 357.2 | 0.195 |  |  |  |
| 157.22 | 0.021 | 207.22 | 0.03 | 257.22 | 0.128 |  | 307.22 | 0.192 | 357.22 | 0.209 |  |  |  |
| 157.24 | 0.022 | 207.24 | 0.021 | 257.24 | 0.135 |  | 307.24 | 0.176 | 357.24 | 0.215 |  |  |  |
| 157.26 | 0.026 | 207.26 | 0.041 | 257.26 | 0.145 |  | 307.26 | 0.178 | 357.26 | 0.213 |  |  |  |
| 157.28 | 0.05 | 207.28 | 0.047 | 257.28 | 0.135 |  | 307.28 | 0.173 | 357.28 | 0.19 |  |  |  |
| 157.3 | 0.052 | 207.3 | 0.066 | 257.3 | 0.175 |  | 307.3 | 0.172 | 357.3 | 0.191 |  |  |  |
| 157.32 | 0.057 | 207.32 | 0.06 | 257.32 | 0.177 |  | 307.32 | 0.182 | 357.32 | 0.189 |  |  |  |
| 157.34 | 0.065 | 207.34 | 0.092 | 257.34 | 0.155 |  | 307.34 | 0.214 | 357.34 | 0.191 |  |  |  |
| 157.36 | 0.03 | 207.36 | 0.141 | 257.36 | 0.131 |  | 307.36 | 0.219 | 357.36 | 0.193 |  |  |  |
| 157.38 | 0.011 | 207.38 | 0.217 | 257.38 | 0.118 |  | 307.38 | 0.216 | 357.38 | 0.182 |  |  |  |
| 157.4 | 0.043 | 207.4 | 0.257 | 257.4 | 0.126 |  | 307.4 | 0.188 | 357.4 | 0.175 |  |  |  |
| 157.42 | 0.045 | 207.42 | 0.258 | 257.42 | 0.175 |  | 307.42 | 0.177 | 357.42 | 0.178 |  |  |  |
| 157.44 | 0.105 | 207.44 | 0.203 | 257.44 | 0.218 |  | 307.44 | 0.173 | 357.44 | 0.19 |  |  |  |
| 157.46 | 0.122 | 207.46 | 0.146 | 257.46 | 0.273 |  | 307.46 | 0.171 | 357.46 | 0.198 |  |  |  |
| 157.48 | 0.091 | 207.48 | 0.158 | 257.48 | 0.313 |  | 307.48 | 0.173 | 357.48 | 0.176 |  |  |  |
| 157.5 | 0.071 | 207.5 | 0.158 | 257.5 | 0.261 |  | 307.5 | 0.172 | 357.5 | 0.173 |  |  |  |
| 157.52 | 0.076 | 207.52 | 0.152 | 257.52 | 0.141 |  | 307.52 | 0.165 | 357.52 | 0.163 |  |  |  |
| 157.54 | 0.077 | 207.54 | 0.14 | 257.54 | 0.114 |  | 307.54 | 0.165 | 357.54 | 0.171 |  |  |  |
| 157.56 | 0.108 | 207.56 | 0.13 | 257.56 | 0.104 |  | 307.56 | 0.174 | 357.56 | 0.198 |  |  |  |
| 157.58 | 0.146 | 207.58 | 0.131 | 257.58 | 0.098 |  | 307.58 | 0.179 | 357.58 | 0.2 |  |  |  |
| 157.6 | 0.147 | 207.6 | 0.149 | 257.6 | 0.121 |  | 307.6 | 0.18 | 357.6 | 0.185 |  |  |  |
| 157.62 | 0.096 | 207.62 | 0.25 | 257.62 | 0.114 |  | 307.62 | 0.178 | 357.62 | 0.18 |  |  |  |
| 157.64 | 0.085 | 207.64 | 0.316 | 257.64 | 0.106 |  | 307.64 | 0.176 | 357.64 | 0.182 |  |  |  |
| 157.66 | 0.079 | 207.66 | 0.154 | 257.66 | 0.257 |  | 307.66 | 0.172 | 357.66 | 0.193 |  |  |  |
| 157.68 | 0.071 | 207.68 | 0.113 | 257.68 | 0.235 |  | 307.68 | 0.165 | 357.68 | 0.203 |  |  |  |
| 157.7 | 0.058 | 207.7 | 0.109 | 257.7 | 0.188 |  | 307.7 | 0.169 | 357.7 | 0.194 |  |  |  |
| 157.72 | 0.047 | 207.72 | 0.128 | 257.72 | 0.15 |  | 307.72 | 0.165 | 357.72 | 0.186 |  |  |  |
| 157.74 | 0.061 | 207.74 | 0.178 | 257.74 | 0.094 |  | 307.74 | 0.156 | 357.74 | 0.189 |  |  |  |
| 157.76 | 0.106 | 207.76 | 0.228 | 257.76 | 0.093 |  | 307.76 | 0.146 | 357.76 | 0.203 |  |  |  |
| 157.78 | 0.166 | 207.78 | 0.259 | 257.78 | 0.08 |  | 307.78 | 0.131 | 357.78 | 0.212 |  |  |  |
| 157.8 | 0.221 | 207.8 | 0.24 | 257.8 | 0.079 |  | 307.8 | 0.139 | 357.8 | 0.21 |  |  |  |
| 157.82 | 0.226 | 207.82 | 0.182 | 257.82 | 0.08 |  | 307.82 | 0.139 | 357.82 | 0.21 |  |  |  |
| 157.84 | 0.219 | 207.84 | 0.134 | 257.84 | 0.078 |  | 307.84 | 0.133 | 357.84 | 0.209 |  |  |  |
| 157.86 | 0.19 | 207.86 | 0.113 | 257.86 | 0.078 |  | 307.86 | 0.133 | 357.86 | 0.211 |  |  |  |
| 157.88 | 0.126 | 207.88 | 0.107 | 257.88 | 0.078 |  | 307.88 | 0.079 | 357.88 | 0.207 |  |  |  |
| 157.9 | 0.123 | 207.9 | 0.1 | 257.9 | 0.077 |  | 307.9 | 0.052 | 357.9 | 0.2 |  |  |  |
| 157.92 | 0.147 | 207.92 | 0.09 | 257.92 | 0.064 |  | 307.92 | 0.067 | 357.92 | 0.196 |  |  |  |
| 157.94 | 0.138 | 207.94 | 0.093 | 257.94 | 0.078 |  | 307.94 | 0.143 | 357.94 | 0.196 |  |  |  |
| 157.96 | 0.146 | 207.96 | 0.096 | 257.96 | 0.083 |  | 307.96 | 0.169 | 357.96 | 0.203 |  |  |  |
| 157.98 | 0.148 | 207.98 | 0.103 | 257.98 | 0.079 |  | 307.98 | 0.184 | 357.98 | 0.211 |  |  |  |
| 158 | 0.152 | 208 | 0.097 | 258 | 0.075 |  | 308 | 0.192 | 358 | 0.211 |  |  |  |
| 158.02 | 0.146 | 208.02 | 0.088 | 258.02 | 0.077 |  | 308.02 | 0.171 | 358.02 | 0.211 |  |  |  |
| 158.04 | 0.121 | 208.04 | 0.044 | 258.04 | 0.076 |  | 308.04 | 0.166 | 358.04 | 0.211 |  |  |  |
| 158.06 | 0.101 | 208.06 | 0.1 | 258.06 | 0.009 |  | 308.06 | 0.165 | 358.06 | 0.209 |  |  |  |
| 158.08 | 0.077 | 208.08 | 0.14 | 258.08 | 0.013 |  | 308.08 | 0.172 | 358.08 | 0.189 |  |  |  |
| 158.1 | 0.018 | 208.1 | 0.139 | 258.1 | 0.015 |  | 308.1 | 0.185 | 358.1 | 0.189 |  |  |  |
| 158.12 | 0.019 | 208.12 | 0.115 | 258.12 | 0.019 |  | 308.12 | 0.212 | 358.12 | 0.193 |  |  |  |
| 158.14 | 0.02 | 208.14 | 0.082 | 258.14 | 0.027 |  | 308.14 | 0.212 | 358.14 | 0.206 |  |  |  |
| 158.16 | 0.137 | 208.16 | 0.031 | 258.16 | 0.033 |  | 308.16 | 0.184 | 358.16 | 0.212 |  |  |  |
| 158.18 | 0.273 | 208.18 | 0.059 | 258.18 | 0.02 |  | 308.18 | 0.179 | 358.18 | 0.209 |  |  |  |
| 158.2 | 0.288 | 208.2 | 0.068 | 258.2 | 0.055 |  | 308.2 | 0.007 | 358.2 | 0.203 |  |  |  |
| 158.22 | 0.239 | 208.22 | 0.098 | 258.22 | 0.063 |  | 308.22 | 0.019 | 358.22 | 0.195 |  |  |  |
| 158.24 | 0.176 | 208.24 | 0.115 | 258.24 | 0.066 |  | 308.24 | 0.032 | 358.24 | 0.108 |  |  |  |
| 158.26 | 0.146 | 208.26 | 0.135 | 258.26 | 0.072 |  | 308.26 | 0.064 | 358.26 | 0.193 |  |  |  |
| 158.28 | 0.144 | 208.28 | 0.147 | 258.28 | 0.077 |  | 308.28 | 0.073 | 358.28 | 0.195 |  |  |  |
| 158.3 | 0.147 | 208.3 | 0.155 | 258.3 | 0.074 |  | 308.3 | 0.082 | 358.3 | 0.18 |  |  |  |
| 158.32 | 0.013 | 208.32 | 0.159 | 258.32 | 0.079 |  | 308.32 | 0.07 | 358.32 | 0.099 |  |  |  |
| 158.34 | 0.032 | 208.34 | 0.144 | 258.34 | 0.078 |  | 308.34 | 0.055 | 358.34 | 0.13 |  |  |  |
| 158.36 | 0.023 | 208.36 | 0.12 | 258.36 | 0.076 |  | 308.36 | 0.068 | 358.36 | 0.182 |  |  |  |
| 158.38 | 0.027 | 208.38 | 0.12 | 258.38 | 0.082 |  | 308.38 | 0.103 | 358.38 | 0.198 |  |  |  |
| 158.4 | 0.071 | 208.4 | 0.135 | 258.4 | 0.082 |  | 308.4 | 0.149 | 358.4 | 0.215 |  |  |  |
| 158.42 | 0.081 | 208.42 | 0.201 | 258.42 | 0.095 |  | 308.42 | 0.202 | 358.42 | 0.212 |  |  |  |
| 158.44 | 0.05 | 208.44 | 0.237 | 258.44 | 0.098 |  | 308.44 | 0.219 | 358.44 | 0.229 |  |  |  |
| 158.46 | 0.034 | 208.46 | 0.111 | 258.46 | 0.117 |  | 308.46 | 0.177 | 358.46 | 0.234 |  |  |  |
| 158.48 | 0.038 | 208.48 | 0.113 | 258.48 | 0.105 |  | 308.48 | 0.167 | 358.48 | 0.235 |  |  |  |
| 158.5 | 0.041 | 208.5 | 0.158 | 258.5 | 0.098 |  | 308.5 | 0.189 | 358.5 | 0.235 |  |  |  |
| 158.52 | 0.039 | 208.52 | 0.207 | 258.52 | 0.09 |  | 308.52 | 0.216 | 358.52 | 0.223 |  |  |  |
| 158.54 | 0.038 | 208.54 | 0.19 | 258.54 | 0.087 |  | 308.54 | 0.23 | 358.54 | 0.214 |  |  |  |
| 158.56 | 0.039 | 208.56 | 0.108 | 258.56 | 0.09 |  | 308.56 | 0.245 | 358.56 | 0.206 |  |  |  |
| 158.58 | 0.053 | 208.58 | 0.094 | 258.58 | 0.096 |  | 308.58 | 0.257 | 358.58 | 0.211 |  |  |  |
| 158.6 | 0.062 | 208.6 | 0.073 | 258.6 | 0.109 |  | 308.6 | 0.158 | 358.6 | 0.185 |  |  |  |
| 158.62 | 0.05 | 208.62 | 0.044 | 258.62 | 0.129 |  | 308.62 | 0.17 | 358.62 | 0.195 |  |  |  |
| 158.64 | 0.066 | 208.64 | 0.058 | 258.64 | 0.127 |  | 308.64 | 0.178 | 358.64 | 0.204 |  |  |  |
| 158.66 | 0.068 | 208.66 | 0.102 | 258.66 | 0.118 |  | 308.66 | 0.18 | 358.66 | 0.2 |  |  |  |
| 158.68 | 0.088 | 208.68 | 0.101 | 258.68 | 0.118 |  | 308.68 | 0.182 | 358.68 | 0.203 |  |  |  |
| 158.7 | 0.118 | 208.7 | 0.141 | 258.7 | 0.135 |  | 308.7 | 0.178 | 358.7 | 0.205 |  |  |  |
| 158.72 | 0.01 | 208.72 | 0.211 | 258.72 | 0.166 |  | 308.72 | 0.186 | 358.72 | 0.21 |  |  |  |
| 158.74 | 0.067 | 208.74 | 0.103 | 258.74 | 0.181 |  | 308.74 | 0.192 | 358.74 | 0.204 |  |  |  |
| 158.76 | 0.077 | 208.76 | 0.065 | 258.76 | 0.186 |  | 308.76 | 0.189 | 358.76 | 0.147 |  |  |  |
| 158.78 | 0.025 | 208.78 | 0.049 | 258.78 | 0.189 |  | 308.78 | 0.191 | 358.78 | 0.15 |  |  |  |
| 158.8 | 0 | 208.8 | 0.121 | 258.8 | 0.19 |  | 308.8 | 0.202 | 358.8 | 0.182 |  |  |  |
| 158.82 | 0.005 | 208.82 | 0.134 | 258.82 | 0.196 |  | 308.82 | 0.219 | 358.82 | 0.179 |  |  |  |
| 158.84 | 0 | 208.84 | 0.134 | 258.84 | 0.213 |  | 308.84 | 0.252 | 358.84 | 0.177 |  |  |  |
| 158.86 | -0.013 | 208.86 | 0.048 | 258.86 | 0.036 |  | 308.86 | 0.261 | 358.86 | 0.201 |  |  |  |
| 158.88 | -0.011 | 208.88 | 0.209 | 258.88 | 0.047 |  | 308.88 | 0.197 | 358.88 | 0.239 |  |  |  |
| 158.9 | 0.01 | 208.9 | 0.21 | 258.9 | 0.063 |  | 308.9 | 0.199 | 358.9 | 0.205 |  |  |  |
| 158.92 | 0.021 | 208.92 | 0.193 | 258.92 | 0.075 |  | 308.92 | 0.164 | 358.92 | 0.198 |  |  |  |
| 158.94 | 0.142 | 208.94 | 0.216 | 258.94 | 0.08 |  | 308.94 | 0.127 | 358.94 | 0.222 |  |  |  |
| 158.96 | 0.145 | 208.96 | 0.186 | 258.96 | 0.091 |  | 308.96 | 0.081 | 358.96 | 0.223 |  |  |  |
| 158.98 | 0.125 | 208.98 | 0.114 | 258.98 | 0.095 |  | 308.98 | 0.051 | 358.98 | 0.211 |  |  |  |
| 159 | 0.116 | 209 | 0.11 | 259 | 0.084 |  | 309 | 0.064 | 359 | 0.213 |  |  |  |
| 159.02 | 0.116 | 209.02 | 0.112 | 259.02 | 0.023 |  | 309.02 | 0.097 | 359.02 | 0.206 |  |  |  |
| 159.04 | 0.124 | 209.04 | 0.102 | 259.04 | 0.049 |  | 309.04 | 0.096 | 359.04 | 0.189 |  |  |  |
| 159.06 | 0.135 | 209.06 | 0.1 | 259.06 | 0.062 |  | 309.06 | 0.102 | 359.06 | 0.196 |  |  |  |
| 159.08 | 0.128 | 209.08 | 0.193 | 259.08 | 0.073 |  | 309.08 | 0.098 | 359.08 | 0.206 |  |  |  |
| 159.1 | 0.13 | 209.1 | 0.169 | 259.1 | 0.089 |  | 309.1 | 0.119 | 359.1 | 0.194 |  |  |  |
| 159.12 | 0.102 | 209.12 | 0.049 | 259.12 | 0.105 |  | 309.12 | 0.117 | 359.12 | 0.166 |  |  |  |
| 159.14 | 0.106 | 209.14 | 0.105 | 259.14 | 0.115 |  | 309.14 | 0.159 | 359.14 | 0.16 |  |  |  |
| 159.16 | 0.11 | 209.16 | 0.123 | 259.16 | 0.126 |  | 309.16 | 0.203 | 359.16 | 0.172 |  |  |  |
| 159.18 | 0.121 | 209.18 | 0.104 | 259.18 | 0.138 |  | 309.18 | 0.212 | 359.18 | 0.18 |  |  |  |
| 159.2 | 0.112 | 209.2 | 0.092 | 259.2 | 0.143 |  | 309.2 | 0.2 | 359.2 | 0.195 |  |  |  |
| 159.22 | 0.11 | 209.22 | 0.083 | 259.22 | 0.146 |  | 309.22 | 0.204 | 359.22 | 0.2 |  |  |  |
| 159.24 | 0.107 | 209.24 | 0.144 | 259.24 | 0.153 |  | 309.24 | 0.21 | 359.24 | 0.197 |  |  |  |
| 159.26 | 0.104 | 209.26 | 0.17 | 259.26 | 0.007 |  | 309.26 | 0.15 | 359.26 | 0.213 |  |  |  |
| 159.28 | 0.095 | 209.28 | 0.176 | 259.28 | -0.003 |  | 309.28 | 0.164 | 359.28 | 0.22 |  |  |  |
| 159.3 | 0.093 | 209.3 | 0.167 | 259.3 | -0.006 |  | 309.3 | 0.204 | 359.3 | 0.217 |  |  |  |
| 159.32 | 0.096 | 209.32 | 0.133 | 259.32 | 0.01 |  | 309.32 | 0.178 | 359.32 | 0.217 |  |  |  |
| 159.34 | 0.099 | 209.34 | 0.108 | 259.34 | 0.024 |  | 309.34 | 0.16 | 359.34 | 0.22 |  |  |  |
| 159.36 | 0.108 | 209.36 | 0.096 | 259.36 | 0.033 |  | 309.36 | 0.16 | 359.36 | 0.213 |  |  |  |
| 159.38 | 0.118 | 209.38 | 0.101 | 259.38 | 0.042 |  | 309.38 | 0.196 | 359.38 | 0.19 |  |  |  |
| 159.4 | 0.129 | 209.4 | 0.116 | 259.4 | 0.05 |  | 309.4 | 0.181 | 359.4 | 0.161 |  |  |  |
| 159.42 | 0.154 | 209.42 | 0.148 | 259.42 | 0.072 |  | 309.42 | 0.146 | 359.42 | 0.139 |  |  |  |
| 159.44 | 0.197 | 209.44 | 0.158 | 259.44 | 0.116 |  | 309.44 | 0.127 | 359.44 | 0.135 |  |  |  |
| 159.46 | 0.257 | 209.46 | 0.155 | 259.46 | 0.147 |  | 309.46 | 0.13 | 359.46 | 0.137 |  |  |  |
| 159.48 | 0.299 | 209.48 | 0.15 | 259.48 | 0.2 |  | 309.48 | 0.136 | 359.48 | 0.135 |  |  |  |
| 159.5 | 0.257 | 209.5 | 0.015 | 259.5 | 0.201 |  | 309.5 | 0.105 | 359.5 | 0.132 |  |  |  |
| 159.52 | 0.065 | 209.52 | -0.032 | 259.52 | 0.14 |  | 309.52 | 0.11 | 359.52 | 0.146 |  |  |  |
| 159.54 | 0.065 | 209.54 | -0.048 | 259.54 | 0.012 |  | 309.54 | 0.13 | 359.54 | 0.152 |  |  |  |
| 159.56 | 0.124 | 209.56 | -0.048 | 259.56 | -0.001 |  | 309.56 | 0.16 | 359.56 | 0.177 |  |  |  |
| 159.58 | 0.155 | 209.58 | -0.04 | 259.58 | 0.022 |  | 309.58 | 0.217 | 359.58 | 0.22 |  |  |  |
| 159.6 | 0.188 | 209.6 | -0.03 | 259.6 | 0.079 |  | 309.6 | 0.232 | 359.6 | 0.216 |  |  |  |
| 159.62 | 0.187 | 209.62 | -0.009 | 259.62 | 0.092 |  | 309.62 | 0.235 | 359.62 | 0.214 |  |  |  |
| 159.64 | 0.183 | 209.64 | 0.01 | 259.64 | 0.089 |  | 309.64 | 0.231 | 359.64 | 0.22 |  |  |  |
| 159.66 | 0.169 | 209.66 | 0.02 | 259.66 | 0.072 |  | 309.66 | 0.234 | 359.66 | 0.216 |  |  |  |
| 159.68 | 0.155 | 209.68 | 0.026 | 259.68 | 0.08 |  | 309.68 | 0.234 | 359.68 | 0.214 |  |  |  |
| 159.7 | 0.148 | 209.7 | 0.038 | 259.7 | 0.095 |  | 309.7 | 0.22 | 359.7 | 0.198 |  |  |  |
| 159.72 | 0.146 | 209.72 | 0.064 | 259.72 | 0.108 |  | 309.72 | 0.17 | 359.72 | 0.196 |  |  |  |
| 159.74 | 0.143 | 209.74 | 0.086 | 259.74 | 0.106 |  | 309.74 | 0.138 | 359.74 | 0.174 |  |  |  |
| 159.76 | 0.128 | 209.76 | 0.092 | 259.76 | 0.109 |  | 309.76 | 0.132 | 359.76 | 0.146 |  |  |  |
| 159.78 | 0.124 | 209.78 | 0.103 | 259.78 | 0.118 |  | 309.78 | 0.143 | 359.78 | 0.124 |  |  |  |
| 159.8 | 0.127 | 209.8 | 0.112 | 259.8 | 0.128 |  | 309.8 | 0.16 | 359.8 | 0.117 |  |  |  |
| 159.82 | 0.134 | 209.82 | 0.129 | 259.82 | 0.145 |  | 309.82 | 0.188 | 359.82 | 0.124 |  |  |  |
| 159.84 | 0.14 | 209.84 | 0.146 | 259.84 | 0.132 |  | 309.84 | 0.201 | 359.84 | 0.144 |  |  |  |
| 159.86 | 0.153 | 209.86 | 0.139 | 259.86 | 0.142 |  | 309.86 | 0.198 | 359.86 | 0.185 |  |  |  |
| 159.88 | 0.145 | 209.88 | 0.132 | 259.88 | 0.156 |  | 309.88 | 0.194 | 359.88 | 0.225 |  |  |  |
| 159.9 | 0.143 | 209.9 | 0.016 | 259.9 | 0.183 |  | 309.9 | 0.202 | 359.9 | 0.229 |  |  |  |
| 159.92 | 0.01 | 209.92 | 0.048 | 259.92 | 0.188 |  | 309.92 | 0.188 | 359.92 | 0.232 |  |  |  |
| 159.94 | -0.005 | 209.94 | 0.086 | 259.94 | 0.175 |  | 309.94 | 0.195 | 359.94 | 0.225 |  |  |  |
| 159.96 | -0.039 | 209.96 | 0.082 | 259.96 | 0.18 |  | 309.96 | 0.186 | 359.96 | 0.221 |  |  |  |
| 159.98 | 0.031 | 209.98 | 0.064 | 259.98 | 0.177 |  | 309.98 | 0.126 | 359.98 | 0.214 |  |  |  |
| 160 | 0.005 | 210 | 0.047 | 260 | 0.16 |  | 310 | 0.096 | 360 | 0.215 |  |  |  |
| 160.02 | 0.006 | 210.02 | 0.066 | 260.02 | 0.144 |  | 310.02 | 0.09 | 360.02 | 0.2 |  |  |  |
| 160.04 | -0.001 | 210.04 | 0.087 | 260.04 | 0.152 |  | 310.04 | 0.105 | 360.04 | 0.204 |  |  |  |
| 160.06 | 0.015 | 210.06 | 0.089 | 260.06 | 0.083 |  | 310.06 | 0.187 | 360.06 | 0.098 |  |  |  |
| 160.08 | 0.067 | 210.08 | 0.08 | 260.08 | 0.087 |  | 310.08 | 0.187 | 360.08 | 0.156 |  |  |  |
| 160.1 | 0.056 | 210.1 | 0.092 | 260.1 | 0.102 |  | 310.1 | 0.188 | 360.1 | 0.153 |  |  |  |
| 160.12 | 0.057 | 210.12 | 0.105 | 260.12 | 0.153 |  | 310.12 | 0.202 | 360.12 | 0.162 |  |  |  |
| 160.14 | 0.078 | 210.14 | 0.112 | 260.14 | 0.156 |  | 310.14 | 0.2 | 360.14 | 0.171 |  |  |  |
| 160.16 | 0.067 | 210.16 | 0.1 | 260.16 | 0.154 |  | 310.16 | 0.2 | 360.16 | 0.171 |  |  |  |
| 160.18 | 0.064 | 210.18 | 0.122 | 260.18 | 0.16 |  | 310.18 | 0.18 | 360.18 | 0.183 |  |  |  |
| 160.2 | 0.056 | 210.2 | 0.108 | 260.2 | 0.158 |  | 310.2 | 0.174 | 360.2 | 0.186 |  |  |  |
| 160.22 | 0.042 | 210.22 | 0.12 | 260.22 | 0.156 |  | 310.22 | 0.165 | 360.22 | 0.2 |  |  |  |
| 160.24 | 0.038 | 210.24 | 0.111 | 260.24 | 0.159 |  | 310.24 | 0.168 | 360.24 | 0.1 |  |  |  |
| 160.26 | 0.02 | 210.26 | 0.17 | 260.26 | 0.161 |  | 310.26 | 0.179 | 360.26 | 0.184 |  |  |  |
| 160.28 | 0.014 | 210.28 | 0.204 | 260.28 | 0.165 |  | 310.28 | 0.18 | 360.28 | 0.169 |  |  |  |
| 160.3 | 0.018 | 210.3 | 0.189 | 260.3 | 0.137 |  | 310.3 | 0.178 | 360.3 | 0.126 |  |  |  |
| 160.32 | 0.028 | 210.32 | 0.147 | 260.32 | 0.151 |  | 310.32 | 0.174 | 360.32 | 0.225 |  |  |  |
| 160.34 | 0.063 | 210.34 | 0.117 | 260.34 | 0.212 |  | 310.34 | 0.176 | 360.34 | 0.249 |  |  |  |
| 160.36 | 0.096 | 210.36 | 0.095 | 260.36 | 0.189 |  | 310.36 | 0.2 | 360.36 | 0.214 |  |  |  |
| 160.38 | 0.108 | 210.38 | 0.115 | 260.38 | 0.164 |  | 310.38 | 0.213 | 360.38 | 0.239 |  |  |  |
| 160.4 | 0.113 | 210.4 | 0.135 | 260.4 | 0.152 |  | 310.4 | 0.213 | 360.4 | 0.243 |  |  |  |
| 160.42 | 0.119 | 210.42 | 0.158 | 260.42 | 0.161 |  | 310.42 | 0.208 | 360.42 | 0.232 |  |  |  |
| 160.44 | 0.121 | 210.44 | 0.128 | 260.44 | 0.161 |  | 310.44 | 0.208 | 360.44 | 0.091 |  |  |  |
| 160.46 | 0.125 | 210.46 | 0.078 | 260.46 | 0.071 |  | 310.46 | 0.21 | 360.46 | 0.16 |  |  |  |
| 160.48 | 0.111 | 210.48 | 0.089 | 260.48 | 0.006 |  | 310.48 | 0.201 | 360.48 | 0.164 |  |  |  |
| 160.5 | 0.1 | 210.5 | 0.016 | 260.5 | 0.013 |  | 310.5 | 0.064 | 360.5 | 0.101 |  |  |  |
| 160.52 | 0.118 | 210.52 | 0.012 | 260.52 | 0.036 |  | 310.52 | 0.078 | 360.52 | 0.131 |  |  |  |
| 160.54 | 0.097 | 210.54 | 0.009 | 260.54 | 0.093 |  | 310.54 | 0.103 | 360.54 | 0.175 |  |  |  |
| 160.56 | 0.084 | 210.56 | 0.01 | 260.56 | 0.109 |  | 310.56 | 0.115 | 360.56 | 0.226 |  |  |  |
| 160.58 | 0.095 | 210.58 | 0.015 | 260.58 | 0.145 |  | 310.58 | 0.115 | 360.58 | 0.216 |  |  |  |
| 160.6 | 0.105 | 210.6 | 0.015 | 260.6 | 0.15 |  | 310.6 | 0.11 | 360.6 | 0.161 |  |  |  |
| 160.62 | 0.112 | 210.62 | 0.02 | 260.62 | 0.156 |  | 310.62 | 0.122 | 360.62 | 0.183 |  |  |  |
| 160.64 | 0.119 | 210.64 | 0.023 | 260.64 | 0.154 |  | 310.64 | 0.132 | 360.64 | 0.16 |  |  |  |
| 160.66 | 0.115 | 210.66 | 0.022 | 260.66 | 0.154 |  | 310.66 | 0.159 | 360.66 | 0.143 |  |  |  |
| 160.68 | 0.107 | 210.68 | 0.025 | 260.68 | 0.152 |  | 310.68 | 0.149 | 360.68 | 0.142 |  |  |  |
| 160.7 | 0.085 | 210.7 | 0.009 | 260.7 | 0.155 |  | 310.7 | 0.133 | 360.7 | 0.156 |  |  |  |
| 160.72 | 0.093 | 210.72 | 0.006 | 260.72 | 0.165 |  | 310.72 | 0.122 | 360.72 | 0.16 |  |  |  |
| 160.74 | 0.114 | 210.74 | 0.002 | 260.74 | 0.156 |  | 310.74 | 0.142 | 360.74 | 0.168 |  |  |  |
| 160.76 | 0.06 | 210.76 | -0.014 | 260.76 | 0.178 |  | 310.76 | 0.142 | 360.76 | 0.189 |  |  |  |
| 160.78 | 0.086 | 210.78 | -0.002 | 260.78 | 0.175 |  | 310.78 | 0.141 | 360.78 | 0.168 |  |  |  |
| 160.8 | 0.08 | 210.8 | 0.011 | 260.8 | 0.169 |  | 310.8 | 0.146 | 360.8 | 0.185 |  |  |  |
| 160.82 | 0.09 | 210.82 | 0.012 | 260.82 | 0.175 |  | 310.82 | 0.151 | 360.82 | 0.206 |  |  |  |
| 160.84 | 0.201 | 210.84 | 0.009 | 260.84 | 0.196 |  | 310.84 | 0.158 | 360.84 | 0.213 |  |  |  |
| 160.86 | 0.171 | 210.86 | 0.003 | 260.86 | 0.187 |  | 310.86 | 0.17 | 360.86 | 0.161 |  |  |  |
| 160.88 | 0.127 | 210.88 | 0.002 | 260.88 | 0.187 |  | 310.88 | 0.187 | 360.88 | 0.16 |  |  |  |
| 160.9 | 0.126 | 210.9 | 0.005 | 260.9 | 0.186 |  | 310.9 | 0.097 | 360.9 | 0.178 |  |  |  |
| 160.92 | 0.133 | 210.92 | 0.007 | 260.92 | 0.189 |  | 310.92 | 0.115 | 360.92 | 0.202 |  |  |  |
| 160.94 | 0.126 | 210.94 | 0.009 | 260.94 | 0.189 |  | 310.94 | 0.14 | 360.94 | 0.216 |  |  |  |
| 160.96 | 0.136 | 210.96 | 0.009 | 260.96 | 0.177 |  | 310.96 | 0.157 | 360.96 | 0.21 |  |  |  |
| 160.98 | 0.111 | 210.98 | 0.008 | 260.98 | 0.168 |  | 310.98 | 0.156 | 360.98 | 0.194 |  |  |  |
| 161 | 0.052 | 211 | 0.001 | 261 | 0.164 |  | 311 | 0.056 | 361 | 0.168 |  |  |  |
| 161.02 | 0.026 | 211.02 | 0.026 | 261.02 | 0.161 |  | 311.02 | 0.049 | 361.02 | 0.158 |  |  |  |
| 161.04 | 0.029 | 211.04 | 0.029 | 261.04 | 0.187 |  | 311.04 | 0.065 | 361.04 | 0.15 |  |  |  |
| 161.06 | 0.029 | 211.06 | 0.026 | 261.06 | 0.204 |  | 311.06 | 0.096 | 361.06 | 0.155 |  |  |  |
| 161.08 | 0.028 | 211.08 | 0.022 | 261.08 | 0.236 |  | 311.08 | 0.1 | 361.08 | 0.166 |  |  |  |
| 161.1 | 0.024 | 211.1 | 0.063 | 261.1 | 0.239 |  | 311.1 | 0.098 | 361.1 | 0.166 |  |  |  |
| 161.12 | 0.014 | 211.12 | 0.061 | 261.12 | 0.233 |  | 311.12 | 0.135 | 361.12 | 0.182 |  |  |  |
| 161.14 | 0.002 | 211.14 | 0.056 | 261.14 | 0.197 |  | 311.14 | 0.158 | 361.14 | 0.187 |  |  |  |
| 161.16 | 0 | 211.16 | 0.053 | 261.16 | 0.169 |  | 311.16 | 0.171 | 361.16 | 0.192 |  |  |  |
| 161.18 | -0.006 | 211.18 | 0.053 | 261.18 | 0.158 |  | 311.18 | 0.195 | 361.18 | 0.196 |  |  |  |
| 161.2 | -0.001 | 211.2 | 0.046 | 261.2 | 0.156 |  | 311.2 | 0.218 | 361.2 | 0.17 |  |  |  |
| 161.22 | 0.002 | 211.22 | 0.039 | 261.22 | 0.158 |  | 311.22 | 0.192 | 361.22 | 0.166 |  |  |  |
| 161.24 | 0.002 | 211.24 | 0.035 | 261.24 | 0.164 |  | 311.24 | 0.198 | 361.24 | 0.165 |  |  |  |
| 161.26 | -0.007 | 211.26 | 0.031 | 261.26 | 0.154 |  | 311.26 | 0.205 | 361.26 | 0.176 |  |  |  |
| 161.28 | -0.006 | 211.28 | 0.033 | 261.28 | 0.117 |  | 311.28 | 0.207 | 361.28 | 0.177 |  |  |  |
| 161.3 | 0 | 211.3 | 0.029 | 261.3 | 0.095 |  | 311.3 | 0.192 | 361.3 | 0.175 |  |  |  |
| 161.32 | 0.023 | 211.32 | 0.017 | 261.32 | 0.126 |  | 311.32 | 0.179 | 361.32 | 0.174 |  |  |  |
| 161.34 | 0.051 | 211.34 | 0.011 | 261.34 | 0.125 |  | 311.34 | 0.171 | 361.34 | 0.199 |  |  |  |
| 161.36 | 0.084 | 211.36 | 0.005 | 261.36 | 0.136 |  | 311.36 | 0.19 | 361.36 | 0.204 |  |  |  |
| 161.38 | 0.107 | 211.38 | 0.006 | 261.38 | 0.143 |  | 311.38 | 0.192 | 361.38 | 0.215 |  |  |  |
| 161.4 | 0.114 | 211.4 | 0.008 | 261.4 | 0.144 |  | 311.4 | 0.189 | 361.4 | 0.223 |  |  |  |
| 161.42 | 0.102 | 211.42 | 0.012 | 261.42 | 0.135 |  | 311.42 | 0.185 | 361.42 | 0.231 |  |  |  |
| 161.44 | 0.115 | 211.44 | 0.021 | 261.44 | 0.153 |  | 311.44 | 0.176 | 361.44 | 0.232 |  |  |  |
| 161.46 | 0.13 | 211.46 | 0.03 | 261.46 | 0.171 |  | 311.46 | 0.183 | 361.46 | 0.212 |  |  |  |
| 161.48 | 0.124 | 211.48 | 0.037 | 261.48 | 0.177 |  | 311.48 | 0.193 | 361.48 | 0.186 |  |  |  |
| 161.5 | 0.097 | 211.5 | 0.037 | 261.5 | 0.193 |  | 311.5 | 0.195 | 361.5 | 0.174 |  |  |  |
| 161.52 | 0.11 | 211.52 | 0.039 | 261.52 | 0.215 |  | 311.52 | 0.178 | 361.52 | 0.166 |  |  |  |
| 161.54 | -0.006 | 211.54 | 0.033 | 261.54 | 0.218 |  | 311.54 | 0.163 | 361.54 | 0.16 |  |  |  |
| 161.56 | 0.044 | 211.56 | 0.023 | 261.56 | 0.199 |  | 311.56 | 0.17 | 361.56 | 0.163 |  |  |  |
| 161.58 | 0.088 | 211.58 | 0.028 | 261.58 | 0.171 |  | 311.58 | 0.19 | 361.58 | 0.174 |  |  |  |
| 161.6 | 0.109 | 211.6 | 0.039 | 261.6 | 0.189 |  | 311.6 | 0.2 | 361.6 | 0.187 |  |  |  |
| 161.62 | 0.102 | 211.62 | 0.048 | 261.62 | 0.214 |  | 311.62 | 0.191 | 361.62 | 0.185 |  |  |  |
| 161.64 | 0.091 | 211.64 | 0.056 | 261.64 | 0.201 |  | 311.64 | 0.146 | 361.64 | 0.175 |  |  |  |
| 161.66 | 0.083 | 211.66 | 0.062 | 261.66 | 0.195 |  | 311.66 | 0.145 | 361.66 | 0.162 |  |  |  |
| 161.68 | 0.076 | 211.68 | 0.065 | 261.68 | 0.198 |  | 311.68 | 0.148 | 361.68 | 0.165 |  |  |  |
| 161.7 | 0.074 | 211.7 | 0.056 | 261.7 | 0.141 |  | 311.7 | 0.146 | 361.7 | 0.192 |  |  |  |
| 161.72 | 0.063 | 211.72 | 0.048 | 261.72 | 0.14 |  | 311.72 | 0.14 | 361.72 | 0.197 |  |  |  |
| 161.74 | 0.076 | 211.74 | 0.045 | 261.74 | 0.149 |  | 311.74 | 0.137 | 361.74 | 0.217 |  |  |  |
| 161.76 | 0.135 | 211.76 | 0.066 | 261.76 | 0.147 |  | 311.76 | 0.147 | 361.76 | 0.202 |  |  |  |
| 161.78 | 0.18 | 211.78 | 0.129 | 261.78 | 0.143 |  | 311.78 | 0.158 | 361.78 | 0.267 |  |  |  |
| 161.8 | 0.195 | 211.8 | 0.117 | 261.8 | 0.138 |  | 311.8 | 0.18 | 361.8 | 0.309 |  |  |  |
| 161.82 | 0.192 | 211.82 | 0.048 | 261.82 | 0.137 |  | 311.82 | 0.188 | 361.82 | 0.36 |  |  |  |
| 161.84 | 0.165 | 211.84 | 0.024 | 261.84 | 0.146 |  | 311.84 | 0.184 | 361.84 | 0.325 |  |  |  |
| 161.86 | 0.137 | 211.86 | 0.014 | 261.86 | 0.149 |  | 311.86 | 0.217 | 361.86 | 0.287 |  |  |  |
| 161.88 | 0.111 | 211.88 | 0.014 | 261.88 | 0.144 |  | 311.88 | 0.224 | 361.88 | 0.235 |  |  |  |
| 161.9 | 0.093 | 211.9 | 0.015 | 261.9 | 0.141 |  | 311.9 | 0.211 | 361.9 | 0.198 |  |  |  |
| 161.92 | 0.08 | 211.92 | 0.016 | 261.92 | 0.15 |  | 311.92 | 0.188 | 361.92 | 0.176 |  |  |  |
| 161.94 | 0.059 | 211.94 | 0.017 | 261.94 | 0.082 |  | 311.94 | 0.141 | 361.94 | 0.156 |  |  |  |
| 161.96 | 0.052 | 211.96 | 0.011 | 261.96 | 0.08 |  | 311.96 | 0.109 | 361.96 | 0.151 |  |  |  |
| 161.98 | 0.043 | 211.98 | 0.007 | 261.98 | 0.157 |  | 311.98 | 0.094 | 361.98 | 0.156 |  |  |  |
| 162 | 0.035 | 212 | 0.007 | 262 | 0.168 |  | 312 | 0.092 | 362 | 0.148 |  |  |  |
| 162.02 | 0.034 | 212.02 | 0.006 | 262.02 | 0.15 |  | 312.02 | 0.119 | 362.02 | 0.135 |  |  |  |
| 162.04 | 0.038 | 212.04 | 0.005 | 262.04 | 0.141 |  | 312.04 | 0.151 | 362.04 | 0.091 |  |  |  |
| 162.06 | 0.053 | 212.06 | 0.005 | 262.06 | 0.041 |  | 312.06 | 0.169 | 362.06 | 0.073 |  |  |  |
| 162.08 | 0.089 | 212.08 | 0.01 | 262.08 | 0.031 |  | 312.08 | 0.186 | 362.08 | 0.186 |  |  |  |
| 162.1 | 0.104 | 212.1 | 0.014 | 262.1 | 0.055 |  | 312.1 | 0.192 | 362.1 | 0.178 |  |  |  |
| 162.12 | 0.102 | 212.12 | 0.015 | 262.12 | 0.06 |  | 312.12 | 0.206 | 362.12 | 0.158 |  |  |  |
| 162.14 | 0.084 | 212.14 | 0.016 | 262.14 | 0.097 |  | 312.14 | 0.211 | 362.14 | 0.134 |  |  |  |
| 162.16 | 0.06 | 212.16 | 0.02 | 262.16 | 0.101 |  | 312.16 | 0.203 | 362.16 | 0.097 |  |  |  |
| 162.18 | 0.065 | 212.18 | 0.027 | 262.18 | 0.083 |  | 312.18 | 0.195 | 362.18 | 0.076 |  |  |  |
| 162.2 | 0.103 | 212.2 | 0.035 | 262.2 | 0.051 |  | 312.2 | 0.189 | 362.2 | 0.081 |  |  |  |
| 162.22 | 0.12 | 212.22 | 0.036 | 262.22 | 0.049 |  | 312.22 | 0.193 | 362.22 | 0.099 |  |  |  |
| 162.24 | 0.131 | 212.24 | 0.029 | 262.24 | 0.088 |  | 312.24 | 0.211 | 362.24 | 0.113 |  |  |  |
| 162.26 | 0.125 | 212.26 | 0.031 | 262.26 | 0.105 |  | 312.26 | 0.206 | 362.26 | 0.106 |  |  |  |
| 162.28 | 0.137 | 212.28 | 0.037 | 262.28 | 0.123 |  | 312.28 | 0.2 | 362.28 | 0.086 |  |  |  |
| 162.3 | 0.133 | 212.3 | 0.052 | 262.3 | 0.128 |  | 312.3 | 0.195 | 362.3 | 0.164 |  |  |  |
| 162.32 | 0.12 | 212.32 | 0.058 | 262.32 | 0.142 |  | 312.32 | 0.194 | 362.32 | 0.168 |  |  |  |
| 162.34 | 0.141 | 212.34 | 0.061 | 262.34 | 0.154 |  | 312.34 | 0.19 | 362.34 | 0.179 |  |  |  |
| 162.36 | 0.099 | 212.36 | 0.064 | 262.36 | 0.157 |  | 312.36 | 0.185 | 362.36 | 0.203 |  |  |  |
| 162.38 | 0.082 | 212.38 | 0.062 | 262.38 | 0.162 |  | 312.38 | 0.187 | 362.38 | 0.211 |  |  |  |
| 162.4 | 0.064 | 212.4 | 0.066 | 262.4 | 0.158 |  | 312.4 | 0.19 | 362.4 | 0.202 |  |  |  |
| 162.42 | 0.009 | 212.42 | 0.06 | 262.42 | 0.151 |  | 312.42 | 0.181 | 362.42 | 0.188 |  |  |  |
| 162.44 | 0.062 | 212.44 | 0.05 | 262.44 | 0.145 |  | 312.44 | 0.195 | 362.44 | 0.184 |  |  |  |
| 162.46 | 0.071 | 212.46 | 0.04 | 262.46 | 0.163 |  | 312.46 | 0.202 | 362.46 | 0.196 |  |  |  |
| 162.48 | 0.086 | 212.48 | 0.035 | 262.48 | 0.172 |  | 312.48 | 0.215 | 362.48 | 0.206 |  |  |  |
| 162.5 | 0.115 | 212.5 | 0.032 | 262.5 | 0.151 |  | 312.5 | 0.217 | 362.5 | 0.203 |  |  |  |
| 162.52 | 0.16 | 212.52 | 0.028 | 262.52 | 0.114 |  | 312.52 | 0.179 | 362.52 | 0.195 |  |  |  |
| 162.54 | 0.181 | 212.54 | 0.023 | 262.54 | 0.099 |  | 312.54 | 0.172 | 362.54 | 0.194 |  |  |  |
| 162.56 | 0.167 | 212.56 | 0.018 | 262.56 | 0.018 |  | 312.56 | 0.156 | 362.56 | 0.193 |  |  |  |
| 162.58 | 0.112 | 212.58 | 0.016 | 262.58 | 0.062 |  | 312.58 | 0.198 | 362.58 | 0.202 |  |  |  |
| 162.6 | 0.094 | 212.6 | 0.016 | 262.6 | 0.08 |  | 312.6 | 0.183 | 362.6 | 0.204 |  |  |  |
| 162.62 | 0.085 | 212.62 | 0.018 | 262.62 | 0.073 |  | 312.62 | 0.222 | 362.62 | 0.209 |  |  |  |
| 162.64 | 0.083 | 212.64 | 0.027 | 262.64 | 0.061 |  | 312.64 | 0.216 | 362.64 | 0.205 |  |  |  |
| 162.66 | 0.106 | 212.66 | 0.044 | 262.66 | 0.056 |  | 312.66 | 0.224 | 362.66 | 0.198 |  |  |  |
| 162.68 | 0.115 | 212.68 | 0.056 | 262.68 | 0.068 |  | 312.68 | 0.225 | 362.68 | 0.196 |  |  |  |
| 162.7 | 0.102 | 212.7 | 0.019 | 262.7 | 0.05 |  | 312.7 | 0.201 | 362.7 | 0.2 |  |  |  |
| 162.72 | 0.104 | 212.72 | 0.022 | 262.72 | 0.062 |  | 312.72 | 0.191 | 362.72 | 0.209 |  |  |  |
| 162.74 | 0.006 | 212.74 | 0.023 | 262.74 | 0.076 |  | 312.74 | 0.198 | 362.74 | 0.213 |  |  |  |
| 162.76 | -0.008 | 212.76 | 0.026 | 262.76 | 0.099 |  | 312.76 | 0.218 | 362.76 | 0.21 |  |  |  |
| 162.78 | -0.01 | 212.78 | 0.028 | 262.78 | 0.109 |  | 312.78 | 0.222 | 362.78 | 0.193 |  |  |  |
| 162.8 | 0.023 | 212.8 | 0.028 | 262.8 | 0.102 |  | 312.8 | 0.219 | 362.8 | 0.193 |  |  |  |
| 162.82 | 0.047 | 212.82 | 0.013 | 262.82 | 0.091 |  | 312.82 | 0.218 | 362.82 | 0.188 |  |  |  |
| 162.84 | 0.023 | 212.84 | 0.031 | 262.84 | 0.05 |  | 312.84 | 0.214 | 362.84 | 0.187 |  |  |  |
| 162.86 | 0.039 | 212.86 | 0.027 | 262.86 | 0.064 |  | 312.86 | 0.208 | 362.86 | 0.186 |  |  |  |
| 162.88 | 0.043 | 212.88 | 0.004 | 262.88 | 0.067 |  | 312.88 | 0.198 | 362.88 | 0.167 |  |  |  |
| 162.9 | 0.035 | 212.9 | 0.008 | 262.9 | 0.052 |  | 312.9 | 0.159 | 362.9 | 0.154 |  |  |  |
| 162.92 | 0.011 | 212.92 | 0.025 | 262.92 | 0.101 |  | 312.92 | 0.151 | 362.92 | 0.154 |  |  |  |
| 162.94 | -0.011 | 212.94 | 0.045 | 262.94 | 0.178 |  | 312.94 | 0.163 | 362.94 | 0.166 |  |  |  |
| 162.96 | 0.005 | 212.96 | 0.046 | 262.96 | 0.229 |  | 312.96 | 0.168 | 362.96 | 0.186 |  |  |  |
| 162.98 | 0.006 | 212.98 | 0.044 | 262.98 | 0.189 |  | 312.98 | 0.188 | 362.98 | 0.201 |  |  |  |
| 163 | 0.005 | 213 | 0.044 | 263 | 0.163 |  | 313 | 0.2 | 363 | 0.191 |  |  |  |
| 163.02 | 0 | 213.02 | 0.046 | 263.02 | 0.135 |  | 313.02 | 0.197 | 363.02 | 0.171 |  |  |  |
| 163.04 | -0.018 | 213.04 | 0.049 | 263.04 | 0.125 |  | 313.04 | 0.198 | 363.04 | 0.189 |  |  |  |
| 163.06 | -0.007 | 213.06 | 0.05 | 263.06 | 0.108 |  | 313.06 | 0.199 | 363.06 | 0.23 |  |  |  |
| 163.08 | 0.057 | 213.08 | 0.044 | 263.08 | 0.095 |  | 313.08 | 0.202 | 363.08 | 0.245 |  |  |  |
| 163.1 | 0.021 | 213.1 | 0.027 | 263.1 | 0.1 |  | 313.1 | 0.199 | 363.1 | 0.209 |  |  |  |
| 163.12 | 0.055 | 213.12 | 0.021 | 263.12 | 0.113 |  | 313.12 | 0.198 | 363.12 | 0.23 |  |  |  |
| 163.14 | 0.104 | 213.14 | 0.019 | 263.14 | 0.133 |  | 313.14 | 0.201 | 363.14 | 0.235 |  |  |  |
| 163.16 | 0.189 | 213.16 | 0.02 | 263.16 | 0.133 |  | 313.16 | 0.201 | 363.16 | 0.193 |  |  |  |
| 163.18 | 0.244 | 213.18 | 0.016 | 263.18 | 0.141 |  | 313.18 | 0.195 | 363.18 | 0.165 |  |  |  |
| 163.2 | 0.22 | 213.2 | 0.014 | 263.2 | 0.148 |  | 313.2 | 0.217 | 363.2 | 0.163 |  |  |  |
| 163.22 | 0.15 | 213.22 | 0.013 | 263.22 | 0.148 |  | 313.22 | 0.22 | 363.22 | 0.164 |  |  |  |
| 163.24 | 0.161 | 213.24 | 0.011 | 263.24 | 0.141 |  | 313.24 | 0.24 | 363.24 | 0.172 |  |  |  |
| 163.26 | 0.169 | 213.26 | 0.011 | 263.26 | 0.143 |  | 313.26 | 0.239 | 363.26 | 0.153 |  |  |  |
| 163.28 | 0.144 | 213.28 | 0.011 | 263.28 | 0.146 |  | 313.28 | 0.233 | 363.28 | 0.176 |  |  |  |
| 163.3 | 0.123 | 213.3 | 0.012 | 263.3 | 0.158 |  | 313.3 | 0.214 | 363.3 | 0.201 |  |  |  |
| 163.32 | 0.123 | 213.32 | 0.007 | 263.32 | 0.18 |  | 313.32 | 0.164 | 363.32 | 0.207 |  |  |  |
| 163.34 | 0.153 | 213.34 | 0.005 | 263.34 | 0.182 |  | 313.34 | 0.146 | 363.34 | 0.204 |  |  |  |
| 163.36 | 0.152 | 213.36 | 0.007 | 263.36 | 0.011 |  | 313.36 | 0.135 | 363.36 | 0.201 |  |  |  |
| 163.38 | 0.142 | 213.38 | 0.015 | 263.38 | -0.005 |  | 313.38 | 0.114 | 363.38 | 0.187 |  |  |  |
| 163.4 | 0.133 | 213.4 | 0.02 | 263.4 | 0.019 |  | 313.4 | 0.118 | 363.4 | 0.18 |  |  |  |
| 163.42 | 0.125 | 213.42 | 0.026 | 263.42 | 0.038 |  | 313.42 | 0.071 | 363.42 | 0.182 |  |  |  |
| 163.44 | 0.117 | 213.44 | 0.03 | 263.44 | 0.048 |  | 313.44 | 0.099 | 363.44 | 0.193 |  |  |  |
| 163.46 | 0.11 | 213.46 | 0.024 | 263.46 | 0.048 |  | 313.46 | 0.159 | 363.46 | 0.194 |  |  |  |
| 163.48 | 0.125 | 213.48 | 0.025 | 263.48 | 0.039 |  | 313.48 | 0.205 | 363.48 | 0.206 |  |  |  |
| 163.5 | 0.129 | 213.5 | 0.083 | 263.5 | 0.034 |  | 313.5 | 0.207 | 363.5 | 0.214 |  |  |  |
| 163.52 | 0.103 | 213.52 | 0.115 | 263.52 | 0.034 |  | 313.52 | 0.199 | 363.52 | 0.215 |  |  |  |
| 163.54 | 0.006 | 213.54 | 0.126 | 263.54 | 0.038 |  | 313.54 | 0.198 | 363.54 | 0.202 |  |  |  |
| 163.56 | 0.01 | 213.56 | 0.13 | 263.56 | 0.049 |  | 313.56 | 0.203 | 363.56 | 0.185 |  |  |  |
| 163.58 | 0.04 | 213.58 | 0.143 | 263.58 | 0.059 |  | 313.58 | 0.203 | 363.58 | 0.167 |  |  |  |
| 163.6 | 0.056 | 213.6 | 0.164 | 263.6 | 0.075 |  | 313.6 | 0.207 | 363.6 | 0.179 |  |  |  |
| 163.62 | 0.058 | 213.62 | 0.009 | 263.62 | 0.085 |  | 313.62 | 0.207 | 363.62 | 0.191 |  |  |  |
| 163.64 | 0.029 | 213.64 | 0.012 | 263.64 | 0.166 |  | 313.64 | 0.207 | 363.64 | 0.199 |  |  |  |
| 163.66 | 0.052 | 213.66 | 0.022 | 263.66 | 0.009 |  | 313.66 | 0.209 | 363.66 | 0.188 |  |  |  |
| 163.68 | 0.057 | 213.68 | 0.03 | 263.68 | 0.029 |  | 313.68 | 0.205 | 363.68 | 0.189 |  |  |  |
| 163.7 | 0.081 | 213.7 | 0.03 | 263.7 | 0.054 |  | 313.7 | 0.206 | 363.7 | 0.195 |  |  |  |
| 163.72 | 0.105 | 213.72 | 0.026 | 263.72 | 0.053 |  | 313.72 | 0.18 | 363.72 | 0.201 |  |  |  |
| 163.74 | 0.11 | 213.74 | 0.03 | 263.74 | 0.038 |  | 313.74 | 0.172 | 363.74 | 0.198 |  |  |  |
| 163.76 | 0.114 | 213.76 | 0.025 | 263.76 | 0.034 |  | 313.76 | 0.179 | 363.76 | 0.192 |  |  |  |
| 163.78 | 0.104 | 213.78 | 0.02 | 263.78 | 0.037 |  | 313.78 | 0.195 | 363.78 | 0.153 |  |  |  |
| 163.8 | 0.105 | 213.8 | 0.02 | 263.8 | 0.04 |  | 313.8 | 0.199 | 363.8 | 0.16 |  |  |  |
| 163.82 | 0.122 | 213.82 | 0.028 | 263.82 | 0.034 |  | 313.82 | 0.203 | 363.82 | 0.204 |  |  |  |
| 163.84 | 0.135 | 213.84 | 0.058 | 263.84 | 0.036 |  | 313.84 | 0.201 | 363.84 | 0.216 |  |  |  |
| 163.86 | 0.145 | 213.86 | 0.077 | 263.86 | 0.039 |  | 313.86 | 0.193 | 363.86 | 0.231 |  |  |  |
| 163.88 | 0.106 | 213.88 | 0.103 | 263.88 | 0.027 |  | 313.88 | 0.178 | 363.88 | 0.256 |  |  |  |
| 163.9 | 0.087 | 213.9 | 0.091 | 263.9 | 0.012 |  | 313.9 | 0.158 | 363.9 | 0.257 |  |  |  |
| 163.92 | 0.103 | 213.92 | 0.082 | 263.92 | 0.018 |  | 313.92 | 0.192 | 363.92 | 0.369 |  |  |  |
| 163.94 | 0.029 | 213.94 | 0.066 | 263.94 | 0.021 |  | 313.94 | 0.194 | 363.94 | 0.3012 |  |  |  |
| 163.96 | 0.033 | 213.96 | 0.065 | 263.96 | 0.02 |  | 313.96 | 0.194 | 363.96 | 0.288 |  |  |  |
| 163.98 | 0.037 | 213.98 | 0.087 | 263.98 | 0.043 |  | 313.98 | 0.195 | 363.98 | 0.393 |  |  |  |
| 164 | 0.048 | 214 | 0.112 | 264 | 0.094 |  | 314 | 0.214 | 364 | 0.315 |  |  |  |
| 164.02 | 0.046 | 214.02 | 0.115 | 264.02 | 0.137 |  | 314.02 | 0.214 | 364.02 | 0.233 |  |  |  |
| 164.04 | 0.039 | 214.04 | 0.116 | 264.04 | 0.145 |  | 314.04 | 0.214 | 364.04 | 0.206 |  |  |  |
| 164.06 | 0.025 | 214.06 | 0.127 | 264.06 | 0.146 |  | 314.06 | 0.203 | 364.06 | 0.21 |  |  |  |
| 164.08 | 0.015 | 214.08 | 0.14 | 264.08 | 0.129 |  | 314.08 | 0.201 | 364.08 | 0.209 |  |  |  |
| 164.1 | 0.005 | 214.1 | 0.151 | 264.1 | 0.121 |  | 314.1 | 0.21 | 364.1 | 0.129 |  |  |  |
| 164.12 | -0.01 | 214.12 | 0.121 | 264.12 | 0.117 |  | 314.12 | 0.168 | 364.12 | 0.182 |  |  |  |
| 164.14 | -0.022 | 214.14 | 0.103 | 264.14 | 0.119 |  | 314.14 | 0.164 | 364.14 | 0.188 |  |  |  |
| 164.16 | -0.024 | 214.16 | 0.101 | 264.16 | 0.121 |  | 314.16 | 0.172 | 364.16 | 0.192 |  |  |  |
| 164.18 | -0.015 | 214.18 | 0.1 | 264.18 | 0.124 |  | 314.18 | 0.174 | 364.18 | 0.191 |  |  |  |
| 164.2 | -0.008 | 214.2 | 0.1 | 264.2 | 0.16 |  | 314.2 | 0.182 | 364.2 | 0.192 |  |  |  |
| 164.22 | 0.018 | 214.22 | 0.098 | 264.22 | 0.202 |  | 314.22 | 0.188 | 364.22 | 0.181 |  |  |  |
| 164.24 | 0.039 | 214.24 | 0.104 | 264.24 | 0.157 |  | 314.24 | 0.191 | 364.24 | 0.189 |  |  |  |
| 164.26 | 0.064 | 214.26 | 0.111 | 264.26 | 0.169 |  | 314.26 | 0.194 | 364.26 | 0.189 |  |  |  |
| 164.28 | 0.08 | 214.28 | 0.145 | 264.28 | 0.176 |  | 314.28 | 0.19 | 364.28 | 0.208 |  |  |  |
| 164.3 | 0.104 | 214.3 | 0.045 | 264.3 | 0.17 |  | 314.3 | 0.192 | 364.3 | 0.207 |  |  |  |
| 164.32 | 0.108 | 214.32 | 0.043 | 264.32 | 0.16 |  | 314.32 | 0.191 | 364.32 | 0.208 |  |  |  |
| 164.34 | 0.134 | 214.34 | 0.039 | 264.34 | 0.157 |  | 314.34 | 0.185 | 364.34 | 0.21 |  |  |  |
| 164.36 | 0.14 | 214.36 | 0.051 | 264.36 | 0.143 |  | 314.36 | 0.183 | 364.36 | 0.184 |  |  |  |
| 164.38 | 0.131 | 214.38 | 0.043 | 264.38 | 0.123 |  | 314.38 | 0.192 | 364.38 | 0.158 |  |  |  |
| 164.4 | 0.121 | 214.4 | 0.059 | 264.4 | 0.15 |  | 314.4 | 0.201 | 364.4 | 0.161 |  |  |  |
| 164.42 | 0.11 | 214.42 | 0.069 | 264.42 | 0.179 |  | 314.42 | 0.194 | 364.42 | 0.201 |  |  |  |
| 164.44 | 0.093 | 214.44 | 0.07 | 264.44 | 0.176 |  | 314.44 | 0.188 | 364.44 | 0.198 |  |  |  |
| 164.46 | 0.102 | 214.46 | 0.089 | 264.46 | 0.207 |  | 314.46 | 0.195 | 364.46 | 0.172 |  |  |  |
| 164.48 | 0.095 | 214.48 | 0.102 | 264.48 | 0.201 |  | 314.48 | 0.205 | 364.48 | 0.174 |  |  |  |
| 164.5 | 0.09 | 214.5 | 0.119 | 264.5 | 0.206 |  | 314.5 | 0.201 | 364.5 | 0.191 |  |  |  |
| 164.52 | 0.08 | 214.52 | 0.114 | 264.52 | 0.198 |  | 314.52 | 0.194 | 364.52 | 0.206 |  |  |  |
| 164.54 | 0.09 | 214.54 | 0.093 | 264.54 | 0.009 |  | 314.54 | 0.194 | 364.54 | 0.198 |  |  |  |
| 164.56 | 0.114 | 214.56 | 0.072 | 264.56 | 0.006 |  | 314.56 | 0.185 | 364.56 | 0.193 |  |  |  |
| 164.58 | 0.173 | 214.58 | 0.056 | 264.58 | 0.012 |  | 314.58 | 0.167 | 364.58 | 0.183 |  |  |  |
| 164.6 | 0.19 | 214.6 | 0.069 | 264.6 | 0.035 |  | 314.6 | 0.191 | 364.6 | 0.172 |  |  |  |
| 164.62 | 0.179 | 214.62 | 0.079 | 264.62 | 0.063 |  | 314.62 | 0.202 | 364.62 | 0.165 |  |  |  |
| 164.64 | 0.13 | 214.64 | 0.075 | 264.64 | 0.027 |  | 314.64 | 0.191 | 364.64 | 0.165 |  |  |  |
| 164.66 | 0.092 | 214.66 | 0.075 | 264.66 | 0 |  | 314.66 | 0.215 | 364.66 | 0.165 |  |  |  |
| 164.68 | 0.063 | 214.68 | 0.096 | 264.68 | 0.021 |  | 314.68 | 0.225 | 364.68 | 0.171 |  |  |  |
| 164.7 | 0.106 | 214.7 | 0.007 | 264.7 | 0.057 |  | 314.7 | 0.23 | 364.7 | 0.174 |  |  |  |
| 164.72 | 0.132 | 214.72 | 0.013 | 264.72 | 0.089 |  | 314.72 | 0.222 | 364.72 | 0.186 |  |  |  |
| 164.74 | 0.136 | 214.74 | 0 | 264.74 | 0.139 |  | 314.74 | 0.159 | 364.74 | 0.108 |  |  |  |
| 164.76 | 0.154 | 214.76 | -0.02 | 264.76 | 0.168 |  | 314.76 | 0.221 | 364.76 | 0.125 |  |  |  |
| 164.78 | 0.033 | 214.78 | -0.04 | 264.78 | 0.179 |  | 314.78 | 0.223 | 364.78 | 0.183 |  |  |  |
| 164.8 | 0.085 | 214.8 | -0.038 | 264.8 | 0.203 |  | 314.8 | 0.213 | 364.8 | 0.197 |  |  |  |
| 164.82 | 0.117 | 214.82 | 0.035 | 264.82 | 0.218 |  | 314.82 | 0.225 | 364.82 | 0.193 |  |  |  |
| 164.84 | 0.093 | 214.84 | 0.015 | 264.84 | 0.221 |  | 314.84 | 0.216 | 364.84 | 0.185 |  |  |  |
| 164.86 | 0.07 | 214.86 | 0.017 | 264.86 | 0.213 |  | 314.86 | 0.197 | 364.86 | 0.167 |  |  |  |
| 164.88 | 0.094 | 214.88 | 0.036 | 264.88 | 0.191 |  | 314.88 | 0.208 | 364.88 | 0.146 |  |  |  |
| 164.9 | 0.101 | 214.9 | 0.043 | 264.9 | 0.173 |  | 314.9 | 0.221 | 364.9 | 0.127 |  |  |  |
| 164.92 | 0.101 | 214.92 | 0.05 | 264.92 | 0.161 |  | 314.92 | 0.213 | 364.92 | 0.127 |  |  |  |
| 164.94 | 0.096 | 214.94 | 0.065 | 264.94 | -0.059 |  | 314.94 | 0.196 | 364.94 | 0.141 |  |  |  |
| 164.96 | 0.102 | 214.96 | 0.085 | 264.96 | 0.035 |  | 314.96 | 0.2 | 364.96 | 0.155 |  |  |  |
| 164.98 | 0.09 | 214.98 | 0.1 | 264.98 | 0.119 |  | 314.98 | 0.194 | 364.98 | 0.167 |  |  |  |
| 165 | 0.038 | 215 | 0.107 | 265 | 0.168 |  | 315 | 0.185 | 365 | 0.183 |  |  |  |
| 165.02 | 0.051 | 215.02 | 0.11 | 265.02 | 0.177 |  | 315.02 | 0.188 | 365.02 | 0.177 |  |  |  |
| 165.04 | 0.067 | 215.04 | 0.189 | 265.04 | 0.164 |  | 315.04 | 0.189 | 365.04 | 0.188 |  |  |  |
| 165.06 | 0.094 | 215.06 | 0.023 | 265.06 | 0.134 |  | 315.06 | 0.187 | 365.06 | 0.2 |  |  |  |
| 165.08 | 0.124 | 215.08 | 0.062 | 265.08 | 0.106 |  | 315.08 | 0.163 | 365.08 | 0.21 |  |  |  |
| 165.1 | 0.109 | 215.1 | 0.129 | 265.1 | 0.116 |  | 315.1 | 0.136 | 365.1 | 0.214 |  |  |  |
| 165.12 | 0.108 | 215.12 | 0.148 | 265.12 | 0.123 |  | 315.12 | 0.132 | 365.12 | 0.209 |  |  |  |
| 165.14 | 0.082 | 215.14 | 0.141 | 265.14 | 0.108 |  | 315.14 | 0.139 | 365.14 | 0.211 |  |  |  |
| 165.16 | 0.083 | 215.16 | 0.138 | 265.16 | 0.086 |  | 315.16 | 0.146 | 365.16 | 0.222 |  |  |  |
| 165.18 | 0.076 | 215.18 | 0.142 | 265.18 | 0.097 |  | 315.18 | 0.137 | 365.18 | 0.196 |  |  |  |
| 165.2 | 0.08 | 215.2 | 0.156 | 265.2 | 0.122 |  | 315.2 | 0.135 | 365.2 | 0.189 |  |  |  |
| 165.22 | 0.084 | 215.22 | 0.162 | 265.22 | 0.154 |  | 315.22 | 0.136 | 365.22 | 0.189 |  |  |  |
| 165.24 | 0.101 | 215.24 | 0.169 | 265.24 | 0.186 |  | 315.24 | 0.134 | 365.24 | 0.199 |  |  |  |
| 165.26 | 0.104 | 215.26 | 0.172 | 265.26 | 0.075 |  | 315.26 | 0.099 | 365.26 | 0.204 |  |  |  |
| 165.28 | 0.102 | 215.28 | 0.135 | 265.28 | 0.139 |  | 315.28 | 0.08 | 365.28 | 0.21 |  |  |  |
| 165.3 | 0.103 | 215.3 | 0.101 | 265.3 | 0.166 |  | 315.3 | 0.189 | 365.3 | 0.215 |  |  |  |
| 165.32 | 0.098 | 215.32 | 0.081 | 265.32 | 0.14 |  | 315.32 | 0.128 | 365.32 | 0.208 |  |  |  |
| 165.34 | 0.094 | 215.34 | 0.07 | 265.34 | 0.08 |  | 315.34 | 0.127 | 365.34 | 0.199 |  |  |  |
| 165.36 | 0.106 | 215.36 | 0.062 | 265.36 | 0.041 |  | 315.36 | 0.119 | 365.36 | 0.206 |  |  |  |
| 165.38 | 0.118 | 215.38 | 0.057 | 265.38 | 0.038 |  | 315.38 | 0.118 | 365.38 | 0.213 |  |  |  |
| 165.4 | 0.149 | 215.4 | 0.05 | 265.4 | 0.038 |  | 315.4 | 0.121 | 365.4 | 0.224 |  |  |  |
| 165.42 | 0.148 | 215.42 | 0.04 | 265.42 | 0.047 |  | 315.42 | 0.122 | 365.42 | 0.201 |  |  |  |
| 165.44 | 0.153 | 215.44 | 0.034 | 265.44 | 0.071 |  | 315.44 | 0.135 | 365.44 | 0.2 |  |  |  |
| 165.46 | 0.181 | 215.46 | 0.031 | 265.46 | 0.12 |  | 315.46 | 0.14 | 365.46 | 0.179 |  |  |  |
| 165.48 | 0.213 | 215.48 | 0.031 | 265.48 | 0.169 |  | 315.48 | 0.143 | 365.48 | 0.189 |  |  |  |
| 165.5 | 0.223 | 215.5 | 0.039 | 265.5 | 0.192 |  | 315.5 | 0.14 | 365.5 | 0.208 |  |  |  |
| 165.52 | 0.2 | 215.52 | 0.033 | 265.52 | 0.202 |  | 315.52 | 0.131 | 365.52 | 0.209 |  |  |  |
| 165.54 | 0.01 | 215.54 | 0.024 | 265.54 | 0.193 |  | 315.54 | 0.138 | 365.54 | 0.218 |  |  |  |
| 165.56 | 0.013 | 215.56 | 0.012 | 265.56 | 0.157 |  | 315.56 | 0.169 | 365.56 | 0.201 |  |  |  |
| 165.58 | 0.01 | 215.58 | 0.019 | 265.58 | 0.175 |  | 315.58 | 0.178 | 365.58 | 0.195 |  |  |  |
| 165.6 | 0.011 | 215.6 | 0.023 | 265.6 | 0.199 |  | 315.6 | 0.155 | 365.6 | 0.2 |  |  |  |
| 165.62 | -0.01 | 215.62 | 0.023 | 265.62 | 0.214 |  | 315.62 | 0.13 | 365.62 | 0.219 |  |  |  |
| 165.64 | -0.017 | 215.64 | 0.024 | 265.64 | 0.011 |  | 315.64 | 0.138 | 365.64 | 0.218 |  |  |  |
| 165.66 | -0.028 | 215.66 | 0.022 | 265.66 | 0.001 |  | 315.66 | 0.172 | 365.66 | 0.213 |  |  |  |
| 165.68 | -0.014 | 215.68 | 0.024 | 265.68 | 0.029 |  | 315.68 | 0.205 | 365.68 | 0.209 |  |  |  |
| 165.7 | 0 | 215.7 | 0.028 | 265.7 | 0.016 |  | 315.7 | 0.207 | 365.7 | 0.2 |  |  |  |
| 165.72 | 0.015 | 215.72 | 0.036 | 265.72 | 0.042 |  | 315.72 | 0.182 | 365.72 | 0.189 |  |  |  |
| 165.74 | 0.019 | 215.74 | 0.041 | 265.74 | 0.042 |  | 315.74 | 0.171 | 365.74 | 0.186 |  |  |  |
| 165.76 | 0.036 | 215.76 | 0.042 | 265.76 | 0.061 |  | 315.76 | 0.124 | 365.76 | 0.191 |  |  |  |
| 165.78 | 0.052 | 215.78 | 0.038 | 265.78 | 0.07 |  | 315.78 | 0.137 | 365.78 | 0.184 |  |  |  |
| 165.8 | 0.07 | 215.8 | 0.036 | 265.8 | 0.077 |  | 315.8 | 0.131 | 365.8 | 0.196 |  |  |  |
| 165.82 | 0.092 | 215.82 | 0.035 | 265.82 | 0.089 |  | 315.82 | 0.116 | 365.82 | 0.199 |  |  |  |
| 165.84 | 0.105 | 215.84 | 0.045 | 265.84 | 0.086 |  | 315.84 | 0.098 | 365.84 | 0.22 |  |  |  |
| 165.86 | 0.117 | 215.86 | 0.046 | 265.86 | 0.09 |  | 315.86 | 0.085 | 365.86 | 0.235 |  |  |  |
| 165.88 | 0.137 | 215.88 | 0.023 | 265.88 | 0.112 |  | 315.88 | 0.076 | 365.88 | 0.234 |  |  |  |
| 165.9 | 0.147 | 215.9 | 0.042 | 265.9 | 0.12 |  | 315.9 | 0.059 | 365.9 | 0.233 |  |  |  |
| 165.92 | 0.157 | 215.92 | -0.008 | 265.92 | 0.129 |  | 315.92 | 0.051 | 365.92 | 0.237 |  |  |  |
| 165.94 | 0.158 | 215.94 | 0.006 | 265.94 | 0.165 |  | 315.94 | 0.063 | 365.94 | 0.223 |  |  |  |
| 165.96 | 0.14 | 215.96 | 0.016 | 265.96 | 0.181 |  | 315.96 | 0.114 | 365.96 | 0.214 |  |  |  |
| 165.98 | 0.124 | 215.98 | 0.02 | 265.98 | 0.191 |  | 315.98 | 0.114 | 365.98 | 0.222 |  |  |  |
| 166 | 0.114 | 216 | 0.032 | 266 | 0.192 |  | 316 | 0.109 | 366 | 0.228 |  |  |  |
| 166.02 | 0.122 | 216.02 | 0.048 | 266.02 | 0.119 |  | 316.02 | 0.106 | 366.02 | 0.233 |  |  |  |
| 166.04 | 0.121 | 216.04 | 0.06 | 266.04 | 0.145 |  | 316.04 | 0.13 | 366.04 | 0.238 |  |  |  |
| 166.06 | 0.091 | 216.06 | 0.064 | 266.06 | 0.17 |  | 316.06 | 0.164 | 366.06 | 0.234 |  |  |  |
| 166.08 | 0.064 | 216.08 | 0.05 | 266.08 | 0.155 |  | 316.08 | 0.174 | 366.08 | 0.214 |  |  |  |
| 166.1 | 0.127 | 216.1 | 0.035 | 266.1 | 0.135 |  | 316.1 | 0.173 | 366.1 | 0.205 |  |  |  |
| 166.12 | 0.208 | 216.12 | 0.022 | 266.12 | 0.14 |  | 316.12 | 0.181 | 366.12 | 0.214 |  |  |  |
| 166.14 | 0.229 | 216.14 | 0.016 | 266.14 | 0.144 |  | 316.14 | 0.199 | 366.14 | 0.224 |  |  |  |
| 166.16 | 0.215 | 216.16 | 0.008 | 266.16 | 0.158 |  | 316.16 | 0.191 | 366.16 | 0.225 |  |  |  |
| 166.18 | 0.188 | 216.18 | 0.003 | 266.18 | 0.176 |  | 316.18 | 0.01 | 366.18 | 0.21 |  |  |  |
| 166.2 | 0.153 | 216.2 | -0.001 | 266.2 | 0.19 |  | 316.2 | 0.01 | 366.2 | 0.213 |  |  |  |
| 166.22 | 0.13 | 216.22 | 0.01 | 266.22 | 0.213 |  | 316.22 | 0.011 | 366.22 | 0.221 |  |  |  |
| 166.24 | 0.115 | 216.24 | 0.025 | 266.24 | 0.2 |  | 316.24 | 0.026 | 366.24 | 0.225 |  |  |  |
| 166.26 | 0.105 | 216.26 | 0.027 | 266.26 | 0.208 |  | 316.26 | 0.034 | 366.26 | 0.24 |  |  |  |
| 166.28 | 0.099 | 216.28 | 0.022 | 266.28 | 0.247 |  | 316.28 | 0.046 | 366.28 | 0.239 |  |  |  |
| 166.3 | 0.099 | 216.3 | 0.032 | 266.3 | 0.198 |  | 316.3 | 0.066 | 366.3 | 0.24 |  |  |  |
| 166.32 | 0.103 | 216.32 | 0.034 | 266.32 | 0.095 |  | 316.32 | 0.071 | 366.32 | 0.23 |  |  |  |
| 166.34 | 0.103 | 216.34 | 0.036 | 266.34 | 0.124 |  | 316.34 | 0.082 | 366.34 | 0.212 |  |  |  |
| 166.36 | 0.068 | 216.36 | 0.042 | 266.36 | 0.12 |  | 316.36 | 0.093 | 366.36 | 0.169 |  |  |  |
| 166.38 | 0.134 | 216.38 | 0.038 | 266.38 | 0.181 |  | 316.38 | 0.104 | 366.38 | 0.183 |  |  |  |
| 166.4 | 0.133 | 216.4 | 0.029 | 266.4 | 0.146 |  | 316.4 | 0.113 | 366.4 | 0.218 |  |  |  |
| 166.42 | 0.146 | 216.42 | 0.034 | 266.42 | 0.148 |  | 316.42 | 0.13 | 366.42 | 0.231 |  |  |  |
| 166.44 | 0.152 | 216.44 | 0.036 | 266.44 | 0.166 |  | 316.44 | 0.141 | 366.44 | 0.235 |  |  |  |
| 166.46 | 0.152 | 216.46 | 0.041 | 266.46 | 0.185 |  | 316.46 | 0.147 | 366.46 | 0.22 |  |  |  |
| 166.48 | 0.157 | 216.48 | 0.035 | 266.48 | 0.212 |  | 316.48 | 0.152 | 366.48 | 0.214 |  |  |  |
| 166.5 | 0.152 | 216.5 | 0.037 | 266.5 | 0.233 |  | 316.5 | 0.145 | 366.5 | 0.162 |  |  |  |
| 166.52 | 0.146 | 216.52 | 0.071 | 266.52 | 0.248 |  | 316.52 | 0.143 | 366.52 | 0.175 |  |  |  |
| 166.54 | 0.128 | 216.54 | 0.098 | 266.54 | 0.241 |  | 316.54 | 0.151 | 366.54 | 0.229 |  |  |  |
| 166.56 | 0.154 | 216.56 | 0.117 | 266.56 | 0.214 |  | 316.56 | 0.156 | 366.56 | 0.223 |  |  |  |
| 166.58 | 0.187 | 216.58 | 0.126 | 266.58 | 0.189 |  | 316.58 | 0.164 | 366.58 | 0.162 |  |  |  |
| 166.6 | 0.17 | 216.6 | 0.128 | 266.6 | 0.15 |  | 316.6 | 0.172 | 366.6 | 0.134 |  |  |  |
| 166.62 | 0.142 | 216.62 | 0.122 | 266.62 | 0.011 |  | 316.62 | 0.189 | 366.62 | 0.155 |  |  |  |
| 166.64 | 0.131 | 216.64 | 0.088 | 266.64 | 0.039 |  | 316.64 | 0.189 | 366.64 | 0.182 |  |  |  |
| 166.66 | 0.122 | 216.66 | 0.083 | 266.66 | 0.067 |  | 316.66 | 0.191 | 366.66 | 0.155 |  |  |  |
| 166.68 | 0.123 | 216.68 | 0.075 | 266.68 | 0.077 |  | 316.68 | 0.192 | 366.68 | 0.208 |  |  |  |
| 166.7 | 0.108 | 216.7 | 0.069 | 266.7 | 0.065 |  | 316.7 | 0.193 | 366.7 | 0.213 |  |  |  |
| 166.72 | 0.097 | 216.72 | 0.059 | 266.72 | 0.079 |  | 316.72 | 0.182 | 366.72 | 0.216 |  |  |  |
| 166.74 | 0.081 | 216.74 | 0.058 | 266.74 | 0.079 |  | 316.74 | 0.179 | 366.74 | 0.206 |  |  |  |
| 166.76 | 0.007 | 216.76 | 0.058 | 266.76 | 0.069 |  | 316.76 | 0.185 | 366.76 | 0.199 |  |  |  |
| 166.78 | 0.008 | 216.78 | 0.055 | 266.78 | 0.056 |  | 316.78 | 0.18 | 366.78 | 0.177 |  |  |  |
| 166.8 | 0.002 | 216.8 | 0.054 | 266.8 | 0.074 |  | 316.8 | 0.176 | 366.8 | 0.157 |  |  |  |
| 166.82 | 0.005 | 216.82 | 0.052 | 266.82 | 0.106 |  | 316.82 | 0.166 | 366.82 | 0.142 |  |  |  |
| 166.84 | 0.013 | 216.84 | 0.049 | 266.84 | 0.127 |  | 316.84 | 0.188 | 366.84 | 0.133 |  |  |  |
| 166.86 | 0.029 | 216.86 | 0.048 | 266.86 | 0.127 |  | 316.86 | 0.217 | 366.86 | 0.132 |  |  |  |
| 166.88 | 0.038 | 216.88 | 0.046 | 266.88 | 0.123 |  | 316.88 | 0.223 | 366.88 | 0.133 |  |  |  |
| 166.9 | 0.044 | 216.9 | 0.043 | 266.9 | 0.142 |  | 316.9 | 0.219 | 366.9 | 0.152 |  |  |  |
| 166.92 | 0.046 | 216.92 | 0.041 | 266.92 | 0.182 |  | 316.92 | 0.214 | 366.92 | 0.157 |  |  |  |
| 166.94 | 0.045 | 216.94 | 0.038 | 266.94 | 0.229 |  | 316.94 | 0.21 | 366.94 | 0.151 |  |  |  |
| 166.96 | 0.054 | 216.96 | 0.034 | 266.96 | 0.3 |  | 316.96 | 0.219 | 366.96 | 0.149 |  |  |  |
| 166.98 | 0.071 | 216.98 | 0.017 | 266.98 | 0.334 |  | 316.98 | 0.228 | 366.98 | 0.159 |  |  |  |
| 167 | 0.085 | 217 | 0.019 | 267 | 0.286 |  | 317 | 0.21 | 367 | 0.155 |  |  |  |
| 167.02 | 0.098 | 217.02 | 0.024 | 267.02 | 0.019 |  | 317.02 | 0.166 | 367.02 | 0.167 |  |  |  |
| 167.04 | 0.113 | 217.04 | 0.025 | 267.04 | 0.015 |  | 317.04 | 0.154 | 367.04 | 0.18 |  |  |  |
| 167.06 | 0.112 | 217.06 | 0.024 | 267.06 | 0.017 |  | 317.06 | 0.088 | 367.06 | 0.186 |  |  |  |
| 167.08 | 0.094 | 217.08 | 0.023 | 267.08 | 0.047 |  | 317.08 | 0.099 | 367.08 | 0.185 |  |  |  |
| 167.1 | 0.086 | 217.1 | 0.007 | 267.1 | 0.068 |  | 317.1 | 0.101 | 367.1 | 0.185 |  |  |  |
| 167.12 | 0.09 | 217.12 | 0.011 | 267.12 | 0.058 |  | 317.12 | 0.104 | 367.12 | 0.184 |  |  |  |
| 167.14 | 0.097 | 217.14 | 0.013 | 267.14 | 0.047 |  | 317.14 | 0.112 | 367.14 | 0.187 |  |  |  |
| 167.16 | 0.165 | 217.16 | 0.012 | 267.16 | 0.049 |  | 317.16 | 0.13 | 367.16 | 0.192 |  |  |  |
| 167.18 | 0.181 | 217.18 | 0.012 | 267.18 | 0.039 |  | 317.18 | 0.138 | 367.18 | 0.196 |  |  |  |
| 167.2 | 0.184 | 217.2 | 0.011 | 267.2 | 0.045 |  | 317.2 | 0.132 | 367.2 | 0.195 |  |  |  |
| 167.22 | 0.164 | 217.22 | 0.011 | 267.22 | 0.054 |  | 317.22 | 0.127 | 367.22 | 0.19 |  |  |  |
| 167.24 | 0.128 | 217.24 | 0.014 | 267.24 | 0.068 |  | 317.24 | 0.133 | 367.24 | 0.197 |  |  |  |
| 167.26 | 0.121 | 217.26 | 0.012 | 267.26 | 0.07 |  | 317.26 | 0.134 | 367.26 | 0.211 |  |  |  |
| 167.28 | 0.122 | 217.28 | 0.012 | 267.28 | 0.072 |  | 317.28 | 0.152 | 367.28 | 0.214 |  |  |  |
| 167.3 | 0.123 | 217.3 | 0.015 | 267.3 | 0.084 |  | 317.3 | 0.16 | 367.3 | 0.212 |  |  |  |
| 167.32 | 0.097 | 217.32 | 0.018 | 267.32 | 0.112 |  | 317.32 | 0.163 | 367.32 | 0.214 |  |  |  |
| 167.34 | 0.121 | 217.34 | 0.02 | 267.34 | 0.142 |  | 317.34 | 0.139 | 367.34 | 0.213 |  |  |  |
| 167.36 | 0.199 | 217.36 | 0.022 | 267.36 | 0.176 |  | 317.36 | 0.121 | 367.36 | 0.207 |  |  |  |
| 167.38 | 0.168 | 217.38 | 0.022 | 267.38 | 0.184 |  | 317.38 | 0.111 | 367.38 | 0.203 |  |  |  |
| 167.4 | 0.104 | 217.4 | 0.017 | 267.4 | 0.185 |  | 317.4 | 0.127 | 367.4 | 0.19 |  |  |  |
| 167.42 | 0.087 | 217.42 | 0.015 | 267.42 | 0.242 |  | 317.42 | 0.149 | 367.42 | 0.193 |  |  |  |
| 167.44 | 0.09 | 217.44 | 0.019 | 267.44 | 0.264 |  | 317.44 | 0.182 | 367.44 | 0.219 |  |  |  |
| 167.46 | 0.109 | 217.46 | 0.018 | 267.46 | 0.26 |  | 317.46 | 0.207 | 367.46 | 0.212 |  |  |  |
| 167.48 | 0.118 | 217.48 | 0.013 | 267.48 | 0.256 |  | 317.48 | 0.208 | 367.48 | 0.218 |  |  |  |
| 167.5 | 0.118 | 217.5 | 0.031 | 267.5 | 0.247 |  | 317.5 | 0.209 | 367.5 | 0.228 |  |  |  |
| 167.52 | 0.098 | 217.52 | 0.039 | 267.52 | 0.227 |  | 317.52 | 0.132 | 367.52 | 0.222 |  |  |  |
| 167.54 | 0.091 | 217.54 | 0.057 | 267.54 | 0.211 |  | 317.54 | 0.092 | 367.54 | 0.221 |  |  |  |
| 167.56 | 0.083 | 217.56 | 0.067 | 267.56 | 0.168 |  | 317.56 | 0.119 | 367.56 | 0.216 |  |  |  |
| 167.58 | 0.13 | 217.58 | 0.063 | 267.58 | 0.089 |  | 317.58 | 0.124 | 367.58 | 0.213 |  |  |  |
| 167.6 | 0.093 | 217.6 | 0.055 | 267.6 | 0.094 |  | 317.6 | 0.136 | 367.6 | 0.207 |  |  |  |
| 167.62 | 0.042 | 217.62 | 0.044 | 267.62 | 0.148 |  | 317.62 | 0.161 | 367.62 | 0.207 |  |  |  |
| 167.64 | 0.13 | 217.64 | 0.042 | 267.64 | 0.158 |  | 317.64 | 0.152 | 367.64 | 0.193 |  |  |  |
| 167.66 | 0.093 | 217.66 | 0.04 | 267.66 | 0.162 |  | 317.66 | 0.148 | 367.66 | 0.174 |  |  |  |
| 167.68 | 0.042 | 217.68 | 0.038 | 267.68 | 0.224 |  | 317.68 | 0.118 | 367.68 | 0.183 |  |  |  |
| 167.7 | 0.092 | 217.7 | 0.032 | 267.7 | 0.286 |  | 317.7 | 0.145 | 367.7 | 0.187 |  |  |  |
| 167.72 | 0.028 | 217.72 | 0.028 | 267.72 | 0.188 |  | 317.72 | 0.181 | 367.72 | 0.186 |  |  |  |
| 167.74 | 0.042 | 217.74 | 0.026 | 267.74 | 0.244 |  | 317.74 | 0.216 | 367.74 | 0.186 |  |  |  |
| 167.76 | 0.038 | 217.76 | 0.027 | 267.76 | 0.272 |  | 317.76 | 0.222 | 367.76 | 0.148 |  |  |  |
| 167.78 | 0.038 | 217.78 | 0.031 | 267.78 | 0.359 |  | 317.78 | 0.211 | 367.78 | 0.137 |  |  |  |
| 167.8 | 0.037 | 217.8 | 0.023 | 267.8 | 0.267 |  | 317.8 | 0.198 | 367.8 | 0.167 |  |  |  |
| 167.82 | 0.076 | 217.82 | 0.015 | 267.82 | 0.034 |  | 317.82 | 0.208 | 367.82 | 0.201 |  |  |  |
| 167.84 | 0.084 | 217.84 | 0.015 | 267.84 | -0.039 |  | 317.84 | 0.211 | 367.84 | 0.217 |  |  |  |
| 167.86 | 0.092 | 217.86 | 0.028 | 267.86 | -0.011 |  | 317.86 | 0.208 | 367.86 | 0.232 |  |  |  |
| 167.88 | 0.078 | 217.88 | 0.043 | 267.88 | -0.0134 |  | 317.88 | 0.156 | 367.88 | 0.248 |  |  |  |
| 167.9 | 0.104 | 217.9 | 0.045 | 267.9 | -0.014 |  | 317.9 | 0.111 | 367.9 | 0.223 |  |  |  |
| 167.92 | 0.119 | 217.92 | 0.048 | 267.92 | -0.0152 |  | 317.92 | 0.136 | 367.92 | 0.228 |  |  |  |
| 167.94 | 0.115 | 217.94 | 0.055 | 267.94 | -0.016 |  | 317.94 | 0.184 | 367.94 | 0.225 |  |  |  |
| 167.96 | 0.066 | 217.96 | 0.056 | 267.96 | -0.0129 |  | 317.96 | 0.177 | 367.96 | 0.216 |  |  |  |
| 167.98 | 0.061 | 217.98 | 0.057 | 267.98 | -0.0102 |  | 317.98 | 0.17 | 367.98 | 0.215 |  |  |  |
| 168 | 0.084 | 218 | 0.053 | 268 | -0.065 |  | 318 | 0.011 | 368 | 0.212 |  |  |  |
| 168.02 | 0.079 | 218.02 | 0.048 | 268.02 | 0.012 |  | 318.02 | 0.032 | 368.02 | 0.22 |  |  |  |
| 168.04 | 0.14 | 218.04 | 0.049 | 268.04 | 0.111 |  | 318.04 | 0.046 | 368.04 | 0.206 |  |  |  |
| 168.06 | 0.159 | 218.06 | 0.058 | 268.06 | 0.149 |  | 318.06 | 0.082 | 368.06 | 0.193 |  |  |  |
| 168.08 | 0.157 | 218.08 | 0.061 | 268.08 | 0.137 |  | 318.08 | 0.093 | 368.08 | 0.215 |  |  |  |
| 168.1 | 0.16 | 218.1 | 0.057 | 268.1 | 0.112 |  | 318.1 | 0.107 | 368.1 | 0.204 |  |  |  |
| 168.12 | 0.163 | 218.12 | 0.064 | 268.12 | 0.071 |  | 318.12 | 0.117 | 368.12 | 0.195 |  |  |  |
| 168.14 | 0.148 | 218.14 | 0.066 | 268.14 | 0.088 |  | 318.14 | 0.121 | 368.14 | 0.197 |  |  |  |
| 168.16 | 0.156 | 218.16 | 0.066 | 268.16 | 0.141 |  | 318.16 | 0.105 | 368.16 | 0.2 |  |  |  |
| 168.18 | 0.156 | 218.18 | 0.046 | 268.18 | 0.175 |  | 318.18 | 0.039 | 368.18 | 0.171 |  |  |  |
| 168.2 | 0.151 | 218.2 | 0.055 | 268.2 | 0.21 |  | 318.2 | 0.038 | 368.2 | 0.151 |  |  |  |
| 168.22 | 0.145 | 218.22 | 0.077 | 268.22 | 0.08 |  | 318.22 | 0.129 | 368.22 | 0.176 |  |  |  |
| 168.24 | 0.141 | 218.24 | 0.096 | 268.24 | 0.044 |  | 318.24 | 0.146 | 368.24 | 0.192 |  |  |  |
| 168.26 | 0.148 | 218.26 | 0.104 | 268.26 | 0.014 |  | 318.26 | 0.151 | 368.26 | 0.192 |  |  |  |
| 168.28 | 0.186 | 218.28 | 0.101 | 268.28 | 0.022 |  | 318.28 | 0.158 | 368.28 | 0.205 |  |  |  |
| 168.3 | 0.182 | 218.3 | 0.074 | 268.3 | 0.058 |  | 318.3 | 0.162 | 368.3 | 0.189 |  |  |  |
| 168.32 | 0.179 | 218.32 | 0.041 | 268.32 | 0.065 |  | 318.32 | 0.174 | 368.32 | 0.213 |  |  |  |
| 168.34 | 0.138 | 218.34 | 0.06 | 268.34 | 0.042 |  | 318.34 | 0.183 | 368.34 | 0.219 |  |  |  |
| 168.36 | 0.145 | 218.36 | 0.067 | 268.36 | 0.097 |  | 318.36 | 0.177 | 368.36 | 0.224 |  |  |  |
| 168.38 | 0.135 | 218.38 | 0.072 | 268.38 | 0.12 |  | 318.38 | 0.179 | 368.38 | 0.222 |  |  |  |
| 168.4 | 0.094 | 218.4 | 0.089 | 268.4 | 0.137 |  | 318.4 | 0.19 | 368.4 | 0.215 |  |  |  |
| 168.42 | 0.068 | 218.42 | 0.117 | 268.42 | 0.145 |  | 318.42 | 0.159 | 368.42 | 0.182 |  |  |  |
| 168.44 | 0.038 | 218.44 | 0.127 | 268.44 | 0.155 |  | 318.44 | 0.149 | 368.44 | 0.191 |  |  |  |
| 168.46 | 0.021 | 218.46 | 0.109 | 268.46 | 0.163 |  | 318.46 | 0.134 | 368.46 | 0.2 |  |  |  |
| 168.48 | 0.007 | 218.48 | 0.07 | 268.48 | 0.155 |  | 318.48 | 0.135 | 368.48 | 0.219 |  |  |  |
| 168.5 | -0.012 | 218.5 | 0.069 | 268.5 | 0.152 |  | 318.5 | 0.129 | 368.5 | 0.226 |  |  |  |
| 168.52 | 0.024 | 218.52 | 0.073 | 268.52 | 0.173 |  | 318.52 | 0.118 | 368.52 | 0.232 |  |  |  |
| 168.54 | 0.039 | 218.54 | 0.07 | 268.54 | 0.216 |  | 318.54 | 0.093 | 368.54 | 0.241 |  |  |  |
| 168.56 | 0.057 | 218.56 | 0.06 | 268.56 | 0.251 |  | 318.56 | 0.171 | 368.56 | 0.254 |  |  |  |
| 168.58 | 0.064 | 218.58 | 0.055 | 268.58 | 0.251 |  | 318.58 | 0.189 | 368.58 | 0.26 |  |  |  |
| 168.6 | 0.076 | 218.6 | 0.062 | 268.6 | 0.207 |  | 318.6 | 0.219 | 368.6 | 0.248 |  |  |  |
| 168.62 | 0.038 | 218.62 | 0.063 | 268.62 | 0.253 |  | 318.62 | 0.219 | 368.62 | 0.24 |  |  |  |
| 168.64 | 0.052 | 218.64 | 0.055 | 268.64 | 0.229 |  | 318.64 | 0.198 | 368.64 | 0.233 |  |  |  |
| 168.66 | 0.085 | 218.66 | 0.04 | 268.66 | 0.228 |  | 318.66 | 0.185 | 368.66 | 0.21 |  |  |  |
| 168.68 | 0.127 | 218.68 | 0.036 | 268.68 | 0.23 |  | 318.68 | 0.197 | 368.68 | 0.228 |  |  |  |
| 168.7 | 0.115 | 218.7 | 0.057 | 268.7 | 0.239 |  | 318.7 | 0.222 | 368.7 | 0.232 |  |  |  |
| 168.72 | 0.1 | 218.72 | 0.063 | 268.72 | 0.254 |  | 318.72 | 0.235 | 368.72 | 0.229 |  |  |  |
| 168.74 | 0.086 | 218.74 | 0.062 | 268.74 | 0.251 |  | 318.74 | 0.22 | 368.74 | 0.229 |  |  |  |
| 168.76 | 0.068 | 218.76 | 0.063 | 268.76 | 0.247 |  | 318.76 | 0.218 | 368.76 | 0.23 |  |  |  |
| 168.78 | 0.113 | 218.78 | 0.058 | 268.78 | 0.212 |  | 318.78 | 0.21 | 368.78 | 0.23 |  |  |  |
| 168.8 | 0.121 | 218.8 | 0.055 | 268.8 | 0.173 |  | 318.8 | 0.201 | 368.8 | 0.225 |  |  |  |
| 168.82 | 0.135 | 218.82 | 0.051 | 268.82 | 0.155 |  | 318.82 | 0.213 | 368.82 | 0.21 |  |  |  |
| 168.84 | 0.13 | 218.84 | 0.056 | 268.84 | 0.196 |  | 318.84 | 0.214 | 368.84 | 0.191 |  |  |  |
| 168.86 | 0.151 | 218.86 | 0.079 | 268.86 | 0.221 |  | 318.86 | 0.203 | 368.86 | 0.168 |  |  |  |
| 168.88 | 0.191 | 218.88 | 0.174 | 268.88 | 0.215 |  | 318.88 | 0.2 | 368.88 | 0.186 |  |  |  |
| 168.9 | 0.169 | 218.9 | 0.262 | 268.9 | 0.197 |  | 318.9 | 0.189 | 368.9 | 0.193 |  |  |  |
| 168.92 | 0.144 | 218.92 | 0.193 | 268.92 | 0.191 |  | 318.92 | 0.221 | 368.92 | 0.202 |  |  |  |
| 168.94 | 0.145 | 218.94 | 0.138 | 268.94 | 0.226 |  | 318.94 | 0.224 | 368.94 | 0.21 |  |  |  |
| 168.96 | 0.264 | 218.96 | 0.106 | 268.96 | 0.22 |  | 318.96 | 0.206 | 368.96 | 0.217 |  |  |  |
| 168.98 | 0.198 | 218.98 | 0.073 | 268.98 | 0.222 |  | 318.98 | 0.184 | 368.98 | 0.214 |  |  |  |
| 169 | 0.141 | 219 | 0.071 | 269 | 0.225 |  | 319 | 0.195 | 369 | 0.21 |  |  |  |
| 169.02 | 0.002 | 219.02 | 0.072 | 269.02 | 0.013 |  | 319.02 | 0.206 | 369.02 | 0.191 |  |  |  |
| 169.04 | -0.004 | 219.04 | 0.069 | 269.04 | 0.015 |  | 319.04 | 0.18 | 369.04 | 0.208 |  |  |  |
| 169.06 | 0.009 | 219.06 | 0.063 | 269.06 | 0.043 |  | 319.06 | 0.167 | 369.06 | 0.201 |  |  |  |
| 169.08 | 0.022 | 219.08 | 0.061 | 269.08 | 0.074 |  | 319.08 | 0.19 | 369.08 | 0.208 |  |  |  |
| 169.1 | 0.035 | 219.1 | 0.055 | 269.1 | 0.082 |  | 319.1 | 0.215 | 369.1 | 0.217 |  |  |  |
| 169.12 | 0.051 | 219.12 | 0.07 | 269.12 | 0.117 |  | 319.12 | 0.192 | 369.12 | 0.226 |  |  |  |
| 169.14 | 0.082 | 219.14 | 0.072 | 269.14 | 0.175 |  | 319.14 | 0.183 | 369.14 | 0.225 |  |  |  |
| 169.16 | 0.161 | 219.16 | 0.063 | 269.16 | 0.244 |  | 319.16 | 0.139 | 369.16 | 0.219 |  |  |  |
| 169.18 | 0.093 | 219.18 | 0.052 | 269.18 | 0.227 |  | 319.18 | 0.155 | 369.18 | 0.213 |  |  |  |
| 169.2 | 0.087 | 219.2 | 0.035 | 269.2 | 0.145 |  | 319.2 | 0.189 | 369.2 | 0.211 |  |  |  |
| 169.22 | 0.098 | 219.22 | 0.023 | 269.22 | 0.122 |  | 319.22 | 0.202 | 369.22 | 0.213 |  |  |  |
| 169.24 | 0.127 | 219.24 | 0.021 | 269.24 | 0.128 |  | 319.24 | 0.201 | 369.24 | 0.212 |  |  |  |
| 169.26 | 0.158 | 219.26 | 0.018 | 269.26 | 0.094 |  | 319.26 | 0.202 | 369.26 | 0.208 |  |  |  |
| 169.28 | 0.498 | 219.28 | 0.015 | 269.28 | 0.129 |  | 319.28 | 0.206 | 369.28 | 0.181 |  |  |  |
| 169.3 | 0.148 | 219.3 | 0.016 | 269.3 | 0.112 |  | 319.3 | 0.207 | 369.3 | 0.194 |  |  |  |
| 169.32 | 0.081 | 219.32 | 0.009 | 269.32 | 0.126 |  | 319.32 | 0.138 | 369.32 | 0.201 |  |  |  |
| 169.34 | 0.064 | 219.34 | 0.004 | 269.34 | 0.144 |  | 319.34 | 0.154 | 369.34 | 0.203 |  |  |  |
| 169.36 | 0.059 | 219.36 | 0.003 | 269.36 | 0.145 |  | 319.36 | 0.134 | 369.36 | 0.205 |  |  |  |
| 169.38 | 0.054 | 219.38 | 0 | 269.38 | 0.149 |  | 319.38 | 0.141 | 369.38 | 0.22 |  |  |  |
| 169.4 | 0.069 | 219.4 | 0.008 | 269.4 | 0.154 |  | 319.4 | 0.159 | 369.4 | 0.229 |  |  |  |
| 169.42 | 0.085 | 219.42 | 0.006 | 269.42 | 0.106 |  | 319.42 | 0.18 | 369.42 | 0.234 |  |  |  |
| 169.44 | 0.092 | 219.44 | 0.006 | 269.44 | 0.107 |  | 319.44 | 0.204 | 369.44 | 0.225 |  |  |  |
| 169.46 | 0.109 | 219.46 | 0.007 | 269.46 | 0.09 |  | 319.46 | 0.234 | 369.46 | 0.223 |  |  |  |
| 169.48 | 0.179 | 219.48 | 0.057 | 269.48 | 0.086 |  | 319.48 | 0.202 | 369.48 | 0.226 |  |  |  |
| 169.5 | 0.055 | 219.5 | 0.056 | 269.5 | 0.104 |  | 319.5 | 0.216 | 369.5 | 0.231 |  |  |  |
| 169.52 | 0.067 | 219.52 | 0.061 | 269.52 | 0.136 |  | 319.52 | 0.257 | 369.52 | 0.241 |  |  |  |
| 169.54 | 0.036 | 219.54 | 0.066 | 269.54 | 0.154 |  | 319.54 | 0.258 | 369.54 | 0.236 |  |  |  |
| 169.56 | 0.038 | 219.56 | 0.065 | 269.56 | 0.164 |  | 319.56 | 0.254 | 369.56 | 0.23 |  |  |  |
| 169.58 | 0.056 | 219.58 | 0.23 | 269.58 | 0.161 |  | 319.58 | 0.237 | 369.58 | 0.234 |  |  |  |
| 169.6 | 0.084 | 219.6 | 0.22 | 269.6 | 0.152 |  | 319.6 | 0.186 | 369.6 | 0.238 |  |  |  |
| 169.62 | 0.135 | 219.62 | 0.208 | 269.62 | 0.14 |  | 319.62 | 0.229 | 369.62 | 0.231 |  |  |  |
| 169.64 | 0.15 | 219.64 | 0.199 | 269.64 | 0.138 |  | 319.64 | 0.237 | 369.64 | 0.227 |  |  |  |
| 169.66 | 0.126 | 219.66 | 0.188 | 269.66 | 0.14 |  | 319.66 | 0.24 | 369.66 | 0.233 |  |  |  |
| 169.68 | 0.12 | 219.68 | 0.179 | 269.68 | 0.141 |  | 319.68 | 0.221 | 369.68 | 0.239 |  |  |  |
| 169.7 | 0.118 | 219.7 | 0.167 | 269.7 | 0.139 |  | 319.7 | 0.24 | 369.7 | 0.189 |  |  |  |
| 169.72 | 0.084 | 219.72 | 0.151 | 269.72 | 0.147 |  | 319.72 | 0.104 | 369.72 | 0.208 |  |  |  |
| 169.74 | 0.151 | 219.74 | 0.14 | 269.74 | 0.174 |  | 319.74 | 0.124 | 369.74 | 0.289 |  |  |  |
| 169.76 | 0.147 | 219.76 | 0.121 | 269.76 | 0.204 |  | 319.76 | 0.122 | 369.76 | 0.172 |  |  |  |
| 169.78 | 0.188 | 219.78 | 0.038 | 269.78 | 0.191 |  | 319.78 | 0.144 | 369.78 | 0.163 |  |  |  |
| 169.8 | 0.112 | 219.8 | 0.038 | 269.8 | 0.176 |  | 319.8 | 0.197 | 369.8 | 0.192 |  |  |  |
| 169.82 | 0.098 | 219.82 | 0.043 | 269.82 | 0.174 |  | 319.82 | 0.188 | 369.82 | 0.189 |  |  |  |
| 169.84 | 0.103 | 219.84 | 0.041 | 269.84 | 0.187 |  | 319.84 | 0.154 | 369.84 | 0.186 |  |  |  |
| 169.86 | 0.116 | 219.86 | 0.045 | 269.86 | 0.172 |  | 319.86 | 0.134 | 369.86 | 0.173 |  |  |  |
| 169.88 | 0.125 | 219.88 | 0.046 | 269.88 | 0.116 |  | 319.88 | 0.177 | 369.88 | 0.178 |  |  |  |
| 169.9 | 0.114 | 219.9 | 0.037 | 269.9 | 0.137 |  | 319.9 | 0.181 | 369.9 | 0.189 |  |  |  |
| 169.92 | 0.103 | 219.92 | 0.027 | 269.92 | 0.164 |  | 319.92 | 0.167 | 369.92 | 0.201 |  |  |  |
| 169.94 | 0.116 | 219.94 | 0.019 | 269.94 | 0.173 |  | 319.94 | 0.15 | 369.94 | 0.215 |  |  |  |
| 169.96 | 0.135 | 219.96 | 0.016 | 269.96 | 0.183 |  | 319.96 | 0.088 | 369.96 | 0.22 |  |  |  |
| 169.98 | 0.141 | 219.98 | 0.007 | 269.98 | 0.193 |  | 319.98 | 0.046 | 369.98 | 0.234 |  |  |  |
| 170 | 0.153 | 220 | -0.008 | 270 | 0.187 |  | 320 | 0.068 | 370 | 0.253 |  |  |  |
| 170.02 | 0.075 | 220.02 | -0.019 | 270.02 | 0.162 |  | 320.02 | 0.116 | 370.02 | 0.262 |  |  |  |
| 170.04 | 0.071 | 220.04 | -0.03 | 270.04 | 0.157 |  | 320.04 | 0.204 | 370.04 | 0.256 |  |  |  |
| 170.06 | 0.073 | 220.06 | -0.042 | 270.06 | 0.155 |  | 320.06 | 0.19 | 370.06 | 0.225 |  |  |  |
| 170.08 | 0.05 | 220.08 | -0.031 | 270.08 | 0.155 |  | 320.08 | 0.184 | 370.08 | 0.223 |  |  |  |
| 170.1 | 0.082 | 220.1 | -0.033 | 270.1 | 0.165 |  | 320.1 | 0.229 | 370.1 | 0.229 |  |  |  |
| 170.12 | 0.109 | 220.12 | -0.032 | 270.12 | 0.179 |  | 320.12 | 0.223 | 370.12 | 0.214 |  |  |  |
| 170.14 | 0.111 | 220.14 | -0.03 | 270.14 | 0.212 |  | 320.14 | 0.232 | 370.14 | 0.213 |  |  |  |
| 170.16 | 0.101 | 220.16 | -0.029 | 270.16 | 0.222 |  | 320.16 | 0.204 | 370.16 | 0.22 |  |  |  |
| 170.18 | 0.08 | 220.18 | -0.029 | 270.18 | 0.234 |  | 320.18 | 0.204 | 370.18 | 0.234 |  |  |  |
| 170.2 | 0.071 | 220.2 | -0.027 | 270.2 | 0.232 |  | 320.2 | 0.23 | 370.2 | 0.246 |  |  |  |
| 170.22 | 0.061 | 220.22 | -0.026 | 270.22 | 0.264 |  | 320.22 | 0.233 | 370.22 | 0.224 |  |  |  |
| 170.24 | 0.046 | 220.24 | -0.027 | 270.24 | 0.256 |  | 320.24 | 0.241 | 370.24 | 0.191 |  |  |  |
| 170.26 | 0.02 | 220.26 | -0.028 | 270.26 | 0.234 |  | 320.26 | 0.257 | 370.26 | 0.199 |  |  |  |
| 170.28 | 0.047 | 220.28 | -0.031 | 270.28 | 0.221 |  | 320.28 | 0.249 | 370.28 | 0.199 |  |  |  |
| 170.3 | 0.084 | 220.3 | -0.036 | 270.3 | 0.224 |  | 320.3 | 0.235 | 370.3 | 0.198 |  |  |  |
| 170.32 | 0.108 | 220.32 | -0.037 | 270.32 | 0.222 |  | 320.32 | 0.232 | 370.32 | 0.198 |  |  |  |
| 170.34 | 0.103 | 220.34 | -0.034 | 270.34 | 0.204 |  | 320.34 | 0.215 | 370.34 | 0.21 |  |  |  |
| 170.36 | 0.114 | 220.36 | -0.029 | 270.36 | 0.187 |  | 320.36 | 0.222 | 370.36 | 0.227 |  |  |  |
| 170.38 | 0.128 | 220.38 | -0.007 | 270.38 | 0.176 |  | 320.38 | 0.202 | 370.38 | 0.236 |  |  |  |
| 170.4 | 0.148 | 220.4 | 0 | 270.4 | 0.177 |  | 320.4 | 0.191 | 370.4 | 0.237 |  |  |  |
| 170.42 | 0.002 | 220.42 | 0.005 | 270.42 | 0.175 |  | 320.42 | 0.164 | 370.42 | 0.238 |  |  |  |
| 170.44 | -0.051 | 220.44 | 0.012 | 270.44 | 0.162 |  | 320.44 | 0.162 | 370.44 | 0.241 |  |  |  |
| 170.46 | -0.021 | 220.46 | 0.015 | 270.46 | 0.16 |  | 320.46 | 0.19 | 370.46 | 0.213 |  |  |  |
| 170.48 | -0.006 | 220.48 | 0.021 | 270.48 | 0.151 |  | 320.48 | 0.185 | 370.48 | 0.185 |  |  |  |
| 170.5 | -0.004 | 220.5 | 0.027 | 270.5 | 0.156 |  | 320.5 | 0.156 | 370.5 | 0.182 |  |  |  |
| 170.52 | 0.003 | 220.52 | 0.034 | 270.52 | 0.134 |  | 320.52 | 0.198 | 370.52 | 0.176 |  |  |  |
| 170.54 | 0.018 | 220.54 | 0.039 | 270.54 | 0.143 |  | 320.54 | 0.154 | 370.54 | 0.184 |  |  |  |
| 170.56 | 0.041 | 220.56 | 0.043 | 270.56 | 0.164 |  | 320.56 | 0.196 | 370.56 | 0.191 |  |  |  |
| 170.58 | 0.057 | 220.58 | 0.046 | 270.58 | 0.168 |  | 320.58 | 0.163 | 370.58 | 0.193 |  |  |  |
| 170.6 | 0.051 | 220.6 | 0.048 | 270.6 | 0.181 |  | 320.6 | 0.112 | 370.6 | 0.195 |  |  |  |
| 170.62 | 0.051 | 220.62 | 0.048 | 270.62 | 0.187 |  | 320.62 | 0.202 | 370.62 | 0.202 |  |  |  |
| 170.64 | 0.079 | 220.64 | 0.049 | 270.64 | 0.214 |  | 320.64 | 0.223 | 370.64 | 0.201 |  |  |  |
| 170.66 | 0.145 | 220.66 | 0.049 | 270.66 | 0.221 |  | 320.66 | 0.241 | 370.66 | 0.205 |  |  |  |
| 170.68 | 0.174 | 220.68 | 0.045 | 270.68 | 0.202 |  | 320.68 | 0.256 | 370.68 | 0.212 |  |  |  |
| 170.7 | 0.124 | 220.7 | 0.041 | 270.7 | 0.187 |  | 320.7 | 0.255 | 370.7 | 0.207 |  |  |  |
| 170.72 | 0.085 | 220.72 | 0.039 | 270.72 | 0.184 |  | 320.72 | 0.241 | 370.72 | 0.184 |  |  |  |
| 170.74 | 0.079 | 220.74 | 0.017 | 270.74 | 0.178 |  | 320.74 | 0.25 | 370.74 | 0.214 |  |  |  |
| 170.76 | 0.074 | 220.76 | 0.018 | 270.76 | 0.175 |  | 320.76 | 0.255 | 370.76 | 0.219 |  |  |  |
| 170.78 | 0.075 | 220.78 | 0.035 | 270.78 | 0.171 |  | 320.78 | 0.199 | 370.78 | 0.229 |  |  |  |
| 170.8 | 0.089 | 220.8 | 0.028 | 270.8 | 0.169 |  | 320.8 | 0.218 | 370.8 | 0.24 |  |  |  |
| 170.82 | 0.089 | 220.82 | 0.014 | 270.82 | 0.149 |  | 320.82 | 0.219 | 370.82 | 0.224 |  |  |  |
| 170.84 | 0.051 | 220.84 | 0.006 | 270.84 | 0.148 |  | 320.84 | 0.232 | 370.84 | 0.161 |  |  |  |
| 170.86 | 0.115 | 220.86 | -0.003 | 270.86 | 0.136 |  | 320.86 | 0.228 | 370.86 | 0.171 |  |  |  |
| 170.88 | 0.127 | 220.88 | -0.011 | 270.88 | 0.142 |  | 320.88 | 0.211 | 370.88 | 0.186 |  |  |  |
| 170.9 | 0.093 | 220.9 | -0.022 | 270.9 | 0.144 |  | 320.9 | 0.187 | 370.9 | 0.208 |  |  |  |
| 170.92 | 0.073 | 220.92 | -0.031 | 270.92 | 0.134 |  | 320.92 | 0.192 | 370.92 | 0.222 |  |  |  |
| 170.94 | 0.086 | 220.94 | -0.042 | 270.94 | 0.156 |  | 320.94 | 0.18 | 370.94 | 0.227 |  |  |  |
| 170.96 | 0.079 | 220.96 | -0.042 | 270.96 | 0.149 |  | 320.96 | 0.183 | 370.96 | 0.225 |  |  |  |
| 170.98 | 0.072 | 220.98 | -0.039 | 270.98 | 0.125 |  | 320.98 | 0.217 | 370.98 | 0.213 |  |  |  |
| 171 | 0.081 | 221 | -0.04 | 271 | 0.117 |  | 321 | 0.163 | 371 | 0.192 |  |  |  |
| 171.02 | 0.093 | 221.02 | -0.042 | 271.02 | 0.008 |  | 321.02 | 0.159 | 371.02 | 0.176 |  |  |  |
| 171.04 | 0.1 | 221.04 | -0.044 | 271.04 | 0.014 |  | 321.04 | 0.142 | 371.04 | 0.171 |  |  |  |
| 171.06 | 0.099 | 221.06 | -0.045 | 271.06 | 0.016 |  | 321.06 | 0.125 | 371.06 | 0.181 |  |  |  |
| 171.08 | 0.094 | 221.08 | -0.046 | 271.08 | 0.024 |  | 321.08 | 0.099 | 371.08 | 0.181 |  |  |  |
| 171.1 | 0.093 | 221.1 | -0.048 | 271.1 | 0.027 |  | 321.1 | 0.066 | 371.1 | 0.173 |  |  |  |
| 171.12 | 0.134 | 221.12 | -0.052 | 271.12 | 0.026 |  | 321.12 | 0.145 | 371.12 | 0.18 |  |  |  |
| 171.14 | 0.133 | 221.14 | -0.06 | 271.14 | 0.042 |  | 321.14 | 0.135 | 371.14 | 0.171 |  |  |  |
| 171.16 | 0.143 | 221.16 | -0.06 | 271.16 | 0.079 |  | 321.16 | 0.2 | 371.16 | 0.149 |  |  |  |
| 171.18 | 0.152 | 221.18 | -0.013 | 271.18 | 0.096 |  | 321.18 | 0.25 | 371.18 | 0.142 |  |  |  |
| 171.2 | 0.203 | 221.2 | -0.012 | 271.2 | 0.1 |  | 321.2 | 0.287 | 371.2 | 0.14 |  |  |  |
| 171.22 | 0.393 | 221.22 | -0.018 | 271.22 | 0.093 |  | 321.22 | 0.245 | 371.22 | 0.119 |  |  |  |
| 171.24 | 0.286 | 221.24 | 0.016 | 271.24 | 0.089 |  | 321.24 | 0.172 | 371.24 | 0.211 |  |  |  |
| 171.26 | 0.2718 | 221.26 | 0.044 | 271.26 | 0.086 |  | 321.26 | 0.14 | 371.26 | 0.225 |  |  |  |
| 171.28 | 0.378 | 221.28 | 0.008 | 271.28 | 0.089 |  | 321.28 | 0.134 | 371.28 | 0.234 |  |  |  |
| 171.3 | 0.2874 | 221.3 | 0.002 | 271.3 | 0.1 |  | 321.3 | 0.113 | 371.3 | 0.238 |  |  |  |
| 171.32 | 0.3144 | 221.32 | 0.049 | 271.32 | 0.118 |  | 321.32 | 0.142 | 371.32 | 0.229 |  |  |  |
| 171.34 | 0.3246 | 221.34 | 0.087 | 271.34 | 0.146 |  | 321.34 | 0.186 | 371.34 | 0.215 |  |  |  |
| 171.36 | 0.3528 | 221.36 | 0.073 | 271.36 | 0.161 |  | 321.36 | 0.216 | 371.36 | 0.204 |  |  |  |
| 171.38 | 0.29 | 221.38 | 0.055 | 271.38 | 0.166 |  | 321.38 | 0.182 | 371.38 | 0.202 |  |  |  |
| 171.4 | 0.2766 | 221.4 | 0.051 | 271.4 | 0.177 |  | 321.4 | 0.193 | 371.4 | 0.189 |  |  |  |
| 171.42 | 0.3768 | 221.42 | 0.063 | 271.42 | 0.084 |  | 321.42 | 0.236 | 371.42 | 0.189 |  |  |  |
| 171.44 | 0.4386 | 221.44 | 0.081 | 271.44 | 0.133 |  | 321.44 | 0.302 | 371.44 | 0.185 |  |  |  |
| 171.46 | 0.324 | 221.46 | 0.101 | 271.46 | 0.144 |  | 321.46 | 0.24 | 371.46 | 0.145 |  |  |  |
| 171.48 | 0.308 | 221.48 | 0.103 | 271.48 | 0.106 |  | 321.48 | 0.322 | 371.48 | 0.138 |  |  |  |
| 171.5 | 0.187 | 221.5 | 0.068 | 271.5 | 0.038 |  | 321.5 | 0.249 | 371.5 | 0.123 |  |  |  |
| 171.52 | 0.166 | 221.52 | 0.076 | 271.52 | -0.018 |  | 321.52 | 0.212 | 371.52 | 0.124 |  |  |  |
| 171.54 | 0.201 | 221.54 | 0.092 | 271.54 | 0.034 |  | 321.54 | 0.204 | 371.54 | 0.136 |  |  |  |
| 171.56 | 0.23 | 221.56 | 0.08 | 271.56 | 0.058 |  | 321.56 | 0.202 | 371.56 | 0.151 |  |  |  |
| 171.58 | 0.206 | 221.58 | 0.059 | 271.58 | 0.06 |  | 321.58 | 0.194 | 371.58 | 0.174 |  |  |  |
| 171.6 | 0.124 | 221.6 | 0.035 | 271.6 | 0.058 |  | 321.6 | 0.197 | 371.6 | 0.2 |  |  |  |
| 171.62 | 0.06 | 221.62 | 0.025 | 271.62 | 0.062 |  | 321.62 | 0.185 | 371.62 | 0.215 |  |  |  |
| 171.64 | 0.058 | 221.64 | 0.026 | 271.64 | 0.073 |  | 321.64 | 0.196 | 371.64 | 0.217 |  |  |  |
| 171.66 | 0.062 | 221.66 | 0.004 | 271.66 | 0.047 |  | 321.66 | 0.199 | 371.66 | 0.206 |  |  |  |
| 171.68 | 0.049 | 221.68 | -0.027 | 271.68 | 0.033 |  | 321.68 | 0.175 | 371.68 | 0.19 |  |  |  |
| 171.7 | 0.048 | 221.7 | -0.041 | 271.7 | 0.063 |  | 321.7 | 0.139 | 371.7 | 0.17 |  |  |  |
| 171.72 | 0.063 | 221.72 | -0.036 | 271.72 | 0.078 |  | 321.72 | 0.16 | 371.72 | 0.148 |  |  |  |
| 171.74 | 0.084 | 221.74 | -0.025 | 271.74 | 0.126 |  | 321.74 | 0.124 | 371.74 | 0.154 |  |  |  |
| 171.76 | 0.106 | 221.76 | -0.018 | 271.76 | 0.142 |  | 321.76 | 0.157 | 371.76 | 0.185 |  |  |  |
| 171.78 | 0.112 | 221.78 | -0.01 | 271.78 | 0.154 |  | 321.78 | 0.188 | 371.78 | 0.205 |  |  |  |
| 171.8 | 0.095 | 221.8 | -0.005 | 271.8 | 0.164 |  | 321.8 | 0.181 | 371.8 | 0.204 |  |  |  |
| 171.82 | 0.069 | 221.82 | -0.007 | 271.82 | 0.185 |  | 321.82 | 0.184 | 371.82 | 0.206 |  |  |  |
| 171.84 | 0.081 | 221.84 | -0.027 | 271.84 | 0.188 |  | 321.84 | 0.205 | 371.84 | 0.211 |  |  |  |
| 171.86 | 0.083 | 221.86 | 0.027 | 271.86 | 0.184 |  | 321.86 | 0.228 | 371.86 | 0.212 |  |  |  |
| 171.88 | 0.096 | 221.88 | 0.068 | 271.88 | 0.173 |  | 321.88 | 0.247 | 371.88 | 0.203 |  |  |  |
| 171.9 | 0.103 | 221.9 | 0.037 | 271.9 | 0.17 |  | 321.9 | 0.293 | 371.9 | 0.176 |  |  |  |
| 171.92 | 0.101 | 221.92 | 0.012 | 271.92 | 0.18 |  | 321.92 | 0.263 | 371.92 | 0.167 |  |  |  |
| 171.94 | 0.082 | 221.94 | 0.014 | 271.94 | 0.176 |  | 321.94 | 0.211 | 371.94 | 0.169 |  |  |  |
| 171.96 | 0.075 | 221.96 | 0.012 | 271.96 | 0.168 |  | 321.96 | 0.175 | 371.96 | 0.176 |  |  |  |
| 171.98 | 0.076 | 221.98 | 0.034 | 271.98 | 0.172 |  | 321.98 | 0.175 | 371.98 | 0.174 |  |  |  |
| 172 | 0.086 | 222 | 0.055 | 272 | 0.163 |  | 322 | 0.118 | 372 | 0.17 |  |  |  |
| 172.02 | 0.006 | 222.02 | 0.051 | 272.02 | 0.16 |  | 322.02 | 0.15 | 372.02 | 0.168 |  |  |  |
| 172.04 | 0.006 | 222.04 | 0.053 | 272.04 | 0.171 |  | 322.04 | 0.142 | 372.04 | 0.168 |  |  |  |
| 172.06 | -0.002 | 222.06 | 0.061 | 272.06 | 0.205 |  | 322.06 | 0.286 | 372.06 | 0.187 |  |  |  |
| 172.08 | -0.003 | 222.08 | 0.04 | 272.08 | 0.204 |  | 322.08 | 0.298 | 372.08 | 0.204 |  |  |  |
| 172.1 | 0.002 | 222.1 | 0.038 | 272.1 | 0.18 |  | 322.1 | 0.302 | 372.1 | 0.218 |  |  |  |
| 172.12 | 0.007 | 222.12 | 0.033 | 272.12 | 0.169 |  | 322.12 | 0.305 | 372.12 | 0.198 |  |  |  |
| 172.14 | 0.008 | 222.14 | 0.029 | 272.14 | 0.167 |  | 322.14 | 0.303 | 372.14 | 0.156 |  |  |  |
| 172.16 | -0.009 | 222.16 | 0.05 | 272.16 | 0.166 |  | 322.16 | 0.259 | 372.16 | 0.165 |  |  |  |
| 172.18 | 0.09 | 222.18 | 0.044 | 272.18 | 0.167 |  | 322.18 | 0.242 | 372.18 | 0.165 |  |  |  |
| 172.2 | -0.005 | 222.2 | 0.056 | 272.2 | 0.172 |  | 322.2 | 0.256 | 372.2 | 0.159 |  |  |  |
| 172.22 | 0.015 | 222.22 | 0.065 | 272.22 | 0.013 |  | 322.22 | 0.278 | 372.22 | 0.149 |  |  |  |
| 172.24 | 0.03 | 222.24 | 0.058 | 272.24 | 0.012 |  | 322.24 | 0.249 | 372.24 | 0.145 |  |  |  |
| 172.26 | 0.041 | 222.26 | 0.066 | 272.26 | 0.028 |  | 322.26 | 0.255 | 372.26 | 0.142 |  |  |  |
| 172.28 | 0.062 | 222.28 | 0.089 | 272.28 | 0.019 |  | 322.28 | 0.274 | 372.28 | 0.145 |  |  |  |
| 172.3 | 0.079 | 222.3 | 0.097 | 272.3 | 0.019 |  | 322.3 | 0.295 | 372.3 | 0.222 |  |  |  |
| 172.32 | 0.082 | 222.32 | 0.085 | 272.32 | 0.022 |  | 322.32 | 0.275 | 372.32 | 0.227 |  |  |  |
| 172.34 | 0.084 | 222.34 | 0.055 | 272.34 | 0.035 |  | 322.34 | 0.281 | 372.34 | 0.23 |  |  |  |
| 172.36 | 0.085 | 222.36 | 0.03 | 272.36 | 0.066 |  | 322.36 | 0.323 | 372.36 | 0.232 |  |  |  |
| 172.38 | 0.077 | 222.38 | 0.032 | 272.38 | 0.071 |  | 322.38 | 0.329 | 372.38 | 0.223 |  |  |  |
| 172.4 | 0.126 | 222.4 | 0.049 | 272.4 | 0.073 |  | 322.4 | 0.302 | 372.4 | 0.163 |  |  |  |
| 172.42 | 0.153 | 222.42 | 0.046 | 272.42 | 0.085 |  | 322.42 | 0.279 | 372.42 | 0.153 |  |  |  |
| 172.44 | 0.111 | 222.44 | 0.049 | 272.44 | 0.098 |  | 322.44 | 0.273 | 372.44 | 0.175 |  |  |  |
| 172.46 | 0.097 | 222.46 | 0.066 | 272.46 | 0.085 |  | 322.46 | 0.272 | 372.46 | 0.194 |  |  |  |
| 172.48 | 0.095 | 222.48 | 0.069 | 272.48 | 0.132 |  | 322.48 | 0.269 | 372.48 | 0.204 |  |  |  |
| 172.5 | 0.096 | 222.5 | 0.063 | 272.5 | 0.135 |  | 322.5 | 0.271 | 372.5 | 0.214 |  |  |  |
| 172.52 | 0.099 | 222.52 | 0.069 | 272.52 | 0.131 |  | 322.52 | 0.26 | 372.52 | 0.237 |  |  |  |
| 172.54 | 0.102 | 222.54 | 0.072 | 272.54 | 0.141 |  | 322.54 | 0.186 | 372.54 | 0.228 |  |  |  |
| 172.56 | 0.109 | 222.56 | 0.04 | 272.56 | 0.144 |  | 322.56 | 0.144 | 372.56 | 0.222 |  |  |  |
| 172.58 | 0.113 | 222.58 | 0.008 | 272.58 | 0.177 |  | 322.58 | 0.211 | 372.58 | 0.218 |  |  |  |
| 172.6 | 0.104 | 222.6 | 0.001 | 272.6 | 0.216 |  | 322.6 | 0.284 | 372.6 | 0.217 |  |  |  |
| 172.62 | 0.06 | 222.62 | -0.003 | 272.62 | 0.009 |  | 322.62 | 0.314 | 372.62 | 0.2 |  |  |  |
| 172.64 | 0.065 | 222.64 | -0.003 | 272.64 | 0.022 |  | 322.64 | 0.318 | 372.64 | 0.194 |  |  |  |
| 172.66 | 0.073 | 222.66 | 0.006 | 272.66 | 0.022 |  | 322.66 | 0.305 | 372.66 | 0.21 |  |  |  |
| 172.68 | 0.078 | 222.68 | 0.009 | 272.68 | 0.01 |  | 322.68 | 0.303 | 372.68 | 0.217 |  |  |  |
| 172.7 | 0.125 | 222.7 | 0.004 | 272.7 | 0.027 |  | 322.7 | 0.301 | 372.7 | 0.219 |  |  |  |
| 172.72 | 0.159 | 222.72 | 0.008 | 272.72 | 0.008 |  | 322.72 | 0.303 | 372.72 | 0.238 |  |  |  |
| 172.74 | 0.163 | 222.74 | 0.018 | 272.74 | 0.001 |  | 322.74 | 0.312 | 372.74 | 0.247 |  |  |  |
| 172.76 | 0.126 | 222.76 | 0.026 | 272.76 | -0.013 |  | 322.76 | 0.314 | 372.76 | 0.244 |  |  |  |
| 172.78 | 0.096 | 222.78 | 0.054 | 272.78 | 0 |  | 322.78 | 0.322 | 372.78 | 0.228 |  |  |  |
| 172.8 | 0.1 | 222.8 | 0.024 | 272.8 | 0.005 |  | 322.8 | 0.343 | 372.8 | 0.199 |  |  |  |
| 172.82 | 0.107 | 222.82 | 0.08 | 272.82 | 0.016 |  | 322.82 | 0.352 | 372.82 | 0.165 |  |  |  |
| 172.84 | 0.068 | 222.84 | 0.082 | 272.84 | 0.029 |  | 322.84 | 0.379 | 372.84 | 0.149 |  |  |  |
| 172.86 | 0.09 | 222.86 | 0.118 | 272.86 | 0.04 |  | 322.86 | 0.355 | 372.86 | 0.15 |  |  |  |
| 172.88 | 0.109 | 222.88 | 0.092 | 272.88 | 0.05 |  | 322.88 | 0.256 | 372.88 | 0.151 |  |  |  |
| 172.9 | 0.13 | 222.9 | 0.076 | 272.9 | 0.067 |  | 322.9 | 0.064 | 372.9 | 0.154 |  |  |  |
| 172.92 | 0.128 | 222.92 | 0.068 | 272.92 | 0.088 |  | 322.92 | 0.281 | 372.92 | 0.157 |  |  |  |
| 172.94 | 0.115 | 222.94 | 0.028 | 272.94 | 0.106 |  | 322.94 | 0.276 | 372.94 | 0.16 |  |  |  |
| 172.96 | 0.099 | 222.96 | 0.042 | 272.96 | 0.133 |  | 322.96 | 0.271 | 372.96 | 0.151 |  |  |  |
| 172.98 | 0.097 | 222.98 | 0.032 | 272.98 | 0.154 |  | 322.98 | 0.232 | 372.98 | 0.157 |  |  |  |
| 173 | 0.115 | 223 | 0.032 | 273 | 0.15 |  | 323 | 0.254 | 373 | 0.144 |  |  |  |
| 173.02 | 0.132 | 223.02 | 0.052 | 273.02 | 0.121 |  | 323.02 | 0.271 | 373.02 | 0.134 |  |  |  |
| 173.04 | 0.136 | 223.04 | 0.071 | 273.04 | 0.123 |  | 323.04 | 0.283 | 373.04 | 0.14 |  |  |  |
| 173.06 | 0.136 | 223.06 | 0.023 | 273.06 | 0.106 |  | 323.06 | 0.276 | 373.06 | 0.15 |  |  |  |
| 173.08 | 0.13 | 223.08 | 0.017 | 273.08 | 0.1 |  | 323.08 | 0.265 | 373.08 | 0.152 |  |  |  |
| 173.1 | 0.124 | 223.1 | 0.027 | 273.1 | 0.096 |  | 323.1 | 0.268 | 373.1 | 0.156 |  |  |  |
| 173.12 | 0.053 | 223.12 | 0.027 | 273.12 | 0.087 |  | 323.12 | 0.266 | 373.12 | 0.157 |  |  |  |
| 173.14 | 0.098 | 223.14 | 0.03 | 273.14 | 0.094 |  | 323.14 | 0.234 | 373.14 | 0.157 |  |  |  |
| 173.16 | 0.104 | 223.16 | 0.027 | 273.16 | 0.111 |  | 323.16 | 0.239 | 373.16 | 0.162 |  |  |  |
| 173.18 | 0.116 | 223.18 | 0.018 | 273.18 | 0.109 |  | 323.18 | 0.214 | 373.18 | 0.162 |  |  |  |
| 173.2 | 0.124 | 223.2 | 0.008 | 273.2 | 0.07 |  | 323.2 | 0.187 | 373.2 | 0.154 |  |  |  |
| 173.22 | 0.12 | 223.22 | -0.001 | 273.22 | 0.145 |  | 323.22 | 0.205 | 373.22 | 0.155 |  |  |  |
| 173.24 | 0.255 | 223.24 | -0.01 | 273.24 | 0.166 |  | 323.24 | 0.237 | 373.24 | 0.167 |  |  |  |
| 173.26 | 0.036 | 223.26 | -0.015 | 273.26 | 0.158 |  | 323.26 | 0.234 | 373.26 | 0.175 |  |  |  |
| 173.28 | 0.285 | 223.28 | -0.015 | 273.28 | 0.136 |  | 323.28 | 0.218 | 373.28 | 0.176 |  |  |  |
| 173.3 | 0.144 | 223.3 | -0.02 | 273.3 | 0.11 |  | 323.3 | 0.19 | 373.3 | 0.178 |  |  |  |
| 173.32 | 0.118 | 223.32 | -0.026 | 273.32 | 0.102 |  | 323.32 | 0.195 | 373.32 | 0.184 |  |  |  |
| 173.34 | 0.107 | 223.34 | -0.028 | 273.34 | 0.105 |  | 323.34 | 0.192 | 373.34 | 0.201 |  |  |  |
| 173.36 | 0.111 | 223.36 | -0.028 | 273.36 | 0.103 |  | 323.36 | 0.195 | 373.36 | 0.215 |  |  |  |
| 173.38 | 0.118 | 223.38 | -0.029 | 273.38 | 0.112 |  | 323.38 | 0.198 | 373.38 | 0.22 |  |  |  |
| 173.4 | 0.1 | 223.4 | -0.034 | 273.4 | 0.121 |  | 323.4 | 0.209 | 373.4 | 0.216 |  |  |  |
| 173.42 | 0.097 | 223.42 | -0.042 | 273.42 | 0.007 |  | 323.42 | 0.225 | 373.42 | 0.208 |  |  |  |
| 173.44 | 0.088 | 223.44 | -0.028 | 273.44 | 0.015 |  | 323.44 | 0.229 | 373.44 | 0.212 |  |  |  |
| 173.46 | 0.076 | 223.46 | -0.008 | 273.46 | 0.008 |  | 323.46 | 0.235 | 373.46 | 0.2 |  |  |  |
| 173.48 | 0.08 | 223.48 | -0.044 | 273.48 | -0.004 |  | 323.48 | 0.233 | 373.48 | 0.19 |  |  |  |
| 173.5 | 0.093 | 223.5 | -0.032 | 273.5 | 0.002 |  | 323.5 | 0.232 | 373.5 | 0.189 |  |  |  |
| 173.52 | 0.087 | 223.52 | -0.042 | 273.52 | 0.002 |  | 323.52 | 0.231 | 373.52 | 0.19 |  |  |  |
| 173.54 | 0.082 | 223.54 | -0.028 | 273.54 | 0.008 |  | 323.54 | 0.229 | 373.54 | 0.198 |  |  |  |
| 173.56 | 0.078 | 223.56 | -0.029 | 273.56 | 0.026 |  | 323.56 | 0.232 | 373.56 | 0.198 |  |  |  |
| 173.58 | 0.08 | 223.58 | -0.021 | 273.58 | 0.022 |  | 323.58 | 0.231 | 373.58 | 0.195 |  |  |  |
| 173.6 | 0.074 | 223.6 | -0.008 | 273.6 | 0.023 |  | 323.6 | 0.232 | 373.6 | 0.181 |  |  |  |
| 173.62 | 0.04 | 223.62 | 0.01 | 273.62 | 0.039 |  | 323.62 | 0.227 | 373.62 | 0.162 |  |  |  |
| 173.64 | 0.041 | 223.64 | 0.033 | 273.64 | 0.054 |  | 323.64 | 0.2 | 373.64 | 0.147 |  |  |  |
| 173.66 | 0.057 | 223.66 | 0.039 | 273.66 | 0.065 |  | 323.66 | 0.186 | 373.66 | 0.151 |  |  |  |
| 173.68 | 0.09 | 223.68 | 0.072 | 273.68 | 0.049 |  | 323.68 | 0.181 | 373.68 | 0.15 |  |  |  |
| 173.7 | 0.098 | 223.7 | 0.114 | 273.7 | 0.046 |  | 323.7 | 0.222 | 373.7 | 0.144 |  |  |  |
| 173.72 | 0.044 | 223.72 | 0.116 | 273.72 | 0.042 |  | 323.72 | 0.181 | 373.72 | 0.147 |  |  |  |
| 173.74 | 0.048 | 223.74 | 0.102 | 273.74 | 0.033 |  | 323.74 | 0.196 | 373.74 | 0.151 |  |  |  |
| 173.76 | 0.034 | 223.76 | 0.074 | 273.76 | 0.03 |  | 323.76 | 0.237 | 373.76 | 0.148 |  |  |  |
| 173.78 | 0.062 | 223.78 | 0.043 | 273.78 | 0.038 |  | 323.78 | 0.247 | 373.78 | 0.09 |  |  |  |
| 173.8 | 0.092 | 223.8 | 0.037 | 273.8 | 0.048 |  | 323.8 | 0.255 | 373.8 | 0.119 |  |  |  |
| 173.82 | 0.063 | 223.82 | 0.029 | 273.82 | 0.002 |  | 323.82 | 0.262 | 373.82 | 0.116 |  |  |  |
| 173.84 | 0.2442 | 223.84 | 0.017 | 273.84 | 0.005 |  | 323.84 | 0.265 | 373.84 | 0.129 |  |  |  |
| 173.86 | 0.3036 | 223.86 | 0.017 | 273.86 | 0.01 |  | 323.86 | 0.26 | 373.86 | 0.131 |  |  |  |
| 173.88 | 0.27 | 223.88 | 0.012 | 273.88 | 0.01 |  | 323.88 | 0.252 | 373.88 | 0.12 |  |  |  |
| 173.9 | 0.305 | 223.9 | 0.025 | 273.9 | 0.007 |  | 323.9 | 0.244 | 373.9 | 0.114 |  |  |  |
| 173.92 | 0.165 | 223.92 | 0.038 | 273.92 | 0.011 |  | 323.92 | 0.238 | 373.92 | 0.109 |  |  |  |
| 173.94 | 0.122 | 223.94 | 0.054 | 273.94 | 0.014 |  | 323.94 | 0.249 | 373.94 | 0.112 |  |  |  |
| 173.96 | 0.1 | 223.96 | 0.058 | 273.96 | 0.017 |  | 323.96 | 0.261 | 373.96 | 0.119 |  |  |  |
| 173.98 | 0.094 | 223.98 | 0.052 | 273.98 | 0.007 |  | 323.98 | 0.248 | 373.98 | 0.149 |  |  |  |
| 174 | 0.136 | 224 | 0.031 | 274 | 0.016 |  | 324 | 0.248 | 374 | 0.173 |  |  |  |
| 174.02 | 0.087 | 224.02 | 0.012 | 274.02 | 0.019 |  | 324.02 | 0.25 | 374.02 | 0.184 |  |  |  |
| 174.04 | 0.089 | 224.04 | 0.006 | 274.04 | 0.021 |  | 324.04 | 0.241 | 374.04 | 0.184 |  |  |  |
| 174.06 | 0.09 | 224.06 | -0.003 | 274.06 | 0.022 |  | 324.06 | 0.255 | 374.06 | 0.182 |  |  |  |
| 174.08 | 0.093 | 224.08 | -0.008 | 274.08 | 0.025 |  | 324.08 | 0.289 | 374.08 | 0.176 |  |  |  |
| 174.1 | 0.101 | 224.1 | 0.005 | 274.1 | 0.028 |  | 324.1 | 0.291 | 374.1 | 0.172 |  |  |  |
| 174.12 | 0.103 | 224.12 | 0.018 | 274.12 | 0.029 |  | 324.12 | 0.277 | 374.12 | 0.164 |  |  |  |
| 174.14 | 0.08 | 224.14 | 0.03 | 274.14 | 0.028 |  | 324.14 | 0.266 | 374.14 | 0.156 |  |  |  |
| 174.16 | 0.066 | 224.16 | 0.025 | 274.16 | 0.034 |  | 324.16 | 0.268 | 374.16 | 0.148 |  |  |  |
| 174.18 | 0.056 | 224.18 | 0.008 | 274.18 | 0.042 |  | 324.18 | 0.246 | 374.18 | 0.141 |  |  |  |
| 174.2 | 0.036 | 224.2 | -0.007 | 274.2 | 0.043 |  | 324.2 | 0.248 | 374.2 | 0.136 |  |  |  |
| 174.22 | 0.041 | 224.22 | -0.016 | 274.22 | 0.088 |  | 324.22 | 0.266 | 374.22 | 0.143 |  |  |  |
| 174.24 | 0.058 | 224.24 | -0.027 | 274.24 | 0.097 |  | 324.24 | 0.282 | 374.24 | 0.159 |  |  |  |
| 174.26 | 0.08 | 224.26 | -0.04 | 274.26 | 0.096 |  | 324.26 | 0.287 | 374.26 | 0.169 |  |  |  |
| 174.28 | 0.098 | 224.28 | -0.048 | 274.28 | 0.095 |  | 324.28 | 0.151 | 374.28 | 0.149 |  |  |  |
| 174.3 | 0.126 | 224.3 | -0.041 | 274.3 | 0.092 |  | 324.3 | 0.297 | 374.3 | 0.116 |  |  |  |
| 174.32 | 0.175 | 224.32 | -0.036 | 274.32 | 0.091 |  | 324.32 | 0.292 | 374.32 | 0.122 |  |  |  |
| 174.34 | 0.232 | 224.34 | 0.013 | 274.34 | 0.092 |  | 324.34 | 0.254 | 374.34 | 0.128 |  |  |  |
| 174.36 | 0.255 | 224.36 | 0.03 | 274.36 | 0.107 |  | 324.36 | 0.234 | 374.36 | 0.132 |  |  |  |
| 174.38 | 0.231 | 224.38 | 0.078 | 274.38 | 0.14 |  | 324.38 | 0.173 | 374.38 | 0.135 |  |  |  |
| 174.4 | 0.193 | 224.4 | 0.064 | 274.4 | 0.178 |  | 324.4 | 0.18 | 374.4 | 0.139 |  |  |  |
| 174.42 | 0.145 | 224.42 | 0.008 | 274.42 | 0.012 |  | 324.42 | 0.243 | 374.42 | 0.139 |  |  |  |
| 174.44 | 0.088 | 224.44 | -0.013 | 274.44 | 0.038 |  | 324.44 | 0.239 | 374.44 | 0.141 |  |  |  |
| 174.46 | 0.074 | 224.46 | -0.016 | 274.46 | 0.042 |  | 324.46 | 0.25 | 374.46 | 0.145 |  |  |  |
| 174.48 | 0.072 | 224.48 | -0.017 | 274.48 | 0.05 |  | 324.48 | 0.285 | 374.48 | 0.125 |  |  |  |
| 174.5 | 0.042 | 224.5 | -0.011 | 274.5 | 0.059 |  | 324.5 | 0.211 | 374.5 | 0.122 |  |  |  |
| 174.52 | 0.04 | 224.52 | -0.008 | 274.52 | 0.067 |  | 324.52 | 0.214 | 374.52 | 0.12 |  |  |  |
| 174.54 | 0.082 | 224.54 | 0.007 | 274.54 | 0.068 |  | 324.54 | 0.255 | 374.54 | 0.126 |  |  |  |
| 174.56 | 0.087 | 224.56 | 0.029 | 274.56 | 0.057 |  | 324.56 | 0.201 | 374.56 | 0.13 |  |  |  |
| 174.58 | 0.081 | 224.58 | 0.065 | 274.58 | 0.045 |  | 324.58 | 0.187 | 374.58 | 0.129 |  |  |  |
| 174.6 | 0.077 | 224.6 | 0.116 | 274.6 | 0.045 |  | 324.6 | 0.154 | 374.6 | 0.129 |  |  |  |
| 174.62 | 0.071 | 224.62 | 0.148 | 274.62 | 0.069 |  | 324.62 | 0.126 | 374.62 | 0.13 |  |  |  |
| 174.64 | 0.045 | 224.64 | 0.16 | 274.64 | 0.05 |  | 324.64 | 0.126 | 374.64 | 0.131 |  |  |  |
| 174.66 | 0.051 | 224.66 | 0.148 | 274.66 | 0.009 |  | 324.66 | 0.102 | 374.66 | 0.129 |  |  |  |
| 174.68 | 0.058 | 224.68 | 0.117 | 274.68 | -0.003 |  | 324.68 | 0.099 | 374.68 | 0.129 |  |  |  |
| 174.7 | 0.043 | 224.7 | 0.181 | 274.7 | 0.026 |  | 324.7 | 0.131 | 374.7 | 0.127 |  |  |  |
| 174.72 | 0.03 | 224.72 | 0.042 | 274.72 | 0.008 |  | 324.72 | 0.219 | 374.72 | 0.121 |  |  |  |
| 174.74 | 0.041 | 224.74 | -0.003 | 274.74 | 0.024 |  | 324.74 | 0.132 | 374.74 | 0.112 |  |  |  |
| 174.76 | 0.035 | 224.76 | 0.009 | 274.76 | 0.149 |  | 324.76 | 0.126 | 374.76 | 0.127 |  |  |  |
| 174.78 | 0.027 | 224.78 | 0.015 | 274.78 | 0.153 |  | 324.78 | 0.156 | 374.78 | 0.16 |  |  |  |
| 174.8 | 0.02 | 224.8 | 0.016 | 274.8 | 0.142 |  | 324.8 | 0.133 | 374.8 | 0.153 |  |  |  |
| 174.82 | 0.014 | 224.82 | 0.018 | 274.82 | 0.213 |  | 324.82 | 0.108 | 374.82 | 0.146 |  |  |  |
| 174.84 | 0.011 | 224.84 | 0.008 | 274.84 | 0.201 |  | 324.84 | 0.108 | 374.84 | 0.155 |  |  |  |
| 174.86 | 0.009 | 224.86 | 0.003 | 274.86 | 0.21 |  | 324.86 | 0.131 | 374.86 | 0.189 |  |  |  |
| 174.88 | 0.008 | 224.88 | -0.002 | 274.88 | 0.207 |  | 324.88 | 0.144 | 374.88 | 0.216 |  |  |  |
| 174.9 | 0.007 | 224.9 | -0.013 | 274.9 | 0.199 |  | 324.9 | 0.199 | 374.9 | 0.216 |  |  |  |
| 174.92 | 0.005 | 224.92 | -0.023 | 274.92 | 0.193 |  | 324.92 | 0.278 | 374.92 | 0.193 |  |  |  |
| 174.94 | 0.006 | 224.94 | -0.031 | 274.94 | 0.181 |  | 324.94 | 0.356 | 374.94 | 0.163 |  |  |  |
| 174.96 | 0.006 | 224.96 | -0.039 | 274.96 | 0.177 |  | 324.96 | 0.364 | 374.96 | 0.151 |  |  |  |
| 174.98 | 0 | 224.98 | -0.043 | 274.98 | 0.185 |  | 324.98 | 0.343 | 374.98 | 0.133 |  |  |  |
| 175 | 0.002 | 225 | -0.044 | 275 | 0.196 |  | 325 | 0.301 | 375 | 0.126 |  |  |  |
| 175.02 | 0.006 | 225.02 | -0.044 | 275.02 | 0.194 |  | 325.02 | 0.197 | 375.02 | 0.13 |  |  |  |
| 175.04 | 0.006 | 225.04 | -0.048 | 275.04 | 0.169 |  | 325.04 | 0.147 | 375.04 | 0.134 |  |  |  |
| 175.06 | 0.011 | 225.06 | -0.051 | 275.06 | 0.175 |  | 325.06 | 0.144 | 375.06 | 0.113 |  |  |  |
| 175.08 | 0.012 | 225.08 | -0.048 | 275.08 | 0.182 |  | 325.08 | 0.136 | 375.08 | 0.118 |  |  |  |
| 175.1 | 0.015 | 225.1 | -0.036 | 275.1 | 0.185 |  | 325.1 | 0.155 | 375.1 | 0.133 |  |  |  |
| 175.12 | 0.018 | 225.12 | -0.022 | 275.12 | 0.188 |  | 325.12 | 0.217 | 375.12 | 0.142 |  |  |  |
| 175.14 | 0.022 | 225.14 | -0.012 | 275.14 | 0.181 |  | 325.14 | 0.214 | 375.14 | 0.144 |  |  |  |
| 175.16 | 0.029 | 225.16 | -0.009 | 275.16 | 0.184 |  | 325.16 | 0.192 | 375.16 | 0.145 |  |  |  |
| 175.18 | 0.037 | 225.18 | -0.012 | 275.18 | 0.177 |  | 325.18 | 0.118 | 375.18 | 0.144 |  |  |  |
| 175.2 | 0.044 | 225.2 | -0.02 | 275.2 | 0.165 |  | 325.2 | 0.183 | 375.2 | 0.14 |  |  |  |
| 175.22 | 0.048 | 225.22 | -0.026 | 275.22 | 0.01 |  | 325.22 | 0.215 | 375.22 | 0.135 |  |  |  |
| 175.24 | 0.054 | 225.24 | -0.032 | 275.24 | 0.025 |  | 325.24 | 0.233 | 375.24 | 0.129 |  |  |  |
| 175.26 | 0.011 | 225.26 | -0.02 | 275.26 | 0.026 |  | 325.26 | 0.233 | 375.26 | 0.148 |  |  |  |
| 175.28 | 0.027 | 225.28 | -0.038 | 275.28 | 0.023 |  | 325.28 | 0.22 | 375.28 | 0.158 |  |  |  |
| 175.3 | 0.034 | 225.3 | -0.024 | 275.3 | 0.033 |  | 325.3 | 0.203 | 375.3 | 0.176 |  |  |  |
| 175.32 | 0.034 | 225.32 | -0.006 | 275.32 | 0.043 |  | 325.32 | 0.179 | 375.32 | 0.192 |  |  |  |
| 175.34 | 0.038 | 225.34 | -0.018 | 275.34 | 0 |  | 325.34 | 0.158 | 375.34 | 0.193 |  |  |  |
| 175.36 | 0.042 | 225.36 | -0.004 | 275.36 | -0.039 |  | 325.36 | 0.158 | 375.36 | 0.179 |  |  |  |
| 175.38 | 0.047 | 225.38 | 0.001 | 275.38 | -0.003 |  | 325.38 | 0.219 | 375.38 | 0.164 |  |  |  |
| 175.4 | 0.079 | 225.4 | 0.02 | 275.4 | 0.067 |  | 325.4 | 0.304 | 375.4 | 0.15 |  |  |  |
| 175.42 | 0.072 | 225.42 | 0.013 | 275.42 | 0.087 |  | 325.42 | 0.329 | 375.42 | 0.148 |  |  |  |
| 175.44 | 0.064 | 225.44 | 0.015 | 275.44 | 0.089 |  | 325.44 | 0.282 | 375.44 | 0.156 |  |  |  |
| 175.46 | 0.062 | 225.46 | 0.017 | 275.46 | 0.092 |  | 325.46 | 0.198 | 375.46 | 0.165 |  |  |  |
| 175.48 | 0.066 | 225.48 | 0.018 | 275.48 | 0.107 |  | 325.48 | 0.164 | 375.48 | 0.15 |  |  |  |
| 175.5 | 0.08 | 225.5 | 0.016 | 275.5 | 0.113 |  | 325.5 | 0.187 | 375.5 | 0.129 |  |  |  |
| 175.52 | 0.109 | 225.52 | 0.107 | 275.52 | 0.129 |  | 325.52 | 0.241 | 375.52 | 0.122 |  |  |  |
| 175.54 | 0.055 | 225.54 | 0.196 | 275.54 | 0.144 |  | 325.54 | 0.262 | 375.54 | 0.114 |  |  |  |
| 175.56 | 0.02 | 225.56 | 0.021 | 275.56 | 0.15 |  | 325.56 | 0.255 | 375.56 | 0.121 |  |  |  |
| 175.58 | 0.01 | 225.58 | 0.007 | 275.58 | 0.089 |  | 325.58 | 0.191 | 375.58 | 0.119 |  |  |  |
| 175.6 | 0.006 | 225.6 | 0.009 | 275.6 | 0.076 |  | 325.6 | 0.146 | 375.6 | 0.119 |  |  |  |
| 175.62 | -0.004 | 225.62 | 0.014 | 275.62 | 0.1 |  | 325.62 | 0.135 | 375.62 | 0.122 |  |  |  |
| 175.64 | 0.007 | 225.64 | 0.032 | 275.64 | 0.017 |  | 325.64 | 0.107 | 375.64 | 0.127 |  |  |  |
| 175.66 | 0.012 | 225.66 | 0.02 | 275.66 | 0.133 |  | 325.66 | 0.09 | 375.66 | 0.134 |  |  |  |
| 175.68 | 0.004 | 225.68 | 0.025 | 275.68 | 0.128 |  | 325.68 | 0.067 | 375.68 | 0.147 |  |  |  |
| 175.7 | 0 | 225.7 | 0.029 | 275.7 | 0.184 |  | 325.7 | 0.067 | 375.7 | 0.159 |  |  |  |
| 175.72 | -0.003 | 225.72 | 0.03 | 275.72 | 0.204 |  | 325.72 | 0.085 | 375.72 | 0.165 |  |  |  |
| 175.74 | 0.001 | 225.74 | 0.023 | 275.74 | 0.208 |  | 325.74 | 0.098 | 375.74 | 0.169 |  |  |  |
| 175.76 | 0.001 | 225.76 | 0.01 | 275.76 | 0.214 |  | 325.76 | 0.13 | 375.76 | 0.169 |  |  |  |
| 175.78 | 0.006 | 225.78 | 0 | 275.78 | 0.217 |  | 325.78 | 0.142 | 375.78 | 0.171 |  |  |  |
| 175.8 | 0.023 | 225.8 | -0.007 | 275.8 | 0.215 |  | 325.8 | 0.149 | 375.8 | 0.143 |  |  |  |
| 175.82 | 0.011 | 225.82 | -0.009 | 275.82 | 0.218 |  | 325.82 | 0.157 | 375.82 | 0.229 |  |  |  |
| 175.84 | 0.002 | 225.84 | -0.011 | 275.84 | 0.211 |  | 325.84 | 0.168 | 375.84 | 0.252 |  |  |  |
| 175.86 | -0.005 | 225.86 | -0.013 | 275.86 | 0.208 |  | 325.86 | 0.168 | 375.86 | 0.241 |  |  |  |
| 175.88 | 0.003 | 225.88 | -0.022 | 275.88 | 0.205 |  | 325.88 | 0.171 | 375.88 | 0.206 |  |  |  |
| 175.9 | 0.028 | 225.9 | -0.039 | 275.9 | 0.192 |  | 325.9 | 0.17 | 375.9 | 0.19 |  |  |  |
| 175.92 | 0.022 | 225.92 | -0.041 | 275.92 | 0.185 |  | 325.92 | 0.17 | 375.92 | 0.178 |  |  |  |
| 175.94 | 0.022 | 225.94 | -0.03 | 275.94 | 0.183 |  | 325.94 | 0.17 | 375.94 | 0.174 |  |  |  |
| 175.96 | 0.057 | 225.96 | -0.025 | 275.96 | 0.2 |  | 325.96 | 0.163 | 375.96 | 0.172 |  |  |  |
| 175.98 | 0.028 | 225.98 | -0.016 | 275.98 | 0.203 |  | 325.98 | 0.156 | 375.98 | 0.167 |  |  |  |
| 176 | 0.033 | 226 | -0.003 | 276 | 0.202 |  | 326 | 0.144 | 376 | 0.16 |  |  |  |
| 176.02 | 0.033 | 226.02 | 0.026 | 276.02 | 0.004 |  | 326.02 | 0.135 | 376.02 | 0.157 |  |  |  |
| 176.04 | 0.035 | 226.04 | 0.081 | 276.04 | 0.018 |  | 326.04 | 0.131 | 376.04 | 0.154 |  |  |  |
| 176.06 | 0.037 | 226.06 | 0.229 | 276.06 | 0.029 |  | 326.06 | 0.136 | 376.06 | 0.15 |  |  |  |
| 176.08 | 0.04 | 226.08 | 0.218 | 276.08 | 0.029 |  | 326.08 | 0.136 | 376.08 | 0.146 |  |  |  |
| 176.1 | 0.04 | 226.1 | 0.249 | 276.1 | 0.045 |  | 326.1 | 0.134 | 376.1 | 0.142 |  |  |  |
| 176.12 | 0.027 | 226.12 | 0.175 | 276.12 | 0.05 |  | 326.12 | 0.128 | 376.12 | 0.128 |  |  |  |
| 176.14 | 0.054 | 226.14 | 0.037 | 276.14 | 0.055 |  | 326.14 | 0.121 | 376.14 | 0.119 |  |  |  |
| 176.16 | 0.061 | 226.16 | 0.004 | 276.16 | 0.06 |  | 326.16 | 0.126 | 376.16 | 0.116 |  |  |  |
| 176.18 | 0.061 | 226.18 | 0.004 | 276.18 | 0.066 |  | 326.18 | 0.126 | 376.18 | 0.121 |  |  |  |
| 176.2 | 0.06 | 226.2 | 0.007 | 276.2 | 0.075 |  | 326.2 | 0.127 | 376.2 | 0.133 |  |  |  |
| 176.22 | 0.058 | 226.22 | 0.01 | 276.22 | 0.093 |  | 326.22 | 0.127 | 376.22 | 0.133 |  |  |  |
| 176.24 | 0.053 | 226.24 | 0.009 | 276.24 | 0.088 |  | 326.24 | 0.127 | 376.24 | 0.137 |  |  |  |
| 176.26 | 0.052 | 226.26 | 0.004 | 276.26 | 0.107 |  | 326.26 | 0.125 | 376.26 | 0.138 |  |  |  |
| 176.28 | 0.073 | 226.28 | 0 | 276.28 | 0.128 |  | 326.28 | 0.121 | 376.28 | 0.137 |  |  |  |
| 176.3 | 0.08 | 226.3 | 0.015 | 276.3 | 0.136 |  | 326.3 | 0.104 | 376.3 | 0.135 |  |  |  |
| 176.32 | 0.125 | 226.32 | 0.024 | 276.32 | 0.136 |  | 326.32 | 0.103 | 376.32 | 0.133 |  |  |  |
| 176.34 | 0.101 | 226.34 | 0.041 | 276.34 | 0.14 |  | 326.34 | 0.121 | 376.34 | 0.132 |  |  |  |
| 176.36 | 0.082 | 226.36 | 0.034 | 276.36 | 0.144 |  | 326.36 | 0.117 | 376.36 | 0.136 |  |  |  |
| 176.38 | 0.256 | 226.38 | 0.023 | 276.38 | 0.157 |  | 326.38 | 0.118 | 376.38 | 0.145 |  |  |  |
| 176.4 | 0.173 | 226.4 | 0.015 | 276.4 | 0.187 |  | 326.4 | 0.116 | 376.4 | 0.153 |  |  |  |
| 176.42 | 0.198 | 226.42 | 0.01 | 276.42 | 0.194 |  | 326.42 | 0.121 | 376.42 | 0.148 |  |  |  |
| 176.44 | 0.207 | 226.44 | 0.01 | 276.44 | 0.182 |  | 326.44 | 0.13 | 376.44 | 0.13 |  |  |  |
| 176.46 | 0.197 | 226.46 | 0.021 | 276.46 | 0.182 |  | 326.46 | 0.158 | 376.46 | 0.139 |  |  |  |
| 176.48 | 0.23 | 226.48 | 0.02 | 276.48 | 0.154 |  | 326.48 | 0.189 | 376.48 | 0.14 |  |  |  |
| 176.5 | 0.245 | 226.5 | 0.006 | 276.5 | 0.063 |  | 326.5 | 0.217 | 376.5 | 0.127 |  |  |  |
| 176.52 | 0.106 | 226.52 | -0.021 | 276.52 | 0.058 |  | 326.52 | 0.174 | 376.52 | 0.125 |  |  |  |
| 176.54 | 0.041 | 226.54 | -0.026 | 276.54 | 0.074 |  | 326.54 | 0.217 | 376.54 | 0.131 |  |  |  |
| 176.56 | 0.021 | 226.56 | -0.023 | 276.56 | 0.078 |  | 326.56 | 0.229 | 376.56 | 0.135 |  |  |  |
| 176.58 | 0.015 | 226.58 | -0.025 | 276.58 | 0.079 |  | 326.58 | 0.247 | 376.58 | 0.137 |  |  |  |
| 176.6 | -0.011 | 226.6 | -0.02 | 276.6 | 0.099 |  | 326.6 | 0.26 | 376.6 | 0.14 |  |  |  |
| 176.62 | -0.006 | 226.62 | -0.007 | 276.62 | 0.078 |  | 326.62 | 0.266 | 376.62 | 0.141 |  |  |  |
| 176.64 | 0 | 226.64 | 0 | 276.64 | 0.047 |  | 326.64 | 0.266 | 376.64 | 0.145 |  |  |  |
| 176.66 | 0.005 | 226.66 | 0 | 276.66 | 0.056 |  | 326.66 | 0.264 | 376.66 | 0.149 |  |  |  |
| 176.68 | 0.011 | 226.68 | -0.016 | 276.68 | 0.079 |  | 326.68 | 0.258 | 376.68 | 0.144 |  |  |  |
| 176.7 | 0.013 | 226.7 | -0.038 | 276.7 | 0.09 |  | 326.7 | 0.247 | 376.7 | 0.143 |  |  |  |
| 176.72 | 0.017 | 226.72 | -0.039 | 276.72 | 0.069 |  | 326.72 | 0.232 | 376.72 | 0.159 |  |  |  |
| 176.74 | 0.018 | 226.74 | 0.005 | 276.74 | 0.111 |  | 326.74 | 0.212 | 376.74 | 0.161 |  |  |  |
| 176.76 | 0.021 | 226.76 | 0.037 | 276.76 | 0.167 |  | 326.76 | 0.196 | 376.76 | 0.15 |  |  |  |
| 176.78 | 0.024 | 226.78 | 0.118 | 276.78 | 0.192 |  | 326.78 | 0.185 | 376.78 | 0.153 |  |  |  |
| 176.8 | 0.033 | 226.8 | 0.146 | 276.8 | 0.216 |  | 326.8 | 0.179 | 376.8 | 0.156 |  |  |  |
| 176.82 | 0.022 | 226.82 | 0.212 | 276.82 | 0.183 |  | 326.82 | 0.188 | 376.82 | 0.157 |  |  |  |
| 176.84 | 0.023 | 226.84 | 0.243 | 276.84 | 0.173 |  | 326.84 | 0.181 | 376.84 | 0.162 |  |  |  |
| 176.86 | 0.022 | 226.86 | 0.273 | 276.86 | 0.214 |  | 326.86 | 0.167 | 376.86 | 0.157 |  |  |  |
| 176.88 | 0.01 | 226.88 | 0.306 | 276.88 | 0.226 |  | 326.88 | 0.165 | 376.88 | 0.174 |  |  |  |
| 176.9 | -0.001 | 226.9 | 0.279 | 276.9 | 0.227 |  | 326.9 | 0.169 | 376.9 | 0.182 |  |  |  |
| 176.92 | -0.003 | 226.92 | 0.348 | 276.92 | 0.221 |  | 326.92 | 0.168 | 376.92 | 0.192 |  |  |  |
| 176.94 | -0.002 | 226.94 | 0.23 | 276.94 | 0.201 |  | 326.94 | 0.192 | 376.94 | 0.204 |  |  |  |
| 176.96 | -0.001 | 226.96 | 0.235 | 276.96 | 0.195 |  | 326.96 | 0.247 | 376.96 | 0.262 |  |  |  |
| 176.98 | -0.006 | 226.98 | 0.222 | 276.98 | 0.193 |  | 326.98 | 0.252 | 376.98 | 0.209 |  |  |  |
| 177 | -0.011 | 227 | 0.188 | 277 | 0.202 |  | 327 | 0.23 | 377 | 0.159 |  |  |  |
| 177.02 | -0.012 | 227.02 | 0.246 | 277.02 | 0.206 |  | 327.02 | 0.227 | 377.02 | 0.168 |  |  |  |
| 177.04 | -0.007 | 227.04 | 0.223 | 277.04 | 0.205 |  | 327.04 | 0.242 | 377.04 | 0.187 |  |  |  |
| 177.06 | -0.003 | 227.06 | 0.211 | 277.06 | 0.235 |  | 327.06 | 0.245 | 377.06 | 0.214 |  |  |  |
| 177.08 | 0.003 | 227.08 | 0.196 | 277.08 | 0.238 |  | 327.08 | 0.234 | 377.08 | 0.271 |  |  |  |
| 177.1 | 0.006 | 227.1 | 0.156 | 277.1 | 0.221 |  | 327.1 | 0.224 | 377.1 | 0.254 |  |  |  |
| 177.12 | 0.008 | 227.12 | 0 | 277.12 | 0.198 |  | 327.12 | 0.217 | 377.12 | 0.188 |  |  |  |
| 177.14 | 0.009 | 227.14 | -0.023 | 277.14 | 0.194 |  | 327.14 | 0.227 | 377.14 | 0.138 |  |  |  |
| 177.16 | 0.009 | 227.16 | -0.018 | 277.16 | 0.168 |  | 327.16 | 0.212 | 377.16 | 0.118 |  |  |  |
| 177.18 | -0.001 | 227.18 | -0.007 | 277.18 | 0.128 |  | 327.18 | 0.184 | 377.18 | 0.107 |  |  |  |
| 177.2 | -0.009 | 227.2 | -0.005 | 277.2 | 0.106 |  | 327.2 | 0.141 | 377.2 | 0.101 |  |  |  |
| 177.22 | -0.001 | 227.22 | -0.015 | 277.22 | 0.119 |  | 327.22 | 0.123 | 377.22 | 0.096 |  |  |  |
| 177.24 | 0.015 | 227.24 | -0.021 | 277.24 | 0.138 |  | 327.24 | 0.114 | 377.24 | 0.095 |  |  |  |
| 177.26 | 0.041 | 227.26 | -0.019 | 277.26 | 0.234 |  | 327.26 | 0.146 | 377.26 | 0.106 |  |  |  |
| 177.28 | 0.053 | 227.28 | -0.019 | 277.28 | 0.262 |  | 327.28 | 0.194 | 377.28 | 0.177 |  |  |  |
| 177.3 | 0.067 | 227.3 | -0.036 | 277.3 | 0.245 |  | 327.3 | 0.198 | 377.3 | 0.159 |  |  |  |
| 177.32 | 0.061 | 227.32 | -0.058 | 277.32 | 0.172 |  | 327.32 | 0.202 | 377.32 | 0.149 |  |  |  |
| 177.34 | 0.045 | 227.34 | -0.071 | 277.34 | 0.128 |  | 327.34 | 0.224 | 377.34 | 0.138 |  |  |  |
| 177.36 | 0.05 | 227.36 | -0.077 | 277.36 | 0.11 |  | 327.36 | 0.267 | 377.36 | 0.135 |  |  |  |
| 177.38 | 0.039 | 227.38 | -0.079 | 277.38 | 0.126 |  | 327.38 | 0.257 | 377.38 | 0.125 |  |  |  |
| 177.4 | 0.025 | 227.4 | -0.081 | 277.4 | 0.124 |  | 327.4 | 0.212 | 377.4 | 0.119 |  |  |  |
| 177.42 | 0.022 | 227.42 | -0.081 | 277.42 | 0.122 |  | 327.42 | 0.227 | 377.42 | 0.115 |  |  |  |
| 177.44 | 0.036 | 227.44 | -0.075 | 277.44 | 0.138 |  | 327.44 | 0.283 | 377.44 | 0.115 |  |  |  |
| 177.46 | 0.052 | 227.46 | -0.062 | 277.46 | 0.166 |  | 327.46 | 0.191 | 377.46 | 0.106 |  |  |  |
| 177.48 | 0.058 | 227.48 | -0.049 | 277.48 | 0.179 |  | 327.48 | 0.209 | 377.48 | 0.115 |  |  |  |
| 177.5 | 0.051 | 227.5 | -0.039 | 277.5 | 0.161 |  | 327.5 | 0.175 | 377.5 | 0.143 |  |  |  |
| 177.52 | 0.039 | 227.52 | -0.027 | 277.52 | 0.145 |  | 327.52 | 0.131 | 377.52 | 0.207 |  |  |  |
| 177.54 | 0.028 | 227.54 | 0 | 277.54 | 0.136 |  | 327.54 | 0.132 | 377.54 | 0.214 |  |  |  |
| 177.56 | 0.027 | 227.56 | 0 | 277.56 | 0.101 |  | 327.56 | 0.135 | 377.56 | 0.192 |  |  |  |
| 177.58 | 0.024 | 227.58 | -0.014 | 277.58 | 0.076 |  | 327.58 | 0.135 | 377.58 | 0.15 |  |  |  |
| 177.6 | 0.003 | 227.6 | -0.008 | 277.6 | 0.072 |  | 327.6 | 0.151 | 377.6 | 0.119 |  |  |  |
| 177.62 | 0.001 | 227.62 | 0.006 | 277.62 | 0.137 |  | 327.62 | 0.152 | 377.62 | 0.105 |  |  |  |
| 177.64 | -0.009 | 227.64 | 0.016 | 277.64 | 0.179 |  | 327.64 | 0.164 | 377.64 | 0.103 |  |  |  |
| 177.66 | -0.011 | 227.66 | 0.016 | 277.66 | 0.172 |  | 327.66 | 0.171 | 377.66 | 0.102 |  |  |  |
| 177.68 | -0.006 | 227.68 | 0.024 | 277.68 | 0.141 |  | 327.68 | 0.136 | 377.68 | 0.109 |  |  |  |
| 177.7 | 0.004 | 227.7 | 0.063 | 277.7 | 0.134 |  | 327.7 | 0.151 | 377.7 | 0.15 |  |  |  |
| 177.72 | 0.017 | 227.72 | 0.087 | 277.72 | 0.126 |  | 327.72 | 0.183 | 377.72 | 0.202 |  |  |  |
| 177.74 | 0.063 | 227.74 | 0.064 | 277.74 | 0.116 |  | 327.74 | 0.199 | 377.74 | 0.212 |  |  |  |
| 177.76 | 0.079 | 227.76 | 0.052 | 277.76 | 0.1 |  | 327.76 | 0.217 | 377.76 | 0.2 |  |  |  |
| 177.78 | 0.074 | 227.78 | 0.041 | 277.78 | 0.094 |  | 327.78 | 0.216 | 377.78 | 0.189 |  |  |  |
| 177.8 | 0.069 | 227.8 | 0.062 | 277.8 | 0.104 |  | 327.8 | 0.153 | 377.8 | 0.159 |  |  |  |
| 177.82 | 0.074 | 227.82 | 0.072 | 277.82 | 0.112 |  | 327.82 | 0.274 | 377.82 | 0.108 |  |  |  |
| 177.84 | 0.022 | 227.84 | 0.075 | 277.84 | 0.121 |  | 327.84 | 0.248 | 377.84 | 0.135 |  |  |  |
| 177.86 | 0.097 | 227.86 | 0.066 | 277.86 | 0.11 |  | 327.86 | 0.233 | 377.86 | 0.148 |  |  |  |
| 177.88 | 0.102 | 227.88 | 0.06 | 277.88 | 0.006 |  | 327.88 | 0.229 | 377.88 | 0.149 |  |  |  |
| 177.9 | 0.094 | 227.9 | 0.057 | 277.9 | 0.012 |  | 327.9 | 0.238 | 377.9 | 0.147 |  |  |  |
| 177.92 | 0.09 | 227.92 | 0.001 | 277.92 | 0.025 |  | 327.92 | 0.239 | 377.92 | 0.144 |  |  |  |
| 177.94 | 0.102 | 227.94 | -0.009 | 277.94 | 0.031 |  | 327.94 | 0.216 | 377.94 | 0.146 |  |  |  |
| 177.96 | 0.118 | 227.96 | -0.074 | 277.96 | 0.031 |  | 327.96 | 0.186 | 377.96 | 0.134 |  |  |  |
| 177.98 | 0.121 | 227.98 | -0.062 | 277.98 | 0.032 |  | 327.98 | 0.18 | 377.98 | 0.141 |  |  |  |
| 178 | 0.127 | 228 | -0.065 | 278 | 0.035 |  | 328 | 0.184 | 378 | 0.147 |  |  |  |
| 178.02 | 0.133 | 228.02 | -0.069 | 278.02 | 0.036 |  | 328.02 | 0.167 | 378.02 | 0.142 |  |  |  |
| 178.04 | 0.137 | 228.04 | -0.07 | 278.04 | 0.042 |  | 328.04 | 0.159 | 378.04 | 0.143 |  |  |  |
| 178.06 | 0.14 | 228.06 | -0.064 | 278.06 | 0.05 |  | 328.06 | 0.145 | 378.06 | 0.16 |  |  |  |
| 178.08 | 0.135 | 228.08 | -0.055 | 278.08 | 0.068 |  | 328.08 | 0.14 | 378.08 | 0.167 |  |  |  |
| 178.1 | 0.12 | 228.1 | -0.059 | 278.1 | 0.07 |  | 328.1 | 0.139 | 378.1 | 0.144 |  |  |  |
| 178.12 | 0.097 | 228.12 | -0.073 | 278.12 | 0.085 |  | 328.12 | 0.138 | 378.12 | 0.138 |  |  |  |
| 178.14 | 0.107 | 228.14 | -0.079 | 278.14 | 0.088 |  | 328.14 | 0.135 | 378.14 | 0.135 |  |  |  |
| 178.16 | 0.143 | 228.16 | -0.084 | 278.16 | 0.135 |  | 328.16 | 0.088 | 378.16 | 0.131 |  |  |  |
| 178.18 | 0.155 | 228.18 | -0.045 | 278.18 | 0.041 |  | 328.18 | 0.131 | 378.18 | 0.104 |  |  |  |
| 178.2 | 0.15 | 228.2 | -0.039 | 278.2 | 0.036 |  | 328.2 | 0.145 | 378.2 | 0.153 |  |  |  |
| 178.22 | 0.159 | 228.22 | -0.032 | 278.22 | 0.042 |  | 328.22 | 0.165 | 378.22 | 0.154 |  |  |  |
| 178.24 | 0.184 | 228.24 | -0.037 | 278.24 | 0.064 |  | 328.24 | 0.185 | 378.24 | 0.15 |  |  |  |
| 178.26 | 0.219 | 228.26 | -0.045 | 278.26 | 0.086 |  | 328.26 | 0.196 | 378.26 | 0.148 |  |  |  |
| 178.28 | 0.214 | 228.28 | -0.036 | 278.28 | 0.13 |  | 328.28 | 0.196 | 378.28 | 0.139 |  |  |  |
| 178.3 | 0.189 | 228.3 | -0.039 | 278.3 | 0.121 |  | 328.3 | 0.191 | 378.3 | 0.14 |  |  |  |
| 178.32 | 0.148 | 228.32 | 0 | 278.32 | 0.047 |  | 328.32 | 0.181 | 378.32 | 0.142 |  |  |  |
| 178.34 | 0.165 | 228.34 | 0.006 | 278.34 | -0.002 |  | 328.34 | 0.176 | 378.34 | 0.147 |  |  |  |
| 178.36 | 0.192 | 228.36 | 0.014 | 278.36 | -0.019 |  | 328.36 | 0.189 | 378.36 | 0.149 |  |  |  |
| 178.38 | 0.188 | 228.38 | 0.015 | 278.38 | -0.025 |  | 328.38 | 0.204 | 378.38 | 0.148 |  |  |  |
| 178.4 | 0.166 | 228.4 | 0.011 | 278.4 | -0.013 |  | 328.4 | 0.217 | 378.4 | 0.157 |  |  |  |
| 178.42 | 0.137 | 228.42 | 0.004 | 278.42 | 0.004 |  | 328.42 | 0.214 | 378.42 | 0.148 |  |  |  |
| 178.44 | 0.13 | 228.44 | 0.005 | 278.44 | 0.019 |  | 328.44 | 0.216 | 378.44 | 0.135 |  |  |  |
| 178.46 | 0.124 | 228.46 | 0.01 | 278.46 | 0.035 |  | 328.46 | 0.212 | 378.46 | 0.131 |  |  |  |
| 178.48 | 0.115 | 228.48 | 0.014 | 278.48 | 0.043 |  | 328.48 | 0.193 | 378.48 | 0.129 |  |  |  |
| 178.5 | 0.105 | 228.5 | 0.015 | 278.5 | 0.044 |  | 328.5 | 0.193 | 378.5 | 0.117 |  |  |  |
| 178.52 | 0.097 | 228.52 | 0.02 | 278.52 | 0.049 |  | 328.52 | 0.191 | 378.52 | 0.121 |  |  |  |
| 178.54 | 0.103 | 228.54 | 0.026 | 278.54 | 0.054 |  | 328.54 | 0.168 | 378.54 | 0.157 |  |  |  |
| 178.56 | 0.114 | 228.56 | 0.035 | 278.56 | 0.08 |  | 328.56 | 0.171 | 378.56 | 0.158 |  |  |  |
| 178.58 | 0.117 | 228.58 | 0.038 | 278.58 | 0.081 |  | 328.58 | 0.183 | 378.58 | 0.158 |  |  |  |
| 178.6 | 0.11 | 228.6 | 0.041 | 278.6 | 0.104 |  | 328.6 | 0.176 | 378.6 | 0.156 |  |  |  |
| 178.62 | 0.186 | 228.62 | 0.039 | 278.62 | 0.102 |  | 328.62 | 0.168 | 378.62 | 0.157 |  |  |  |
| 178.64 | 0.226 | 228.64 | 0.035 | 278.64 | 0.096 |  | 328.64 | 0.172 | 378.64 | 0.155 |  |  |  |
| 178.66 | 0.184 | 228.66 | 0.04 | 278.66 | 0.086 |  | 328.66 | 0.165 | 378.66 | 0.147 |  |  |  |
| 178.68 | 0.14 | 228.68 | 0.04 | 278.68 | 0.125 |  | 328.68 | 0.157 | 378.68 | 0.169 |  |  |  |
| 178.7 | 0.14 | 228.7 | 0.041 | 278.7 | 0.119 |  | 328.7 | 0.149 | 378.7 | 0.181 |  |  |  |
| 178.72 | 0.182 | 228.72 | 0.042 | 278.72 | 0.112 |  | 328.72 | 0.156 | 378.72 | 0.206 |  |  |  |
| 178.74 | 0.152 | 228.74 | 0.028 | 278.74 | 0.099 |  | 328.74 | 0.163 | 378.74 | 0.23 |  |  |  |
| 178.76 | 0.142 | 228.76 | 0.037 | 278.76 | 0.096 |  | 328.76 | 0.173 | 378.76 | 0.228 |  |  |  |
| 178.78 | 0.15 | 228.78 | 0.057 | 278.78 | 0.093 |  | 328.78 | 0.19 | 378.78 | 0.222 |  |  |  |
| 178.8 | 0.158 | 228.8 | 0.058 | 278.8 | 0.106 |  | 328.8 | 0.192 | 378.8 | 0.206 |  |  |  |
| 178.82 | 0.145 | 228.82 | 0.054 | 278.82 | 0.112 |  | 328.82 | 0.199 | 378.82 | 0.201 |  |  |  |
| 178.84 | 0.12 | 228.84 | 0.05 | 278.84 | 0.13 |  | 328.84 | 0.187 | 378.84 | 0.209 |  |  |  |
| 178.86 | 0.085 | 228.86 | 0.037 | 278.86 | 0.132 |  | 328.86 | 0.159 | 378.86 | 0.208 |  |  |  |
| 178.88 | 0.08 | 228.88 | 0.024 | 278.88 | 0.114 |  | 328.88 | 0.158 | 378.88 | 0.207 |  |  |  |
| 178.9 | 0.246 | 228.9 | 0.009 | 278.9 | 0.114 |  | 328.9 | 0.18 | 378.9 | 0.208 |  |  |  |
| 178.92 | 0.203 | 228.92 | -0.002 | 278.92 | 0.119 |  | 328.92 | 0.185 | 378.92 | 0.204 |  |  |  |
| 178.94 | 0.14 | 228.94 | -0.016 | 278.94 | 0.119 |  | 328.94 | 0.182 | 378.94 | 0.189 |  |  |  |
| 178.96 | 0.13 | 228.96 | -0.025 | 278.96 | 0.109 |  | 328.96 | 0.18 | 378.96 | 0.156 |  |  |  |
| 178.98 | 0.142 | 228.98 | -0.028 | 278.98 | 0.102 |  | 328.98 | 0.165 | 378.98 | 0.176 |  |  |  |
| 179 | 0.155 | 229 | -0.03 | 279 | 0.1 |  | 329 | 0.181 | 379 | 0.202 |  |  |  |
| 179.02 | 0.168 | 229.02 | -0.022 | 279.02 | 0.003 |  | 329.02 | 0.183 | 379.02 | 0.211 |  |  |  |
| 179.04 | 0.176 | 229.04 | -0.019 | 279.04 | 0.018 |  | 329.04 | 0.184 | 379.04 | 0.212 |  |  |  |
| 179.06 | 0.18 | 229.06 | -0.012 | 279.06 | 0.041 |  | 329.06 | 0.184 | 379.06 | 0.208 |  |  |  |
| 179.08 | 0.169 | 229.08 | -0.003 | 279.08 | 0.054 |  | 329.08 | 0.188 | 379.08 | 0.205 |  |  |  |
| 179.1 | 0.162 | 229.1 | -0.004 | 279.1 | 0.085 |  | 329.1 | 0.193 | 379.1 | 0.204 |  |  |  |
| 179.12 | 0.155 | 229.12 | 0.026 | 279.12 | 0.103 |  | 329.12 | 0.189 | 379.12 | 0.206 |  |  |  |
| 179.14 | 0.151 | 229.14 | 0.006 | 279.14 | 0.112 |  | 329.14 | 0.179 | 379.14 | 0.217 |  |  |  |
| 179.16 | 0.153 | 229.16 | -0.003 | 279.16 | 0.104 |  | 329.16 | 0.164 | 379.16 | 0.216 |  |  |  |
| 179.18 | 0.154 | 229.18 | -0.008 | 279.18 | 0.103 |  | 329.18 | 0.156 | 379.18 | 0.202 |  |  |  |
| 179.2 | 0.15 | 229.2 | 0.013 | 279.2 | 0.082 |  | 329.2 | 0.156 | 379.2 | 0.198 |  |  |  |
| 179.22 | 0.139 | 229.22 | 0.022 | 279.22 | 0.059 |  | 329.22 | 0.169 | 379.22 | 0.17 |  |  |  |
| 179.24 | 0.136 | 229.24 | 0.025 | 279.24 | 0.045 |  | 329.24 | 0.169 | 379.24 | 0.185 |  |  |  |
| 179.26 | 0.138 | 229.26 | 0.042 | 279.26 | 0.076 |  | 329.26 | 0.166 | 379.26 | 0.18 |  |  |  |
| 179.28 | 0.165 | 229.28 | 0.027 | 279.28 | 0.135 |  | 329.28 | 0.16 | 379.28 | 0.192 |  |  |  |
| 179.3 | 0.253 | 229.3 | 0.021 | 279.3 | 0.168 |  | 329.3 | 0.171 | 379.3 | 0.231 |  |  |  |
| 179.32 | 0.244 | 229.32 | 0.002 | 279.32 | 0.158 |  | 329.32 | 0.204 | 379.32 | 0.229 |  |  |  |
| 179.34 | 0.155 | 229.34 | 0.006 | 279.34 | 0.171 |  | 329.34 | 0.239 | 379.34 | 0.205 |  |  |  |
| 179.36 | 0.127 | 229.36 | 0.022 | 279.36 | 0.162 |  | 329.36 | 0.264 | 379.36 | 0.212 |  |  |  |
| 179.38 | 0.114 | 229.38 | 0.038 | 279.38 | 0.14 |  | 329.38 | 0.262 | 379.38 | 0.221 |  |  |  |
| 179.4 | 0.123 | 229.4 | 0.046 | 279.4 | 0.11 |  | 329.4 | 0.235 | 379.4 | 0.216 |  |  |  |
| 179.42 | 0.146 | 229.42 | 0.016 | 279.42 | 0.111 |  | 329.42 | 0.19 | 379.42 | 0.219 |  |  |  |
| 179.44 | 0.162 | 229.44 | 0.048 | 279.44 | 0.113 |  | 329.44 | 0.184 | 379.44 | 0.227 |  |  |  |
| 179.46 | 0.159 | 229.46 | 0.041 | 279.46 | 0.121 |  | 329.46 | 0.213 | 379.46 | 0.228 |  |  |  |
| 179.48 | 0.149 | 229.48 | 0.034 | 279.48 | 0.117 |  | 329.48 | 0.215 | 379.48 | 0.206 |  |  |  |
| 179.5 | 0.14 | 229.5 | 0.049 | 279.5 | 0.114 |  | 329.5 | 0.194 | 379.5 | 0.206 |  |  |  |
| 179.52 | 0.127 | 229.52 | 0.061 | 279.52 | 0.129 |  | 329.52 | 0.181 | 379.52 | 0.209 |  |  |  |
| 179.54 | 0.101 | 229.54 | 0.059 | 279.54 | 0.125 |  | 329.54 | 0.168 | 379.54 | 0.208 |  |  |  |
| 179.56 | 0.093 | 229.56 | 0.047 | 279.56 | 0.135 |  | 329.56 | 0.163 | 379.56 | 0.206 |  |  |  |
| 179.58 | 0.105 | 229.58 | 0.022 | 279.58 | 0.139 |  | 329.58 | 0.154 | 379.58 | 0.206 |  |  |  |
| 179.6 | 0.134 | 229.6 | 0.012 | 279.6 | 0.138 |  | 329.6 | 0.157 | 379.6 | 0.203 |  |  |  |
| 179.62 | 0.188 | 229.62 | 0.005 | 279.62 | 0.142 |  | 329.62 | 0.157 | 379.62 | 0.182 |  |  |  |
| 179.64 | 0.206 | 229.64 | 0.011 | 279.64 | 0.143 |  | 329.64 | 0.157 | 379.64 | 0.188 |  |  |  |
| 179.66 | 0.301 | 229.66 | 0.015 | 279.66 | 0.134 |  | 329.66 | 0.15 | 379.66 | 0.212 |  |  |  |
| 179.68 | 0.291 | 229.68 | 0.009 | 279.68 | 0.129 |  | 329.68 | 0.157 | 379.68 | 0.202 |  |  |  |
| 179.7 | 0.098 | 229.7 | -0.023 | 279.7 | 0.122 |  | 329.7 | 0.175 | 379.7 | 0.196 |  |  |  |
| 179.72 | 0.18 | 229.72 | -0.017 | 279.72 | 0.123 |  | 329.72 | 0.181 | 379.72 | 0.198 |  |  |  |
| 179.74 | 0.191 | 229.74 | -0.013 | 279.74 | 0.122 |  | 329.74 | 0.191 | 379.74 | 0.209 |  |  |  |
| 179.76 | 0.145 | 229.76 | -0.01 | 279.76 | 0.115 |  | 329.76 | 0.194 | 379.76 | 0.223 |  |  |  |
| 179.78 | 0.143 | 229.78 | -0.007 | 279.78 | 0.098 |  | 329.78 | 0.194 | 379.78 | 0.218 |  |  |  |
| 179.8 | 0.151 | 229.8 | 0 | 279.8 | 0.078 |  | 329.8 | 0.195 | 379.8 | 0.211 |  |  |  |
| 179.82 | 0.156 | 229.82 | -0.004 | 279.82 | 0.067 |  | 329.82 | 0.191 | 379.82 | 0.202 |  |  |  |
| 179.84 | 0.158 | 229.84 | -0.012 | 279.84 | 0.065 |  | 329.84 | 0.214 | 379.84 | 0.202 |  |  |  |
| 179.86 | 0.167 | 229.86 | -0.013 | 279.86 | 0.029 |  | 329.86 | 0.219 | 379.86 | 0.2 |  |  |  |
| 179.88 | 0.164 | 229.88 | -0.004 | 279.88 | 0.059 |  | 329.88 | 0.223 | 379.88 | 0.204 |  |  |  |
| 179.9 | 0.172 | 229.9 | 0.014 | 279.9 | 0.096 |  | 329.9 | 0.219 | 379.9 | 0.2 |  |  |  |
| 179.92 | 0.165 | 229.92 | 0.032 | 279.92 | 0.108 |  | 329.92 | 0.215 | 379.92 | 0.186 |  |  |  |
| 179.94 | 0.15 | 229.94 | 0.031 | 279.94 | 0.098 |  | 329.94 | 0.215 | 379.94 | 0.173 |  |  |  |
| 179.96 | 0.14 | 229.96 | 0.019 | 279.96 | 0.086 |  | 329.96 | 0.202 | 379.96 | 0.186 |  |  |  |
| 179.98 | 0.135 | 229.98 | -0.001 | 279.98 | 0.124 |  | 329.98 | 0.201 | 379.98 | 0.2 |  |  |  |
| 180 | 0.134 | 230 | -0.005 | 280 | 0.236 |  | 330 | 0.2 | 380 | 0.22 |  |  |  |
| 180.02 | 0.146 | 230.02 | -0.006 | 280.02 | 0.237 |  | 330.02 | 0.218 | 380.02 | 0.184 |  |  |  |
| 180.04 | 0.157 | 230.04 | 0 | 280.04 | 0.15 |  | 330.04 | 0.226 | 380.04 | 0.181 |  |  |  |
| 180.06 | 0.138 | 230.06 | 0.006 | 280.06 | 0.111 |  | 330.06 | 0.233 | 380.06 | 0.199 |  |  |  |
| 180.08 | 0.121 | 230.08 | 0.019 | 280.08 | 0.098 |  | 330.08 | 0.23 | 380.08 | 0.209 |  |  |  |
| 180.1 | 0.127 | 230.1 | 0.039 | 280.1 | 0.077 |  | 330.1 | 0.227 | 380.1 | 0.207 |  |  |  |
| 180.12 | 0.178 | 230.12 | 0.039 | 280.12 | 0.073 |  | 330.12 | 0.226 | 380.12 | 0.206 |  |  |  |
| 180.14 | 0.278 | 230.14 | 0.052 | 280.14 | 0.078 |  | 330.14 | 0.229 | 380.14 | 0.202 |  |  |  |
| 180.16 | 0.304 | 230.16 | 0.07 | 280.16 | 0.08 |  | 330.16 | 0.228 | 380.16 | 0.177 |  |  |  |
| 180.18 | 0.243 | 230.18 | 0.075 | 280.18 | 0.076 |  | 330.18 | 0.23 | 380.18 | 0.182 |  |  |  |
| 180.2 | 0.16 | 230.2 | 0.084 | 280.2 | 0.069 |  | 330.2 | 0.237 | 380.2 | 0.191 |  |  |  |
| 180.22 | 0.139 | 230.22 | 0.109 | 280.22 | 0.071 |  | 330.22 | 0.25 | 380.22 | 0.182 |  |  |  |
| 180.24 | 0.134 | 230.24 | 0.115 | 280.24 | 0.084 |  | 330.24 | 0.238 | 380.24 | 0.185 |  |  |  |
| 180.26 | 0.138 | 230.26 | 0.098 | 280.26 | 0.084 |  | 330.26 | 0.24 | 380.26 | 0.195 |  |  |  |
| 180.28 | 0.132 | 230.28 | 0.073 | 280.28 | 0.084 |  | 330.28 | 0.238 | 380.28 | 0.216 |  |  |  |
| 180.3 | 0.131 | 230.3 | 0.076 | 280.3 | 0.083 |  | 330.3 | 0.202 | 380.3 | 0.212 |  |  |  |
| 180.32 | 0.14 | 230.32 | 0.071 | 280.32 | 0.17 |  | 330.32 | 0.211 | 380.32 | 0.21 |  |  |  |
| 180.34 | 0.16 | 230.34 | 0.089 | 280.34 | 0.182 |  | 330.34 | 0.208 | 380.34 | 0.207 |  |  |  |
| 180.36 | 0.185 | 230.36 | 0.095 | 280.36 | 0.174 |  | 330.36 | 0.195 | 380.36 | 0.19 |  |  |  |
| 180.38 | 0.178 | 230.38 | 0.087 | 280.38 | 0.181 |  | 330.38 | 0.207 | 380.38 | 0.205 |  |  |  |
| 180.4 | 0.125 | 230.4 | 0.08 | 280.4 | 0.191 |  | 330.4 | 0.25 | 380.4 | 0.197 |  |  |  |
| 180.42 | 0.074 | 230.42 | 0.069 | 280.42 | 0.183 |  | 330.42 | 0.26 | 380.42 | 0.195 |  |  |  |
| 180.44 | 0.06 | 230.44 | 0.052 | 280.44 | 0.193 |  | 330.44 | 0.255 | 380.44 | 0.201 |  |  |  |
| 180.46 | 0.057 | 230.46 | 0.045 | 280.46 | 0.198 |  | 330.46 | 0.266 | 380.46 | 0.208 |  |  |  |
| 180.48 | 0.072 | 230.48 | 0.054 | 280.48 | 0.222 |  | 330.48 | 0.264 | 380.48 | 0.211 |  |  |  |
| 180.5 | 0.125 | 230.5 | 0.059 | 280.5 | 0.18 |  | 330.5 | 0.219 | 380.5 | 0.215 |  |  |  |
| 180.52 | 0.157 | 230.52 | 0.04 | 280.52 | 0.125 |  | 330.52 | 0.173 | 380.52 | 0.217 |  |  |  |
| 180.54 | 0.193 | 230.54 | 0.01 | 280.54 | 0.131 |  | 330.54 | 0.178 | 380.54 | 0.213 |  |  |  |
| 180.56 | 0.19 | 230.56 | -0.003 | 280.56 | 0.17 |  | 330.56 | 0.177 | 380.56 | 0.215 |  |  |  |
| 180.58 | 0.181 | 230.58 | -0.009 | 280.58 | 0.169 |  | 330.58 | 0.199 | 380.58 | 0.216 |  |  |  |
| 180.6 | 0.196 | 230.6 | -0.013 | 280.6 | 0.167 |  | 330.6 | 0.206 | 380.6 | 0.226 |  |  |  |
| 180.62 | 0.341 | 230.62 | -0.01 | 280.62 | 0.162 |  | 330.62 | 0.217 | 380.62 | 0.222 |  |  |  |
| 180.64 | 0.2832 | 230.64 | -0.012 | 280.64 | 0.145 |  | 330.64 | 0.232 | 380.64 | 0.222 |  |  |  |
| 180.66 | 0.2778 | 230.66 | -0.025 | 280.66 | 0.132 |  | 330.66 | 0.252 | 380.66 | 0.215 |  |  |  |
| 180.68 | 0.343 | 230.68 | -0.033 | 280.68 | 0.131 |  | 330.68 | 0.272 | 380.68 | 0.202 |  |  |  |
| 180.7 | 0.182 | 230.7 | -0.024 | 280.7 | 0.131 |  | 330.7 | 0.272 | 380.7 | 0.194 |  |  |  |
| 180.72 | 0.159 | 230.72 | -0.023 | 280.72 | 0.132 |  | 330.72 | 0.228 | 380.72 | 0.216 |  |  |  |
| 180.74 | 0.165 | 230.74 | -0.027 | 280.74 | 0.125 |  | 330.74 | 0.136 | 380.74 | 0.22 |  |  |  |
| 180.76 | 0.167 | 230.76 | -0.029 | 280.76 | 0.118 |  | 330.76 | 0.117 | 380.76 | 0.215 |  |  |  |
| 180.78 | 0.163 | 230.78 | -0.029 | 280.78 | 0.109 |  | 330.78 | 0.136 | 380.78 | 0.214 |  |  |  |
| 180.8 | 0.142 | 230.8 | -0.033 | 280.8 | 0.106 |  | 330.8 | 0.156 | 380.8 | 0.21 |  |  |  |
| 180.82 | 0.118 | 230.82 | -0.042 | 280.82 | 0.081 |  | 330.82 | 0.173 | 380.82 | 0.215 |  |  |  |
| 180.84 | 0.124 | 230.84 | -0.033 | 280.84 | 0.104 |  | 330.84 | 0.175 | 380.84 | 0.217 |  |  |  |
| 180.86 | 0.133 | 230.86 | -0.015 | 280.86 | 0.111 |  | 330.86 | 0.156 | 380.86 | 0.196 |  |  |  |
| 180.88 | 0.134 | 230.88 | 0.005 | 280.88 | 0.11 |  | 330.88 | 0.154 | 380.88 | 0.19 |  |  |  |
| 180.9 | 0.133 | 230.9 | 0.019 | 280.9 | 0.106 |  | 330.9 | 0.137 | 380.9 | 0.181 |  |  |  |
| 180.92 | 0.133 | 230.92 | 0.036 | 280.92 | 0.102 |  | 330.92 | 0.12 | 380.92 | 0.191 |  |  |  |
| 180.94 | 0.148 | 230.94 | 0.042 | 280.94 | 0.104 |  | 330.94 | 0.121 | 380.94 | 0.194 |  |  |  |
| 180.96 | 0.191 | 230.96 | 0.054 | 280.96 | 0.111 |  | 330.96 | 0.079 | 380.96 | 0.192 |  |  |  |
| 180.98 | 0.339 | 230.98 | 0.045 | 280.98 | 0.128 |  | 330.98 | 0.111 | 380.98 | 0.186 |  |  |  |
| 181 | 0.347 | 231 | 0.047 | 281 | 0.151 |  | 331 | 0.18 | 381 | 0.174 |  |  |  |
| 181.02 | 0.329 | 231.02 | 0.066 | 281.02 | 0.165 |  | 331.02 | 0.187 | 381.02 | 0.177 |  |  |  |
| 181.04 | 0.177 | 231.04 | 0.097 | 281.04 | 0.154 |  | 331.04 | 0.161 | 381.04 | 0.182 |  |  |  |
| 181.06 | 0.128 | 231.06 | 0.067 | 281.06 | 0.14 |  | 331.06 | 0.095 | 381.06 | 0.181 |  |  |  |
| 181.08 | 0.162 | 231.08 | 0.075 | 281.08 | 0.128 |  | 331.08 | 0.154 | 381.08 | 0.192 |  |  |  |
| 181.1 | 0.172 | 231.1 | 0.065 | 281.1 | 0.128 |  | 331.1 | 0.171 | 381.1 | 0.188 |  |  |  |
| 181.12 | 0.154 | 231.12 | 0.063 | 281.12 | 0.114 |  | 331.12 | 0.223 | 381.12 | 0.183 |  |  |  |
| 181.14 | 0.138 | 231.14 | 0.04 | 281.14 | 0.11 |  | 331.14 | 0.218 | 381.14 | 0.192 |  |  |  |
| 181.16 | 0.14 | 231.16 | 0.045 | 281.16 | 0.106 |  | 331.16 | 0.204 | 381.16 | 0.186 |  |  |  |
| 181.18 | 0.108 | 231.18 | 0.062 | 281.18 | 0.116 |  | 331.18 | 0.203 | 381.18 | 0.194 |  |  |  |
| 181.2 | 0.102 | 231.2 | 0.038 | 281.2 | 0.118 |  | 331.2 | 0.226 | 381.2 | 0.197 |  |  |  |
| 181.22 | 0.117 | 231.22 | 0.015 | 281.22 | 0.11 |  | 331.22 | 0.033 | 381.22 | 0.197 |  |  |  |
| 181.24 | 0.108 | 231.24 | -0.011 | 281.24 | 0.093 |  | 331.24 | 0.067 | 381.24 | 0.194 |  |  |  |
| 181.26 | 0.084 | 231.26 | 0.008 | 281.26 | 0.081 |  | 331.26 | 0.074 | 381.26 | 0.195 |  |  |  |
| 181.28 | 0.087 | 231.28 | 0.024 | 281.28 | 0.07 |  | 331.28 | 0.075 | 381.28 | 0.192 |  |  |  |
| 181.3 | 0.125 | 231.3 | 0.022 | 281.3 | 0.061 |  | 331.3 | 0.081 | 381.3 | 0.192 |  |  |  |
| 181.32 | 0.15 | 231.32 | 0.045 | 281.32 | 0.052 |  | 331.32 | 0.089 | 381.32 | 0.197 |  |  |  |
| 181.34 | 0.214 | 231.34 | 0.04 | 281.34 | 0.043 |  | 331.34 | 0.09 | 381.34 | 0.204 |  |  |  |
| 181.36 | 0.237 | 231.36 | 0.035 | 281.36 | 0.035 |  | 331.36 | 0.094 | 381.36 | 0.203 |  |  |  |
| 181.38 | 0.21 | 231.38 | 0.021 | 281.38 | 0.036 |  | 331.38 | 0.102 | 381.38 | 0.2 |  |  |  |
| 181.4 | 0.278 | 231.4 | 0.016 | 281.4 | 0.035 |  | 331.4 | 0.104 | 381.4 | 0.198 |  |  |  |
| 181.42 | 0.361 | 231.42 | 0.007 | 281.42 | 0.029 |  | 331.42 | 0.117 | 381.42 | 0.202 |  |  |  |
| 181.44 | 0.376 | 231.44 | 0.014 | 281.44 | 0.038 |  | 331.44 | 0.122 | 381.44 | 0.188 |  |  |  |
| 181.46 | 0.287 | 231.46 | 0.017 | 281.46 | 0.034 |  | 331.46 | 0.124 | 381.46 | 0.178 |  |  |  |
| 181.48 | 0.167 | 231.48 | 0.005 | 281.48 | 0.032 |  | 331.48 | 0.124 | 381.48 | 0.173 |  |  |  |
| 181.5 | 0.164 | 231.5 | -0.013 | 281.5 | 0.03 |  | 331.5 | 0.13 | 381.5 | 0.178 |  |  |  |
| 181.52 | 0.177 | 231.52 | 0.001 | 281.52 | 0.026 |  | 331.52 | 0.148 | 381.52 | 0.196 |  |  |  |
| 181.54 | 0.188 | 231.54 | 0.014 | 281.54 | 0.028 |  | 331.54 | 0.16 | 381.54 | 0.189 |  |  |  |
| 181.56 | 0.182 | 231.56 | 0.008 | 281.56 | 0.029 |  | 331.56 | 0.155 | 381.56 | 0.186 |  |  |  |
| 181.58 | 0.187 | 231.58 | -0.004 | 281.58 | 0.028 |  | 331.58 | 0.171 | 381.58 | 0.186 |  |  |  |
| 181.6 | 0.169 | 231.6 | -0.012 | 281.6 | 0.047 |  | 331.6 | 0.186 | 381.6 | 0.202 |  |  |  |
| 181.62 | 0.174 | 231.62 | -0.015 | 281.62 | 0.045 |  | 331.62 | 0.189 | 381.62 | 0.203 |  |  |  |
| 181.64 | 0.132 | 231.64 | 0.014 | 281.64 | 0.04 |  | 331.64 | 0.195 | 381.64 | 0.203 |  |  |  |
| 181.66 | 0.144 | 231.66 | 0.028 | 281.66 | 0.067 |  | 331.66 | 0.2 | 381.66 | 0.201 |  |  |  |
| 181.68 | 0.152 | 231.68 | 0.016 | 281.68 | 0.063 |  | 331.68 | 0.198 | 381.68 | 0.202 |  |  |  |
| 181.7 | 0.154 | 231.7 | 0.006 | 281.7 | 0.067 |  | 331.7 | 0.176 | 381.7 | 0.205 |  |  |  |
| 181.72 | 0.185 | 231.72 | 0.011 | 281.72 | 0.064 |  | 331.72 | 0.216 | 381.72 | 0.193 |  |  |  |
| 181.74 | 0.222 | 231.74 | 0.016 | 281.74 | 0.063 |  | 331.74 | 0.219 | 381.74 | 0.187 |  |  |  |
| 181.76 | 0.212 | 231.76 | 0.018 | 281.76 | 0.063 |  | 331.76 | 0.208 | 381.76 | 0.193 |  |  |  |
| 181.78 | 0.173 | 231.78 | 0.037 | 281.78 | 0.06 |  | 331.78 | 0.2 | 381.78 | 0.198 |  |  |  |
| 181.8 | 0.169 | 231.8 | 0.097 | 281.8 | 0.054 |  | 331.8 | 0.207 | 381.8 | 0.197 |  |  |  |
| 181.82 | 0.178 | 231.82 | 0.115 | 281.82 | 0.054 |  | 331.82 | 0.208 | 381.82 | 0.184 |  |  |  |
| 181.84 | 0.181 | 231.84 | 0.108 | 281.84 | 0.05 |  | 331.84 | 0.212 | 381.84 | 0.194 |  |  |  |
| 181.86 | 0.162 | 231.86 | 0.067 | 281.86 | 0 |  | 331.86 | 0.208 | 381.86 | 0.195 |  |  |  |
| 181.88 | 0.129 | 231.88 | 0.064 | 281.88 | 0.021 |  | 331.88 | 0.189 | 381.88 | 0.204 |  |  |  |
| 181.9 | 0.102 | 231.9 | 0.056 | 281.9 | 0.044 |  | 331.9 | 0.197 | 381.9 | 0.211 |  |  |  |
| 181.92 | 0.116 | 231.92 | 0.04 | 281.92 | 0.043 |  | 331.92 | 0.209 | 381.92 | 0.191 |  |  |  |
| 181.94 | 0.103 | 231.94 | 0.023 | 281.94 | 0.048 |  | 331.94 | 0.215 | 381.94 | 0.21 |  |  |  |
| 181.96 | 0.113 | 231.96 | 0.012 | 281.96 | 0.046 |  | 331.96 | 0.204 | 381.96 | 0.204 |  |  |  |
| 181.98 | 0.138 | 231.98 | 0.015 | 281.98 | 0.042 |  | 331.98 | 0.196 | 381.98 | 0.202 |  |  |  |
| 182 | 0.206 | 232 | 0.026 | 282 | 0.03 |  | 332 | 0.201 | 382 | 0.192 |  |  |  |
| 182.02 | 0.234 | 232.02 | 0.022 | 282.02 | 0.085 |  | 332.02 | 0.213 | 382.02 | 0.205 |  |  |  |
| 182.04 | 0.211 | 232.04 | 0.008 | 282.04 | 0.082 |  | 332.04 | 0.212 | 382.04 | 0.215 |  |  |  |
| 182.06 | 0.095 | 232.06 | -0.016 | 282.06 | 0.087 |  | 332.06 | 0.226 | 382.06 | 0.213 |  |  |  |
| 182.08 | 0.087 | 232.08 | -0.019 | 282.08 | 0.03 |  | 332.08 | 0.237 | 382.08 | 0.213 |  |  |  |
| 182.1 | 0.09 | 232.1 | -0.021 | 282.1 | 0.048 |  | 332.1 | 0.232 | 382.1 | 0.16 |  |  |  |
| 182.12 | 0.113 | 232.12 | 0.001 | 282.12 | 0.051 |  | 332.12 | 0.227 | 382.12 | 0.14 |  |  |  |
| 182.14 | 0.138 | 232.14 | 0.021 | 282.14 | 0.087 |  | 332.14 | 0.232 | 382.14 | 0.162 |  |  |  |
| 182.16 | 0.159 | 232.16 | 0.023 | 282.16 | 0.095 |  | 332.16 | 0.233 | 382.16 | 0.192 |  |  |  |
| 182.18 | 0.165 | 232.18 | 0.019 | 282.18 | 0.076 |  | 332.18 | 0.238 | 382.18 | 0.196 |  |  |  |
| 182.2 | 0.174 | 232.2 | -0.007 | 282.2 | 0.049 |  | 332.2 | 0.229 | 382.2 | 0.186 |  |  |  |
| 182.22 | 0.171 | 232.22 | -0.029 | 282.22 | 0.048 |  | 332.22 | 0.204 | 382.22 | 0.186 |  |  |  |
| 182.24 | 0.166 | 232.24 | -0.018 | 282.24 | 0.053 |  | 332.24 | 0.209 | 382.24 | 0.196 |  |  |  |
| 182.26 | 0.163 | 232.26 | -0.016 | 282.26 | 0.055 |  | 332.26 | 0.233 | 382.26 | 0.201 |  |  |  |
| 182.28 | 0.159 | 232.28 | -0.018 | 282.28 | 0.062 |  | 332.28 | 0.258 | 382.28 | 0.168 |  |  |  |
| 182.3 | 0.148 | 232.3 | 0.001 | 282.3 | 0.068 |  | 332.3 | 0.265 | 382.3 | 0.19 |  |  |  |
| 182.32 | 0.156 | 232.32 | 0.013 | 282.32 | 0.113 |  | 332.32 | 0.263 | 382.32 | 0.205 |  |  |  |
| 182.34 | 0.175 | 232.34 | 0.006 | 282.34 | 0.112 |  | 332.34 | 0.272 | 382.34 | 0.21 |  |  |  |
| 182.36 | 0.181 | 232.36 | 0.007 | 282.36 | 0.061 |  | 332.36 | 0.26 | 382.36 | 0.211 |  |  |  |
| 182.38 | 0.184 | 232.38 | 0.017 | 282.38 | 0.068 |  | 332.38 | 0.231 | 382.38 | 0.212 |  |  |  |
| 182.4 | 0.259 | 232.4 | 0.012 | 282.4 | 0.071 |  | 332.4 | 0.196 | 382.4 | 0.199 |  |  |  |
| 182.42 | 0.254 | 232.42 | 0.109 | 282.42 | 0.074 |  | 332.42 | 0.18 | 382.42 | 0.187 |  |  |  |
| 182.44 | 0.107 | 232.44 | 0.111 | 282.44 | 0.076 |  | 332.44 | 0.191 | 382.44 | 0.195 |  |  |  |
| 182.46 | 0.131 | 232.46 | 0.106 | 282.46 | 0.076 |  | 332.46 | 0.218 | 382.46 | 0.218 |  |  |  |
| 182.48 | 0.108 | 232.48 | 0.099 | 282.48 | 0.075 |  | 332.48 | 0.142 | 382.48 | 0.219 |  |  |  |
| 182.5 | 0.106 | 232.5 | 0.084 | 282.5 | 0.09 |  | 332.5 | 0.208 | 382.5 | 0.221 |  |  |  |
| 182.52 | 0.101 | 232.52 | 0.107 | 282.52 | 0.095 |  | 332.52 | 0.211 | 382.52 | 0.227 |  |  |  |
| 182.54 | 0.096 | 232.54 | 0.093 | 282.54 | 0.1 |  | 332.54 | 0.191 | 382.54 | 0.228 |  |  |  |
| 182.56 | 0.088 | 232.56 | 0.079 | 282.56 | 0.108 |  | 332.56 | 0.223 | 382.56 | 0.193 |  |  |  |
| 182.58 | 0.092 | 232.58 | 0.06 | 282.58 | 0.134 |  | 332.58 | 0.242 | 382.58 | 0.19 |  |  |  |
| 182.6 | 0.101 | 232.6 | 0.071 | 282.6 | 0.139 |  | 332.6 | 0.263 | 382.6 | 0.198 |  |  |  |
| 182.62 | 0.114 | 232.62 | 0.073 | 282.62 | 0.151 |  | 332.62 | 0.273 | 382.62 | 0.193 |  |  |  |
| 182.64 | 0.121 | 232.64 | 0.051 | 282.64 | 0.147 |  | 332.64 | 0.269 | 382.64 | 0.157 |  |  |  |
| 182.66 | 0.116 | 232.66 | 0.101 | 282.66 | 0.142 |  | 332.66 | 0.228 | 382.66 | 0.148 |  |  |  |
| 182.68 | 0.109 | 232.68 | 0.114 | 282.68 | 0.189 |  | 332.68 | 0.217 | 382.68 | 0.141 |  |  |  |
| 182.7 | 0.099 | 232.7 | 0.083 | 282.7 | 0.213 |  | 332.7 | 0.226 | 382.7 | 0.129 |  |  |  |
| 182.72 | 0.079 | 232.72 | 0.066 | 282.72 | 0.186 |  | 332.72 | 0.227 | 382.72 | 0.139 |  |  |  |
| 182.74 | 0.069 | 232.74 | 0.065 | 282.74 | 0.168 |  | 332.74 | 0.207 | 382.74 | 0.128 |  |  |  |
| 182.76 | 0.085 | 232.76 | 0.053 | 282.76 | 0.142 |  | 332.76 | 0.207 | 382.76 | 0.12 |  |  |  |
| 182.78 | 0.114 | 232.78 | 0.033 | 282.78 | 0.141 |  | 332.78 | 0.19 | 382.78 | 0.137 |  |  |  |
| 182.8 | 0.133 | 232.8 | 0.029 | 282.8 | 0.01 |  | 332.8 | 0.208 | 382.8 | 0.177 |  |  |  |
| 182.82 | 0.111 | 232.82 | 0.026 | 282.82 | -0.009 |  | 332.82 | 0.254 | 382.82 | 0.22 |  |  |  |
| 182.84 | 0.098 | 232.84 | 0.021 | 282.84 | -0.034 |  | 332.84 | 0.261 | 382.84 | 0.241 |  |  |  |
| 182.86 | 0.036 | 232.86 | 0.017 | 282.86 | -0.031 |  | 332.86 | 0.255 | 382.86 | 0.231 |  |  |  |
| 182.88 | 0.04 | 232.88 | 0.016 | 282.88 | 0 |  | 332.88 | 0.243 | 382.88 | 0.217 |  |  |  |
| 182.9 | 0.056 | 232.9 | 0.02 | 282.9 | 0.034 |  | 332.9 | 0.249 | 382.9 | 0.187 |  |  |  |
| 182.92 | 0.062 | 232.92 | 0.024 | 282.92 | 0.038 |  | 332.92 | 0.242 | 382.92 | 0.139 |  |  |  |
| 182.94 | 0.073 | 232.94 | 0.024 | 282.94 | 0.069 |  | 332.94 | 0.236 | 382.94 | 0.128 |  |  |  |
| 182.96 | 0.103 | 232.96 | 0.016 | 282.96 | 0.071 |  | 332.96 | 0.228 | 382.96 | 0.174 |  |  |  |
| 182.98 | 0.152 | 232.98 | 0.004 | 282.98 | 0.069 |  | 332.98 | 0.235 | 382.98 | 0.159 |  |  |  |
| 183 | 0.175 | 233 | -0.003 | 283 | 0.036 |  | 333 | 0.236 | 383 | 0.159 |  |  |  |
| 183.02 | 0.177 | 233.02 | 0.06 | 283.02 | 0.068 |  | 333.02 | 0.235 | 383.02 | 0.156 |  |  |  |
| 183.04 | 0.18 | 233.04 | 0.065 | 283.04 | 0.082 |  | 333.04 | 0.227 | 383.04 | 0.147 |  |  |  |
| 183.06 | 0.179 | 233.06 | 0.089 | 283.06 | 0.084 |  | 333.06 | 0.197 | 383.06 | 0.141 |  |  |  |
| 183.08 | 0.175 | 233.08 | 0.055 | 283.08 | 0.077 |  | 333.08 | 0.222 | 383.08 | 0.223 |  |  |  |
| 183.1 | 0.165 | 233.1 | 0.036 | 283.1 | 0.114 |  | 333.1 | 0.166 | 383.1 | 0.265 |  |  |  |
| 183.12 | 0.139 | 233.12 | 0.033 | 283.12 | 0.131 |  | 333.12 | 0.164 | 383.12 | 0.269 |  |  |  |
| 183.14 | 0.109 | 233.14 | 0.04 | 283.14 | 0.044 |  | 333.14 | 0.192 | 383.14 | 0.253 |  |  |  |
| 183.16 | 0.075 | 233.16 | 0.047 | 283.16 | 0.155 |  | 333.16 | 0.209 | 383.16 | 0.239 |  |  |  |
| 183.18 | 0.087 | 233.18 | 0.056 | 283.18 | 0.16 |  | 333.18 | 0.191 | 383.18 | 0.227 |  |  |  |
| 183.2 | 0.114 | 233.2 | 0.065 | 283.2 | 0.01 |  | 333.2 | 0.185 | 383.2 | 0.216 |  |  |  |
| 183.22 | 0.133 | 233.22 | 0.072 | 283.22 | 0.114 |  | 333.22 | 0.169 | 383.22 | 0.206 |  |  |  |
| 183.24 | 0.127 | 233.24 | 0.081 | 283.24 | 0.174 |  | 333.24 | 0.161 | 383.24 | 0.175 |  |  |  |
| 183.26 | 0.12 | 233.26 | 0.068 | 283.26 | 0.171 |  | 333.26 | 0.208 | 383.26 | 0.165 |  |  |  |
| 183.28 | 0.111 | 233.28 | 0.056 | 283.28 | 0.173 |  | 333.28 | 0.236 | 383.28 | 0.183 |  |  |  |
| 183.3 | 0.112 | 233.3 | 0.047 | 283.3 | 0.15 |  | 333.3 | 0.237 | 383.3 | 0.198 |  |  |  |
| 183.32 | 0.107 | 233.32 | 0.05 | 283.32 | 0.118 |  | 333.32 | 0.231 | 383.32 | 0.204 |  |  |  |
| 183.34 | 0.112 | 233.34 | 0.053 | 283.34 | 0.081 |  | 333.34 | 0.226 | 383.34 | 0.227 |  |  |  |
| 183.36 | 0.118 | 233.36 | 0.033 | 283.36 | 0.033 |  | 333.36 | 0.226 | 383.36 | 0.232 |  |  |  |
| 183.38 | 0.119 | 233.38 | 0.022 | 283.38 | 0.019 |  | 333.38 | 0.23 | 383.38 | 0.226 |  |  |  |
| 183.4 | 0.114 | 233.4 | 0.006 | 283.4 | 0.026 |  | 333.4 | 0.229 | 383.4 | 0.223 |  |  |  |
| 183.42 | 0.044 | 233.42 | -0.001 | 283.42 | 0.03 |  | 333.42 | 0.227 | 383.42 | 0.2 |  |  |  |
| 183.44 | 0.042 | 233.44 | -0.001 | 283.44 | 0.032 |  | 333.44 | 0.227 | 383.44 | 0.198 |  |  |  |
| 183.46 | 0.032 | 233.46 | -0.002 | 283.46 | 0.064 |  | 333.46 | 0.226 | 383.46 | 0.208 |  |  |  |
| 183.48 | 0.059 | 233.48 | -0.002 | 283.48 | 0.069 |  | 333.48 | 0.236 | 383.48 | 0.208 |  |  |  |
| 183.5 | 0.094 | 233.5 | 0.003 | 283.5 | 0.056 |  | 333.5 | 0.266 | 383.5 | 0.21 |  |  |  |
| 183.52 | 0.159 | 233.52 | 0.01 | 283.52 | 0.046 |  | 333.52 | 0.273 | 383.52 | 0.201 |  |  |  |
| 183.54 | 0.159 | 233.54 | 0.014 | 283.54 | 0.057 |  | 333.54 | 0.267 | 383.54 | 0.172 |  |  |  |
| 183.56 | 0.151 | 233.56 | 0.01 | 283.56 | 0.078 |  | 333.56 | 0.27 | 383.56 | 0.213 |  |  |  |
| 183.58 | 0.144 | 233.58 | 0.007 | 283.58 | 0.106 |  | 333.58 | 0.287 | 383.58 | 0.226 |  |  |  |
| 183.6 | 0.145 | 233.6 | -0.006 | 283.6 | 0.005 |  | 333.6 | 0.304 | 383.6 | 0.231 |  |  |  |
| 183.62 | 0.156 | 233.62 | -0.017 | 283.62 | 0.017 |  | 333.62 | 0.307 | 383.62 | 0.227 |  |  |  |
| 183.64 | 0.124 | 233.64 | -0.014 | 283.64 | 0.03 |  | 333.64 | 0.302 | 383.64 | 0.227 |  |  |  |
| 183.66 | 0.113 | 233.66 | -0.004 | 283.66 | 0.017 |  | 333.66 | 0.282 | 383.66 | 0.204 |  |  |  |
| 183.68 | 0.116 | 233.68 | 0.01 | 283.68 | 0.004 |  | 333.68 | 0.263 | 383.68 | 0.158 |  |  |  |
| 183.7 | 0.132 | 233.7 | 0.023 | 283.7 | 0.005 |  | 333.7 | 0.253 | 383.7 | 0.228 |  |  |  |
| 183.72 | 0.143 | 233.72 | 0.059 | 283.72 | 0.006 |  | 333.72 | 0.252 | 383.72 | 0.23 |  |  |  |
| 183.74 | 0.152 | 233.74 | 0.07 | 283.74 | 0.022 |  | 333.74 | 0.23 | 383.74 | 0.232 |  |  |  |
| 183.76 | 0.129 | 233.76 | 0.068 | 283.76 | 0.043 |  | 333.76 | 0.207 | 383.76 | 0.22 |  |  |  |
| 183.78 | 0.063 | 233.78 | 0.058 | 283.78 | 0.082 |  | 333.78 | 0.192 | 383.78 | 0.224 |  |  |  |
| 183.8 | 0.1 | 233.8 | 0.061 | 283.8 | 0.187 |  | 333.8 | 0.179 | 383.8 | 0.229 |  |  |  |
| 183.82 | 0.148 | 233.82 | 0.072 | 283.82 | 0.263 |  | 333.82 | 0.168 | 383.82 | 0.242 |  |  |  |
| 183.84 | 0.162 | 233.84 | 0.09 | 283.84 | 0.236 |  | 333.84 | 0.175 | 383.84 | 0.243 |  |  |  |
| 183.86 | 0.124 | 233.86 | 0.104 | 283.86 | 0.133 |  | 333.86 | 0.169 | 383.86 | 0.229 |  |  |  |
| 183.88 | 0.084 | 233.88 | 0.112 | 283.88 | 0.037 |  | 333.88 | 0.162 | 383.88 | 0.129 |  |  |  |
| 183.9 | 0.101 | 233.9 | 0.115 | 283.9 | 0.024 |  | 333.9 | 0.159 | 383.9 | 0.226 |  |  |  |
| 183.92 | 0.134 | 233.92 | 0.126 | 283.92 | 0.031 |  | 333.92 | 0.165 | 383.92 | 0.226 |  |  |  |
| 183.94 | 0.162 | 233.94 | 0.135 | 283.94 | 0.039 |  | 333.94 | 0.181 | 383.94 | 0.236 |  |  |  |
| 183.96 | 0.154 | 233.96 | 0.134 | 283.96 | 0.051 |  | 333.96 | 0.206 | 383.96 | 0.233 |  |  |  |
| 183.98 | 0.158 | 233.98 | 0.127 | 283.98 | 0.074 |  | 333.98 | 0.233 | 383.98 | 0.195 |  |  |  |
| 184 | 0.182 | 234 | 0.098 | 284 | 0.079 |  | 334 | 0.242 | 384 | 0.227 |  |  |  |
| 184.02 | 0.157 | 234.02 | 0.106 | 284.02 | 0.082 |  | 334.02 | 0.245 | 384.02 | 0.128 |  |  |  |
| 184.04 | 0.12 | 234.04 | 0.106 | 284.04 | 0.078 |  | 334.04 | 0.267 | 384.04 | 0.225 |  |  |  |
| 184.06 | 0.118 | 234.06 | 0.134 | 284.06 | 0.074 |  | 334.06 | 0.275 | 384.06 | 0.23 |  |  |  |
| 184.08 | 0.129 | 234.08 | 0.141 | 284.08 | 0.077 |  | 334.08 | 0.283 | 384.08 | 0.228 |  |  |  |
| 184.1 | 0.151 | 234.1 | 0.141 | 284.1 | 0.086 |  | 334.1 | 0.217 | 384.1 | 0.233 |  |  |  |
| 184.12 | 0.16 | 234.12 | 0.126 | 284.12 | 0.096 |  | 334.12 | 0.197 | 384.12 | 0.24 |  |  |  |
| 184.14 | 0.219 | 234.14 | 0.099 | 284.14 | 0.095 |  | 334.14 | 0.195 | 384.14 | 0.242 |  |  |  |
| 184.16 | 0.293 | 234.16 | 0.088 | 284.16 | 0.105 |  | 334.16 | 0.188 | 384.16 | 0.243 |  |  |  |
| 184.18 | 0.288 | 234.18 | 0.105 | 284.18 | 0.109 |  | 334.18 | 0.193 | 384.18 | 0.233 |  |  |  |
| 184.2 | 0.191 | 234.2 | 0.099 | 284.2 | 0.111 |  | 334.2 | 0.194 | 384.2 | 0.222 |  |  |  |
| 184.22 | 0.133 | 234.22 | 0.036 | 284.22 | 0.11 |  | 334.22 | 0.19 | 384.22 | 0.242 |  |  |  |
| 184.24 | 0.142 | 234.24 | 0.06 | 284.24 | 0.102 |  | 334.24 | 0.173 | 384.24 | 0.238 |  |  |  |
| 184.26 | 0.152 | 234.26 | 0.073 | 284.26 | 0.095 |  | 334.26 | 0.192 | 384.26 | 0.236 |  |  |  |
| 184.28 | 0.164 | 234.28 | 0.073 | 284.28 | 0.094 |  | 334.28 | 0.217 | 384.28 | 0.235 |  |  |  |
| 184.3 | 0.143 | 234.3 | 0.079 | 284.3 | 0.095 |  | 334.3 | 0.225 | 384.3 | 0.234 |  |  |  |
| 184.32 | 0.123 | 234.32 | 0.082 | 284.32 | 0.092 |  | 334.32 | 0.216 | 384.32 | 0.232 |  |  |  |
| 184.34 | 0.086 | 234.34 | 0.082 | 284.34 | 0.09 |  | 334.34 | 0.232 | 384.34 | 0.221 |  |  |  |
| 184.36 | 0.074 | 234.36 | 0.085 | 284.36 | 0.08 |  | 334.36 | 0.202 | 384.36 | 0.211 |  |  |  |
| 184.38 | 0.074 | 234.38 | 0.064 | 284.38 | 0.073 |  | 334.38 | 0.224 | 384.38 | 0.217 |  |  |  |
| 184.4 | 0.088 | 234.4 | 0.058 | 284.4 | 0.068 |  | 334.4 | 0.263 | 384.4 | 0.217 |  |  |  |
| 184.42 | 0.089 | 234.42 | -0.008 | 284.42 | 0.063 |  | 334.42 | 0.251 | 384.42 | 0.211 |  |  |  |
| 184.44 | 0.08 | 234.44 | -0.005 | 284.44 | 0.059 |  | 334.44 | 0.262 | 384.44 | 0.21 |  |  |  |
| 184.46 | 0.082 | 234.46 | -0.001 | 284.46 | 0.058 |  | 334.46 | 0.257 | 384.46 | 0.202 |  |  |  |
| 184.48 | 0.106 | 234.48 | 0 | 284.48 | 0.054 |  | 334.48 | 0.245 | 384.48 | 0.205 |  |  |  |
| 184.5 | 0.117 | 234.5 | -0.003 | 284.5 | 0.153 |  | 334.5 | 0.264 | 384.5 | 0.195 |  |  |  |
| 184.52 | 0.125 | 234.52 | -0.014 | 284.52 | 0.063 |  | 334.52 | 0.261 | 384.52 | 0.167 |  |  |  |
| 184.54 | 0.087 | 234.54 | -0.016 | 284.54 | 0.05 |  | 334.54 | 0.262 | 384.54 | 0.16 |  |  |  |
| 184.56 | 0.096 | 234.56 | -0.03 | 284.56 | 0.05 |  | 334.56 | 0.26 | 384.56 | 0.163 |  |  |  |
| 184.58 | 0.108 | 234.58 | -0.025 | 284.58 | 0.055 |  | 334.58 | 0.253 | 384.58 | 0.165 |  |  |  |
| 184.6 | 0.132 | 234.6 | -0.02 | 284.6 | 0.059 |  | 334.6 | 0.243 | 384.6 | 0.176 |  |  |  |
| 184.62 | 0.131 | 234.62 | -0.004 | 284.62 | 0.07 |  | 334.62 | 0.234 | 384.62 | 0.181 |  |  |  |
| 184.64 | 0.162 | 234.64 | 0.009 | 284.64 | 0.084 |  | 334.64 | 0.225 | 384.64 | 0.18 |  |  |  |
| 184.66 | 0.194 | 234.66 | 0.025 | 284.66 | 0.1 |  | 334.66 | 0.203 | 384.66 | 0.168 |  |  |  |
| 184.68 | 0.185 | 234.68 | 0.036 | 284.68 | 0.116 |  | 334.68 | 0.204 | 384.68 | 0.158 |  |  |  |
| 184.7 | 0.187 | 234.7 | 0.054 | 284.7 | 0.145 |  | 334.7 | 0.215 | 384.7 | 0.158 |  |  |  |
| 184.72 | 0.201 | 234.72 | 0.063 | 284.72 | 0.18 |  | 334.72 | 0.231 | 384.72 | 0.182 |  |  |  |
| 184.74 | 0.216 | 234.74 | 0.082 | 284.74 | 0.158 |  | 334.74 | 0.186 | 384.74 | 0.207 |  |  |  |
| 184.76 | 0.169 | 234.76 | 0.18 | 284.76 | 0.097 |  | 334.76 | 0.248 | 384.76 | 0.213 |  |  |  |
| 184.78 | 0.143 | 234.78 | 0.079 | 284.78 | 0.071 |  | 334.78 | 0.25 | 384.78 | 0.212 |  |  |  |
| 184.8 | 0.142 | 234.8 | 0.086 | 284.8 | 0 |  | 334.8 | 0.257 | 384.8 | 0.199 |  |  |  |
| 184.82 | 0.143 | 234.82 | 0.085 | 284.82 | 0 |  | 334.82 | 0.257 | 384.82 | 0.189 |  |  |  |
| 184.84 | 0.146 | 234.84 | 0.095 | 284.84 | 0.006 |  | 334.84 | 0.259 | 384.84 | 0.194 |  |  |  |
| 184.86 | 0.159 | 234.86 | 0.149 | 284.86 | 0.009 |  | 334.86 | 0.262 | 384.86 | 0.204 |  |  |  |
| 184.88 | 0.151 | 234.88 | 0.152 | 284.88 | 0.006 |  | 334.88 | 0.26 | 384.88 | 0.212 |  |  |  |
| 184.9 | 0.143 | 234.9 | 0.131 | 284.9 | 0.005 |  | 334.9 | 0.26 | 384.9 | 0.208 |  |  |  |
| 184.92 | 0.133 | 234.92 | 0.108 | 284.92 | 0.005 |  | 334.92 | 0.271 | 384.92 | 0.202 |  |  |  |
| 184.94 | 0.136 | 234.94 | 0.098 | 284.94 | 0.005 |  | 334.94 | 0.265 | 384.94 | 0.203 |  |  |  |
| 184.96 | 0.137 | 234.96 | 0.095 | 284.96 | 0.008 |  | 334.96 | 0.242 | 384.96 | 0.21 |  |  |  |
| 184.98 | 0.126 | 234.98 | 0.095 | 284.98 | 0.01 |  | 334.98 | 0.222 | 384.98 | 0.212 |  |  |  |
| 185 | 0.117 | 235 | 0.093 | 285 | 0.016 |  | 335 | 0.228 | 385 | 0.201 |  |  |  |
| 185.02 | 0.005 | 235.02 | 0.095 | 285.02 | 0.024 |  | 335.02 | 0.229 | 385.02 | 0.153 |  |  |  |
| 185.04 | 0.01 | 235.04 | 0.097 | 285.04 | 0.033 |  | 335.04 | 0.228 | 385.04 | 0.183 |  |  |  |
| 185.06 | 0.019 | 235.06 | 0.084 | 285.06 | 0.04 |  | 335.06 | 0.236 | 385.06 | 0.207 |  |  |  |
| 185.08 | 0.034 | 235.08 | 0.077 | 285.08 | 0.043 |  | 335.08 | 0.257 | 385.08 | 0.2 |  |  |  |
| 185.1 | 0.037 | 235.1 | 0.073 | 285.1 | 0.045 |  | 335.1 | 0.257 | 385.1 | 0.205 |  |  |  |
| 185.12 | 0.03 | 235.12 | 0.072 | 285.12 | 0.053 |  | 335.12 | 0.255 | 385.12 | 0.19 |  |  |  |
| 185.14 | 0.04 | 235.14 | 0.072 | 285.14 | 0.07 |  | 335.14 | 0.252 | 385.14 | 0.171 |  |  |  |
| 185.16 | 0.058 | 235.16 | 0.057 | 285.16 | 0.077 |  | 335.16 | 0.258 | 385.16 | 0.223 |  |  |  |
| 185.18 | 0.039 | 235.18 | 0.006 | 285.18 | 0.077 |  | 335.18 | 0.261 | 385.18 | 0.223 |  |  |  |
| 185.2 | 0.035 | 235.2 | 0.007 | 285.2 | 0.097 |  | 335.2 | 0.217 | 385.2 | 0.221 |  |  |  |
| 185.22 | 0.048 | 235.22 | 0.008 | 285.22 | 0.109 |  | 335.22 | 0.232 | 385.22 | 0.114 |  |  |  |
| 185.24 | 0.053 | 235.24 | 0.007 | 285.24 | 0.123 |  | 335.24 | 0.227 | 385.24 | 0.203 |  |  |  |
| 185.26 | 0.075 | 235.26 | 0.001 | 285.26 | 0.146 |  | 335.26 | 0.24 | 385.26 | 0.186 |  |  |  |
| 185.28 | 0.094 | 235.28 | 0.006 | 285.28 | 0.118 |  | 335.28 | 0.269 | 385.28 | 0.171 |  |  |  |
| 185.3 | 0.125 | 235.3 | 0.014 | 285.3 | 0.126 |  | 335.3 | 0.274 | 385.3 | 0.206 |  |  |  |
| 185.32 | 0.133 | 235.32 | 0.024 | 285.32 | 0.094 |  | 335.32 | 0.256 | 385.32 | 0.205 |  |  |  |
| 185.34 | 0.16 | 235.34 | 0.03 | 285.34 | 0.192 |  | 335.34 | 0.248 | 385.34 | 0.214 |  |  |  |
| 185.36 | 0.26 | 235.36 | 0.033 | 285.36 | 0.292 |  | 335.36 | 0.255 | 385.36 | 0.207 |  |  |  |
| 185.38 | 0.257 | 235.38 | 0.028 | 285.38 | 0.363 |  | 335.38 | 0.251 | 385.38 | 0.21 |  |  |  |
| 185.4 | 0.167 | 235.4 | 0.04 | 285.4 | 0.362 |  | 335.4 | 0.26 | 385.4 | 0.21 |  |  |  |
| 185.42 | 0.158 | 235.42 | 0.042 | 285.42 | 0.298 |  | 335.42 | 0.265 | 385.42 | 0.187 |  |  |  |
| 185.44 | 0.159 | 235.44 | 0.041 | 285.44 | 0.194 |  | 335.44 | 0.252 | 385.44 | 0.187 |  |  |  |
| 185.46 | 0.171 | 235.46 | 0.047 | 285.46 | 0.138 |  | 335.46 | 0.263 | 385.46 | 0.217 |  |  |  |
| 185.48 | 0.173 | 235.48 | 0.052 | 285.48 | 0.123 |  | 335.48 | 0.296 | 385.48 | 0.214 |  |  |  |
| 185.5 | 0.169 | 235.5 | 0.127 | 285.5 | 0.13 |  | 335.5 | 0.269 | 385.5 | 0.204 |  |  |  |
| 185.52 | 0.145 | 235.52 | 0.153 | 285.52 | 0.121 |  | 335.52 | 0.284 | 385.52 | 0.208 |  |  |  |
| 185.54 | 0.138 | 235.54 | 0.171 | 285.54 | 0.113 |  | 335.54 | 0.341 | 385.54 | 0.221 |  |  |  |
| 185.56 | 0.135 | 235.56 | 0.181 | 285.56 | 0.158 |  | 335.56 | 0.286 | 385.56 | 0.242 |  |  |  |
| 185.58 | 0.152 | 235.58 | 0.157 | 285.58 | 0.181 |  | 335.58 | 0.255 | 385.58 | 0.231 |  |  |  |
| 185.6 | 0.146 | 235.6 | 0.165 | 285.6 | 0.16 |  | 335.6 | 0.252 | 385.6 | 0.223 |  |  |  |
| 185.62 | 0.151 | 235.62 | 0.163 | 285.62 | 0.144 |  | 335.62 | 0.241 | 385.62 | 0.22 |  |  |  |
| 185.64 | 0.149 | 235.64 | 0.161 | 285.64 | 0.125 |  | 335.64 | 0.201 | 385.64 | 0.218 |  |  |  |
| 185.66 | 0.138 | 235.66 | 0.153 | 285.66 | 0.108 |  | 335.66 | 0.19 | 385.66 | 0.217 |  |  |  |
| 185.68 | 0.128 | 235.68 | 0.117 | 285.68 | 0.091 |  | 335.68 | 0.215 | 385.68 | 0.199 |  |  |  |
| 185.7 | 0.118 | 235.7 | 0.093 | 285.7 | 0.109 |  | 335.7 | 0.237 | 385.7 | 0.187 |  |  |  |
| 185.72 | 0.114 | 235.72 | 0.115 | 285.72 | 0.124 |  | 335.72 | 0.222 | 385.72 | 0.196 |  |  |  |
| 185.74 | 0.119 | 235.74 | 0.089 | 285.74 | 0.101 |  | 335.74 | 0.221 | 385.74 | 0.219 |  |  |  |
| 185.76 | 0.089 | 235.76 | 0.079 | 285.76 | 0.097 |  | 335.76 | 0.218 | 385.76 | 0.192 |  |  |  |
| 185.78 | 0.096 | 235.78 | 0.067 | 285.78 | 0.095 |  | 335.78 | 0.218 | 385.78 | 0.175 |  |  |  |
| 185.8 | 0.103 | 235.8 | 0.059 | 285.8 | 0.092 |  | 335.8 | 0.233 | 385.8 | 0.156 |  |  |  |
| 185.82 | 0.181 | 235.82 | 0.051 | 285.82 | 0.089 |  | 335.82 | 0.211 | 385.82 | 0.213 |  |  |  |
| 185.84 | 0.232 | 235.84 | 0.061 | 285.84 | 0.086 |  | 335.84 | 0.205 | 385.84 | 0.22 |  |  |  |
| 185.86 | 0.241 | 235.86 | 0.102 | 285.86 | 0.083 |  | 335.86 | 0.21 | 385.86 | 0.226 |  |  |  |
| 185.88 | 0.198 | 235.88 | 0.122 | 285.88 | 0.087 |  | 335.88 | 0.227 | 385.88 | 0.23 |  |  |  |
| 185.9 | 0.149 | 235.9 | 0.14 | 285.9 | 0.087 |  | 335.9 | 0.24 | 385.9 | 0.228 |  |  |  |
| 185.92 | 0.124 | 235.92 | 0.161 | 285.92 | 0.089 |  | 335.92 | 0.253 | 385.92 | 0.218 |  |  |  |
| 185.94 | 0.119 | 235.94 | 0.168 | 285.94 | 0.082 |  | 335.94 | 0.219 | 385.94 | 0.141 |  |  |  |
| 185.96 | 0.115 | 235.96 | 0.168 | 285.96 | 0.081 |  | 335.96 | 0.247 | 385.96 | 0.157 |  |  |  |
| 185.98 | 0.116 | 235.98 | 0.012 | 285.98 | 0.09 |  | 335.98 | 0.267 | 385.98 | 0.191 |  |  |  |
| 186 | 0.108 | 236 | -0.023 | 286 | 0.012 |  | 336 | 0.277 | 386 | 0.151 |  |  |  |
| 186.02 | 0.107 | 236.02 | -0.037 | 286.02 | 0.017 |  | 336.02 | 0.288 | 386.02 | 0.165 |  |  |  |
| 186.04 | 0.106 | 236.04 | -0.041 | 286.04 | 0.019 |  | 336.04 | 0.225 | 386.04 | 0.181 |  |  |  |
| 186.06 | 0.089 | 236.06 | -0.038 | 286.06 | 0.022 |  | 336.06 | 0.206 | 386.06 | 0.184 |  |  |  |
| 186.08 | 0.1 | 236.08 | -0.03 | 286.08 | 0.205 |  | 336.08 | 0.165 | 386.08 | 0.19 |  |  |  |
| 186.1 | 0.119 | 236.1 | -0.02 | 286.1 | 0.119 |  | 336.1 | 0.119 | 386.1 | 0.187 |  |  |  |
| 186.12 | 0.135 | 236.12 | -0.012 | 286.12 | 0.102 |  | 336.12 | 0.233 | 386.12 | 0.177 |  |  |  |
| 186.14 | 0.171 | 236.14 | -0.001 | 286.14 | 0.093 |  | 336.14 | 0.224 | 386.14 | 0.164 |  |  |  |
| 186.16 | 0.211 | 236.16 | 0.009 | 286.16 | 0.1 |  | 336.16 | 0.232 | 386.16 | 0.149 |  |  |  |
| 186.18 | 0.224 | 236.18 | 0.026 | 286.18 | 0.13 |  | 336.18 | 0.251 | 386.18 | 0.153 |  |  |  |
| 186.2 | 0.188 | 236.2 | 0.045 | 286.2 | 0.147 |  | 336.2 | 0.283 | 386.2 | 0.15 |  |  |  |
| 186.22 | 0.007 | 236.22 | 0.056 | 286.22 | 0.14 |  | 336.22 | 0.277 | 386.22 | 0.176 |  |  |  |
| 186.24 | -0.029 | 236.24 | 0.06 | 286.24 | 0.127 |  | 336.24 | 0.264 | 386.24 | 0.161 |  |  |  |
| 186.26 | -0.015 | 236.26 | 0.075 | 286.26 | 0.128 |  | 336.26 | 0.251 | 386.26 | 0.199 |  |  |  |
| 186.28 | 0.012 | 236.28 | 0.078 | 286.28 | 0.133 |  | 336.28 | 0.247 | 386.28 | 0.195 |  |  |  |
| 186.3 | 0.019 | 236.3 | 0.089 | 286.3 | 0.132 |  | 336.3 | 0.245 | 386.3 | 0.194 |  |  |  |
| 186.32 | 0.013 | 236.32 | 0.094 | 286.32 | 0.108 |  | 336.32 | 0.278 | 386.32 | 0.197 |  |  |  |
| 186.34 | 0.04 | 236.34 | 0.099 | 286.34 | 0.107 |  | 336.34 | 0.279 | 386.34 | 0.203 |  |  |  |
| 186.36 | 0.046 | 236.36 | 0.102 | 286.36 | 0.116 |  | 336.36 | 0.273 | 386.36 | 0.202 |  |  |  |
| 186.38 | 0.051 | 236.38 | 0.116 | 286.38 | 0.118 |  | 336.38 | 0.27 | 386.38 | 0.195 |  |  |  |
| 186.4 | 0.058 | 236.4 | 0.111 | 286.4 | 0 |  | 336.4 | 0.274 | 386.4 | 0.188 |  |  |  |
| 186.42 | 0.077 | 236.42 | 0.107 | 286.42 | 0.007 |  | 336.42 | 0.273 | 386.42 | 0.178 |  |  |  |
| 186.44 | 0.076 | 236.44 | 0.105 | 286.44 | 0.003 |  | 336.44 | 0.275 | 386.44 | 0.169 |  |  |  |
| 186.46 | 0.056 | 236.46 | 0.099 | 286.46 | 0.011 |  | 336.46 | 0.271 | 386.46 | 0.162 |  |  |  |
| 186.48 | 0.083 | 236.48 | 0.095 | 286.48 | 0.02 |  | 336.48 | 0.262 | 386.48 | 0.139 |  |  |  |
| 186.5 | 0.093 | 236.5 | 0.093 | 286.5 | 0.027 |  | 336.5 | 0.246 | 386.5 | 0.203 |  |  |  |
| 186.52 | 0.102 | 236.52 | 0.096 | 286.52 | 0.037 |  | 336.52 | 0.211 | 386.52 | 0.216 |  |  |  |
| 186.54 | 0.097 | 236.54 | 0.123 | 286.54 | 0.051 |  | 336.54 | 0.201 | 386.54 | 0.215 |  |  |  |
| 186.56 | 0.09 | 236.56 | 0.156 | 286.56 | 0.052 |  | 336.56 | 0.231 | 386.56 | 0.212 |  |  |  |
| 186.58 | 0.09 | 236.58 | 0.141 | 286.58 | 0.049 |  | 336.58 | 0.234 | 386.58 | 0.207 |  |  |  |
| 186.6 | 0.128 | 236.6 | 0.099 | 286.6 | 0.048 |  | 336.6 | 0.234 | 386.6 | 0.194 |  |  |  |
| 186.62 | 0.13 | 236.62 | 0.076 | 286.62 | 0.049 |  | 336.62 | 0.216 | 386.62 | 0.213 |  |  |  |
| 186.64 | 0.135 | 236.64 | 0.104 | 286.64 | 0.058 |  | 336.64 | 0.215 | 386.64 | 0.217 |  |  |  |
| 186.66 | 0.108 | 236.66 | 0.13 | 286.66 | 0.064 |  | 336.66 | 0.223 | 386.66 | 0.193 |  |  |  |
| 186.68 | 0.093 | 236.68 | 0.141 | 286.68 | 0.076 |  | 336.68 | 0.218 | 386.68 | 0.171 |  |  |  |
| 186.7 | 0.085 | 236.7 | 0.145 | 286.7 | 0.081 |  | 336.7 | 0.216 | 386.7 | 0.116 |  |  |  |
| 186.72 | 0.08 | 236.72 | 0.145 | 286.72 | 0.087 |  | 336.72 | 0.234 | 386.72 | 0.092 |  |  |  |
| 186.74 | 0.077 | 236.74 | 0.141 | 286.74 | 0.097 |  | 336.74 | 0.232 | 386.74 | 0.213 |  |  |  |
| 186.76 | 0.084 | 236.76 | 0.139 | 286.76 | 0.105 |  | 336.76 | 0.223 | 386.76 | 0.195 |  |  |  |
| 186.78 | 0.086 | 236.78 | 0.131 | 286.78 | 0.11 |  | 336.78 | 0.219 | 386.78 | 0.214 |  |  |  |
| 186.8 | 0.091 | 236.8 | 0.134 | 286.8 | 0.23 |  | 336.8 | 0.139 | 386.8 | 0.218 |  |  |  |
| 186.82 | 0.083 | 236.82 | 0.142 | 286.82 | 0.163 |  | 336.82 | 0.144 | 386.82 | 0.217 |  |  |  |
| 186.84 | 0.06 | 236.84 | 0.149 | 286.84 | 0.131 |  | 336.84 | 0.211 | 386.84 | 0.218 |  |  |  |
| 186.86 | 0.058 | 236.86 | 0.148 | 286.86 | 0.122 |  | 336.86 | 0.217 | 386.86 | 0.213 |  |  |  |
| 186.88 | 0.066 | 236.88 | 0.134 | 286.88 | 0.127 |  | 336.88 | 0.2 | 386.88 | 0.216 |  |  |  |
| 186.9 | 0.063 | 236.9 | 0.087 | 286.9 | 0.127 |  | 336.9 | 0.172 | 386.9 | 0.224 |  |  |  |
| 186.92 | 0.069 | 236.92 | 0.057 | 286.92 | 0.118 |  | 336.92 | 0.15 | 386.92 | 0.227 |  |  |  |
| 186.94 | 0.076 | 236.94 | 0.069 | 286.94 | 0.116 |  | 336.94 | 0.174 | 386.94 | 0.232 |  |  |  |
| 186.96 | 0.091 | 236.96 | 0.069 | 286.96 | 0.123 |  | 336.96 | 0.252 | 386.96 | 0.219 |  |  |  |
| 186.98 | 0.081 | 236.98 | 0.045 | 286.98 | 0.146 |  | 336.98 | 0.224 | 386.98 | 0.19 |  |  |  |
| 187 | 0.064 | 237 | 0.045 | 287 | 0.179 |  | 337 | 0.225 | 387 | 0.156 |  |  |  |
| 187.02 | 0.053 | 237.02 | 0.047 | 287.02 | 0.2 |  | 337.02 | 0.226 | 387.02 | 0.17 |  |  |  |
| 187.04 | 0.159 | 237.04 | 0.051 | 287.04 | 0.209 |  | 337.04 | 0.239 | 387.04 | 0.211 |  |  |  |
| 187.06 | 0.159 | 237.06 | 0.055 | 287.06 | 0.204 |  | 337.06 | 0.239 | 387.06 | 0.24 |  |  |  |
| 187.08 | 0.164 | 237.08 | 0.05 | 287.08 | 0.201 |  | 337.08 | 0.238 | 387.08 | 0.238 |  |  |  |
| 187.1 | 0.162 | 237.1 | 0.048 | 287.1 | 0.193 |  | 337.1 | 0.226 | 387.1 | 0.223 |  |  |  |
| 187.12 | 0.155 | 237.12 | 0.046 | 287.12 | 0.186 |  | 337.12 | 0.22 | 387.12 | 0.201 |  |  |  |
| 187.14 | 0.141 | 237.14 | 0.053 | 287.14 | 0.201 |  | 337.14 | 0.217 | 387.14 | 0.198 |  |  |  |
| 187.16 | 0.127 | 237.16 | 0.057 | 287.16 | 0.198 |  | 337.16 | 0.225 | 387.16 | 0.205 |  |  |  |
| 187.18 | 0.122 | 237.18 | 0.057 | 287.18 | 0.146 |  | 337.18 | 0.224 | 387.18 | 0.234 |  |  |  |
| 187.2 | 0.125 | 237.2 | 0.039 | 287.2 | 0.144 |  | 337.2 | 0.247 | 387.2 | 0.238 |  |  |  |
| 187.22 | 0.121 | 237.22 | 0.086 | 287.22 | 0.129 |  | 337.22 | 0.253 | 387.22 | 0.234 |  |  |  |
| 187.24 | 0.114 | 237.24 | 0.033 | 287.24 | 0.122 |  | 337.24 | 0.251 | 387.24 | 0.214 |  |  |  |
| 187.26 | 0.115 | 237.26 | 0.042 | 287.26 | 0.127 |  | 337.26 | 0.253 | 387.26 | 0.219 |  |  |  |
| 187.28 | 0.134 | 237.28 | 0.044 | 287.28 | 0.111 |  | 337.28 | 0.246 | 387.28 | 0.189 |  |  |  |
| 187.3 | 0.152 | 237.3 | 0.034 | 287.3 | 0.098 |  | 337.3 | 0.25 | 387.3 | 0.153 |  |  |  |
| 187.32 | 0.148 | 237.32 | 0.04 | 287.32 | 0.088 |  | 337.32 | 0.249 | 387.32 | 0.171 |  |  |  |
| 187.34 | 0.122 | 237.34 | 0.051 | 287.34 | 0.096 |  | 337.34 | 0.278 | 387.34 | 0.171 |  |  |  |
| 187.36 | 0.161 | 237.36 | 0.059 | 287.36 | 0.112 |  | 337.36 | 0.284 | 387.36 | 0.15 |  |  |  |
| 187.38 | 0.206 | 237.38 | 0.06 | 287.38 | 0.118 |  | 337.38 | 0.292 | 387.38 | 0.162 |  |  |  |
| 187.4 | 0.22 | 237.4 | 0.069 | 287.4 | 0.121 |  | 337.4 | 0.273 | 387.4 | 0.165 |  |  |  |
| 187.42 | 0.012 | 237.42 | 0.058 | 287.42 | 0.134 |  | 337.42 | 0.251 | 387.42 | 0.173 |  |  |  |
| 187.44 | -0.02 | 237.44 | 0.037 | 287.44 | 0.148 |  | 337.44 | 0.239 | 387.44 | 0.194 |  |  |  |
| 187.46 | -0.038 | 237.46 | 0.051 | 287.46 | 0.144 |  | 337.46 | 0.226 | 387.46 | 0.2 |  |  |  |
| 187.48 | -0.039 | 237.48 | 0.07 | 287.48 | 0.127 |  | 337.48 | 0.228 | 387.48 | 0.198 |  |  |  |
| 187.5 | -0.024 | 237.5 | 0.193 | 287.5 | 0.11 |  | 337.5 | 0.23 | 387.5 | 0.208 |  |  |  |
| 187.52 | -0.004 | 237.52 | 0.118 | 287.52 | 0.096 |  | 337.52 | 0.221 | 387.52 | 0.179 |  |  |  |
| 187.54 | 0.028 | 237.54 | 0.1 | 287.54 | 0.094 |  | 337.54 | 0.225 | 387.54 | 0.163 |  |  |  |
| 187.56 | 0.075 | 237.56 | 0.111 | 287.56 | 0.089 |  | 337.56 | 0.22 | 387.56 | 0.153 |  |  |  |
| 187.58 | 0.094 | 237.58 | 0.136 | 287.58 | 0.091 |  | 337.58 | 0.218 | 387.58 | 0.152 |  |  |  |
| 187.6 | 0.03 | 237.6 | 0.145 | 287.6 | 0.003 |  | 337.6 | 0.219 | 387.6 | 0.16 |  |  |  |
| 187.62 | 0.021 | 237.62 | 0.141 | 287.62 | 0.09 |  | 337.62 | 0.23 | 387.62 | 0.171 |  |  |  |
| 187.64 | 0.041 | 237.64 | 0.116 | 287.64 | 0.108 |  | 337.64 | 0.251 | 387.64 | 0.174 |  |  |  |
| 187.66 | 0.059 | 237.66 | 0.097 | 287.66 | 0.119 |  | 337.66 | 0.245 | 387.66 | 0.172 |  |  |  |
| 187.68 | 0.08 | 237.68 | 0.088 | 287.68 | 0.11 |  | 337.68 | 0.251 | 387.68 | 0.179 |  |  |  |
| 187.7 | 0.106 | 237.7 | 0.084 | 287.7 | 0.1 |  | 337.7 | 0.244 | 387.7 | 0.195 |  |  |  |
| 187.72 | 0.12 | 237.72 | 0.09 | 287.72 | 0.096 |  | 337.72 | 0.256 | 387.72 | 0.198 |  |  |  |
| 187.74 | 0.117 | 237.74 | 0.094 | 287.74 | 0.093 |  | 337.74 | 0.248 | 387.74 | 0.198 |  |  |  |
| 187.76 | 0.127 | 237.76 | 0.084 | 287.76 | 0.092 |  | 337.76 | 0.232 | 387.76 | 0.199 |  |  |  |
| 187.78 | 0.166 | 237.78 | 0.1 | 287.78 | 0.092 |  | 337.78 | 0.222 | 387.78 | 0.198 |  |  |  |
| 187.8 | 0.133 | 237.8 | 0.121 | 287.8 | 0.093 |  | 337.8 | 0.218 | 387.8 | 0.192 |  |  |  |
| 187.82 | 0.135 | 237.82 | 0.115 | 287.82 | 0.091 |  | 337.82 | 0.219 | 387.82 | 0.179 |  |  |  |
| 187.84 | 0.137 | 237.84 | 0.079 | 287.84 | 0.088 |  | 337.84 | 0.219 | 387.84 | 0.159 |  |  |  |
| 187.86 | 0.127 | 237.86 | 0.099 | 287.86 | 0.098 |  | 337.86 | 0.22 | 387.86 | 0.148 |  |  |  |
| 187.88 | 0.104 | 237.88 | 0.108 | 287.88 | 0.12 |  | 337.88 | 0.221 | 387.88 | 0.158 |  |  |  |
| 187.9 | 0.182 | 237.9 | 0.116 | 287.9 | 0.143 |  | 337.9 | 0.226 | 387.9 | 0.139 |  |  |  |
| 187.92 | 0.175 | 237.92 | 0.123 | 287.92 | 0.159 |  | 337.92 | 0.221 | 387.92 | 0.122 |  |  |  |
| 187.94 | 0.02 | 237.94 | 0.119 | 287.94 | 0.151 |  | 337.94 | 0.219 | 387.94 | 0.123 |  |  |  |
| 187.96 | 0.013 | 237.96 | 0.114 | 287.96 | 0.142 |  | 337.96 | 0.236 | 387.96 | 0.132 |  |  |  |
| 187.98 | 0.031 | 237.98 | 0.106 | 287.98 | 0.133 |  | 337.98 | 0.262 | 387.98 | 0.14 |  |  |  |
| 188 | 0.008 | 238 | 0.1 | 288 | 0.009 |  | 338 | 0.252 | 388 | 0.145 |  |  |  |
| 188.02 | 0.036 | 238.02 | 0.088 | 288.02 | 0.005 |  | 338.02 | 0.234 | 388.02 | 0.146 |  |  |  |
| 188.04 | 0.023 | 238.04 | 0.072 | 288.04 | 0.004 |  | 338.04 | 0.231 | 388.04 | 0.149 |  |  |  |
| 188.06 | -0.015 | 238.06 | 0.062 | 288.06 | 0.003 |  | 338.06 | 0.241 | 388.06 | 0.148 |  |  |  |
| 188.08 | 0.029 | 238.08 | 0.055 | 288.08 | 0.009 |  | 338.08 | 0.237 | 388.08 | 0.141 |  |  |  |
| 188.1 | 0.088 | 238.1 | 0.076 | 288.1 | 0.022 |  | 338.1 | 0.223 | 388.1 | 0.077 |  |  |  |
| 188.12 | 0.119 | 238.12 | 0.08 | 288.12 | 0.031 |  | 338.12 | 0.214 | 388.12 | 0.116 |  |  |  |
| 188.14 | 0.092 | 238.14 | 0.072 | 288.14 | 0.049 |  | 338.14 | 0.212 | 388.14 | 0.118 |  |  |  |
| 188.16 | 0.062 | 238.16 | 0.027 | 288.16 | 0.061 |  | 338.16 | 0.206 | 388.16 | 0.117 |  |  |  |
| 188.18 | 0.041 | 238.18 | 0.076 | 288.18 | 0.059 |  | 338.18 | 0.207 | 388.18 | 0.112 |  |  |  |
| 188.2 | 0.048 | 238.2 | 0.086 | 288.2 | 0.063 |  | 338.2 | 0.222 | 388.2 | 0.113 |  |  |  |
| 188.22 | 0.022 | 238.22 | 0.103 | 288.22 | 0.07 |  | 338.22 | 0.247 | 388.22 | 0.121 |  |  |  |
| 188.24 | 0.02 | 238.24 | 0.112 | 288.24 | 0.079 |  | 338.24 | 0.265 | 388.24 | 0.132 |  |  |  |
| 188.26 | 0.02 | 238.26 | 0.115 | 288.26 | 0.083 |  | 338.26 | 0.25 | 388.26 | 0.146 |  |  |  |
| 188.28 | 0.028 | 238.28 | 0.111 | 288.28 | 0.088 |  | 338.28 | -0.013 | 388.28 | 0.162 |  |  |  |
| 188.3 | 0.045 | 238.3 | 0.109 | 288.3 | 0.096 |  | 338.3 | -0.041 | 388.3 | 0.17 |  |  |  |
| 188.32 | 0.057 | 238.32 | 0.116 | 288.32 | 0.113 |  | 338.32 | -0.023 | 388.32 | 0.181 |  |  |  |
| 188.34 | 0.067 | 238.34 | 0.125 | 288.34 | 0.132 |  | 338.34 | -0.017 | 388.34 | 0.183 |  |  |  |
| 188.36 | 0.076 | 238.36 | 0.124 | 288.36 | 0.139 |  | 338.36 | 0 | 388.36 | 0.189 |  |  |  |
| 188.38 | 0.083 | 238.38 | 0.101 | 288.38 | 0.123 |  | 338.38 | 0.019 | 388.38 | 0.162 |  |  |  |
| 188.4 | 0.01 | 238.4 | 0.075 | 288.4 | -0.0496 |  | 338.4 | 0.013 | 388.4 | 0.168 |  |  |  |
| 188.42 | 0.016 | 238.42 | 0.086 | 288.42 | -0.039 |  | 338.42 | -0.017 | 388.42 | 0.19 |  |  |  |
| 188.44 | 0.024 | 238.44 | 0.068 | 288.44 | -0.0291 |  | 338.44 | 0.041 | 388.44 | 0.206 |  |  |  |
| 188.46 | 0.013 | 238.46 | 0.065 | 288.46 | -0.0185 |  | 338.46 | 0.055 | 388.46 | 0.212 |  |  |  |
| 188.48 | 0.016 | 238.48 | 0.034 | 288.48 | 0.064 |  | 338.48 | 0.073 | 388.48 | 0.202 |  |  |  |
| 188.5 | 0.021 | 238.5 | 0.06 | 288.5 | 0.064 |  | 338.5 | 0.097 | 388.5 | 0.183 |  |  |  |
| 188.52 | 0.032 | 238.52 | 0.086 | 288.52 | 0.187 |  | 338.52 | 0.124 | 388.52 | 0.182 |  |  |  |
| 188.54 | 0.086 | 238.54 | 0.097 | 288.54 | 0.208 |  | 338.54 | 0.139 | 388.54 | 0.195 |  |  |  |
| 188.56 | 0.087 | 238.56 | 0.111 | 288.56 | 0.207 |  | 338.56 | 0.159 | 388.56 | 0.192 |  |  |  |
| 188.58 | 0.061 | 238.58 | 0.111 | 288.58 | 0.18 |  | 338.58 | 0.174 | 388.58 | 0.193 |  |  |  |
| 188.6 | 0.056 | 238.6 | 0.145 | 288.6 | 0.149 |  | 338.6 | 0.188 | 388.6 | 0.181 |  |  |  |
| 188.62 | 0.057 | 238.62 | 0.062 | 288.62 | 0.145 |  | 338.62 | 0.206 | 388.62 | 0.176 |  |  |  |
| 188.64 | 0.053 | 238.64 | 0.085 | 288.64 | 0.156 |  | 338.64 | 0.221 | 388.64 | 0.176 |  |  |  |
| 188.66 | 0.043 | 238.66 | 0.096 | 288.66 | 0.154 |  | 338.66 | 0.239 | 388.66 | 0.177 |  |  |  |
| 188.68 | 0.05 | 238.68 | 0.098 | 288.68 | 0.155 |  | 338.68 | 0.267 | 388.68 | 0.165 |  |  |  |
| 188.7 | 0.055 | 238.7 | 0.073 | 288.7 | 0.156 |  | 338.7 | 0.23 | 388.7 | 0.138 |  |  |  |
| 188.72 | 0.056 | 238.72 | 0.044 | 288.72 | 0.16 |  | 338.72 | 0.247 | 388.72 | 0.202 |  |  |  |
| 188.74 | 0.086 | 238.74 | 0.036 | 288.74 | 0.17 |  | 338.74 | 0.251 | 388.74 | 0.212 |  |  |  |
| 188.76 | 0.096 | 238.76 | 0.041 | 288.76 | 0.169 |  | 338.76 | 0.25 | 388.76 | 0.209 |  |  |  |
| 188.78 | 0.104 | 238.78 | 0.04 | 288.78 | 0.16 |  | 338.78 | 0.256 | 388.78 | 0.205 |  |  |  |
| 188.8 | 0.121 | 238.8 | 0.05 | 288.8 | 0.148 |  | 338.8 | 0.262 | 388.8 | 0.203 |  |  |  |
| 188.82 | 0.075 | 238.82 | 0.045 | 288.82 | 0.146 |  | 338.82 | 0.263 | 388.82 | 0.203 |  |  |  |
| 188.84 | 0.073 | 238.84 | 0.031 | 288.84 | 0.144 |  | 338.84 | 0.257 | 388.84 | 0.225 |  |  |  |
| 188.86 | 0.116 | 238.86 | 0.019 | 288.86 | 0.154 |  | 338.86 | 0.243 | 388.86 | 0.23 |  |  |  |
| 188.88 | 0.117 | 238.88 | 0.019 | 288.88 | 0.153 |  | 338.88 | 0.239 | 388.88 | 0.263 |  |  |  |
| 188.9 | 0.112 | 238.9 | 0.026 | 288.9 | 0.129 |  | 338.9 | 0.245 | 388.9 | 0.26 |  |  |  |
| 188.92 | 0.099 | 238.92 | 0.024 | 288.92 | 0.153 |  | 338.92 | 0.253 | 388.92 | 0.244 |  |  |  |
| 188.94 | 0.088 | 238.94 | 0.036 | 288.94 | 0.163 |  | 338.94 | 0.254 | 388.94 | 0.234 |  |  |  |
| 188.96 | 0.082 | 238.96 | 0.039 | 288.96 | 0.15 |  | 338.96 | 0.252 | 388.96 | 0.244 |  |  |  |
| 188.98 | 0.078 | 238.98 | 0.041 | 288.98 | 0.131 |  | 338.98 | 0.248 | 388.98 | 0.191 |  |  |  |
| 189 | 0.073 | 239 | 0.046 | 289 | 0.113 |  | 339 | 0.247 | 389 | 0.222 |  |  |  |
| 189.02 | 0.071 | 239.02 | 0.05 | 289.02 | 0.111 |  | 339.02 | 0.24 | 389.02 | 0.235 |  |  |  |
| 189.04 | 0.072 | 239.04 | 0.055 | 289.04 | 0.12 |  | 339.04 | 0.212 | 389.04 | 0.242 |  |  |  |
| 189.06 | 0.079 | 239.06 | 0.061 | 289.06 | 0.129 |  | 339.06 | 0.209 | 389.06 | 0.244 |  |  |  |
| 189.08 | 0.088 | 239.08 | 0.062 | 289.08 | 0.133 |  | 339.08 | 0.207 | 389.08 | 0.239 |  |  |  |
| 189.1 | 0.106 | 239.1 | 0.058 | 289.1 | 0.132 |  | 339.1 | 0.234 | 389.1 | 0.216 |  |  |  |
| 189.12 | 0.182 | 239.12 | 0.049 | 289.12 | 0.13 |  | 339.12 | 0.23 | 389.12 | 0.208 |  |  |  |
| 189.14 | 0.227 | 239.14 | 0.045 | 289.14 | 0.109 |  | 339.14 | 0.145 | 389.14 | 0.228 |  |  |  |
| 189.16 | 0.206 | 239.16 | 0.045 | 289.16 | 0.129 |  | 339.16 | 0.137 | 389.16 | 0.219 |  |  |  |
| 189.18 | 0.071 | 239.18 | 0.049 | 289.18 | 0.118 |  | 339.18 | 0.134 | 389.18 | 0.152 |  |  |  |
| 189.2 | 0.003 | 239.2 | 0.056 | 289.2 | 0.113 |  | 339.2 | 0.133 | 389.2 | 0.116 |  |  |  |
| 189.22 | 0 | 239.22 | 0.075 | 289.22 | 0.119 |  | 339.22 | 0.204 | 389.22 | 0.112 |  |  |  |
| 189.24 | -0.001 | 239.24 | 0.054 | 289.24 | 0.121 |  | 339.24 | 0.218 | 389.24 | 0.165 |  |  |  |
| 189.26 | 0 | 239.26 | 0.056 | 289.26 | 0.12 |  | 339.26 | 0.218 | 389.26 | 0.219 |  |  |  |
| 189.28 | 0.006 | 239.28 | 0.084 | 289.28 | 0.124 |  | 339.28 | 0.217 | 389.28 | 0.203 |  |  |  |
| 189.3 | 0.017 | 239.3 | 0.107 | 289.3 | 0.129 |  | 339.3 | 0.205 | 389.3 | 0.18 |  |  |  |
| 189.32 | 0.031 | 239.32 | 0.12 | 289.32 | 0.138 |  | 339.32 | 0.205 | 389.32 | 0.174 |  |  |  |
| 189.34 | 0.042 | 239.34 | 0.121 | 289.34 | 0.147 |  | 339.34 | 0.21 | 389.34 | 0.191 |  |  |  |
| 189.36 | 0.057 | 239.36 | 0.119 | 289.36 | 0.14 |  | 339.36 | 0.223 | 389.36 | 0.19 |  |  |  |
| 189.38 | 0.139 | 239.38 | 0.12 | 289.38 | 0.156 |  | 339.38 | 0.222 | 389.38 | 0.122 |  |  |  |
| 189.4 | 0.205 | 239.4 | 0.123 | 289.4 | 0.154 |  | 339.4 | 0.225 | 389.4 | 0.066 |  |  |  |
| 189.42 | 0.239 | 239.42 | 0.124 | 289.42 | 0.137 |  | 339.42 | 0.224 | 389.42 | 0.164 |  |  |  |
| 189.44 | 0.223 | 239.44 | 0.124 | 289.44 | 0.115 |  | 339.44 | 0.22 | 389.44 | 0.147 |  |  |  |
| 189.46 | 0.148 | 239.46 | 0.121 | 289.46 | 0.111 |  | 339.46 | 0.219 | 389.46 | 0.116 |  |  |  |
| 189.48 | 0.095 | 239.48 | 0.007 | 289.48 | 0.101 |  | 339.48 | 0.214 | 389.48 | 0.105 |  |  |  |
| 189.5 | 0.057 | 239.5 | 0.011 | 289.5 | 0.088 |  | 339.5 | 0.22 | 389.5 | 0.108 |  |  |  |
| 189.52 | 0.067 | 239.52 | 0.021 | 289.52 | 0.075 |  | 339.52 | 0.22 | 389.52 | 0.121 |  |  |  |
| 189.54 | 0.071 | 239.54 | 0.019 | 289.54 | 0.071 |  | 339.54 | 0.21 | 389.54 | 0.11 |  |  |  |
| 189.56 | 0.078 | 239.56 | 0.023 | 289.56 | 0.072 |  | 339.56 | 0.213 | 389.56 | 0.109 |  |  |  |
| 189.58 | 0.09 | 239.58 | 0.029 | 289.58 | 0.069 |  | 339.58 | 0.223 | 389.58 | 0.104 |  |  |  |
| 189.6 | 0.009 | 239.6 | 0.037 | 289.6 | 0.059 |  | 339.6 | 0.211 | 389.6 | 0.104 |  |  |  |
| 189.62 | 0.016 | 239.62 | 0.041 | 289.62 | 0.058 |  | 339.62 | 0.222 | 389.62 | 0.109 |  |  |  |
| 189.64 | 0.028 | 239.64 | 0.033 | 289.64 | 0.091 |  | 339.64 | 0.222 | 389.64 | 0.112 |  |  |  |
| 189.66 | 0.037 | 239.66 | 0.054 | 289.66 | 0.113 |  | 339.66 | 0.215 | 389.66 | 0.094 |  |  |  |
| 189.68 | 0.047 | 239.68 | 0.06 | 289.68 | 0.11 |  | 339.68 | 0.202 | 389.68 | 0.158 |  |  |  |
| 189.7 | 0.048 | 239.7 | 0.064 | 289.7 | 0.122 |  | 339.7 | 0.178 | 389.7 | 0.175 |  |  |  |
| 189.72 | 0.049 | 239.72 | 0.073 | 289.72 | 0.147 |  | 339.72 | 0.169 | 389.72 | 0.173 |  |  |  |
| 189.74 | 0.053 | 239.74 | 0.074 | 289.74 | 0.167 |  | 339.74 | 0.148 | 389.74 | 0.176 |  |  |  |
| 189.76 | 0.074 | 239.76 | 0.07 | 289.76 | 0.171 |  | 339.76 | 0.151 | 389.76 | 0.186 |  |  |  |
| 189.78 | 0.097 | 239.78 | 0.065 | 289.78 | 0.186 |  | 339.78 | 0.14 | 389.78 | 0.183 |  |  |  |
| 189.8 | 0.099 | 239.8 | 0.077 | 289.8 | 0.195 |  | 339.8 | 0.164 | 389.8 | 0.176 |  |  |  |
| 189.82 | 0.087 | 239.82 | 0.102 | 289.82 | 0.201 |  | 339.82 | 0.154 | 389.82 | 0.16 |  |  |  |
| 189.84 | 0.085 | 239.84 | 0.117 | 289.84 | 0.208 |  | 339.84 | 0.154 | 389.84 | 0.155 |  |  |  |
| 189.86 | 0.075 | 239.86 | 0.118 | 289.86 | 0.202 |  | 339.86 | 0.169 | 389.86 | 0.144 |  |  |  |
| 189.88 | 0.079 | 239.88 | 0.074 | 289.88 | 0.201 |  | 339.88 | 0.194 | 389.88 | 0.143 |  |  |  |
| 189.9 | 0.096 | 239.9 | 0.074 | 289.9 | 0.196 |  | 339.9 | 0.175 | 389.9 | 0.141 |  |  |  |
| 189.92 | 0.105 | 239.92 | 0.073 | 289.92 | 0.197 |  | 339.92 | 0.177 | 389.92 | 0.141 |  |  |  |
| 189.94 | 0.154 | 239.94 | 0.069 | 289.94 | 0.191 |  | 339.94 | 0.187 | 389.94 | 0.123 |  |  |  |
| 189.96 | 0.176 | 239.96 | 0.072 | 289.96 | 0.171 |  | 339.96 | 0.198 | 389.96 | 0.165 |  |  |  |
| 189.98 | 0.181 | 239.98 | 0.071 | 289.98 | 0.13 |  | 339.98 | 0.206 | 389.98 | 0.16 |  |  |  |
| 190 | 0.164 | 240 | 0.074 | 290 | 0.127 |  | 340 | 0.221 | 390 | 0.16 |  |  |  |
| 190.02 | 0.103 | 240.02 | 0.073 | 290.02 | 0.132 |  | 340.02 | 0.209 | 390.02 | 0.152 |  |  |  |
| 190.04 | 0.082 | 240.04 | 0.067 | 290.04 | 0.13 |  | 340.04 | 0.19 | 390.04 | 0.127 |  |  |  |
| 190.06 | 0.048 | 240.06 | 0.062 | 290.06 | 0.122 |  | 340.06 | 0.184 | 390.06 | 0.127 |  |  |  |
| 190.08 | 0.036 | 240.08 | 0.061 | 290.08 | 0.129 |  | 340.08 | 0.191 | 390.08 | 0.136 |  |  |  |
| 190.1 | 0.038 | 240.1 | 0.064 | 290.1 | 0.157 |  | 340.1 | 0.214 | 390.1 | 0.132 |  |  |  |
| 190.12 | 0.05 | 240.12 | 0.078 | 290.12 | 0.014 |  | 340.12 | 0.219 | 390.12 | 0.135 |  |  |  |
| 190.14 | 0.058 | 240.14 | 0.088 | 290.14 | 0.065 |  | 340.14 | 0.193 | 390.14 | 0.139 |  |  |  |
| 190.16 | 0.065 | 240.16 | 0.091 | 290.16 | 0.05 |  | 340.16 | 0.181 | 390.16 | 0.125 |  |  |  |
| 190.18 | 0.071 | 240.18 | 0.085 | 290.18 | 0.017 |  | 340.18 | 0.17 | 390.18 | 0.135 |  |  |  |
| 190.2 | 0.074 | 240.2 | 0.086 | 290.2 | 0.043 |  | 340.2 | 0.162 | 390.2 | 0.16 |  |  |  |
| 190.22 | 0.083 | 240.22 | 0.093 | 290.22 | 0.022 |  | 340.22 | 0.222 | 390.22 | 0.163 |  |  |  |
| 190.24 | 0.092 | 240.24 | 0.09 | 290.24 | 0.016 |  | 340.24 | 0.228 | 390.24 | 0.149 |  |  |  |
| 190.26 | 0.106 | 240.26 | 0.109 | 290.26 | 0.038 |  | 340.26 | 0.234 | 390.26 | 0.152 |  |  |  |
| 190.28 | 0.109 | 240.28 | 0.002 | 290.28 | 0.033 |  | 340.28 | 0.235 | 390.28 | 0.161 |  |  |  |
| 190.3 | 0.125 | 240.3 | 0.004 | 290.3 | 0.013 |  | 340.3 | 0.234 | 390.3 | 0.166 |  |  |  |
| 190.32 | 0.144 | 240.32 | 0.013 | 290.32 | 0.02 |  | 340.32 | 0.232 | 390.32 | 0.172 |  |  |  |
| 190.34 | 0.153 | 240.34 | 0.023 | 290.34 | 0.035 |  | 340.34 | 0.213 | 390.34 | 0.157 |  |  |  |
| 190.36 | 0.158 | 240.36 | 0.028 | 290.36 | 0.04 |  | 340.36 | 0.196 | 390.36 | 0.138 |  |  |  |
| 190.38 | 0.159 | 240.38 | 0.029 | 290.38 | 0.057 |  | 340.38 | 0.19 | 390.38 | 0.126 |  |  |  |
| 190.4 | 0.152 | 240.4 | 0.023 | 290.4 | 0.064 |  | 340.4 | 0.193 | 390.4 | 0.121 |  |  |  |
| 190.42 | 0.131 | 240.42 | 0.023 | 290.42 | 0.056 |  | 340.42 | 0.208 | 390.42 | 0.126 |  |  |  |
| 190.44 | 0.107 | 240.44 | 0.028 | 290.44 | 0.057 |  | 340.44 | 0.212 | 390.44 | 0.123 |  |  |  |
| 190.46 | 0.116 | 240.46 | 0.032 | 290.46 | 0.09 |  | 340.46 | 0.218 | 390.46 | 0.147 |  |  |  |
| 190.48 | 0.129 | 240.48 | -0.057 | 290.48 | 0.097 |  | 340.48 | 0.222 | 390.48 | 0.146 |  |  |  |
| 190.5 | 0.139 | 240.5 | -0.051 | 290.5 | 0.105 |  | 340.5 | 0.219 | 390.5 | 0.143 |  |  |  |
| 190.52 | 0.145 | 240.52 | -0.024 | 290.52 | 0.014 |  | 340.52 | 0.219 | 390.52 | 0.151 |  |  |  |
| 190.54 | 0.116 | 240.54 | -0.014 | 290.54 | 0.007 |  | 340.54 | 0.012 | 390.54 | 0.186 |  |  |  |
| 190.56 | 0.124 | 240.56 | -0.038 | 290.56 | 0.011 |  | 340.56 | 0.02 | 390.56 | 0.22 |  |  |  |
| 190.58 | 0.271 | 240.58 | -0.023 | 290.58 | 0.025 |  | 340.58 | 0.016 | 390.58 | 0.277 |  |  |  |
| 190.6 | 0.166 | 240.6 | -0.022 | 290.6 | 0.034 |  | 340.6 | 0.007 | 390.6 | 0.247 |  |  |  |
| 190.62 | 0.138 | 240.62 | -0.023 | 290.62 | 0.045 |  | 340.62 | 0.028 | 390.62 | 0.175 |  |  |  |
| 190.64 | 0.107 | 240.64 | -0.02 | 290.64 | 0.054 |  | 340.64 | 0.035 | 390.64 | 0.138 |  |  |  |
| 190.66 | 0.104 | 240.66 | -0.011 | 290.66 | 0.142 |  | 340.66 | 0.048 | 390.66 | 0.11 |  |  |  |
| 190.68 | 0.124 | 240.68 | 0 | 290.68 | 0.154 |  | 340.68 | 0.078 | 390.68 | 0.101 |  |  |  |
| 190.7 | 0.128 | 240.7 | 0.002 | 290.7 | 0.154 |  | 340.7 | 0.085 | 390.7 | 0.103 |  |  |  |
| 190.72 | 0.127 | 240.72 | 0.002 | 290.72 | 0.152 |  | 340.72 | 0.09 | 390.72 | 0.103 |  |  |  |
| 190.74 | 0.136 | 240.74 | 0.003 | 290.74 | 0.142 |  | 340.74 | 0.104 | 390.74 | 0.085 |  |  |  |
| 190.76 | 0.149 | 240.76 | 0.011 | 290.76 | 0.144 |  | 340.76 | 0.116 | 390.76 | 0.093 |  |  |  |
| 190.78 | 0.175 | 240.78 | 0.012 | 290.78 | 0.162 |  | 340.78 | 0.127 | 390.78 | 0.092 |  |  |  |
| 190.8 | 0.177 | 240.8 | 0.011 | 290.8 | 0.169 |  | 340.8 | 0.134 | 390.8 | 0.097 |  |  |  |
| 190.82 | 0.156 | 240.82 | 0.009 | 290.82 | 0.177 |  | 340.82 | 0.14 | 390.82 | 0.093 |  |  |  |
| 190.84 | 0.142 | 240.84 | 0.01 | 290.84 | 0.174 |  | 340.84 | 0.147 | 390.84 | 0.098 |  |  |  |
| 190.86 | 0.12 | 240.86 | 0.004 | 290.86 | 0.175 |  | 340.86 | 0.185 | 390.86 | 0.121 |  |  |  |
| 190.88 | 0.116 | 240.88 | 0.014 | 290.88 | 0.176 |  | 340.88 | 0.201 | 390.88 | 0.132 |  |  |  |
| 190.9 | 0.127 | 240.9 | 0.01 | 290.9 | 0.188 |  | 340.9 | 0.218 | 390.9 | 0.133 |  |  |  |
| 190.92 | 0.173 | 240.92 | 0.001 | 290.92 | 0.174 |  | 340.92 | 0.227 | 390.92 | 0.148 |  |  |  |
| 190.94 | 0.235 | 240.94 | -0.015 | 290.94 | 0.18 |  | 340.94 | 0.231 | 390.94 | 0.151 |  |  |  |
| 190.96 | 0.314 | 240.96 | -0.019 | 290.96 | 0.181 |  | 340.96 | 0.223 | 390.96 | 0.146 |  |  |  |
| 190.98 | 0.2406 | 240.98 | -0.02 | 290.98 | 0.175 |  | 340.98 | 0.191 | 390.98 | 0.148 |  |  |  |
| 191 | 0.2808 | 241 | -0.021 | 291 | 0.174 |  | 341 | 0.17 | 391 | 0.156 |  |  |  |
| 191.02 | 0.2718 | 241.02 | -0.022 | 291.02 | 0.194 |  | 341.02 | 0.217 | 391.02 | 0.175 |  |  |  |
| 191.04 | 0.317 | 241.04 | -0.02 | 291.04 | 0.207 |  | 341.04 | 0.217 | 391.04 | 0.182 |  |  |  |
| 191.06 | 0.188 | 241.06 | -0.018 | 291.06 | 0.218 |  | 341.06 | 0.22 | 391.06 | 0.181 |  |  |  |
| 191.08 | 0.125 | 241.08 | 0 | 291.08 | 0.193 |  | 341.08 | 0.218 | 391.08 | 0.173 |  |  |  |
| 191.1 | 0.104 | 241.1 | -0.002 | 291.1 | 0.136 |  | 341.1 | 0.22 | 391.1 | 0.169 |  |  |  |
| 191.12 | 0.098 | 241.12 | -0.005 | 291.12 | 0.13 |  | 341.12 | 0.212 | 391.12 | 0.143 |  |  |  |
| 191.14 | 0.106 | 241.14 | -0.008 | 291.14 | 0.173 |  | 341.14 | 0.214 | 391.14 | 0.164 |  |  |  |
| 191.16 | 0.116 | 241.16 | -0.01 | 291.16 | 0.2 |  | 341.16 | 0.194 | 391.16 | 0.172 |  |  |  |
| 191.18 | 0.112 | 241.18 | -0.01 | 291.18 | 0.206 |  | 341.18 | 0.176 | 391.18 | 0.182 |  |  |  |
| 191.2 | 0.102 | 241.2 | -0.01 | 291.2 | 0.194 |  | 341.2 | 0.224 | 391.2 | 0.165 |  |  |  |
| 191.22 | 0.132 | 241.22 | -0.011 | 291.22 | 0.176 |  | 341.22 | 0.229 | 391.22 | 0.171 |  |  |  |
| 191.24 | 0.105 | 241.24 | -0.011 | 291.24 | 0.153 |  | 341.24 | 0.226 | 391.24 | 0.181 |  |  |  |
| 191.26 | 0.109 | 241.26 | -0.013 | 291.26 | 0.164 |  | 341.26 | 0.225 | 391.26 | 0.22 |  |  |  |
| 191.28 | 0.111 | 241.28 | -0.013 | 291.28 | 0.193 |  | 341.28 | 0.231 | 391.28 | 0.232 |  |  |  |
| 191.3 | 0.112 | 241.3 | -0.01 | 291.3 | 0.193 |  | 341.3 | 0.219 | 391.3 | 0.209 |  |  |  |
| 191.32 | 0.124 | 241.32 | -0.008 | 291.32 | 0.156 |  | 341.32 | 0.228 | 391.32 | 0.199 |  |  |  |
| 191.34 | 0.137 | 241.34 | -0.009 | 291.34 | 0.162 |  | 341.34 | 0.226 | 391.34 | 0.198 |  |  |  |
| 191.36 | 0.139 | 241.36 | -0.011 | 291.36 | 0.118 |  | 341.36 | 0.204 | 391.36 | 0.201 |  |  |  |
| 191.38 | 0.137 | 241.38 | -0.008 | 291.38 | 0.008 |  | 341.38 | 0.189 | 391.38 | 0.192 |  |  |  |
| 191.4 | 0.139 | 241.4 | -0.002 | 291.4 | 0.031 |  | 341.4 | 0.188 | 391.4 | 0.186 |  |  |  |
| 191.42 | 0.159 | 241.42 | -0.014 | 291.42 | 0.128 |  | 341.42 | 0.19 | 391.42 | 0.149 |  |  |  |
| 191.44 | 0.264 | 241.44 | -0.02 | 291.44 | 0.173 |  | 341.44 | 0.191 | 391.44 | 0.141 |  |  |  |
| 191.46 | 0.36 | 241.46 | -0.023 | 291.46 | 0.157 |  | 341.46 | 0.201 | 391.46 | 0.205 |  |  |  |
| 191.48 | 0.369 | 241.48 | -0.025 | 291.48 | 0.16 |  | 341.48 | 0.179 | 391.48 | 0.22 |  |  |  |
| 191.5 | 0.289 | 241.5 | -0.027 | 291.5 | 0.176 |  | 341.5 | 0.149 | 391.5 | 0.206 |  |  |  |
| 191.52 | 0.191 | 241.52 | 0.023 | 291.52 | 0.193 |  | 341.52 | 0.143 | 391.52 | 0.189 |  |  |  |
| 191.54 | 0.138 | 241.54 | 0.018 | 291.54 | 0.212 |  | 341.54 | 0.177 | 391.54 | 0.203 |  |  |  |
| 191.56 | 0.072 | 241.56 | 0.016 | 291.56 | 0.23 |  | 341.56 | 0.179 | 391.56 | 0.233 |  |  |  |
| 191.58 | 0.067 | 241.58 | 0.044 | 291.58 | 0.231 |  | 341.58 | 0.17 | 391.58 | 0.208 |  |  |  |
| 191.6 | 0.073 | 241.6 | 0.056 | 291.6 | 0.224 |  | 341.6 | 0.159 | 391.6 | 0.135 |  |  |  |
| 191.62 | 0.072 | 241.62 | -0.035 | 291.62 | 0.211 |  | 341.62 | 0.177 | 391.62 | 0.095 |  |  |  |
| 191.64 | 0.073 | 241.64 | -0.036 | 291.64 | 0.206 |  | 341.64 | 0.193 | 391.64 | 0.095 |  |  |  |
| 191.66 | 0.081 | 241.66 | -0.035 | 291.66 | 0.212 |  | 341.66 | 0.188 | 391.66 | 0.121 |  |  |  |
| 191.68 | 0.084 | 241.68 | -0.033 | 291.68 | 0.215 |  | 341.68 | 0.169 | 391.68 | 0.126 |  |  |  |
| 191.7 | 0.085 | 241.7 | -0.029 | 291.7 | 0.217 |  | 341.7 | 0.177 | 391.7 | 0.128 |  |  |  |
| 191.72 | 0.087 | 241.72 | -0.014 | 291.72 | 0.015 |  | 341.72 | 0.175 | 391.72 | 0.13 |  |  |  |
| 191.74 | 0.073 | 241.74 | 0.007 | 291.74 | 0.025 |  | 341.74 | 0.176 | 391.74 | 0.137 |  |  |  |
| 191.76 | 0.073 | 241.76 | 0.029 | 291.76 | 0.068 |  | 341.76 | 0.179 | 391.76 | 0.14 |  |  |  |
| 191.78 | 0.098 | 241.78 | 0.043 | 291.78 | 0.084 |  | 341.78 | 0.182 | 391.78 | 0.146 |  |  |  |
| 191.8 | 0.108 | 241.8 | 0.051 | 291.8 | 0.083 |  | 341.8 | 0.189 | 391.8 | 0.118 |  |  |  |
| 191.82 | 0.109 | 241.82 | 0.061 | 291.82 | 0.077 |  | 341.82 | 0.18 | 391.82 | 0.131 |  |  |  |
| 191.84 | 0.111 | 241.84 | 0.056 | 291.84 | 0.086 |  | 341.84 | 0.18 | 391.84 | 0.213 |  |  |  |
| 191.86 | 0.117 | 241.86 | 0.029 | 291.86 | 0.097 |  | 341.86 | 0.184 | 391.86 | 0.189 |  |  |  |
| 191.88 | 0.12 | 241.88 | 0.007 | 291.88 | 0.108 |  | 341.88 | 0.191 | 391.88 | 0.227 |  |  |  |
| 191.9 | 0.121 | 241.9 | 0.001 | 291.9 | 0.121 |  | 341.9 | 0.189 | 391.9 | 0.236 |  |  |  |
| 191.92 | 0.115 | 241.92 | 0.001 | 291.92 | 0.139 |  | 341.92 | 0.213 | 391.92 | 0.239 |  |  |  |
| 191.94 | 0.081 | 241.94 | 0.005 | 291.94 | 0.152 |  | 341.94 | 0.227 | 391.94 | 0.236 |  |  |  |
| 191.96 | 0.173 | 241.96 | 0.01 | 291.96 | 0.153 |  | 341.96 | 0.246 | 391.96 | 0.225 |  |  |  |
| 191.98 | 0.126 | 241.98 | 0.011 | 291.98 | 0.152 |  | 341.98 | 0.249 | 391.98 | 0.182 |  |  |  |
| 192 | 0.149 | 242 | 0.007 | 292 | 0.171 |  | 342 | 0.25 | 392 | 0.149 |  |  |  |
| 192.02 | 0.161 | 242.02 | 0.012 | 292.02 | 0.177 |  | 342.02 | 0.251 | 392.02 | 0.187 |  |  |  |
| 192.04 | 0.162 | 242.04 | 0.019 | 292.04 | 0.233 |  | 342.04 | 0.247 | 392.04 | 0.159 |  |  |  |
| 192.06 | 0.159 | 242.06 | 0.02 | 292.06 | 0.319 |  | 342.06 | 0.244 | 392.06 | 0.153 |  |  |  |
| 192.08 | 0.158 | 242.08 | 0.011 | 292.08 | 0.357 |  | 342.08 | 0.254 | 392.08 | 0.156 |  |  |  |
| 192.1 | 0.169 | 242.1 | 0 | 292.1 | 0.271 |  | 342.1 | 0.25 | 392.1 | 0.146 |  |  |  |
| 192.12 | 0.171 | 242.12 | -0.004 | 292.12 | 0.016 |  | 342.12 | 0.223 | 392.12 | 0.116 |  |  |  |
| 192.14 | 0.177 | 242.14 | -0.001 | 292.14 | -0.052 |  | 342.14 | 0.21 | 392.14 | 0.189 |  |  |  |
| 192.16 | 0.178 | 242.16 | -0.001 | 292.16 | -0.069 |  | 342.16 | 0.219 | 392.16 | 0.188 |  |  |  |
| 192.18 | 0.178 | 242.18 | -0.002 | 292.18 | -0.068 |  | 342.18 | 0.25 | 392.18 | 0.192 |  |  |  |
| 192.2 | 0.18 | 242.2 | -0.004 | 292.2 | -0.061 |  | 342.2 | 0.264 | 392.2 | 0.188 |  |  |  |
| 192.22 | 0.18 | 242.22 | 0 | 292.22 | -0.05 |  | 342.22 | 0.259 | 392.22 | 0.194 |  |  |  |
| 192.24 | 0.182 | 242.24 | 0.005 | 292.24 | -0.039 |  | 342.24 | 0.246 | 392.24 | 0.18 |  |  |  |
| 192.26 | 0.177 | 242.26 | 0.014 | 292.26 | -0.024 |  | 342.26 | 0.225 | 392.26 | 0.18 |  |  |  |
| 192.28 | 0.172 | 242.28 | 0.02 | 292.28 | -0.009 |  | 342.28 | 0.201 | 392.28 | 0.184 |  |  |  |
| 192.3 | 0.169 | 242.3 | 0.025 | 292.3 | 0.003 |  | 342.3 | 0.177 | 392.3 | 0.182 |  |  |  |
| 192.32 | 0.169 | 242.32 | 0.018 | 292.32 | 0.012 |  | 342.32 | 0.222 | 392.32 | 0.17 |  |  |  |
| 192.34 | 0.155 | 242.34 | 0.005 | 292.34 | 0.017 |  | 342.34 | 0.235 | 392.34 | 0.163 |  |  |  |
| 192.36 | 0.134 | 242.36 | 0.013 | 292.36 | 0.032 |  | 342.36 | 0.231 | 392.36 | 0.183 |  |  |  |
| 192.38 | 0.121 | 242.38 | 0.019 | 292.38 | 0.049 |  | 342.38 | 0.214 | 392.38 | 0.179 |  |  |  |
| 192.4 | 0.111 | 242.4 | 0.015 | 292.4 | 0.059 |  | 342.4 | 0.19 | 392.4 | 0.175 |  |  |  |
| 192.42 | 0.104 | 242.42 | 0.017 | 292.42 | 0.069 |  | 342.42 | 0.159 | 392.42 | 0.157 |  |  |  |
| 192.44 | 0.11 | 242.44 | 0.024 | 292.44 | 0.085 |  | 342.44 | 0.147 | 392.44 | 0.152 |  |  |  |
| 192.46 | 0.12 | 242.46 | 0.032 | 292.46 | 0.1 |  | 342.46 | 0.156 | 392.46 | 0.163 |  |  |  |
| 192.48 | 0.156 | 242.48 | 0.043 | 292.48 | 0.112 |  | 342.48 | 0.16 | 392.48 | 0.147 |  |  |  |
| 192.5 | 0.218 | 242.5 | 0.046 | 292.5 | 0.116 |  | 342.5 | 0.16 | 392.5 | 0.095 |  |  |  |
| 192.52 | 0.255 | 242.52 | 0.043 | 292.52 | 0.008 |  | 342.52 | 0.146 | 392.52 | 0.097 |  |  |  |
| 192.54 | 0.245 | 242.54 | 0.045 | 292.54 | 0.005 |  | 342.54 | 0.221 | 392.54 | 0.053 |  |  |  |
| 192.56 | 0.176 | 242.56 | 0.055 | 292.56 | -0.029 |  | 342.56 | 0.272 | 392.56 | 0.16 |  |  |  |
| 192.58 | 0.153 | 242.58 | 0.097 | 292.58 | -0.032 |  | 342.58 | 0.382 | 392.58 | 0.184 |  |  |  |
| 192.6 | 0.155 | 242.6 | 0.086 | 292.6 | -0.021 |  | 342.6 | 0.2496 | 392.6 | 0.185 |  |  |  |
| 192.62 | 0.171 | 242.62 | 0.103 | 292.62 | -0.011 |  | 342.62 | 0.38 | 392.62 | 0.168 |  |  |  |
| 192.64 | 0.185 | 242.64 | 0.127 | 292.64 | -0.008 |  | 342.64 | 0.226 | 392.64 | 0.11 |  |  |  |
| 192.66 | 0.167 | 242.66 | 0.151 | 292.66 | 0.004 |  | 342.66 | 0.217 | 392.66 | 0.103 |  |  |  |
| 192.68 | 0.16 | 242.68 | 0.215 | 292.68 | 0.009 |  | 342.68 | 0.209 | 392.68 | 0.103 |  |  |  |
| 192.7 | 0.169 | 242.7 | 0.255 | 292.7 | 0.041 |  | 342.7 | 0.123 | 392.7 | 0.121 |  |  |  |
| 192.72 | 0.212 | 242.72 | 0.322 | 292.72 | 0.061 |  | 342.72 | 0.097 | 392.72 | 0.116 |  |  |  |
| 192.74 | 0.196 | 242.74 | 0.356 | 292.74 | 0.068 |  | 342.74 | 0.071 | 392.74 | 0.115 |  |  |  |
| 192.76 | 0.157 | 242.76 | 0.354 | 292.76 | 0.068 |  | 342.76 | 0.063 | 392.76 | 0.131 |  |  |  |
| 192.78 | 0.151 | 242.78 | 0.327 | 292.78 | 0.074 |  | 342.78 | 0.047 | 392.78 | 0.117 |  |  |  |
| 192.8 | 0.155 | 242.8 | 0.277 | 292.8 | 0.098 |  | 342.8 | 0.054 | 392.8 | 0.119 |  |  |  |
| 192.82 | 0.177 | 242.82 | 0.22 | 292.82 | 0.111 |  | 342.82 | 0.061 | 392.82 | 0.096 |  |  |  |
| 192.84 | 0.241 | 242.84 | 0.168 | 292.84 | 0.05 |  | 342.84 | 0.073 | 392.84 | 0.088 |  |  |  |
| 192.86 | 0.282 | 242.86 | 0.153 | 292.86 | 0.045 |  | 342.86 | 0.093 | 392.86 | 0.094 |  |  |  |
| 192.88 | 0.275 | 242.88 | 0.119 | 292.88 | 0.095 |  | 342.88 | 0.115 | 392.88 | 0.096 |  |  |  |
| 192.9 | 0.206 | 242.9 | 0.084 | 292.9 | 0.129 |  | 342.9 | 0.137 | 392.9 | 0.101 |  |  |  |
| 192.92 | 0.165 | 242.92 | 0.044 | 292.92 | 0.009 |  | 342.92 | 0.148 | 392.92 | 0.107 |  |  |  |
| 192.94 | 0.158 | 242.94 | 0.087 | 292.94 | 0.2466 |  | 342.94 | 0.156 | 392.94 | 0.106 |  |  |  |
| 192.96 | 0.177 | 242.96 | 0.073 | 292.96 | 0.295 |  | 342.96 | 0.162 | 392.96 | 0.098 |  |  |  |
| 192.98 | 0.165 | 242.98 | 0.044 | 292.98 | 0.149 |  | 342.98 | 0.165 | 392.98 | 0.123 |  |  |  |
| 193 | 0.093 | 243 | 0.054 | 293 | 0.07 |  | 343 | 0.168 | 393 | 0.121 |  |  |  |
| 193.02 | 0.099 | 243.02 | 0.034 | 293.02 | 0.055 |  | 343.02 | 0.176 | 393.02 | 0.127 |  |  |  |
| 193.04 | 0.132 | 243.04 | 0.03 | 293.04 | 0.061 |  | 343.04 | 0.182 | 393.04 | 0.126 |  |  |  |
| 193.06 | 0.139 | 243.06 | 0.026 | 293.06 | 0.066 |  | 343.06 | 0.192 | 393.06 | 0.132 |  |  |  |
| 193.08 | 0.13 | 243.08 | 0.032 | 293.08 | 0.071 |  | 343.08 | 0.218 | 393.08 | 0.115 |  |  |  |
| 193.1 | 0.1 | 243.1 | 0.032 | 293.1 | 0.072 |  | 343.1 | 0.22 | 393.1 | 0.133 |  |  |  |
| 193.12 | 0.09 | 243.12 | 0.031 | 293.12 | 0.077 |  | 343.12 | 0.22 | 393.12 | 0.136 |  |  |  |
| 193.14 | 0.123 | 243.14 | 0.041 | 293.14 | 0.077 |  | 343.14 | 0.228 | 393.14 | 0.133 |  |  |  |
| 193.16 | 0.173 | 243.16 | 0.047 | 293.16 | 0.079 |  | 343.16 | 0.194 | 393.16 | 0.131 |  |  |  |
| 193.18 | 0.196 | 243.18 | 0.093 | 293.18 | 0.09 |  | 343.18 | 0.184 | 393.18 | 0.142 |  |  |  |
| 193.2 | 0.17 | 243.2 | 0.125 | 293.2 | 0.101 |  | 343.2 | 0.173 | 393.2 | 0.152 |  |  |  |
| 193.22 | 0.186 | 243.22 | 0.052 | 293.22 | 0.11 |  | 343.22 | 0.183 | 393.22 | 0.118 |  |  |  |
| 193.24 | 0.223 | 243.24 | 0.074 | 293.24 | 0.112 |  | 343.24 | 0.172 | 393.24 | 0.15 |  |  |  |
| 193.26 | 0.201 | 243.26 | 0.215 | 293.26 | 0.113 |  | 343.26 | 0.194 | 393.26 | 0.141 |  |  |  |
| 193.28 | 0.19 | 243.28 | 0.211 | 293.28 | 0.116 |  | 343.28 | 0.196 | 393.28 | 0.133 |  |  |  |
| 193.3 | 0.152 | 243.3 | 0.185 | 293.3 | 0.106 |  | 343.3 | 0.197 | 393.3 | 0.13 |  |  |  |
| 193.32 | 0.132 | 243.32 | 0.149 | 293.32 | 0.014 |  | 343.32 | 0.199 | 393.32 | 0.125 |  |  |  |
| 193.34 | 0.112 | 243.34 | 0.041 | 293.34 | 0.017 |  | 343.34 | 0.202 | 393.34 | 0.151 |  |  |  |
| 193.36 | 0.147 | 243.36 | 0.065 | 293.36 | 0.013 |  | 343.36 | 0.206 | 393.36 | 0.184 |  |  |  |
| 193.38 | 0.217 | 243.38 | 0.08 | 293.38 | 0.015 |  | 343.38 | 0.204 | 393.38 | 0.187 |  |  |  |
| 193.4 | 0.259 | 243.4 | 0.007 | 293.4 | 0.012 |  | 343.4 | 0.217 | 393.4 | 0.179 |  |  |  |
| 193.42 | 0.245 | 243.42 | 0.088 | 293.42 | 0.017 |  | 343.42 | 0.201 | 393.42 | 0.16 |  |  |  |
| 193.44 | 0.197 | 243.44 | -0.005 | 293.44 | 0.018 |  | 343.44 | 0.197 | 393.44 | 0.146 |  |  |  |
| 193.46 | 0.167 | 243.46 | 0.115 | 293.46 | 0.023 |  | 343.46 | 0.191 | 393.46 | 0.154 |  |  |  |
| 193.48 | 0.137 | 243.48 | 0.11 | 293.48 | 0.026 |  | 343.48 | 0.181 | 393.48 | 0.158 |  |  |  |
| 193.5 | 0.083 | 243.5 | 0.066 | 293.5 | 0.073 |  | 343.5 | 0.213 | 393.5 | 0.181 |  |  |  |
| 193.52 | 0.066 | 243.52 | 0.085 | 293.52 | 0.086 |  | 343.52 | 0.191 | 393.52 | 0.153 |  |  |  |
| 193.54 | 0.091 | 243.54 | 0.096 | 293.54 | 0.091 |  | 343.54 | 0.189 | 393.54 | 0.102 |  |  |  |
| 193.56 | 0.113 | 243.56 | 0.073 | 293.56 | 0.1 |  | 343.56 | 0.172 | 393.56 | 0.119 |  |  |  |
| 193.58 | 0.142 | 243.58 | 0.037 | 293.58 | 0.104 |  | 343.58 | 0.164 | 393.58 | 0.157 |  |  |  |
| 193.6 | 0.152 | 243.6 | 0.021 | 293.6 | 0.101 |  | 343.6 | 0.163 | 393.6 | 0.133 |  |  |  |
| 193.62 | 0.145 | 243.62 | 0.028 | 293.62 | 0.105 |  | 343.62 | 0.093 | 393.62 | 0.151 |  |  |  |
| 193.64 | 0.112 | 243.64 | 0.027 | 293.64 | 0.105 |  | 343.64 | 0.159 | 393.64 | 0.155 |  |  |  |
| 193.66 | 0.11 | 243.66 | 0.023 | 293.66 | 0.108 |  | 343.66 | 0.245 | 393.66 | 0.144 |  |  |  |
| 193.68 | 0.182 | 243.68 | 0.015 | 293.68 | 0.112 |  | 343.68 | 0.281 | 393.68 | 0.136 |  |  |  |
| 193.7 | 0.201 | 243.7 | 0.001 | 293.7 | 0.117 |  | 343.7 | 0.279 | 393.7 | 0.14 |  |  |  |
| 193.72 | 0.203 | 243.72 | 0.035 | 293.72 | 0.008 |  | 343.72 | 0.293 | 393.72 | 0.135 |  |  |  |
| 193.74 | 0.156 | 243.74 | 0.074 | 293.74 | 0.007 |  | 343.74 | 0.253 | 393.74 | 0.131 |  |  |  |
| 193.76 | 0.148 | 243.76 | 0.089 | 293.76 | 0.004 |  | 343.76 | 0.203 | 393.76 | 0.13 |  |  |  |
| 193.78 | 0.139 | 243.78 | 0.082 | 293.78 | 0.002 |  | 343.78 | 0.191 | 393.78 | 0.138 |  |  |  |
| 193.8 | 0.193 | 243.8 | 0.082 | 293.8 | 0.004 |  | 343.8 | 0.212 | 393.8 | 0.149 |  |  |  |
| 193.82 | 0.231 | 243.82 | 0.1 | 293.82 | 0.011 |  | 343.82 | 0.247 | 393.82 | 0.127 |  |  |  |
| 193.84 | 0.217 | 243.84 | 0.093 | 293.84 | 0.018 |  | 343.84 | 0.252 | 393.84 | 0.109 |  |  |  |
| 193.86 | 0.189 | 243.86 | 0.084 | 293.86 | 0.027 |  | 343.86 | 0.241 | 393.86 | 0.147 |  |  |  |
| 193.88 | 0.196 | 243.88 | 0.083 | 293.88 | 0.037 |  | 343.88 | 0.242 | 393.88 | 0.143 |  |  |  |
| 193.9 | 0.211 | 243.9 | 0.098 | 293.9 | 0.052 |  | 343.9 | 0.241 | 393.9 | 0.14 |  |  |  |
| 193.92 | 0.19 | 243.92 | 0.115 | 293.92 | 0.06 |  | 343.92 | 0.233 | 393.92 | 0.227 |  |  |  |
| 193.94 | 0.143 | 243.94 | 0.124 | 293.94 | 0.064 |  | 343.94 | 0.22 | 393.94 | 0.133 |  |  |  |
| 193.96 | 0.151 | 243.96 | 0.128 | 293.96 | 0.069 |  | 343.96 | 0.198 | 393.96 | 0.127 |  |  |  |
| 193.98 | 0.179 | 243.98 | 0.115 | 293.98 | 0.076 |  | 343.98 | 0.196 | 393.98 | 0.147 |  |  |  |
| 194 | 0.168 | 244 | 0.124 | 294 | 0.082 |  | 344 | 0.182 | 394 | 0.179 |  |  |  |
| 194.02 | 0.141 | 244.02 | 0.128 | 294.02 | 0.088 |  | 344.02 | 0.171 | 394.02 | 0.189 |  |  |  |
| 194.04 | 0.37 | 244.04 | 0.115 | 294.04 | 0.098 |  | 344.04 | 0.173 | 394.04 | 0.19 |  |  |  |
| 194.06 | 0.174 | 244.06 | 0.135 | 294.06 | 0.104 |  | 344.06 | 0.174 | 394.06 | 0.195 |  |  |  |
| 194.08 | 0.184 | 244.08 | 0.169 | 294.08 | 0.108 |  | 344.08 | 0.164 | 394.08 | 0.19 |  |  |  |
| 194.1 | 0.186 | 244.1 | 0.166 | 294.1 | 0.106 |  | 344.1 | 0.153 | 394.1 | 0.198 |  |  |  |
| 194.12 | 0.206 | 244.12 | 0.137 | 294.12 | 0.102 |  | 344.12 | 0.145 | 394.12 | 0.181 |  |  |  |
| 194.14 | 0.242 | 244.14 | 0.122 | 294.14 | 0.091 |  | 344.14 | 0.149 | 394.14 | 0.176 |  |  |  |
| 194.16 | 0.278 | 244.16 | 0.127 | 294.16 | 0.089 |  | 344.16 | 0.159 | 394.16 | 0.192 |  |  |  |
| 194.18 | 0.232 | 244.18 | 0.135 | 294.18 | 0.094 |  | 344.18 | 0.161 | 394.18 | 0.191 |  |  |  |
| 194.2 | 0.187 | 244.2 | 0.146 | 294.2 | 0.095 |  | 344.2 | 0.153 | 394.2 | 0.179 |  |  |  |
| 194.22 | 0.176 | 244.22 | 0.162 | 294.22 | 0.092 |  | 344.22 | 0.149 | 394.22 | 0.153 |  |  |  |
| 194.24 | 0.192 | 244.24 | 0.168 | 294.24 | 0.08 |  | 344.24 | 0.139 | 394.24 | 0.16 |  |  |  |
| 194.26 | 0.194 | 244.26 | 0.007 | 294.26 | 0.089 |  | 344.26 | 0.145 | 394.26 | 0.195 |  |  |  |
| 194.28 | 0.183 | 244.28 | 0.007 | 294.28 | 0.089 |  | 344.28 | 0.139 | 394.28 | 0.188 |  |  |  |
| 194.3 | 0.164 | 244.3 | 0.003 | 294.3 | 0.09 |  | 344.3 | 0.135 | 394.3 | 0.169 |  |  |  |
| 194.32 | 0.142 | 244.32 | -0.055 | 294.32 | 0.092 |  | 344.32 | 0.133 | 394.32 | 0.181 |  |  |  |
| 194.34 | 0.142 | 244.34 | -0.044 | 294.34 | 0.095 |  | 344.34 | 0.144 | 394.34 | 0.178 |  |  |  |
| 194.36 | 0.152 | 244.36 | 0.023 | 294.36 | 0.096 |  | 344.36 | 0.147 | 394.36 | 0.152 |  |  |  |
| 194.38 | 0.155 | 244.38 | 0.035 | 294.38 | 0.097 |  | 344.38 | 0.139 | 394.38 | 0.119 |  |  |  |
| 194.4 | 0.149 | 244.4 | 0 | 294.4 | 0.095 |  | 344.4 | 0.13 | 394.4 | 0.169 |  |  |  |
| 194.42 | 0.143 | 244.42 | 0.048 | 294.42 | 0.094 |  | 344.42 | 0.129 | 394.42 | 0.178 |  |  |  |
| 194.44 | 0.142 | 244.44 | 0.078 | 294.44 | 0.1 |  | 344.44 | 0.143 | 394.44 | 0.158 |  |  |  |
| 194.46 | 0.155 | 244.46 | 0.089 | 294.46 | 0.101 |  | 344.46 | 0.154 | 394.46 | 0.156 |  |  |  |
| 194.48 | 0.186 | 244.48 | 0.075 | 294.48 | 0.103 |  | 344.48 | 0.149 | 394.48 | 0.174 |  |  |  |
| 194.5 | 0.215 | 244.5 | 0.065 | 294.5 | 0.104 |  | 344.5 | 0.122 | 394.5 | 0.197 |  |  |  |
| 194.52 | 0.22 | 244.52 | 0.094 | 294.52 | 0.137 |  | 344.52 | 0.109 | 394.52 | 0.208 |  |  |  |
| 194.54 | 0.207 | 244.54 | 0.121 | 294.54 | 0.132 |  | 344.54 | 0.116 | 394.54 | 0.158 |  |  |  |
| 194.56 | 0.189 | 244.56 | 0.144 | 294.56 | 0.133 |  | 344.56 | 0.118 | 394.56 | 0.155 |  |  |  |
| 194.58 | 0.172 | 244.58 | 0.155 | 294.58 | 0.136 |  | 344.58 | 0.118 | 394.58 | 0.157 |  |  |  |
| 194.6 | 0.163 | 244.6 | 0.164 | 294.6 | 0.221 |  | 344.6 | 0.135 | 394.6 | 0.189 |  |  |  |
| 194.62 | 0.169 | 244.62 | 0.164 | 294.62 | 0.216 |  | 344.62 | 0.154 | 394.62 | 0.188 |  |  |  |
| 194.64 | 0.187 | 244.64 | 0.148 | 294.64 | 0.166 |  | 344.64 | 0.166 | 394.64 | 0.155 |  |  |  |
| 194.66 | 0.192 | 244.66 | 0.157 | 294.66 | 0.159 |  | 344.66 | 0.186 | 394.66 | 0.168 |  |  |  |
| 194.68 | 0.191 | 244.68 | 0.158 | 294.68 | 0.139 |  | 344.68 | 0.194 | 394.68 | 0.157 |  |  |  |
| 194.7 | 0.1 | 244.7 | 0.179 | 294.7 | 0.141 |  | 344.7 | 0.202 | 394.7 | 0.169 |  |  |  |
| 194.72 | 0.033 | 244.72 | 0.168 | 294.72 | 0.144 |  | 344.72 | 0.207 | 394.72 | 0.183 |  |  |  |
| 194.74 | 0.108 | 244.74 | 0.141 | 294.74 | 0.151 |  | 344.74 | 0.203 | 394.74 | 0.166 |  |  |  |
| 194.76 | 0.115 | 244.76 | 0.144 | 294.76 | 0.149 |  | 344.76 | 0.208 | 394.76 | 0.143 |  |  |  |
| 194.78 | 0.079 | 244.78 | 0.145 | 294.78 | 0.145 |  | 344.78 | 0.214 | 394.78 | 0.146 |  |  |  |
| 194.8 | 0.059 | 244.8 | 0.13 | 294.8 | 0.143 |  | 344.8 | 0.208 | 394.8 | 0.154 |  |  |  |
| 194.82 | 0.073 | 244.82 | 0.124 | 294.82 | 0.142 |  | 344.82 | 0.204 | 394.82 | 0.153 |  |  |  |
| 194.84 | 0.122 | 244.84 | 0.145 | 294.84 | 0.137 |  | 344.84 | 0.191 | 394.84 | 0.16 |  |  |  |
| 194.86 | 0.158 | 244.86 | 0.156 | 294.86 | 0.136 |  | 344.86 | 0.212 | 394.86 | 0.191 |  |  |  |
| 194.88 | 0.196 | 244.88 | 0.148 | 294.88 | 0.129 |  | 344.88 | 0.205 | 394.88 | 0.224 |  |  |  |
| 194.9 | 0.257 | 244.9 | 0.129 | 294.9 | 0.123 |  | 344.9 | 0.196 | 394.9 | 0.27 |  |  |  |
| 194.92 | 0.29 | 244.92 | 0.173 | 294.92 | 0.031 |  | 344.92 | 0.198 | 394.92 | 0.269 |  |  |  |
| 194.94 | 0.27 | 244.94 | 0.18 | 294.94 | 0.031 |  | 344.94 | 0.206 | 394.94 | 0.217 |  |  |  |
| 194.96 | 0.214 | 244.96 | 0.172 | 294.96 | 0.033 |  | 344.96 | 0.211 | 394.96 | 0.141 |  |  |  |
| 194.98 | 0.156 | 244.98 | 0.162 | 294.98 | 0.034 |  | 344.98 | 0.215 | 394.98 | 0.139 |  |  |  |
| 195 | 0.123 | 245 | 0.155 | 295 | 0.037 |  | 345 | 0.216 | 395 | 0.147 |  |  |  |
| 195.02 | 0.124 | 245.02 | 0.159 | 295.02 | 0.039 |  | 345.02 | 0.227 | 395.02 | 0.172 |  |  |  |
| 195.04 | 0.144 | 245.04 | 0.161 | 295.04 | 0.039 |  | 345.04 | 0.225 | 395.04 | 0.145 |  |  |  |
| 195.06 | 0.148 | 245.06 | 0.008 | 295.06 | 0.055 |  | 345.06 | 0.214 | 395.06 | 0.12 |  |  |  |
| 195.08 | 0.156 | 245.08 | -0.007 | 295.08 | 0.063 |  | 345.08 | 0.229 | 395.08 | 0.113 |  |  |  |
| 195.1 | 0.009 | 245.1 | -0.011 | 295.1 | 0.069 |  | 345.1 | 0.228 | 395.1 | 0.111 |  |  |  |
| 195.12 | 0.015 | 245.12 | -0.013 | 295.12 | 0.099 |  | 345.12 | 0.22 | 395.12 | 0.129 |  |  |  |
| 195.14 | 0.018 | 245.14 | 0.039 | 295.14 | 0.135 |  | 345.14 | 0.215 | 395.14 | 0.136 |  |  |  |
| 195.16 | 0.017 | 245.16 | 0.056 | 295.16 | 0.15 |  | 345.16 | 0.201 | 395.16 | 0.173 |  |  |  |
| 195.18 | 0.025 | 245.18 | 0.036 | 295.18 | 0.147 |  | 345.18 | 0.192 | 395.18 | 0.197 |  |  |  |
| 195.2 | 0.03 | 245.2 | 0.038 | 295.2 | 0.148 |  | 345.2 | 0.173 | 395.2 | 0.168 |  |  |  |
| 195.22 | 0.02 | 245.22 | 0.045 | 295.22 | 0.15 |  | 345.22 | 0.176 | 395.22 | 0.134 |  |  |  |
| 195.24 | 0.023 | 245.24 | 0.029 | 295.24 | 0.145 |  | 345.24 | 0.215 | 395.24 | 0.126 |  |  |  |
| 195.26 | 0.029 | 245.26 | 0.047 | 295.26 | 0.099 |  | 345.26 | 0.194 | 395.26 | 0.119 |  |  |  |
| 195.28 | 0.059 | 245.28 | 0.06 | 295.28 | 0.082 |  | 345.28 | 0.188 | 395.28 | 0.117 |  |  |  |
| 195.3 | 0.168 | 245.3 | 0.073 | 295.3 | 0.137 |  | 345.3 | 0.182 | 395.3 | 0.128 |  |  |  |
| 195.32 | 0.264 | 245.32 | 0.087 | 295.32 | 0.012 |  | 345.32 | 0.179 | 395.32 | 0.159 |  |  |  |
| 195.34 | 0.305 | 245.34 | 0.102 | 295.34 | 0.023 |  | 345.34 | 0.155 | 395.34 | 0.185 |  |  |  |
| 195.36 | 0.21 | 245.36 | 0.139 | 295.36 | 0.02 |  | 345.36 | 0.127 | 395.36 | 0.143 |  |  |  |
| 195.38 | 0.132 | 245.38 | 0.179 | 295.38 | 0.026 |  | 345.38 | 0.126 | 395.38 | 0.134 |  |  |  |
| 195.4 | 0.115 | 245.4 | 0.22 | 295.4 | 0.032 |  | 345.4 | 0.127 | 395.4 | 0.145 |  |  |  |
| 195.42 | 0.105 | 245.42 | 0.213 | 295.42 | 0.032 |  | 345.42 | 0.132 | 395.42 | 0.143 |  |  |  |
| 195.44 | 0.115 | 245.44 | 0.185 | 295.44 | 0.03 |  | 345.44 | 0.14 | 395.44 | 0.151 |  |  |  |
| 195.46 | 0.117 | 245.46 | 0.15 | 295.46 | 0.036 |  | 345.46 | 0.165 | 395.46 | 0.168 |  |  |  |
| 195.48 | 0.084 | 245.48 | 0.095 | 295.48 | 0.05 |  | 345.48 | 0.19 | 395.48 | 0.192 |  |  |  |
| 195.5 | 0.108 | 245.5 | 0.089 | 295.5 | 0.071 |  | 345.5 | 0.222 | 395.5 | 0.165 |  |  |  |
| 195.52 | 0.15 | 245.52 | 0.09 | 295.52 | 0.062 |  | 345.52 | 0.24 | 395.52 | 0.135 |  |  |  |
| 195.54 | 0.18 | 245.54 | 0.084 | 295.54 | 0.037 |  | 345.54 | 0.228 | 395.54 | 0.139 |  |  |  |
| 195.56 | 0.177 | 245.56 | 0.085 | 295.56 | 0.039 |  | 345.56 | 0.217 | 395.56 | 0.172 |  |  |  |
| 195.58 | 0.096 | 245.58 | 0.087 | 295.58 | 0.036 |  | 345.58 | 0.215 | 395.58 | 0.158 |  |  |  |
| 195.6 | 0.149 | 245.6 | 0.104 | 295.6 | 0.042 |  | 345.6 | 0.213 | 395.6 | 0.175 |  |  |  |
| 195.62 | 0.198 | 245.62 | 0.148 | 295.62 | 0.048 |  | 345.62 | 0.208 | 395.62 | 0.186 |  |  |  |
| 195.64 | 0.219 | 245.64 | 0.152 | 295.64 | 0.057 |  | 345.64 | 0.205 | 395.64 | 0.149 |  |  |  |
| 195.66 | 0.22 | 245.66 | 0.218 | 295.66 | 0.063 |  | 345.66 | 0.203 | 395.66 | 0.125 |  |  |  |
| 195.68 | 0.211 | 245.68 | 0.314 | 295.68 | 0.069 |  | 345.68 | 0.214 | 395.68 | 0.139 |  |  |  |
| 195.7 | 0.2 | 245.7 | 0.321 | 295.7 | 0.079 |  | 345.7 | 0.226 | 395.7 | 0.153 |  |  |  |
| 195.72 | 0.192 | 245.72 | 0.227 | 295.72 | 0.005 |  | 345.72 | 0.225 | 395.72 | 0.146 |  |  |  |
| 195.74 | 0.187 | 245.74 | 0.144 | 295.74 | 0.008 |  | 345.74 | 0.224 | 395.74 | 0.135 |  |  |  |
| 195.76 | 0.185 | 245.76 | 0.05 | 295.76 | 0.017 |  | 345.76 | 0.21 | 395.76 | 0.154 |  |  |  |
| 195.78 | 0.187 | 245.78 | 0.052 | 295.78 | 0.031 |  | 345.78 | 0.214 | 395.78 | 0.183 |  |  |  |
| 195.8 | 0.19 | 245.8 | 0.055 | 295.8 | 0.023 |  | 345.8 | 0.202 | 395.8 | 0.19 |  |  |  |
| 195.82 | 0.195 | 245.82 | 0.063 | 295.82 | 0.026 |  | 345.82 | 0.201 | 395.82 | 0.13 |  |  |  |
| 195.84 | 0.232 | 245.84 | 0.07 | 295.84 | 0.024 |  | 345.84 | 0.195 | 395.84 | 0.136 |  |  |  |
| 195.86 | 0.25 | 245.86 | 0.072 | 295.86 | 0.033 |  | 345.86 | 0.201 | 395.86 | 0.117 |  |  |  |
| 195.88 | 0.208 | 245.88 | 0.078 | 295.88 | 0.032 |  | 345.88 | 0.207 | 395.88 | 0.149 |  |  |  |
| 195.9 | 0.166 | 245.9 | 0.1 | 295.9 | 0.032 |  | 345.9 | 0.21 | 395.9 | 0.177 |  |  |  |
| 195.92 | 0.149 | 245.92 | 0.04 | 295.92 | 0.039 |  | 345.92 | 0.215 | 395.92 | 0.192 |  |  |  |
| 195.94 | 0.138 | 245.94 | 0.048 | 295.94 | 0.053 |  | 345.94 | 0.224 | 395.94 | 0.176 |  |  |  |
| 195.96 | 0.133 | 245.96 | 0.054 | 295.96 | 0.071 |  | 345.96 | 0.232 | 395.96 | 0.169 |  |  |  |
| 195.98 | 0.138 | 245.98 | 0.063 | 295.98 | 0.089 |  | 345.98 | 0.232 | 395.98 | 0.183 |  |  |  |
| 196 | 0.146 | 246 | 0.084 | 296 | 0.088 |  | 346 | 0.223 | 396 | 0.178 |  |  |  |
| 196.02 | 0.148 | 246.02 | 0.12 | 296.02 | 0.088 |  | 346.02 | 0.227 | 396.02 | 0.175 |  |  |  |
| 196.04 | 0.156 | 246.04 | 0.157 | 296.04 | 0.091 |  | 346.04 | 0.226 | 396.04 | 0.17 |  |  |  |
| 196.06 | 0.17 | 246.06 | 0.135 | 296.06 | 0.102 |  | 346.06 | 0.229 | 396.06 | 0.135 |  |  |  |
| 196.08 | 0.198 | 246.08 | 0.111 | 296.08 | 0.112 |  | 346.08 | 0.227 | 396.08 | 0.152 |  |  |  |
| 196.1 | 0.235 | 246.1 | 0.08 | 296.1 | 0.12 |  | 346.1 | 0.236 | 396.1 | 0.143 |  |  |  |
| 196.12 | 0.27 | 246.12 | 0.053 | 296.12 | 0.206 |  | 346.12 | 0.246 | 396.12 | 0.097 |  |  |  |
| 196.14 | 0.292 | 246.14 | 0.061 | 296.14 | 0.197 |  | 346.14 | 0.251 | 396.14 | 0.143 |  |  |  |
| 196.16 | 0.28 | 246.16 | 0.117 | 296.16 | 0.195 |  | 346.16 | 0.197 | 396.16 | 0.121 |  |  |  |
| 196.18 | 0.243 | 246.18 | 0.126 | 296.18 | 0.185 |  | 346.18 | 0.212 | 396.18 | 0.096 |  |  |  |
| 196.2 | 0.2 | 246.2 | 0.125 | 296.2 | 0.155 |  | 346.2 | 0.177 | 396.2 | 0.187 |  |  |  |
| 196.22 | 0.179 | 246.22 | 0.132 | 296.22 | 0.16 |  | 346.22 | 0.147 | 396.22 | 0.2 |  |  |  |
| 196.24 | 0.171 | 246.24 | 0.155 | 296.24 | 0.167 |  | 346.24 | 0.055 | 396.24 | 0.207 |  |  |  |
| 196.26 | 0.131 | 246.26 | 0.194 | 296.26 | 0.198 |  | 346.26 | 0.033 | 396.26 | 0.201 |  |  |  |
| 196.28 | 0.154 | 246.28 | 0.227 | 296.28 | 0.196 |  | 346.28 | 0.156 | 396.28 | 0.164 |  |  |  |
| 196.3 | 0.176 | 246.3 | 0.242 | 296.3 | 0.185 |  | 346.3 | 0.263 | 396.3 | 0.155 |  |  |  |
| 196.32 | 0.191 | 246.32 | 0.26 | 296.32 | 0.188 |  | 346.32 | 0.252 | 396.32 | 0.243 |  |  |  |
| 196.34 | 0.234 | 246.34 | 0.275 | 296.34 | 0.196 |  | 346.34 | 0.248 | 396.34 | 0.236 |  |  |  |
| 196.36 | 0.235 | 246.36 | 0.237 | 296.36 | 0.199 |  | 346.36 | 0.254 | 396.36 | 0.159 |  |  |  |
| 196.38 | 0.209 | 246.38 | 0.215 | 296.38 | 0.202 |  | 346.38 | 0.258 | 396.38 | 0.158 |  |  |  |
| 196.4 | 0.185 | 246.4 | 0.144 | 296.4 | 0.198 |  | 346.4 | 0.25 | 396.4 | 0.168 |  |  |  |
| 196.42 | 0.187 | 246.42 | 0.095 | 296.42 | 0.194 |  | 346.42 | 0.235 | 396.42 | 0.121 |  |  |  |
| 196.44 | 0.194 | 246.44 | 0.084 | 296.44 | 0.187 |  | 346.44 | 0.217 | 396.44 | 0.08 |  |  |  |
| 196.46 | 0.212 | 246.46 | 0.083 | 296.46 | 0.196 |  | 346.46 | 0.213 | 396.46 | 0.131 |  |  |  |
| 196.48 | 0.216 | 246.48 | 0.083 | 296.48 | 0.205 |  | 346.48 | 0.22 | 396.48 | 0.171 |  |  |  |
| 196.5 | 0.206 | 246.5 | 0.076 | 296.5 | 0.207 |  | 346.5 | 0.22 | 396.5 | 0.203 |  |  |  |
| 196.52 | 0.27 | 246.52 | 0.053 | 296.52 | 0.014 |  | 346.52 | 0.197 | 396.52 | 0.209 |  |  |  |
| 196.54 | 0.263 | 246.54 | 0.063 | 296.54 | 0.02 |  | 346.54 | 0.211 | 396.54 | 0.207 |  |  |  |
| 196.56 | 0.173 | 246.56 | 0.126 | 296.56 | 0.035 |  | 346.56 | 0.232 | 396.56 | 0.185 |  |  |  |
| 196.58 | 0.133 | 246.58 | 0.189 | 296.58 | 0.027 |  | 346.58 | 0.236 | 396.58 | 0.153 |  |  |  |
| 196.6 | 0.12 | 246.6 | 0.204 | 296.6 | -0.007 |  | 346.6 | 0.242 | 396.6 | 0.123 |  |  |  |
| 196.62 | 0.133 | 246.62 | 0.136 | 296.62 | 0.001 |  | 346.62 | 0.235 | 396.62 | 0.104 |  |  |  |
| 196.64 | 0.148 | 246.64 | 0.092 | 296.64 | 0.031 |  | 346.64 | 0.215 | 396.64 | 0.117 |  |  |  |
| 196.66 | 0.144 | 246.66 | 0.071 | 296.66 | 0.044 |  | 346.66 | 0.201 | 396.66 | 0.107 |  |  |  |
| 196.68 | 0.151 | 246.68 | 0.068 | 296.68 | 0.034 |  | 346.68 | 0.216 | 396.68 | 0.105 |  |  |  |
| 196.7 | 0.187 | 246.7 | 0.073 | 296.7 | 0.011 |  | 346.7 | 0.231 | 396.7 | 0.088 |  |  |  |
| 196.72 | 0.269 | 246.72 | 0.076 | 296.72 | 0.028 |  | 346.72 | 0.21 | 396.72 | 0.091 |  |  |  |
| 196.74 | 0.321 | 246.74 | 0.084 | 296.74 | 0.078 |  | 346.74 | 0.232 | 396.74 | 0.092 |  |  |  |
| 196.76 | 0.29 | 246.76 | 0.095 | 296.76 | 0.086 |  | 346.76 | 0.257 | 396.76 | 0.093 |  |  |  |
| 196.78 | 0.214 | 246.78 | 0.092 | 296.78 | 0.106 |  | 346.78 | 0.133 | 396.78 | 0.088 |  |  |  |
| 196.8 | 0.159 | 246.8 | 0.085 | 296.8 | 0.126 |  | 346.8 | 0.238 | 396.8 | 0.086 |  |  |  |
| 196.82 | 0.217 | 246.82 | 0.093 | 296.82 | 0.118 |  | 346.82 | 0.288 | 396.82 | 0.083 |  |  |  |
| 196.84 | 0.228 | 246.84 | 0.099 | 296.84 | 0.095 |  | 346.84 | 0.253 | 396.84 | 0.084 |  |  |  |
| 196.86 | 0.207 | 246.86 | 0.098 | 296.86 | 0.105 |  | 346.86 | 0.262 | 396.86 | 0.085 |  |  |  |
| 196.88 | 0.152 | 246.88 | 0.088 | 296.88 | 0.162 |  | 346.88 | 0.304 | 396.88 | 0.081 |  |  |  |
| 196.9 | 0.127 | 246.9 | 0.171 | 296.9 | 0.193 |  | 346.9 | 0.302 | 396.9 | 0.084 |  |  |  |
| 196.92 | 0.144 | 246.92 | 0.149 | 296.92 | 0.263 |  | 346.92 | 0.142 | 396.92 | 0.087 |  |  |  |
| 196.94 | 0.127 | 246.94 | 0.12 | 296.94 | 0.255 |  | 346.94 | 0.074 | 396.94 | 0.097 |  |  |  |
| 196.96 | 0.133 | 246.96 | 0.106 | 296.96 | 0.232 |  | 346.96 | 0.09 | 396.96 | 0.099 |  |  |  |
| 196.98 | 0.149 | 246.98 | 0.094 | 296.98 | 0.315 |  | 346.98 | 0.098 | 396.98 | 0.104 |  |  |  |
| 197 | 0.177 | 247 | 0.136 | 297 | 0.192 |  | 347 | 0.117 | 397 | 0.106 |  |  |  |
| 197.02 | 0.18 | 247.02 | 0.171 | 297.02 | 0.178 |  | 347.02 | 0.058 | 397.02 | 0.105 |  |  |  |
| 197.04 | 0.167 | 247.04 | 0.168 | 297.04 | 0.167 |  | 347.04 | 0.105 | 397.04 | 0.107 |  |  |  |
| 197.06 | 0.145 | 247.06 | 0.154 | 297.06 | 0.155 |  | 347.06 | 0.091 | 397.06 | 0.12 |  |  |  |
| 197.08 | 0.168 | 247.08 | 0.149 | 297.08 | 0.15 |  | 347.08 | 0.075 | 397.08 | 0.137 |  |  |  |
| 197.1 | 0.117 | 247.1 | 0.146 | 297.1 | 0.153 |  | 347.1 | 0.097 | 397.1 | 0.136 |  |  |  |
| 197.12 | 0.087 | 247.12 | 0.131 | 297.12 | 0.152 |  | 347.12 | 0.098 | 397.12 | 0.153 |  |  |  |
| 197.14 | 0.107 | 247.14 | 0.105 | 297.14 | 0.147 |  | 347.14 | 0.077 | 397.14 | 0.186 |  |  |  |
| 197.16 | 0.159 | 247.16 | 0.107 | 297.16 | 0.152 |  | 347.16 | 0.137 | 397.16 | 0.167 |  |  |  |
| 197.18 | 0.179 | 247.18 | 0.125 | 297.18 | 0.174 |  | 347.18 | 0.172 | 397.18 | 0.157 |  |  |  |
| 197.2 | 0.189 | 247.2 | 0.151 | 297.2 | 0.184 |  | 347.2 | 0.18 | 397.2 | 0.167 |  |  |  |
| 197.22 | 0.177 | 247.22 | 0.141 | 297.22 | 0.186 |  | 347.22 | 0.164 | 397.22 | 0.175 |  |  |  |
| 197.24 | 0.172 | 247.24 | 0.113 | 297.24 | 0.193 |  | 347.24 | 0.17 | 397.24 | 0.182 |  |  |  |
| 197.26 | 0.188 | 247.26 | 0.125 | 297.26 | 0.207 |  | 347.26 | 0.172 | 397.26 | 0.171 |  |  |  |
| 197.28 | 0.245 | 247.28 | 0.132 | 297.28 | 0.222 |  | 347.28 | 0.209 | 397.28 | 0.143 |  |  |  |
| 197.3 | 0.278 | 247.3 | 0.128 | 297.3 | 0.222 |  | 347.3 | 0.174 | 397.3 | 0.143 |  |  |  |
| 197.32 | 0.251 | 247.32 | 0.131 | 297.32 | 0.219 |  | 347.32 | 0.151 | 397.32 | 0.139 |  |  |  |
| 197.34 | 0.208 | 247.34 | 0.133 | 297.34 | 0.215 |  | 347.34 | 0.104 | 397.34 | 0.143 |  |  |  |
| 197.36 | 0.193 | 247.36 | 0.159 | 297.36 | 0.221 |  | 347.36 | 0.144 | 397.36 | 0.158 |  |  |  |
| 197.38 | 0.193 | 247.38 | 0.154 | 297.38 | 0.222 |  | 347.38 | 0.178 | 397.38 | 0.168 |  |  |  |
| 197.4 | 0.174 | 247.4 | 0.167 | 297.4 | 0.204 |  | 347.4 | 0.172 | 397.4 | 0.165 |  |  |  |
| 197.42 | 0.154 | 247.42 | 0.185 | 297.42 | 0.208 |  | 347.42 | 0.169 | 397.42 | 0.157 |  |  |  |
| 197.44 | 0.129 | 247.44 | 0.171 | 297.44 | 0.227 |  | 347.44 | 0.191 | 397.44 | 0.146 |  |  |  |
| 197.46 | 0.12 | 247.46 | 0.158 | 297.46 | 0.244 |  | 347.46 | 0.212 | 397.46 | 0.146 |  |  |  |
| 197.48 | 0.118 | 247.48 | 0.133 | 297.48 | 0.256 |  | 347.48 | 0.194 | 397.48 | 0.154 |  |  |  |
| 197.5 | 0.107 | 247.5 | 0.136 | 297.5 | 0.248 |  | 347.5 | 0.131 | 397.5 | 0.155 |  |  |  |
| 197.52 | 0.133 | 247.52 | 0.117 | 297.52 | 0.235 |  | 347.52 | 0.17 | 397.52 | 0.161 |  |  |  |
| 197.54 | 0.181 | 247.54 | 0.119 | 297.54 | 0.225 |  | 347.54 | 0.198 | 397.54 | 0.165 |  |  |  |
| 197.56 | 0.153 | 247.56 | 0.128 | 297.56 | 0.224 |  | 347.56 | 0.2 | 397.56 | 0.166 |  |  |  |
| 197.58 | 0.084 | 247.58 | 0.136 | 297.58 | 0.201 |  | 347.58 | 0.195 | 397.58 | 0.178 |  |  |  |
| 197.6 | 0.041 | 247.6 | 0.153 | 297.6 | 0.189 |  | 347.6 | 0.174 | 397.6 | 0.18 |  |  |  |
| 197.62 | 0.065 | 247.62 | 0.163 | 297.62 | 0.188 |  | 347.62 | 0.128 | 397.62 | 0.181 |  |  |  |
| 197.64 | 0.057 | 247.64 | 0.152 | 297.64 | 0.204 |  | 347.64 | 0.186 | 397.64 | 0.181 |  |  |  |
| 197.66 | 0.066 | 247.66 | 0.169 | 297.66 | 0.214 |  | 347.66 | 0.185 | 397.66 | 0.187 |  |  |  |
| 197.68 | 0.089 | 247.68 | 0.217 | 297.68 | 0.214 |  | 347.68 | 0.189 | 397.68 | 0.182 |  |  |  |
| 197.7 | 0.098 | 247.7 | 0.264 | 297.7 | 0.21 |  | 347.7 | 0.195 | 397.7 | 0.177 |  |  |  |
| 197.72 | 0.102 | 247.72 | 0.253 | 297.72 | 0.099 |  | 347.72 | 0.146 | 397.72 | 0.182 |  |  |  |
| 197.74 | 0.099 | 247.74 | 0.224 | 297.74 | 0.109 |  | 347.74 | 0.19 | 397.74 | 0.188 |  |  |  |
| 197.76 | 0.093 | 247.76 | 0.19 | 297.76 | 0.112 |  | 347.76 | 0.154 | 397.76 | 0.174 |  |  |  |
| 197.78 | 0.079 | 247.78 | 0.165 | 297.78 | 0.121 |  | 347.78 | 0.166 | 397.78 | 0.168 |  |  |  |
| 197.8 | 0.081 | 247.8 | 0.162 | 297.8 | 0.13 |  | 347.8 | 0.159 | 397.8 | 0.186 |  |  |  |
| 197.82 | 0.062 | 247.82 | 0.112 | 297.82 | 0.112 |  | 347.82 | 0.155 | 397.82 | 0.181 |  |  |  |
| 197.84 | 0.057 | 247.84 | 0.228 | 297.84 | 0.115 |  | 347.84 | 0.153 | 397.84 | 0.107 |  |  |  |
| 197.86 | 0.065 | 247.86 | 0.195 | 297.86 | 0.121 |  | 347.86 | 0.152 | 397.86 | 0.113 |  |  |  |
| 197.88 | 0.052 | 247.88 | 0.127 | 297.88 | 0.132 |  | 347.88 | 0.154 | 397.88 | 0.136 |  |  |  |
| 197.9 | 0.058 | 247.9 | 0.148 | 297.9 | 0.151 |  | 347.9 | 0.156 | 397.9 | 0.145 |  |  |  |
| 197.92 | 0.056 | 247.92 | 0.146 | 297.92 | 0.176 |  | 347.92 | 0.164 | 397.92 | 0.148 |  |  |  |
| 197.94 | 0.049 | 247.94 | 0.148 | 297.94 | 0.187 |  | 347.94 | 0.165 | 397.94 | 0.149 |  |  |  |
| 197.96 | 0.069 | 247.96 | 0.18 | 297.96 | 0.163 |  | 347.96 | 0.176 | 397.96 | 0.191 |  |  |  |
| 197.98 | 0.077 | 247.98 | 0.224 | 297.98 | 0.153 |  | 347.98 | 0.199 | 397.98 | 0.185 |  |  |  |
| 198 | 0.083 | 248 | 0.24 | 298 | 0.161 |  | 348 | 0.202 | 398 | 0.173 |  |  |  |
| 198.02 | 0.085 | 248.02 | 0.248 | 298.02 | 0.169 |  | 348.02 | 0.163 | 398.02 | 0.164 |  |  |  |
| 198.04 | 0.082 | 248.04 | 0.244 | 298.04 | 0.174 |  | 348.04 | 0.138 | 398.04 | 0.146 |  |  |  |
| 198.06 | 0.071 | 248.06 | 0.238 | 298.06 | 0.18 |  | 348.06 | 0.134 | 398.06 | 0.121 |  |  |  |
| 198.08 | 0.066 | 248.08 | 0.234 | 298.08 | 0.187 |  | 348.08 | 0.2 | 398.08 | 0.1 |  |  |  |
| 198.1 | 0.066 | 248.1 | 0.253 | 298.1 | 0.191 |  | 348.1 | 0.222 | 398.1 | 0.121 |  |  |  |
| 198.12 | 0.059 | 248.12 | 0.233 | 298.12 | 0.195 |  | 348.12 | 0.22 | 398.12 | 0.168 |  |  |  |
| 198.14 | 0.057 | 248.14 | 0.198 | 298.14 | 0.181 |  | 348.14 | 0.214 | 398.14 | 0.175 |  |  |  |
| 198.16 | 0.053 | 248.16 | 0.169 | 298.16 | 0.18 |  | 348.16 | 0.209 | 398.16 | 0.177 |  |  |  |
| 198.18 | 0.048 | 248.18 | 0.147 | 298.18 | 0.185 |  | 348.18 | 0.213 | 398.18 | 0.14 |  |  |  |
| 198.2 | 0.073 | 248.2 | 0.132 | 298.2 | 0.195 |  | 348.2 | 0.225 | 398.2 | 0.144 |  |  |  |
| 198.22 | 0.099 | 248.22 | 0.093 | 298.22 | 0.199 |  | 348.22 | 0.246 | 398.22 | 0.146 |  |  |  |
| 198.24 | 0.137 | 248.24 | 0.159 | 298.24 | 0.21 |  | 348.24 | 0.254 | 398.24 | 0.18 |  |  |  |
| 198.26 | 0.164 | 248.26 | 0.172 | 298.26 | 0.223 |  | 348.26 | 0.228 | 398.26 | 0.198 |  |  |  |
| 198.28 | 0.135 | 248.28 | 0.175 | 298.28 | 0.218 |  | 348.28 | 0.157 | 398.28 | 0.206 |  |  |  |
| 198.3 | 0.093 | 248.3 | 0.175 | 298.3 | 0.217 |  | 348.3 | 0.154 | 398.3 | 0.185 |  |  |  |
| 198.32 | 0.077 | 248.32 | 0.163 | 298.32 | 0.231 |  | 348.32 | 0.118 | 398.32 | 0.191 |  |  |  |
| 198.34 | 0.06 | 248.34 | 0.165 | 298.34 | 0.248 |  | 348.34 | 0.124 | 398.34 | 0.242 |  |  |  |
| 198.36 | 0.056 | 248.36 | 0.163 | 298.36 | 0.271 |  | 348.36 | 0.096 | 398.36 | 0.243 |  |  |  |
| 198.38 | 0.035 | 248.38 | 0.16 | 298.38 | 0.214 |  | 348.38 | 0.1 | 398.38 | 0.214 |  |  |  |
| 198.4 | 0.023 | 248.4 | 0.144 | 298.4 | 0.217 |  | 348.4 | 0.145 | 398.4 | 0.202 |  |  |  |
| 198.42 | 0.029 | 248.42 | 0.13 | 298.42 | 0.217 |  | 348.42 | 0.167 | 398.42 | 0.226 |  |  |  |
| 198.44 | 0.029 | 248.44 | 0.119 | 298.44 | 0.219 |  | 348.44 | 0.152 | 398.44 | 0.22 |  |  |  |
| 198.46 | 0.025 | 248.46 | 0.095 | 298.46 | 0.222 |  | 348.46 | 0.152 | 398.46 | 0.204 |  |  |  |
| 198.48 | 0.02 | 248.48 | 0.092 | 298.48 | 0.23 |  | 348.48 | 0.162 | 398.48 | 0.195 |  |  |  |
| 198.5 | 0.031 | 248.5 | 0.073 | 298.5 | 0.226 |  | 348.5 | 0.171 | 398.5 | 0.178 |  |  |  |
| 198.52 | 0.013 | 248.52 | 0.055 | 298.52 | 0.221 |  | 348.52 | 0.184 | 398.52 | 0.218 |  |  |  |
| 198.54 | 0.026 | 248.54 | 0.03 | 298.54 | 0.229 |  | 348.54 | 0.199 | 398.54 | 0.212 |  |  |  |
| 198.56 | 0.026 | 248.56 | 0.13 | 298.56 | 0.223 |  | 348.56 | 0.182 | 398.56 | 0.211 |  |  |  |
| 198.58 | 0.084 | 248.58 | 0.132 | 298.58 | 0.215 |  | 348.58 | 0.149 | 398.58 | 0.212 |  |  |  |
| 198.6 | 0.1 | 248.6 | 0.174 | 298.6 | 0.214 |  | 348.6 | 0.136 | 398.6 | 0.214 |  |  |  |
| 198.62 | 0.106 | 248.62 | 0.174 | 298.62 | 0.207 |  | 348.62 | 0.206 | 398.62 | 0.216 |  |  |  |
| 198.64 | 0.105 | 248.64 | 0.178 | 298.64 | 0.197 |  | 348.64 | 0.25 | 398.64 | 0.205 |  |  |  |
| 198.66 | 0.095 | 248.66 | 0.18 | 298.66 | 0.008 |  | 348.66 | 0.256 | 398.66 | 0.235 |  |  |  |
| 198.68 | 0.069 | 248.68 | 0.178 | 298.68 | 0.011 |  | 348.68 | 0.241 | 398.68 | 0.246 |  |  |  |
| 198.7 | 0.083 | 248.7 | 0.181 | 298.7 | 0.031 |  | 348.7 | 0.211 | 398.7 | 0.248 |  |  |  |
| 198.72 | 0.085 | 248.72 | 0.181 | 298.72 | 0.052 |  | 348.72 | 0.212 | 398.72 | 0.242 |  |  |  |
| 198.74 | 0.061 | 248.74 | 0.173 | 298.74 | 0.044 |  | 348.74 | 0.255 | 398.74 | 0.235 |  |  |  |
| 198.76 | 0.05 | 248.76 | 0.163 | 298.76 | 0.052 |  | 348.76 | 0.283 | 398.76 | 0.24 |  |  |  |
| 198.78 | 0.055 | 248.78 | 0.163 | 298.78 | 0.048 |  | 348.78 | 0.243 | 398.78 | 0.245 |  |  |  |
| 198.8 | 0.091 | 248.8 | 0.166 | 298.8 | 0.05 |  | 348.8 | 0.145 | 398.8 | 0.236 |  |  |  |
| 198.82 | 0.06 | 248.82 | 0.17 | 298.82 | 0.052 |  | 348.82 | 0.16 | 398.82 | 0.222 |  |  |  |
| 198.84 | 0.047 | 248.84 | 0.166 | 298.84 | 0.063 |  | 348.84 | 0.165 | 398.84 | 0.212 |  |  |  |
| 198.86 | 0.048 | 248.86 | 0.169 | 298.86 | 0.072 |  | 348.86 | 0.193 | 398.86 | 0.199 |  |  |  |
| 198.88 | 0.052 | 248.88 | 0.178 | 298.88 | 0.086 |  | 348.88 | 0.257 | 398.88 | 0.177 |  |  |  |
| 198.9 | 0.058 | 248.9 | 0.178 | 298.9 | 0.09 |  | 348.9 | 0.215 | 398.9 | 0.185 |  |  |  |
| 198.92 | 0.062 | 248.92 | 0.188 | 298.92 | 0.099 |  | 348.92 | 0.184 | 398.92 | 0.206 |  |  |  |
| 198.94 | 0.068 | 248.94 | 0.207 | 298.94 | 0.105 |  | 348.94 | 0.177 | 398.94 | 0.198 |  |  |  |
| 198.96 | 0.072 | 248.96 | 0.215 | 298.96 | 0.107 |  | 348.96 | 0.201 | 398.96 | 0.197 |  |  |  |
| 198.98 | 0.076 | 248.98 | 0.193 | 298.98 | 0.11 |  | 348.98 | 0.217 | 398.98 | 0.197 |  |  |  |
| 199 | 0.073 | 249 | 0.198 | 299 | 0.117 |  | 349 | 0.172 | 399 | 0.209 |  |  |  |
| 199.02 | 0.072 | 249.02 | 0.254 | 299.02 | 0.12 |  | 349.02 | 0.107 | 399.02 | 0.209 |  |  |  |
| 199.04 | 0.07 | 249.04 | 0.339 | 299.04 | 0.126 |  | 349.04 | 0.123 | 399.04 | 0.211 |  |  |  |
| 199.06 | 0.066 | 249.06 | 0.381 | 299.06 | 0.125 |  | 349.06 | 0.175 | 399.06 | 0.214 |  |  |  |
| 199.08 | 0.06 | 249.08 | 0.289 | 299.08 | 0.117 |  | 349.08 | 0.2 | 399.08 | 0.215 |  |  |  |
| 199.1 | 0.054 | 249.1 | 0.212 | 299.1 | 0.12 |  | 349.1 | 0.229 | 399.1 | 0.214 |  |  |  |
| 199.12 | 0.052 | 249.12 | 0.188 | 299.12 | 0.135 |  | 349.12 | 0.238 | 399.12 | 0.218 |  |  |  |
| 199.14 | 0.047 | 249.14 | 0.168 | 299.14 | 0.145 |  | 349.14 | 0.229 | 399.14 | 0.219 |  |  |  |
| 199.16 | 0.04 | 249.16 | 0.163 | 299.16 | 0.166 |  | 349.16 | 0.207 | 399.16 | 0.219 |  |  |  |
| 199.18 | 0.046 | 249.18 | 0.177 | 299.18 | 0.171 |  | 349.18 | 0.19 | 399.18 | 0.227 |  |  |  |
| 199.2 | 0.044 | 249.2 | 0.179 | 299.2 | 0.188 |  | 349.2 | 0.181 | 399.2 | 0.245 |  |  |  |
| 199.22 | 0.038 | 249.22 | 0.175 | 299.22 | 0.214 |  | 349.22 | 0.153 | 399.22 | 0.273 |  |  |  |
| 199.24 | 0.018 | 249.24 | 0.158 | 299.24 | 0.211 |  | 349.24 | 0.16 | 399.24 | 0.306 |  |  |  |
| 199.26 | 0.024 | 249.26 | 0.156 | 299.26 | 0.198 |  | 349.26 | 0.167 | 399.26 | 0.288 |  |  |  |
| 199.28 | 0.039 | 249.28 | 0.162 | 299.28 | 0.205 |  | 349.28 | 0.162 | 399.28 | 0.265 |  |  |  |
| 199.3 | 0.041 | 249.3 | 0.162 | 299.3 | 0.211 |  | 349.3 | 0.162 | 399.3 | 0.298 |  |  |  |
| 199.32 | 0.041 | 249.32 | 0.147 | 299.32 | 0.213 |  | 349.32 | 0.167 | 399.32 | 0.373 |  |  |  |
| 199.34 | 0.042 | 249.34 | 0.125 | 299.34 | 0.205 |  | 349.34 | 0.2 | 399.34 | 0.345 |  |  |  |
| 199.36 | 0.05 | 249.36 | 0.138 | 299.36 | 0.198 |  | 349.36 | 0.2 | 399.36 | 0.236 |  |  |  |
| 199.38 | 0.055 | 249.38 | 0.151 | 299.38 | 0.187 |  | 349.38 | 0.202 | 399.38 | 0.231 |  |  |  |
| 199.4 | 0.057 | 249.4 | 0.179 | 299.4 | 0.185 |  | 349.4 | 0.191 | 399.4 | 0.224 |  |  |  |
| 199.42 | 0.068 | 249.42 | 0.202 | 299.42 | 0.184 |  | 349.42 | 0.185 | 399.42 | 0.22 |  |  |  |
| 199.44 | 0.17 | 249.44 | 0.292 | 299.44 | 0.174 |  | 349.44 | 0.187 | 399.44 | 0.218 |  |  |  |
| 199.46 | 0.207 | 249.46 | 0.2442 | 299.46 | 0.169 |  | 349.46 | 0.189 | 399.46 | 0.218 |  |  |  |
| 199.48 | 0.177 | 249.48 | 0.246 | 299.48 | 0.167 |  | 349.48 | 0.196 | 399.48 | 0.208 |  |  |  |
| 199.5 | 0.136 | 249.5 | 0.327 | 299.5 | 0.161 |  | 349.5 | 0.192 | 399.5 | 0.163 |  |  |  |
| 199.52 | 0.139 | 249.52 | 0.218 | 299.52 | 0.15 |  | 349.52 | 0.19 | 399.52 | 0.19 |  |  |  |
| 199.54 | 0.139 | 249.54 | 0.162 | 299.54 | 0.153 |  | 349.54 | 0.186 | 399.54 | 0.195 |  |  |  |
| 199.56 | 0.148 | 249.56 | 0.168 | 299.56 | 0.145 |  | 349.56 | 0.19 | 399.56 | 0.194 |  |  |  |
| 199.58 | 0.272 | 249.58 | 0.186 | 299.58 | 0.143 |  | 349.58 | 0.168 | 399.58 | 0.196 |  |  |  |
| 199.6 | 0.293 | 249.6 | 0.231 | 299.6 | 0.148 |  | 349.6 | 0.185 | 399.6 | 0.196 |  |  |  |
| 199.62 | 0.206 | 249.62 | 0.231 | 299.62 | 0.188 |  | 349.62 | 0.202 | 399.62 | 0.203 |  |  |  |
| 199.64 | 0.103 | 249.64 | 0.235 | 299.64 | 0.19 |  | 349.64 | 0.214 | 399.64 | 0.213 |  |  |  |
| 199.66 | 0.082 | 249.66 | 0.236 | 299.66 | 0.179 |  | 349.66 | 0.19 | 399.66 | 0.208 |  |  |  |
| 199.68 | 0.086 | 249.68 | 0.233 | 299.68 | 0.165 |  | 349.68 | 0.209 | 399.68 | 0.2 |  |  |  |
| 199.7 | 0.102 | 249.7 | 0.207 | 299.7 | 0.162 |  | 349.7 | 0.207 | 399.7 | 0.199 |  |  |  |
| 199.72 | 0.125 | 249.72 | 0.284 | 299.72 | 0.158 |  | 349.72 | 0.201 | 399.72 | 0.201 |  |  |  |
| 199.74 | 0.188 | 249.74 | 0.153 | 299.74 | 0.151 |  | 349.74 | 0.202 | 399.74 | 0.198 |  |  |  |
| 199.76 | 0.261 | 249.76 | 0.157 | 299.76 | 0.149 |  | 349.76 | 0.191 | 399.76 | 0.167 |  |  |  |
| 199.78 | 0.008 | 249.78 | 0.163 | 299.78 | 0.141 |  | 349.78 | 0.175 | 399.78 | 0.159 |  |  |  |
| 199.8 | -0.002 | 249.8 | 0.16 | 299.8 | 0.141 |  | 349.8 | 0.178 | 399.8 | 0.196 |  |  |  |
| 199.82 | -0.073 | 249.82 | 0.171 | 299.82 | 0.21 |  | 349.82 | 0.188 | 399.82 | 0.193 |  |  |  |
| 199.84 | -0.078 | 249.84 | 0.18 | 299.84 | 0.208 |  | 349.84 | 0.186 | 399.84 | 0.193 |  |  |  |
| 199.86 | -0.073 | 249.86 | 0.188 | 299.86 | 0.187 |  | 349.86 | 0.188 | 399.86 | 0.188 |  |  |  |
| 199.88 | -0.05 | 249.88 | 0.234 | 299.88 | 0.175 |  | 349.88 | 0.198 | 399.88 | 0.183 |  |  |  |
| 199.9 | -0.041 | 249.9 | 0.27 | 299.9 | 0.166 |  | 349.9 | 0.197 | 399.9 | 0.19 |  |  |  |
| 199.92 | -0.017 | 249.92 | 0.384 | 299.92 | 0.159 |  | 349.92 | 0.194 | 399.92 | 0.193 |  |  |  |
| 199.94 | 0 | 249.94 | 0.3828 | 299.94 | 0.157 |  | 349.94 | 0.189 | 399.94 | 0.199 |  |  |  |
| 199.96 | 0.014 | 249.96 | 0.261 | 299.96 | 0.155 |  | 349.96 | 0.174 | 399.96 | 0.205 |  |  |  |
| 199.98 | 0.023 | 249.98 | 0.2484 | 299.98 | 0.152 |  | 349.98 | 0.169 | 399.98 | 0.213 |  |  |  |

**Supplementary Table S3. X-ray fluorescence measurements**

| Depth (m) | Ba | Fe | Depth (m) | Ba | Fe |
| --- | --- | --- | --- | --- | --- |
| 150.1 | 0.048 | 2.002 | 259.5 | 0.055 | 3.718 |
| 150.3 | 0.053 | 1.616 | 259.7 | 0.066 | 4.068 |
| 150.5 | 0.061 | 3.896 | 259.8 | 0.064 | 4.571 |
| 150.9 | 0.053 | 5.59 | 260 | 0.056 | 5.075 |
| 151.4 | 0.059 | 3.445 | 260.5 | 0.062 | 5.226 |
| 151.7 | 0.055 | 3.783 | 260.8 | 0.055 | 4.425 |
| 151.9 | 0.06 | 3.643 | 261.1 | 0.054 | 4.344 |
| 152.1 | 0.067 | 3.157 | 261.4 | 0.073 | 4.539 |
| 152.3 | 0.062 | 3.998 | 261.6 | 0.063 | 4.935 |
| 152.5 | 0.062 | 3.704 | 261.9 | 0.058 | 3.836 |
| 152.7 | 0.064 | 5.58 | 262.3 | 0.059 | 4.449 |
| 152.9 | 0.054 | 4.181 | 262.5 | 0.073 | 4.924 |
| 153.1 | 0.056 | 4.234 | 262.7 | 0.065 | 4.929 |
| 153.4 | 0.061 | 3.683 | 263 | 0.061 | 5.288 |
| 153.6 | 0.051 | 2.77 | 263.2 | 0.045 | 2.991 |
| 153.8 | 0.032 | 2.192 | 263.4 | 0.043 | 3.202 |
| 154 | 0.053 | 3.432 | 263.5 | 0.055 | 2.593 |
| 154.2 | 0.054 | 2.066 | 263.9 | 0.073 | 4.397 |
| 154.4 | 0.033 | 1.737 | 264.2 | 0.069 | 3.536 |
| 154.6 | 0.037 | 3.609 | 264.4 | 0.072 | 4.708 |
| 154.8 | 0.034 | 2.164 | 264.7 | 0.041 | 4.862 |
| 155 | 0.026 | 1.824 | 264.9 | 0.07 | 5.369 |
| 155.2 | 0.025 | 1.275 | 265.2 | 0.071 | 3.12 |
| 155.4 | 0.016 | 0.928 | 265.5 | 0.072 | 5.317 |
| 155.6 | 0.029 | 2.311 | 265.7 | 0.059 | 4.902 |
| 155.8 | 0.039 | 1.879 | 265.9 | 0.06 | 5.891 |
| 156 | 0.033 | 1.628 | 266.4 | 0.072 | 9.349 |
| 156.3 | 0.044 | 2.069 | 266.9 | 0.085 | 4.954 |
| 156.6 | 0.04 | 1.967 | 267.3 | 0.07 | 4.087 |
| 156.8 | 0.019 | 1.045 | 267.7 | 0.054 | 9.132 |
| 157 | 0.022 | 0.819 | 268.3 | 0.07 | 3.617 |
| 157.2 | 0.027 | 1.521 | 269.1 | 0.074 | 6.317 |
| 157.4 | 0.032 | 1.844 | 269.4 | 0.064 | 4.557 |
| 157.6 | 0.031 | 1.887 | 269.7 | 0.071 | 3.996 |
| 157.8 | 0.047 | 7.389 | 270.2 | 0.07 | 6.206 |
| 158.1 | 0.041 | 4.479 | 271.1 | 0.068 | 5.089 |
| 158.3 | 0.037 | 0.562 | 271.9 | 0.049 | 4.256 |
| 158.5 | 0.019 | 3.862 | 272.7 | 0.054 | 4.446 |
| 158.7 | 0.034 | 3.445 | 273.3 | 0.075 | 3.499 |
| 158.9 | 0.032 | 5.744 | 273.9 | 0.06 | 2.447 |
| 159.1 | 0.038 | 4.215 | 274.2 | 0.055 | 2.246 |
| 159.3 | 0.043 | 7.303 | 274.7 | 0.065 | 4.488 |
| 159.5 | 0.037 | 3.914 | 275.3 | 0.055 | 4.535 |
| 159.7 | 0.042 | 4.334 | 275.8 | 0.05 | 5.063 |
| 159.9 | 0.032 | 3.816 | 276.3 | 0.058 | 5.182 |
| 160.2 | 0.018 | 2.16 | 276.7 | 0.077 | 5.045 |
| 160.4 | 0.032 | 3.856 | 277.3 | 0.047 | 4.819 |
| 160.5 | 0.028 | 4.472 | 277.9 | 0.075 | 6.106 |
| 160.8 | 0.034 | 4.284 | 278.7 | 0.059 | 3.291 |
| 161 | 0.02 | 1.733 | 279.5 | 0.059 | 3.234 |
| 161.1 | 0.045 | 3.447 | 280.2 | 0.078 | 5.555 |
| 161.4 |  | 0.689 | 280.5 | 0.061 | 4.267 |
| 161.6 | 0.022 | 1.96 | 281 | 0.06 | 4.095 |
| 161.8 | 0.037 | 3.103 | 281.8 | 0.069 | 1.852 |
| 161.9 | 0.055 | 1.472 | 282.5 | 0.062 | 4.368 |
| 162.2 | 0.051 | 4.129 | 283.2 | 0.061 | 3.849 |
| 162.4 | 0.051 | 3.548 | 283.9 | 0.065 | 2.509 |
| 162.6 | 0.045 | 3.962 | 284.5 | 0.057 | 2.23 |
| 126.8 | 0.035 | 4.092 | 285.2 | 0.061 | 4.016 |
| 163 | 0.044 | 3.821 | 285.85 | 0.068 | 3.155 |
| 163.2 | 0.035 | 4.492 | 286.4 | 0.058 | 2.465 |
| 163.4 | 0.042 | 3.479 | 287 | 0.056 | 4.513 |
| 163.6 | 0.048 | 3.794 | 287.55 | 0.064 | 3.013 |
| 163.8 | 0.033 | 3.657 | 288.15 | 0.062 | 3.519 |
| 164 | 0.042 | 3.598 | 288.6 | 0.063 | 3.525 |
| 164.2 | 0.043 | 6.212 | 289.3 | 0.068 | 1.798 |
| 164.4 | 0.029 | 4.092 | 290 | 0.06 | 5.21 |
| 164.6 | 0.033 | 3.378 | 290.8 | 0.075 | 5.108 |
| 164.8 | 0.032 | 3.897 | 291.1 | 0.058 | 4.481 |
| 165 | 0.042 | 4.487 | 291.4 | 0.068 | 5.539 |
| 165.2 | 0.028 | 3.192 | 291.8 | 0.066 | 5.234 |
| 165.4 | 0.036 | 10.083 | 292.15 | 0.062 | 2.973 |
| 165.6 | 0.038 | 3.694 | 292.55 | 0.053 | 2.698 |
| 165.9 | 0.044 | 5.143 | 292.85 | 0.044 | 3.869 |
| 166.1 | 0.039 | 4.868 | 293.9 | 0.047 | 3.477 |
| 166.3 | 0.031 | 3.91 | 294.6 | 0.049 | 3.545 |
| 166.5 | 0.035 | 4.293 | 295.05 | 0.052 | 4.883 |
| 166.7 | 0.033 | 2.51 | 295.4 | 0.045 | 2.879 |
| 167.2 | 0.025 | 3.479 | 296.2 | 0.055 | 3.812 |
| 167.4 | 0.034 | 3.339 | 296.6 | 0.045 | 2.292 |
| 167.6 | 0.038 | 3.367 | 297.25 | 0.065 | 8.281 |
| 167.8 | 0.018 | 1.552 | 297.65 | 0.063 | 6.59 |
| 168.2 | 0.021 | 4.376 | 298 | 0.06 | 4.972 |
| 168.4 | 0.029 | 4.889 | 298.7 | 0.061 | 4.977 |
| 168.6 | 0.016 | 2.866 | 299.05 | 0.059 | 6.219 |
| 168.8 | 0.032 | 3.241 | 299.5 | 0.066 | 5.2 |
| 168.9 | 0.03 | 3.416 | 299.9 | 0.059 | 4.467 |
| 169.1 | 0.054 | 7.022 | 300.45 | 0.058 | 5.228 |
| 169.4 | 0.023 | 2.682 | 300.95 | 0.048 | 3.624 |
| 169.6 | 0.036 | 3.089 | 301.4 | 0.042 | 3.579 |
| 169.8 | 0.036 | 3.163 | 301.85 | 0.066 | 4.642 |
| 169.9 | 0.034 | 3.978 | 302.5 | 0.052 | 2.5 |
| 170.3 | 0.038 | 2.665 | 303.25 | 0.049 | 2.664 |
| 170.4 | 0.032 | 4.526 | 303.75 | 0.058 | 4.299 |
| 170.6 | 0.034 | 4.786 | 304.7 | 0.06 | 5.379 |
| 170.8 | 0.032 | 2.635 | 305.1 | 0.055 | 5.145 |
| 170.9 | 0.031 | 3.588 | 305.6 | 0.056 | 5.449 |
| 171.1 | 0.029 | 3.614 | 306.1 | 0.063 | 8.078 |
| 171.3 | 0.026 | 10.835 | 306.55 | 0.055 | 5.295 |
| 171.5 | 0.034 | 17.049 | 307.05 | 0.054 | 5.495 |
| 171.7 | 0.034 | 2.184 | 307.4 | 0.058 | 5.251 |
| 171.9 | 0.034 | 2.515 | 307.55 | 0.049 | 4.953 |
| 172.2 | 0.034 | 4.27 | 307.8 | 0.045 | 3.519 |
| 172.3 | 0.027 | 2.319 | 307.95 | 0.053 | 4.149 |
| 172.4 | 0.032 | 2.838 | 308.1 | 0.052 | 5.051 |
| 172.5 | 0.033 | 5.456 | 308.3 | 0.058 | 4.945 |
| 172.7 | 0.022 | 3.175 | 308.5 | 0.051 | 5.825 |
| 173 | 0.027 | 4.029 | 308.75 | 0.055 | 4.302 |
| 173.2 | 0.036 | 3.37 | 308.95 | 0.058 | 4.557 |
| 173.4 | 0.033 | 3.512 | 309.15 | 0.057 | 4.445 |
| 173.6 | 0.03 | 3.572 | 309.35 | 0.054 | 5.024 |
| 173.8 | 0.028 | 3.094 | 309.55 | 0.053 | 5.662 |
| 174.1 | 0.026 | 2.386 | 309.75 | 0.058 | 5.288 |
| 174.3 | 0.024 | 2.235 | 309.95 | 0.044 | 4.438 |
| 174.5 | 0.033 | 2.87 | 310.1 | 0.05 | 4.491 |
| 174.7 | 0.027 | 2.494 | 310.35 | 0.045 | 4.556 |
| 174.8 | 0.033 | 4.425 | 310.4 | 0.047 | 4.335 |
| 174.9 | 0.016 | 2.015 | 310.55 | 0.055 | 5.137 |
| 175 | 0.017 | 1.669 | 310.75 | 0.054 | 4.88 |
| 175.1 | 0.027 | 3.033 | 310.95 | 0.051 | 4.657 |
| 175.3 | 0.013 | 1.806 | 311.1 | 0.048 | 3.969 |
| 175.5 | 0.013 | 1.698 | 311.25 | 0.054 | 5.18 |
| 175.7 | 0.017 | 1.875 | 311.4 | 0.055 | 4.714 |
| 175.9 | 0.025 | 1.405 | 311.75 | 0.045 | 2.781 |
| 176.2 | 0.015 | 1.035 | 311.9 | 0.048 | 4.488 |
| 176.5 | 0.016 | 1.714 | 312.05 | 0.052 | 5.277 |
| 176.7 | 0.021 | 1.349 | 312.2 | 0.055 | 5.119 |
| 177 |  | 2.532 | 312.35 | 0.058 | 5.563 |
| 177.2 |  | 2.126 | 312.5 | 0.056 | 5.195 |
| 177.4 | 0.02 | 2.062 | 312.7 | 0.056 | 5.224 |
| 177.6 | 0.034 | 3.726 | 313 | 0.052 | 5.412 |
| 177.8 | 0.032 | 2.842 | 313.3 | 0.052 | 5.299 |
| 178 | 0.027 | 2.613 | 313.55 | 0.063 | 5.396 |
| 178.2 | 0.033 | 3.282 | 313.8 | 0.044 | 4.178 |
| 178.4 | 0.034 | 4.467 | 313.95 | 0.064 | 5.624 |
| 178.6 | 0.04 | 8.454 | 314.2 | 0.05 | 5.161 |
| 178.8 | 0.035 | 4.026 | 314.45 | 0.05 | 5.101 |
| 179 | 0.032 | 5.077 | 314.65 | 0.049 | 5.196 |
| 179.2 | 0.031 | 3.876 | 314.9 | 0.057 | 5.321 |
| 179.3 | 0.033 | 5.297 | 315.1 | 0.054 | 5.399 |
| 179.5 | 0.03 | 4.144 | 315.3 | 0.051 | 5.33 |
| 179.7 | 0.027 | 3.515 | 315.5 | 0.058 | 5.26 |
| 179.9 | 0.039 | 3.811 | 315.75 | 0.057 | 4.917 |
| 180.1 | 0.027 | 4.38 | 316.05 | 0.237 | 2.55 |
| 180.3 | 0.035 | 3.931 | 316.7 | 0.046 | 2.041 |
| 180.5 | 0.042 | 24.09 | 316.95 | 0.052 | 4.431 |
| 180.7 | 0.031 | 3.005 | 317.1 | 0.059 | 4.665 |
| 180.9 | 0.027 | 3.772 | 317.2 | 0.058 | 5.635 |
| 181.1 | 0.033 | 3.841 | 317.3 | 0.05 | 5.148 |
| 181.4 | 0.028 | 4.394 | 317.45 | 0.059 | 5.046 |
| 181.7 | 0.036 | 3.943 | 317.55 | 0.056 | 5.03 |
| 182 | 0.039 | 4.001 | 317.7 | 0.056 | 4.998 |
| 182.2 | 0.035 | 4.228 | 317.85 | 0.046 | 5.143 |
| 182.6 | 0.024 | 2.679 | 317.95 | 0.063 | 6.019 |
| 183 | 0.033 | 3.573 | 318.1 | 0.058 | 5.598 |
| 183.2 | 0.022 | 3.071 | 318.4 | 0.063 | 5.52 |
| 183.9 | 0.019 | 2.531 | 318.6 | 0.058 | 5.63 |
| 184.4 | 0.043 | 5.605 | 318.8 | 0.056 | 6.233 |
| 184.6 | 0.036 | 5.139 | 319.05 | 0.063 | 5.979 |
| 184.8 | 0.033 | 5.782 | 319.2 | 0.055 | 5.278 |
| 185 | 0.032 | 3.653 | 319.5 | 0.053 | 5.272 |
| 185.2 | 0.027 | 6.376 | 319.7 | 0.062 | 5.979 |
| 185.4 | 0.026 | 3.561 | 319.9 | 0.056 | 5.143 |
| 185.6 | 0.029 | 3.784 | 320.1 | 0.045 | 4.876 |
| 158.8 | 0.031 | 3.897 | 320.3 | 0.061 | 5.596 |
| 186.1 | 0.023 | 3.433 | 320.5 | 0.054 | 6.197 |
| 186.3 | 0.033 | 3.479 | 320.7 | 0.045 | 4.986 |
| 186.7 | 0.032 | 3.825 | 320.9 | 0.055 | 5.51 |
| 186.9 | 0.028 | 3.331 | 321.1 | 0.055 | 4.888 |
| 187.2 | 0.033 | 3.666 | 321.3 | 0.057 | 5.57 |
| 187.5 | 0.035 | 3.606 | 321.5 | 0.051 | 6.385 |
| 187.7 | 0.017 | 3.611 | 321.65 | 0.045 | 6.32 |
| 187.9 | 0.019 | 2.021 | 321.8 | 0.055 | 6.244 |
| 188.1 | 0.042 | 19.251 | 321.95 | 0.044 | 5.863 |
| 188.3 | 0.026 | 3.086 | 322.05 | 0.036 | 2.358 |
| 188.6 | 0.023 | 2.812 | 322.15 | 0.061 | 5.632 |
| 188.8 | 0.045 | 10.155 | 322.3 | 0.065 | 6.446 |
| 189.1 | 0.015 | 2.514 | 322.45 | 0.061 | 5.316 |
| 189.3 | 0.022 | 3.361 | 322.55 | 0.067 | 5.872 |
| 189.6 | 0.024 | 3.412 | 322.7 | 0.067 | 6.85 |
| 189.8 | 0.03 | 4.516 | 322.8 | 0.068 | 6.435 |
| 190.1 | 0.033 | 4.121 | 322.95 | 0.055 | 6.788 |
| 190.3 | 0.028 | 12.155 | 323.05 | 0.05 | 6.56 |
| 190.6 | 0.02 | 2.568 | 323.25 | 0.056 | 6.076 |
| 191.1 | 0.023 | 3.056 | 323.45 | 0.052 | 5.235 |
| 191.3 | 0.031 | 2.611 | 323.6 | 0.039 | 3.517 |
| 191.6 | 0.027 | 3.287 | 323.8 | 0.046 | 4.474 |
| 191.8 | 0.026 | 3.102 | 324 | 0.056 | 5.173 |
| 192.1 | 0.032 | 3.569 | 324.1 | 0.051 | 5.246 |
| 192.3 | 0.036 | 6.199 | 324.3 | 0.044 | 5.566 |
| 192.6 | 0.025 | 3.569 | 324.45 | 0.046 | 5.835 |
| 192.9 | 0.033 | 3.925 | 324.65 | 0.061 | 6.318 |
| 193.3 | 0.032 | 3.709 | 324.8 | 0.059 | 5.483 |
| 193.5 | 0.027 | 2.745 | 324.9 | 0.095 | 7.414 |
| 193.8 | 0.03 | 6.157 | 325 | 0.071 | 5.505 |
| 194.2 | 0.034 | 4.476 | 325.15 | 0.083 | 4.899 |
| 194.5 | 0.032 | 4.526 | 325.25 | 0.028 | 8.026 |
| 194.7 | 0.017 | 3.233 | 325.35 | 0.074 | 4.797 |
| 195.1 | 0.031 | 3.559 | 325.5 | 0.059 | 3.797 |
| 195.3 | 0.026 | 3.808 | 325.6 | 0.059 | 3.126 |
| 195.6 | 0.025 | 3.478 | 325.85 | 0.058 | 2.49 |
| 195.8 | 0.03 | 4.424 | 325.95 | 0.067 | 3.23 |
| 196 | 0.03 | 3.396 | 326.2 | 0.072 | 1.62 |
| 196.3 | 0.021 | 5.87 | 326.6 | 0.085 | 4.722 |
| 196.5 | 0.023 | 3.641 | 326.8 | 0.074 | 4.874 |
| 196.7 | 0.034 | 4.499 | 327.05 | 0.074 | 5.225 |
| 196.9 | 0.035 | 4.241 | 327.15 | 0.057 | 3.406 |
| 197.1 | 0.038 | 6.788 | 327.4 | 0.058 | 3.717 |
| 197.4 | 0.034 | 4.89 | 327.6 | 0.039 | 5.22 |
| 197.6 | 0.016 | 2.689 | 327.8 | 0.043 | 4.074 |
| 197.9 | 0.021 | 2.425 | 328.05 | 0.021 | 3.46 |
| 198.1 | 0.024 | 1.929 | 328.3 | 0.064 | 4.886 |
| 198.3 | 0.02 | 2.142 | 328.5 | 0.024 | 3.014 |
| 198.5 | 0.025 | 1.406 | 328.75 | 0.023 | 4.03 |
| 198.7 | 0.023 | 2.598 | 329.05 | 0.021 | 2.822 |
| 198.9 | 0.024 | 2.017 | 329.3 | 0.037 | 2.79 |
| 199.1 | 0.022 | 2.177 | 329.5 | 0.041 | 3.481 |
| 199.3 | 0.02 | 1.915 | 329.7 | 0.048 | 4.815 |
| 199.5 | 0.023 | 1.765 | 329.85 | 0.055 | 5.027 |
| 199.6 | 0.026 | 2.474 | 330.1 | 0.055 | 5.533 |
| 200.1 | 0.04 | 12.69 | 330.2 | 0.051 | 4.972 |
| 200.3 | 0.027 | 4.018 | 330.45 | 0.058 | 4.545 |
| 200.4 | 0.024 | 3.385 | 330.55 | 0.049 | 3.372 |
| 200.5 | 0.022 | 4.573 | 330.8 | 0.043 | 5.03 |
| 200.6 | 0.04 | 6.424 | 331 | 0.033 | 4.943 |
| 200.9 | 0.024 | 3.448 | 331.3 | 0.038 | 4.338 |
| 201.1 | 0.027 | 2.543 | 331.5 | 0.033 | 3.856 |
| 201.3 | 0.031 | 4.027 | 331.65 | 0.039 | 4.536 |
| 201.5 | 0.022 | 4.307 | 331.8 | 0.041 | 4.499 |
| 201.7 | 0.023 | 3.218 | 332 | 0.043 | 5.039 |
| 201.9 | 0.032 | 4.601 | 332.2 | 0.034 | 4.826 |
| 202 | 0.035 | 6.367 | 332.4 | 0.053 | 5.803 |
| 202.2 | 0.031 | 4.036 | 332.6 | 0.051 | 5.914 |
| 202.4 | 0.027 | 5.226 | 332.8 | 0.048 | 5.049 |
| 202.6 | 0.035 | 3.598 | 333.2 | 0.048 | 4.843 |
| 202.7 | 0.03 | 3.748 | 333.25 | 0.058 | 3.856 |
| 202.9 | 0.036 | 8.303 | 333.35 | 0.067 | 5.388 |
| 203.1 | 0.036 | 3.197 | 333.5 | 0.046 | 2.019 |
| 203.3 | 0.022 | 3.01 | 333.65 | 0.066 | 6.005 |
| 203.7 | 0.03 | 3.669 | 333.85 | 0.072 | 3.349 |
| 204 | 0.034 | 2.255 | 334 | 0.058 | 5.74 |
| 204.2 | 0.026 | 3.122 | 334.15 | 0.057 | 5.149 |
| 204.5 | 0.034 | 3.664 | 334.35 | 0.059 | 5.296 |
| 204.8 | 0.032 | 5.666 | 334.5 | 0.066 | 5.731 |
| 205 | 0.03 | 3.753 | 334.75 | 0.069 | 5.348 |
| 205.2 | 0.031 | 3.381 | 334.95 | 0.066 | 5.445 |
| 205.4 | 0.03 | 3.341 | 335.2 | 0.063 | 7.867 |
| 205.8 | 0.032 | 3.023 | 335.3 | 0.065 | 5.363 |
| 206 | 0.026 | 4.094 | 335.5 | 0.074 | 5.377 |
| 206.1 | 0.035 | 2.645 | 335.6 | 0.07 | 5.694 |
| 206.3 | 0.028 | 2.868 | 335.8 | 0.064 | 5.743 |
| 206.5 | 0.037 | 16.724 | 336.1 | 0.07 | 6.681 |
| 206.7 | 0.033 | 3.227 | 336.35 | 0.069 | 6.076 |
| 206.9 | 0.028 | 2.293 | 336.6 | 0.058 | 5.2 |
| 207.1 | 0.025 | 3.018 | 336.85 | 0.056 | 5.314 |
| 207.2 | 0.015 | 3.312 | 337.05 | 0.06 | 5.505 |
| 207.3 | 0.033 | 3.587 | 337.25 | 0.065 | 5.911 |
| 207.5 | 0.034 | 6.31 | 337.5 | 0.062 | 5.229 |
| 207.7 | 0.024 | 3.626 | 337.7 | 0.069 | 4.903 |
| 207.9 | 0.034 | 3.819 | 337.9 | 0.071 | 5.365 |
| 208.1 | 0.037 | 4.009 | 338.1 | 0.07 | 5.443 |
| 208.3 | 0.03 | 4.26 | 338.25 | 0.066 | 6.157 |
| 208.5 | 0.025 | 3.81 | 338.8 | 0.067 | 5.248 |
| 208.7 | 0.031 | 3.272 | 339 | 0.048 | 4.129 |
| 208.9 | 0.024 | 5.67 | 339.1 | 0.05 | 4.335 |
| 209.1 | 0.033 | 4.26 | 339.3 | 0.05 | 4.737 |
| 209.4 | 0.031 | 3.933 | 339.4 | 0.051 | 4.467 |
| 209.6 | 0.028 | 3.694 | 339.5 | 0.061 | 4.803 |
| 209.8 | 0.035 | 3.761 | 339.55 | 0.055 | 5.013 |
| 210.1 | 0.038 | 4.642 | 339.75 | 0.045 | 2.644 |
| 210.3 | 0.033 | 6.394 | 340 | 0.06 | 4.669 |
| 210.4 | 0.028 | 1.666 | 340.25 | 0.065 | 5.317 |
| 210.7 | 0.029 | 1.913 | 340.5 | 0.054 | 4.513 |
| 210.8 | 0.019 | 3.045 | 340.65 | 0.048 | 4.225 |
| 211.1 | 0.018 | 1.915 | 340.8 | 0.063 | 5.161 |
| 211.3 | 0.024 | 9.529 | 341.05 | 0.055 | 4.833 |
| 211.4 | 0.026 | 1.912 | 341.2 | 0.054 | 5.213 |
| 211.6 | 0.02 | 1.685 | 341.35 | 0.055 | 5.294 |
| 211.8 | 0.021 | 1.991 | 341.6 | 0.057 | 4.744 |
| 212.1 | 0.029 | 1.472 | 341.65 | 0.057 | 4.456 |
| 212.3 | 0.023 | 1.334 | 341.8 | 0.033 | 5.385 |
| 212.5 | 0.02 | 1.859 | 342.1 | 0.029 | 3.709 |
| 212.7 | 0.016 | 1.757 | 342.55 | 0.034 | 3.539 |
| 212.8 | 0.011 | 1.876 | 342.8 | 0.031 | 3.905 |
| 212.9 | 0.02 | 1.589 | 343 | 0.051 | 4.901 |
| 213.1 | 0.02 | 0.88 | 343.2 | 0.047 | 4.961 |
| 231.3 | 0.02 | 1.162 | 343.4 | 0.049 | 4.83 |
| 213.4 | 0.013 | 1.151 | 343.6 | 0.043 | 6.398 |
| 213.7 | 0.036 | 3.97 | 343.75 | 0.057 | 6.31 |
| 213.8 | 0.022 | 1.089 | 343.95 | 0.047 | 4.601 |
| 214 | 0.027 | 3.7 | 334.15 | 0.05 | 3.614 |
| 214.2 | 0.029 | 3.4 | 344.25 | 0.053 | 4.348 |
| 214.4 | 0.036 | 4.443 | 344.5 | 0.056 | 4.843 |
| 214.6 | 0.038 | 5.119 | 344.65 | 0.058 | 5.322 |
| 214.8 | 0.037 | 4.384 | 344.8 | 0.043 | 4.957 |
| 215 | 0.029 | 5.361 | 345.1 | 0.045 | 4.188 |
| 215.2 | 0.029 | 3.267 | 345.35 | 0.058 | 4.61 |
| 215.4 | 0.036 | 4.456 | 345.5 | 0.053 | 5.162 |
| 215.5 | 0.036 | 3.093 | 345.75 | 0.066 | 5.675 |
| 215.6 | 0.042 | 2.993 | 346.3 | 0.05 | 4.107 |
| 215.7 | 0.026 | 5.009 | 346.5 | 0.043 | 4.056 |
| 216 | 0.023 | 1.476 | 346.8 | 0.049 | 6.722 |
| 216.2 | 0.03 | 2.08 | 347.2 | 0.036 | 3.958 |
| 216.4 | 0.022 | 7.959 | 347.4 | 0.044 | 2.62 |
| 216.6 | 0.024 | 3.988 | 347.75 | 0.041 | 3.433 |
| 216.8 | 0.041 | 1.833 | 348.05 | 0.061 | 4.25 |
| 217 | 0.035 | 1.679 | 348.5 | 0.066 | 5.024 |
| 217.2 | 0.039 | 1.587 | 349 | 0.062 | 4.86 |
| 217.4 | 0.022 | 1.064 | 349.35 | 0.043 | 3.766 |
| 217.6 | 0.029 | 1.159 | 349.5 | 0.056 | 4.483 |
| 217.8 | 0.026 | 1.93 | 349.65 | 0.041 | 3.434 |
| 217.9 | 0.032 | 2.462 | 349.8 | 0.048 | 4.535 |
| 218.1 | 0.036 | 2.536 | 349.9 | 0.048 | 4.093 |
| 218.3 | 0.03 | 2.545 | 350.1 | 0.039 | 4.609 |
| 218.6 | 0.033 | 1.576 | 350.6 | 0.044 | 4.611 |
| 218.8 | 0.03 | 2.46 | 350.75 | 0.06 | 4.735 |
| 218.9 | 0.025 | 2.478 | 350.9 | 0.047 | 4.408 |
| 219.2 | 0.025 | 1.607 | 351.45 | 0.03 | 2.207 |
| 219.4 | 0.032 | 1.623 | 351.55 | 0.046 | 4.877 |
| 219.6 | 0.026 | 3.504 | 351.7 | 0.037 | 3.22 |
| 219.8 | 0.028 | 2.34 | 351.95 | 0.042 | 3.114 |
| 219.9 | 0.029 | 1.712 | 352.1 | 0.054 | 4.837 |
| 220.1 | 0.018 | 1.665 | 352.35 | 0.059 | 4.8 |
| 220.3 | 0.032 | 1.46 | 352.45 | 0.054 | 4.937 |
| 220.5 | 0.015 | 1.271 | 352.6 | 0.056 | 4.663 |
| 220.7 | 0.013 | 1.025 | 352.7 | 0.051 | 4.965 |
| 220.9 |  | 0.868 | 352.85 | 0.056 | 5.227 |
| 221.1 |  | 1.063 | 353 | 0.051 | 5.19 |
| 221.3 | 0.062 | 8.243 | 353.1 | 0.046 | 5.125 |
| 221.5 | 0.056 | 3.913 | 353.25 | 0.054 | 5.021 |
| 221.7 | 0.043 | 6.061 | 353.4 | 0.054 | 5.006 |
| 221.9 | 0.034 | 10.899 | 353.5 | 0.042 | 4.496 |
| 222.2 | 0.032 | 8.958 | 353.7 | 0.048 | 4.776 |
| 222.4 | 0.025 | 8.097 | 353.9 | 0.054 | 5.334 |
| 222.5 | 0.025 | 3.885 | 354.1 | 0.055 | 5.039 |
| 222.6 | 0.022 | 3.539 | 354.3 | 0.071 | 4.186 |
| 222.8 | 0.026 | 5.086 | 354.6 | 0.055 | 4.982 |
| 223.1 | 0.026 | 2.162 | 354.8 | 0.052 | 3.866 |
| 223.3 | 0.036 | 8.939 | 355 | 0.055 | 4.891 |
| 223.5 | 0.024 | 9.108 | 355.1 | 0.054 | 4.726 |
| 223.8 | 0.032 | 8.622 | 355.3 | 0.068 | 5.341 |
| 224 |  | 10.043 | 355.5 | 0.056 | 4.889 |
| 224.2 | 0.028 | 11.285 | 355.7 | 0.061 | 5.17 |
| 224.5 | 0.032 | 14.367 | 356 | 0.048 | 4.451 |
| 224.7 | 0.047 | 14.855 | 356.25 | 0.062 | 5.979 |
| 225 | 0.088 | 0.421 | 356.5 | 0.062 | 4.436 |
| 225.2 | 0.025 | 14.792 | 356.75 | 0.049 | 2.925 |
| 225.4 | 0.03 | 3.093 | 357.05 | 0.05 | 3.248 |
| 225.7 | 0.036 | 11.035 | 357.4 | 0.06 | 4.488 |
| 225.9 | 0.027 | 11.77 | 357.65 | 0.056 | 4.822 |
| 226.1 | 0.039 | 9.963 | 357.75 | 0.057 | 3.595 |
| 226.3 | 0.041 | 13.469 | 357.85 | 0.073 | 4.906 |
| 226.5 | 0.016 | 0.301 | 358 | 0.072 | 8.773 |
| 226.7 | 0.049 | 3.336 | 358.15 | 0.068 | 5.042 |
| 226.9 | 0.022 | 4.63 | 358.2 | 0.049 | 2.998 |
| 227.1 | 0.017 | 4.864 | 358.3 | 0.062 | 4.675 |
| 227.3 | 0.027 | 9.483 | 358.5 | 0.065 | 6.044 |
| 227.5 | 0.049 | 1.333 | 358.7 | 0.055 | 3.442 |
| 227.7 | 0.03 | 9.573 | 358 | 0.063 | 4.843 |
| 227.8 | 0.031 | 11.593 | 359 | 0.039 | 2.793 |
| 228.1 | 0.037 | 10.004 | 359.2 | 0.043 | 2.614 |
| 228.3 | 0.025 | 9.672 | 359.4 | 0.063 | 5.17 |
| 228.5 | 0.032 | 4.644 | 360.05 | 0.052 | 4.298 |
| 228.7 | 0.026 | 10.075 | 360.3 | 0.066 | 5.39 |
| 228.9 | 0.033 | 6.939 | 361.25 | 0.06 | 4.74 |
| 229.1 | 0.036 | 6.91 | 361.7 | 0.045 | 5.084 |
| 229.3 | 0.051 | 5.874 | 362.3 | 0.04 | 3.937 |
| 229.4 | 0.042 | 5.865 | 362.6 | 0.066 | 4.652 |
| 229.5 | 0.036 | 6.189 | 362.75 | 0.052 | 5.23 |
| 229.6 | 0.041 | 9.579 | 362.85 | 0.055 | 4.558 |
| 229.7 | 0.04 | 4.243 | 363 | 0.064 | 4.559 |
| 229.8 | 0.044 | 2.401 | 363.25 | 0.049 | 3.351 |
| 229.9 | 0.027 | 5.59 | 363.5 | 0.06 | 4.404 |
| 230.1 | 0.043 | 11.02 | 363.7 | 0.053 | 3.56 |
| 230.2 | 0.035 | 6.014 | 363.8 | 0.057 | 4.855 |
| 230.4 | 0.043 | 2.661 | 364 | 0.045 | 9.993 |
| 230.6 | 0.034 | 7.609 | 364.35 | 0.063 | 4.958 |
| 230.7 | 0.031 | 8.995 | 364.6 | 0.06 | 3.943 |
| 230.8 | 0.055 | 0.725 | 364.8 | 0.06 | 4.722 |
| 230.9 | 0.05 | 3.621 | 365 | 0.044 | 2.438 |
| 231.1 | 0.051 | 6.671 | 365.1 | 0.056 | 3.767 |
| 231.2 | 0.023 | 10.518 | 365.5 | 0.076 | 5.256 |
| 231.4 | 0.034 | 1.782 | 366 | 0.064 | 4.519 |
| 231.8 | 0.064 | 3.05 | 366.7 | 0.043 | 3.127 |
| 232 | 0.031 | 7.335 | 367 | 0.067 | 3.829 |
| 232.3 | 0.05 | 9.469 | 367.45 | 0.071 | 5.17 |
| 232.7 | 0.064 | 2.807 | 368 | 0.036 | 3.058 |
| 233 | 0.058 | 6.327 | 368.2 | 0.054 | 4.632 |
| 233.3 | 0.034 | 10.004 | 368.35 | 0.075 | 4.961 |
| 233.6 | 0.064 | 2.873 | 368.5 | 0.063 | 4.926 |
| 234 | 0.064 | 2.615 | 368.85 | 0.079 | 4.322 |
| 234.5 | 0.072 | 3.432 | 369.1 | 0.063 | 3.282 |
| 234.7 | 0.032 | 7.488 | 369.45 | 0.066 | 3.971 |
| 234.9 | 0.044 | 5.385 | 369.8 | 0.074 | 5.041 |
| 235.1 | 0.071 | 2.612 | 370.25 | 0.064 | 4.537 |
| 235.4 | 0.042 | 8.372 | 370.75 | 0.061 | 5.324 |
| 235.6 | 0.051 | 1.341 | 371.15 | 0.075 | 5.243 |
| 235.7 | 0.034 | 6.801 | 371.45 | 0.05 | 4.875 |
| 235.8 | 0.055 | 6.853 | 371.65 | 0.076 | 4.503 |
| 235.9 | 0.045 | 9.308 | 372 | 0.052 | 3.143 |
| 236.2 | 0.053 | 2.68 | 372.3 | 0.066 | 5.081 |
| 236.4 | 0.031 | 8.755 | 372.5 | 0.077 | 4.854 |
| 236.6 | 0.066 | 4.177 | 373.9 | 0.044 | 3.419 |
| 236.9 | 0.282 | 9.338 | 373.25 | 0.062 | 5.013 |
| 237.1 | 0.051 | 7.177 | 373.65 | 0.06 | 4.083 |
| 237.3 | 0.031 | 9.737 | 373.8 | 0.046 | 3.861 |
| 237.5 | 0.057 | 7.807 | 374.8 | 0.042 | 4.91 |
| 237.7 | 0.067 | 2.712 | 375.55 | 0.043 | 1.688 |
| 237.9 | 0.071 | 2.642 | 375.9 | 0.034 | 3.552 |
| 238.1 | 0.057 | 3.047 | 376.25 | 0.044 | 2.906 |
| 238.3 | 0.067 | 3.035 | 376.75 | 0.047 | 3.58 |
| 238.5 | 0.086 | 3.272 | 377.05 | 0.03 | 2.868 |
| 238.7 | 0.039 | 7.461 | 377.4 | 0.044 | 2.159 |
| 238.9 | 0.026 | 7.463 | 377.8 | 0.065 | 3.272 |
| 239.1 | 0.065 | 2.264 | 378.1 | 0.074 | 4.948 |
| 239.3 | 0.058 | 3.608 | 378.3 | 0.069 | 3.749 |
| 239.6 | 0.083 | 3.613 | 378.5 | 0.08 | 5.031 |
| 239.8 | 0.05 | 2.775 | 378.65 | 0.065 | 4.669 |
| 240.2 | 0.058 | 3.456 | 378.95 | 0.073 | 4.707 |
| 240.5 | 0.078 | 3.209 | 379.6 | 0.074 | 4.893 |
| 240.8 | 0.032 | 1.87 | 380 | 0.069 | 4.741 |
| 241.2 | 0.062 | 0.427 | 380.45 | 0.064 | 3.722 |
| 241.5 | 0.076 | 0.752 | 380.5 | 0.064 | 3.709 |
| 241.7 | 0.039 | 1.282 | 381.15 | 0.075 | 4.359 |
| 241.9 | 0.017 | 0.324 | 381.6 | 0.072 | 4.481 |
| 242.2 | 0.076 | 0.999 | 382.1 | 0.06 | 4.251 |
| 242.4 | 0.029 | 0.543 | 382.5 | 0.058 | 2.913 |
| 242.6 | 0.034 | 1.279 | 382.7 | 0.068 | 4.457 |
| 242.8 | 0.122 | 2.455 | 382.9 | 0.065 | 4.361 |
| 243 | 0.035 | 6.86 | 383.15 | 0.061 | 5.628 |
| 243.3 | 0.025 | 1.491 | 383.35 | 0.058 | 3.292 |
| 243.5 | 0.05 | 8.491 | 383.6 | 0.075 | 5.94 |
| 243.7 | 0.051 | 6.627 | 384.1 | 0.068 | 5.55 |
| 243.8 | 0.048 | 4.743 | 384.35 | 0.069 | 5.433 |
| 244 | 0.042 | 3.009 | 834.75 | 0.066 | 5.447 |
| 244.2 | 0.035 | 1.919 | 385.1 | 0.051 | 3.28 |
| 244.3 | 0.035 | 2.645 | 385.15 | 0.067 | 4.17 |
| 244.4 | 0.03 | 2.78 | 385.25 | 0.068 | 4.912 |
| 244.5 | 0.03 | 3.702 | 385.5 | 0.073 | 4.926 |
| 244.7 | 0.041 | 3.691 | 385.75 | 0.069 | 4.859 |
| 244.8 | 0.052 | 3.899 | 386 | 0.059 | 5.079 |
| 245.1 | 0.047 | 3.994 | 386.3 | 0.062 | 5.035 |
| 245.6 | 0.027 | 4.577 | 386.5 | 0.063 | 4.744 |
| 246.2 | 0.049 | 5.702 | 356.75 | 0.072 | 4.924 |
| 246.9 | 0.03 | 3.285 | 386.9 | 0.072 | 5.114 |
| 247.2 | 0.037 | 3.166 | 387.25 | 0.078 | 5.337 |
| 247.4 | 0.032 | 4.147 | 387.45 | 0.076 | 5.625 |
| 247.7 | 0.047 | 3.804 | 387.75 | 0.074 | 4.755 |
| 247.9 | 0.046 | 3.895 | 388.25 | 0.075 | 2.699 |
| 248 | 0.059 | 3.89 | 388.6 | 0.057 | 3.099 |
| 248.2 | 0.057 | 6.528 | 389 | 0.092 | 5.791 |
| 248.4 | 0.051 | 3.818 | 389.4 | 0.057 | 2.702 |
| 248.6 | 0.04 | 3.989 | 389.6 | 0.055 | 4.704 |
| 248.8 | 0.046 | 9.847 | 390 | 0.058 | 2.879 |
| 249 | 0.047 | 3.893 | 390.5 | 0.05 | 2.002 |
| 249.2 | 0.062 | 8.434 | 390.8 | 0.048 | 3.909 |
| 249.5 | 0.044 | 3.654 | 391.5 | 0.076 | 6.381 |
| 249.8 | 0.05 | 5.071 | 392.1 | 0.064 | 4.146 |
| 250 | 0.06 | 10.274 | 392.45 | 0.066 | 4.101 |
| 250.5 | 0.053 | 4.824 | 392.75 | 0.048 | 1.677 |
| 250.8 | 0.046 | 2.961 | 393.75 | 0.063 | 3.999 |
| 251 | 0.042 | 4.811 | 394.25 | 0.058 | 4.464 |
| 251.3 | 0.047 | 3.36 | 394.6 | 0.072 | 4.703 |
| 251.6 | 0.055 | 5.423 | 395.5 | 0.061 | 4.465 |
| 251.8 | 0.053 | 5.456 | 396 | 0.037 | 3.303 |
| 252 | 0.046 | 7.589 | 396.3 | 0.066 | 4.62 |
| 252.2 | 0.062 | 6.239 | 397.2 | 0.049 | 1.925 |
| 252.4 | 0.054 | 7.994 | 398.1 | 0.056 | 3.556 |
| 252.6 | 0.051 | 3.149 | 398.7 | 0.071 | 4.599 |
| 253 | 0.056 | 5.314 | 399.2 | 0.077 | 5.352 |
| 253.2 | 0.059 | 4.429 | 399.5 | 0.089 | 3.92 |
| 253.5 | 0.066 | 3.329 | 399.7 | 0.064 | 4.224 |
| 253.7 | 0.07 | 2.92 | 399.8 | 0.067 | 4.064 |
| 254 | 0.045 | 3.736 | 399.9 | 0.062 | 4.779 |
| 254.3 | 0.069 | 4.142 | 400.48 | 0.061 | 4.862 |
| 254.5 | 0.061 | 8.988 | 400.72 | 0.066 | 3.621 |
| 254.7 | 0.053 | 3.35 | 401.03 | 0.065 | 5.273 |
| 255 | 0.057 | 5.011 | 401.38 | 0.067 | 5.146 |
| 255.3 | 0.042 | 3.245 | 401.75 | 0.077 | 5.243 |
| 255.5 | 0.058 | 5.777 | 401.98 | 0.072 | 4.971 |
| 255.6 | 0.052 | 4.471 | 402.58 | 0.063 | 4.506 |
| 255.9 | 0.052 | 3.543 | 402.9 | 0.066 | 4.318 |
| 256.2 | 0.048 | 4.005 | 403.22 | 0.068 | 3.118 |
| 256.5 | 0.043 | 3.806 | 403.58 | 0.076 | 3.62 |
| 256.7 | 0.053 | 4.07 | 403.78 | 0.07 | 4.901 |
| 275 | 0.045 | 2.626 | 403.98 | 0.069 | 5.314 |
| 257.3 | 0.036 | 5.244 | 404.18 | 0.062 | 4.349 |
| 257.5 | 0.046 | 3.437 | 404.48 | 0.053 | 3.807 |
| 257.7 | 0.048 | 2.105 | 404.56 | 0.073 | 5.637 |
| 258.3 | 0.046 | 2.024 | 404.78 | 0.082 | 4.614 |
| 258.7 | 0.045 | 5.819 | 404.9 | 0.067 | 5.738 |
| 259 | 0.056 | 4.258 | 404.95 | 0.07 | 5.303 |
| 259.3 | 0.032 | 2.897 | 405.05 | 0.062 | 5.444 |

**SI References**

1 Meng, Q.-R., Wu, G.-L., Fan, L.-G. & Wei, H.-H. Tectonic evolution of early Mesozoic sedimentary basins in the North China block. *Earth Sci. Rev.* **190**, 416-438, (2019).

2 Peng, H. *et al.* Middle Triassic transcontinental connection between the North China Craton and the Paleo-Tethys Ocean. *Commun. Earth Environ.* **5**, 775, (2024).

3 Zhu, R. *et al.* High‐precision Dating and Geological Significance of Chang 7 Tuff Zircon of the Triassic Yanchang Formation, Ordos Basin in Central China. *Acta Geol. Sin.* **93**, 1823-1834, (2019).

4 Zhao, X. *et al.* Recovery of lacustrine ecosystems after the end-Permian mass extinction. *Geology* **48**, 609-613, (2020).

5 Cui, J., Zhu, R., Zhang, Z., Ramezani, J. & Li, Y. High Resolution ID‐TIMS Redefines the Distribution and Age of the Main Mesozoic Lacustrine Hydrocarbon Source Rocks in the Ordos Basin, China. *Acta Geol. Sin.* **97**, 581-588, (2023).

6 Chu, R. *et al.* Nonlinear responses to orbital forcing inferred from an analysis of lacustrine-delta sequences spanning the Middle Triassic (Ladinian) hyperthermal episode in the Ordos Basin, China. *Palaeogeogr. Palaeoclimatol. Palaeoecol.* **628**, 111763, (2023).

7 Wang, B. & Ding, Q. Global monsoon: Dominant mode of annual variation in the tropics. *Dyn. Atmos. Oceans* **44**, 165-183, (2008).

8 Landwehrs, J. *et al.* Modes of Pangean lake level cyclicity driven by astronomical climate pacing modulated by continental position and *p*CO_2_. *Proc. Natl. Acad. Sci. USA* **119**, e2203818119, (2022).

9 Ikeda, M. & Tada, R. Reconstruction of the chaotic behavior of the Solar System from geologic records. *Earth Planet. Sci. Lett.* **537**, 116168, (2020).

10 Trotter, J. A., Williams, I. S., Nicora, A., Mazza, M. & Rigo, M. Long-term cycles of Triassic climate change: a new δ^18^O record from conodont apatite. *Earth Planet. Sci. Lett.* **415**, 165-174, (2015).

11 Laskar, J. *et al.* A long-term numerical solution for the insolation quantities of the Earth. *Astron. Astrophy.* **428**, 261-285, (2004).

12 Laskar, J., Fienga, A., Gastineau, M. & Manche, H. La2010: a new orbital solution for the long-term motion of the Earth. *Astron. Astrophy.* **532**, A89, (2011).

13 Zeebe, R. E. & Lourens, L. J. Geologically constrained astronomical solutions for the Cenozoic era. *Earth Planet. Sci. Lett.* **592**, 117595, (2022).

14 Zaffos, A., Finnegan, S. & Peters, S. E. Plate tectonic regulation of global marine animal diversity. *Proc. Natl. Acad. Sci. USA* **114**, 5653-5658, (2017).
